# Amides as modifiable directing groups in electrophilic borylation†

**DOI:** 10.1039/d2sc06483a

**Published:** 2023-03-15

**Authors:** Saqib A. Iqbal, Marina Uzelac, Ismat Nawaz, Zhongxing Wang, T. Harri Jones, Kang Yuan, Clement R. P. Millet, Gary S. Nichol, Ghayoor Abbas Chotana, Michael J. Ingleson

**Affiliations:** a EaStCHEM School of Chemistry, The University of Edinburgh David Brewster Road Edinburgh EH9 3FJ UK michael.ingleson@edinburgh.ac.uk; b Department of Chemistry and Chemical Engineering, Lahore University of Management Sciences Lahore 54792 Pakistan

## Abstract

Amide directed C–H borylation using ≥two equiv. of BBr_3_ forms borenium cations containing a R_2_N(R′)C=O→B(Ar)Br unit which has significant Lewis acidity at the carbonyl carbon. This enables reduction of the amide unit to an amine using hydrosilanes. This approach can be applied sequentially in a one-pot electrophilic borylation–reduction process, which for phenyl-acetylamides generates *ortho* borylated compounds that can be directly oxidised to the 2-(2-aminoethyl)-phenol. Other substrates amenable to the C–H borylation–reduction sequence include mono and diamino-arenes and carbazoles. This represents a simple method to make borylated molecules that would be convoluted to access otherwise (*e.g. N*-octyl-1-BPin-carbazole). Substituent variation is tolerated at boron as well as in the amide unit, with diarylborenium cations also amenable to reduction. This enables a double C–H borylation–reduction–hydrolysis sequence to access B,N-polycyclic aromatic hydrocarbons (PAHs), including an example where both the boron and nitrogen centres contain functionalisable handles (N–H and B–OH). This method is therefore a useful addition to the metal-free borylation toolbox for accessing useful intermediates (ArylBPin) and novel B,N-PAHs.

## Abstract 2

A sequence of amide-directed borylation followed by amide to amine reduction furnishes useful organoboranes that are otherwise convoluted to access.

## Graphical abstract

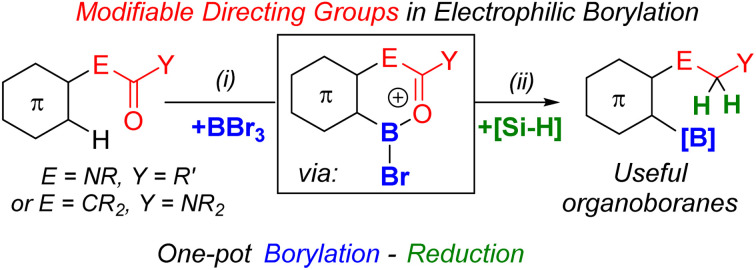

## Introduction

**Fig. 1:**
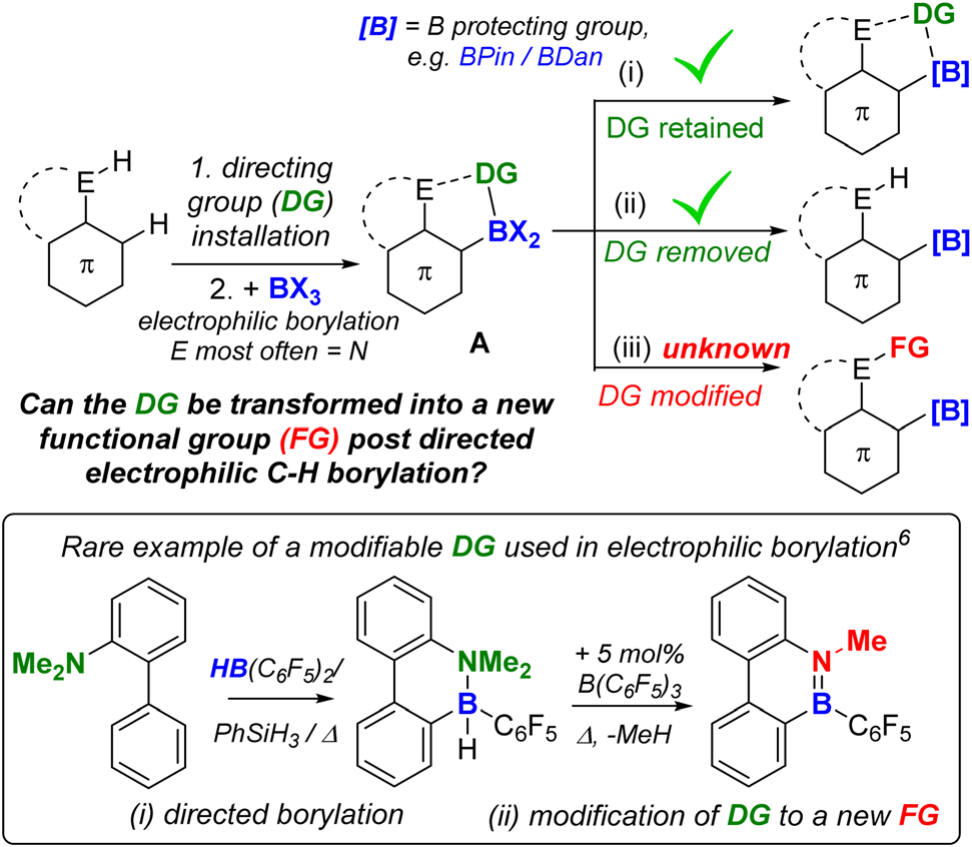
(Top) Three possible fates of DGs post directed borylation. (Bottom) A rare example of a modifiable DG in directed electrophilic borylation to make a B-doped PAH.

Directed C–H borylation is a powerful methodology to install boron moieties onto arenes with high regioselectivity.^1^ This conversion is desirable as organo-boronate esters are powerful intermediates in synthesis.^2^ In this area, notable advances have been made using metal based (principally iridium) catalysts to generate pinacol boronate esters (Ar-BPin).^3^ Recently, it has been demonstrated that directed electrophilic C–H borylation using BX_3_ (X = Cl, Br or I) is a viable metal-free route to install useful boron units, such as BPin, onto arenes.^1*a*,4^ However, this area is less developed than metal-catalysed directed C–H borylation, particularly regarding the utilisation of the directing group (DG) post borylation. To date, the DG is kept intact in the product post electrophilic borylation (Fig. 1(i)) or is removed (Fig. 1(ii)). An attractive alternative is to convert the DG into a new functional group post borylation (Fig. 1(iii)), here it is fulfilling two roles: (a) a DG and (b) a reactive handle for further functionalisation.^5^ Outcome (iii) is significantly underexplored in electrophilic borylation despite its potential to access otherwise challenging to make molecules. To our knowledge, approach (iii) has only been used to make B-doped polycyclic aromatic hydrocarbons (PAHs) in very limited examples (*e.g.* bottom Fig. 1).^6^ Furthermore, approach (iii) has not been used to our knowledge to access synthetically useful organoboranes, such as functionalised ArylBPin species.

**Fig. 2:**
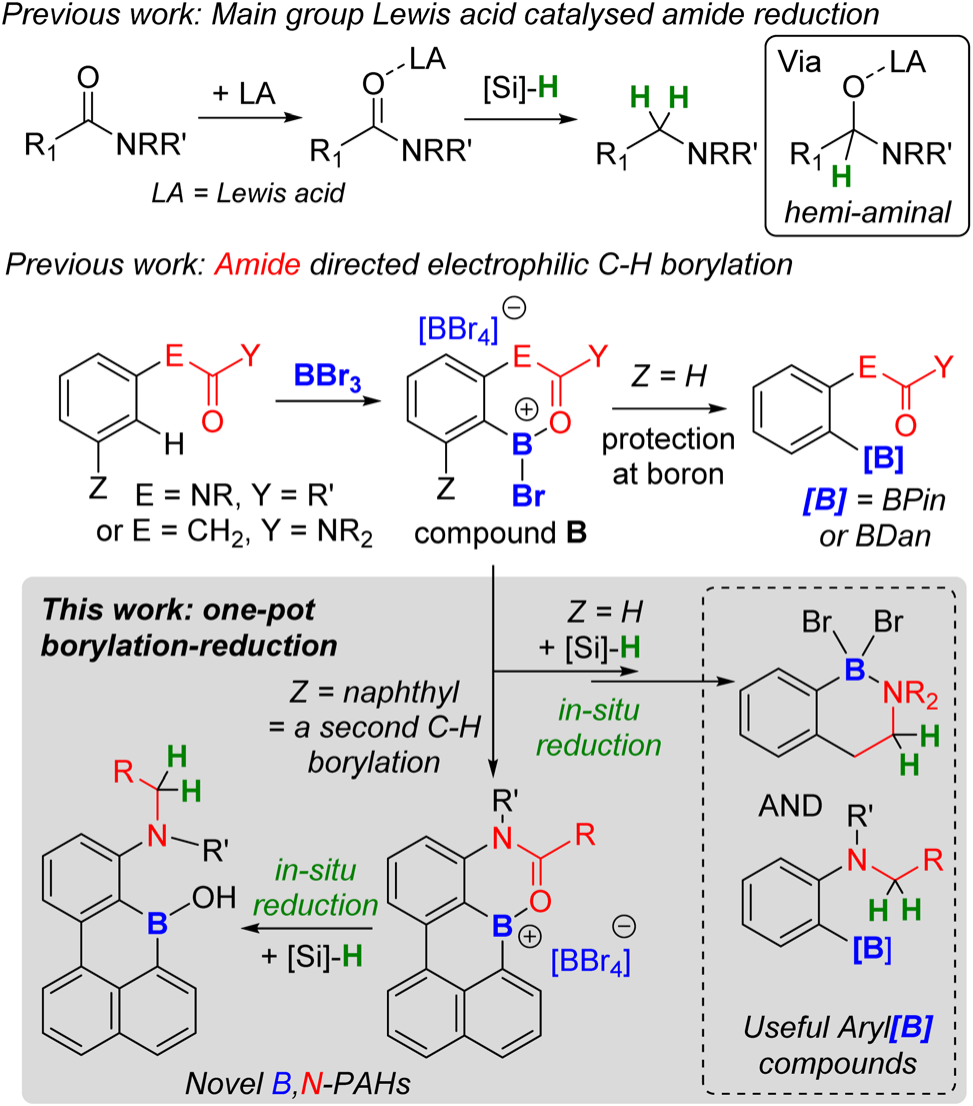
Previous work on: Lewis acid catalysed amide reduction (top); amide directed borylation (middle); and this work (bottom) combining amide directed electrophilic borylation and amide reduction to make novel borylated compounds.

In directed electrophilic borylation, the Lewis basic DG binds the boron Lewis acid (*e.g.*, BX_3_) enabling subsequent access to a borenium cation (a three coordinate borocation) that then effects C–H borylation.^1*a*,4^ Several DGs used in electrophilic borylation contain unsaturated units, *e.g.*, C=O, and in the primary product formed post C–H borylation, *e.g.*A (Fig. 1) or B (Fig. 2), the DG remains bound to a Lewis acidic boron centre.^4^ Thus, the DG will be activated, *e.g.* at the carbonyl carbon in B, towards reaction with nucleophiles. For example, Lewis acid–carbonyl adducts are effectively reduced by silanes or boranes.^7^ These reductions can be selective, for example using silanes and B(C_6_F_5_)_3_ as Lewis acid activator, amides are reduced to amines *via* a hemi-aminal (Fig. 2 top).^8^ Therefore, the primary products from amide directed electrophilic borylation (*e.g.*B) should be amenable to reduction to the amine. This would be notable if the C–B unit remained intact as it would generate a new set of borylated compounds from a common intermediate (*e.g.*B).

Herein we report that hydrosilanes reduce the primary products from amide directed electrophilic borylation to amine containing borylated products. The amide to amine reduction process is applicable to a range of substrates including phenylacetyl, arylamine, and carbazole based amides along with more complex doubly borylated systems. This process is a rare example of using a modifiable directing group in electrophilic borylation and represents a new route to access synthetically useful arylboranes and novel *ortho*-B,N-containing PAHs (an area of significant current interest).^9^

## Results and discussion

### Borylation–reduction of phenyl-acetyl derivatives

**Fig. 3:**
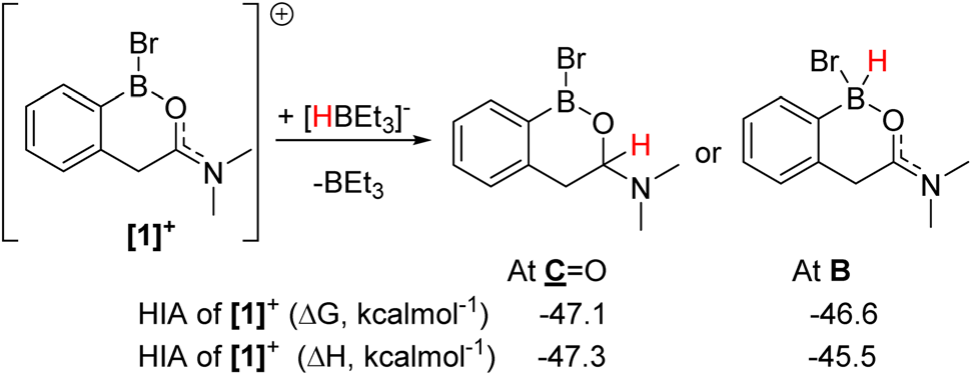
HIA at boron and the carbonyl carbon of [1]^+^.

**Fig. 4:**
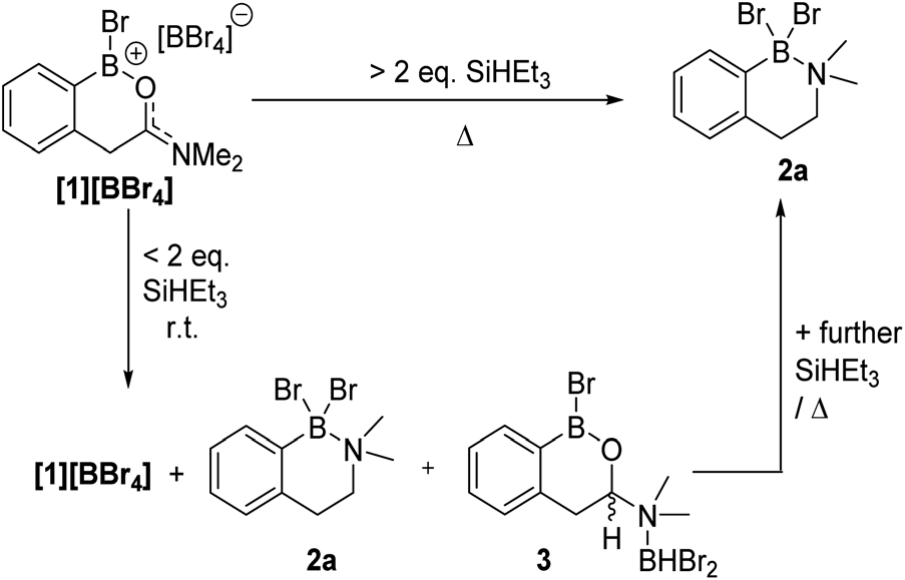
Formation of amine-borane 2a using silanes *via* hemi-aminal 3.

The formation of [1]^+^ (Fig. 3) as the [BBr_4_]^−^ salt by directed electrophilic C–H borylation has been previously reported.^10^ Borenium [1][BBr_4_] was selected to start the reduction studies as the product from amide to amine reduction would form an *ortho* borylated PhCH_2_CH_2_NMe_2_ species predisposed to form a six-membered boracycle (by N→BY_2_ dative bond formation, Y = H or Br, *e.g.*2a, Fig. 4). This boracycle should be bench stable based on previous work,^11^ thereby facilitating isolation and identification of the exocyclic Y substituents on boron (note the identity of Y will control the equivalents of reductant needed). The identity of Y is challenging to predict *a priori*, as in [1]^+^ both boron and the carbonyl carbon are electrophilic and could accept hydride. To assess Lewis acidity the hydride ion affinity (HIA) relative to BEt_3_ was calculated using the previously reported methodology (at M06-2X/6-311G(d,p)/PCM(DCM) level, this level is used throughout this work).^12^ This revealed (Fig. 3) that both positions have comparable Lewis acidity towards hydride, thus hydride transfer is feasible to both carbon and boron. Note, these HIAs are high, being greater than B(C_6_F_5_)_3_,^12^ and comparable to the carbon based Lewis acid *N*-methyl-2-phenyl benzothiazolium cation,^13^ with both these Lewis acids able to activate hydrosilanes.^13,14^

While a range of boranes/borohydrides gave complex mixtures in the reduction of [1][BBr_4_], the use of hydrosilanes resulted in more selective reduction, with SiHEt_3_ providing good results (note modification in the hydrosilane provided limited change in outcome, see Table S1†). The addition of one equiv. of SiHEt_3_ to [1][BBr_4_] at ambient temperature resulted in three major new resonances in the *in situ*^11^B NMR spectrum at *δ*_11B_ = 40.4, 4.5 and −2.7, the latter was a doublet with a ^1^*J*_B–H_ = 156 Hz. The ^1^H NMR spectrum contained a diagnostic doublet of doublets (*δ*_1H_ = 5.60, *J* = 12.2, 3.3 Hz) assigned to the hemi-aminal C–H in 3 (Fig. 4) in addition to resonances further upfield for the diastereotopic PhCH_2_ protons. However, the aromatic region showed there are two new products formed under these conditions along with significant [1][BBr_4_] remaining. Additional studies (*vide infra*) revealed the second product to be the amine borane 2a. The complete consumption of [1][BBr_4_] required at least two equiv. of SiHEt_3_ and produced a mixture of 2a and 3 at ambient temperature (Fig. S7†).

**Fig. 5:**
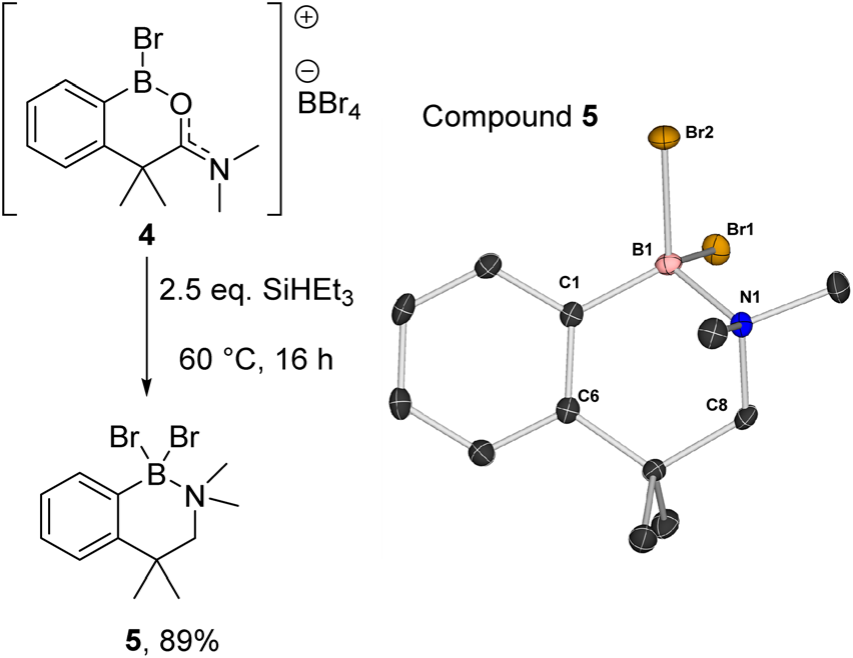
(Left) Formation of amine borane 5. (Right) The solid-state structure of 5, ellipsoids at the 50% probability and hydrogens omitted for clarity.

Compound 2a could be formed as the sole soluble product starting from [1][BBr_4_] by using >2 equiv. SiHEt_3_ and heating. 2a displays an ^11^B NMR singlet at *δ*_11B_ = +4.5 (precluding a BH_2_ formulation as the BH_2_ congener of 2a is a triplet at *δ*_11B_ −5.2),^11^ while the ^1^H NMR spectrum showed two aliphatic triplets consistent with an ArCH_2_CH_2_ unit formed from reduction of amide to amine. Further confirmation of the formation of an amine→BBr_2_ species was forthcoming from the reduction of previously reported 4 (ref. 10) with SiHEt_3_ (Fig. 5). On heating this formed compound 5, displaying a *δ*_11B_ = 5.0, and a singlet at *δ*_1H_ = 3.5 for the CH_2_ unit. 5 could be isolated as a crystalline solid which enabled X-ray diffraction studies to corroborate the formulation. Coordination of the amine to the boron centre in 5 (N–B = 1.604(3) Å) results in a pyramidalised boron centre (ΣBr2–B1–C1/Br1–B1–C1/Br1–C1–Br2 = 329.72(3)°), with this strong dative bond allowing the amine boranes 2a and 5 to be handled on the bench for short periods. Finally, it should be noted the formation of a BBr_2_ unit during reduction is consistent with a 2 : 1 Si–H : [1][BBr_4_] stoichiometry leading to complete amide to amine reduction.

**Scheme 1:**
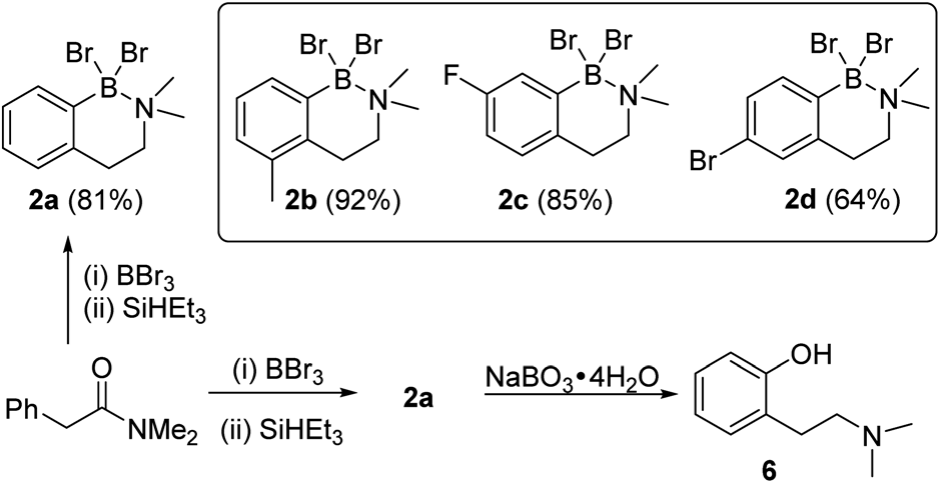
(Top) Borylation reduction tolerating both electron donating and withdrawing groups and substituents at *ortho*, *meta* and *para* positions in the starting material (isolated yields in parentheses). (Bottom) Conversion of *N*,*N*-Me_2_-phenylacetylamide into 2-(2-Me_2_N-ethyl)phenol 6.

The amine boranes also can be formed in a one-pot borylation–reduction process, and starting from *N*,*N*-Me_2_-phenylacetylamide compound 2a was formed in 81% isolated yield (Scheme 1). To determine if this process tolerated electron donating (Me) and electron withdrawing groups (F and Br) three derivatives each functionalised at a different arene position were explored. These led to the formation of 2b–2d in good yield (Scheme 1), albeit requiring modified borylation reaction times (see ESI Section 3†) due to the different electronic effects of the substituents consistent with our previous report.^10^ To determine if the N–B dative bond in 2a prohibits subsequent transformations, the oxidation of 2a was explored. Shi and co-workers have reported that ArylBBr_2_ units that are chelated by amide directing groups can be oxidised using NaBO_3_·4H_2_O.^15^ Analogous conditions were applied for the oxidation of 2a and this led to the full consumption of 2a and formation of the corresponding phenol, 6, as the only new species observed by NMR spectroscopy (Fig. S29†). This indicates that despite the N→B dative bond, these amine boranes are useful intermediates. This is noteworthy as (2-aminoethyl)-phenols are common motifs found in bioactives (*e.g.* psilocin),^16^ while 6 represents an *ortho*-tyramine derivative that can be accessed in just two steps from simple precursors.

With the identity of Y and utility of the amine-boranes 2 confirmed our attention turned to the hemi-aminal, assigned as 3. Multiple attempts to form 3 as the sole boron containing species using a range of silanes/conditions failed, with varying quantities of 2a/[1][BBr_4_] always present, furthermore 2a and 3 proved intractable. However, the identity of 3 is supported by comparison to Me_3_N→BHBr_2_ which displays a *δ*_11B_ = −0.7 doublet with an identical ^1^*J*_B–H_ coupling constant (156 Hz) to that observed in 3.^17^ Furthermore, the −2.7 and 40.4 ppm ^11^B resonances increased in intensity concomitant with each other and with the *δ*_1H_ = 5.60 doublet of doublets. The *δ*_11B_ = 40.4 is consistent with boron in an ArB(OR)Br environment.^18^ Finally, it should be noted that leaving mixtures containing 3 for longer reaction times led to the conversion of 3 into 2a, suggesting 3 is an intermediate in the production of 2a.

**Scheme 2:**
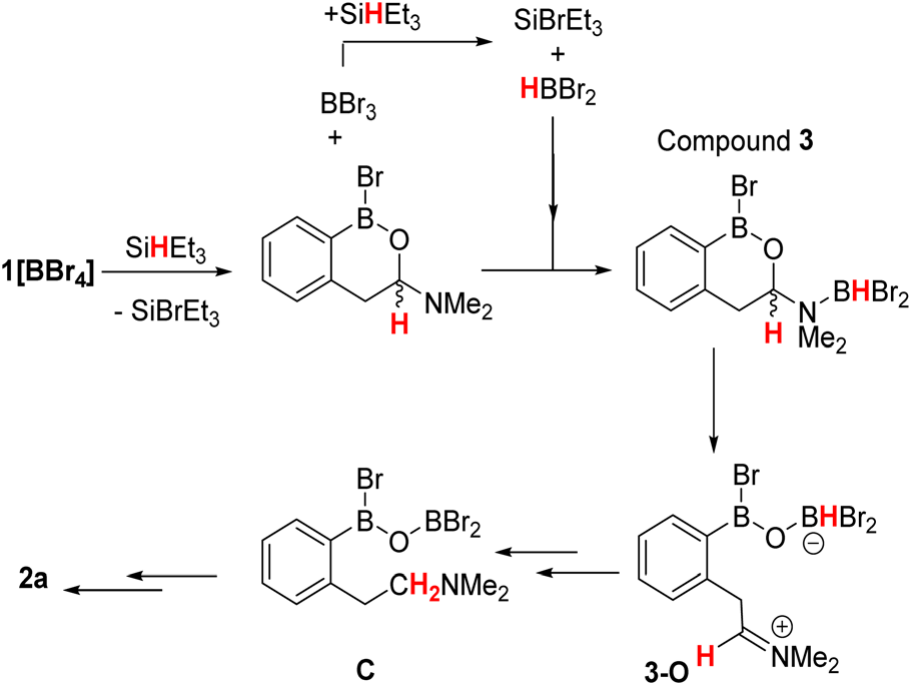
Proposed pathway for the reduction of [1]^+^ to 2a*via*3, 3-O and C.

Based on these observations a mechanism for the formation of 2a can be proposed (Scheme 2, note only a single new resonance in the ^29^Si NMR spectrum was observed at *δ*_29Si_ = 37.9 consistent with SiBrEt_3_). The initial reduction of [1][BBr_4_] would produce the hemi-aminal, with SiBrEt_3_ and BBr_3_ the by-products. BBr_3_ would then react rapidly with SiHEt_3_ producing SiBrEt_3_ and HBBr_2_,^19^ the latter is observed bound to the hemi-aminal nitrogen in compound 3 by NMR spectroscopy (*vide supra*). In the absence of additional silane, the only reducing equivalent now present is HBBr_2_ and we propose this can form the O-bound isomer of 3, termed 3-O (Scheme 2, bottom right). Compound 3-O was calculated to be more stable than 3 by Δ*G* = 8.4 kcal mol^−1^, thus 3 is the kinetic product. The calculated structure of 3-O has the C–O bond already cleaved and instead contains an iminium moiety that is primed for reduction by the B–H unit to furnish C. Support for formation of an ArylB–O–BBr_2_ unit (as shown in C) comes from studies on other related substrates (*vide infra*). Finally, boron substituent scrambling and N→B dative bond formation then will convert C into 2a.

**Scheme 3:**
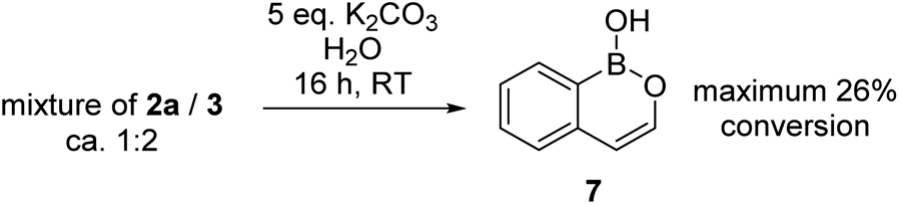
Formation of boron-enolate 7 from 3.

**Scheme 4:**
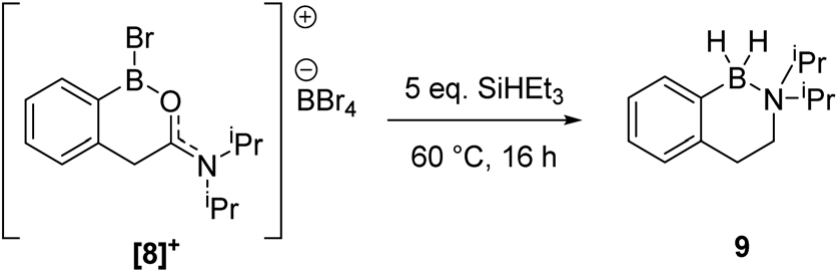
Reduction of [8][BBr_4_] to form compound 9.

During attempts to isolate the hemi-aminal the boron enolate 7 was isolated (Scheme 3), with related boron enolates previously shown to be useful intermediates.^20^ However, under a range of conditions 7 could only be formed in low (max. 26%) conversion from [1][BBr_4_]. In an attempt to form a hemi-aminal selectively the bulk around N was increased by replacing NMe_2_ with N^i^Pr_2_ ([8][BBr_4_], Scheme 4), hypothesising that the extra bulk may preclude HBBr_2_ coordination to N and O and thus prevent the second hydride transfer step. However, using this bulkier amide led to initial hydride transfer to boron, confirming that reduction can occur at boron as well as at carbon, consistent with the HIA calculations. The only reduced product isolable clean starting from [8][BBr_4_] was compound 9 (formed as the major species when using 5 equiv. silane), with no hemi-aminal observed at any point by *in situ* NMR spectroscopy. Compound 9 is formed from hydride transfer to boron, and then slower amide reduction to the amine. Similar amine-borane products were reported by Vedejs and co-workers and were also made by electrophilic borylation of PhCH_2_CH_2_N(R)_2_BH_3_, however this required stoichiometric amounts of the expensive reagent [Ph_3_C][B(C_6_F_5_)_4_].^11^

### Borylation–reduction of aniline derivatives

**Fig. 6:**
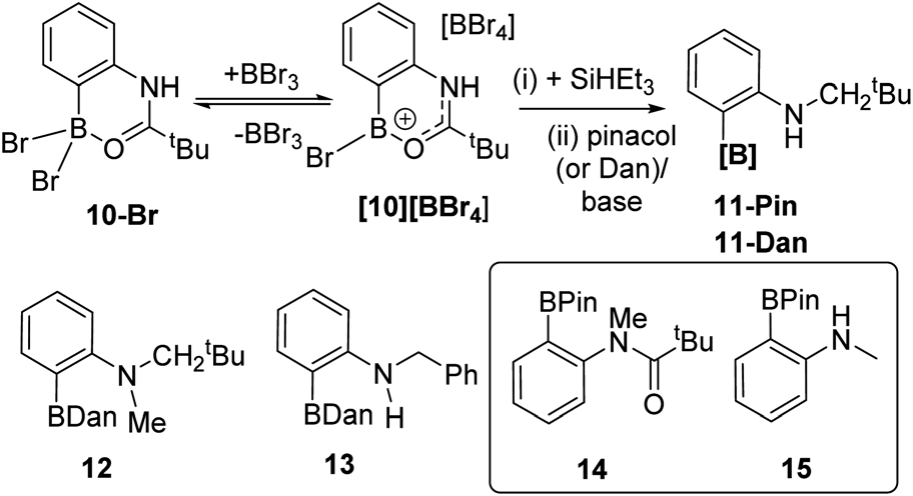
Top, reduction of [10][BBr_4_]. Bottom left, additional substrates isolated as Dan derivatives. Inset, substrate 14 used in Zn catalysed amide reduction, and 15 one of the products from that process.

With a borylation–reduction process in hand its applicability to other substrates was investigated. *N*-Pivaloyl anilines also produce primary products from directed electrophilic borylation using BBr_3_ that contain a boron Lewis acid coordinated to the amide carbonyl (such as 10-Br/[10][BBr_4_], Fig. 6).^21^ It should be noted that as reported for the phenylacetyl derivatives,^10^10-Br and [10][BBr_4_] exist in equilibrium, with the position of the equilibrium favouring [10][BBr_4_] when two equiv. of BBr_3_ are used in the C–H borylation step. This is important as it was found that conditions where borocations, *e.g.*[10][BBr_4_], are the dominant species (over neutral species *e.g.*10-Br) give better amide reduction outcomes using SiHEt_3_ (see Fig. S32†). This is attributed to the carbonyl carbon in borenium cations such as [10][BBr_4_] being more electrophilic than that in neutral derivatives (*e.g.*10-Br) which will facilitate reduction. Attempts to monitor the reduction of [10][BBr_4_] by *in situ* NMR spectroscopy were not informative as these reactions formed complex mixtures from which no intermediate borane could be identified. However, the addition of pinacol or 1,8-diaminonaphthalene (Dan) to these mixtures formed 11-Pin and 11-Dan, respectively (Fig. 6), as the major boron compounds (by NMR spectroscopy *versus* an internal standard, Fig. S33†). Other aniline derivatives, including a tertiary amide and an *N*-benzoyl congener, also were amenable to this borylation/reduction process. The products were protected and isolated as the Dan derivatives, 11-Dan, 12 and 13, due to the enhanced stability of these BDan congeners relative to the BPins (the latter proved sensitive to column chromatography). The ability to vary the protecting group installed on boron (*e.g.* Pin or Dan) is an attractive feature of this approach and is distinct to iridium-catalysed routes which are limited generally to BPin due to the use of B_2_Pin_2_.^22^ Furthermore, attempts to access these products cleanly by amide reduction of the BPin containing arylamide 14 (Fig. 6 inset), was not effective. The reduction of 14 under mild conditions (Zn(OAc)_2_/(EtO)_3_SiH)^23^ led to the formation of multiple ArylBPin species, with their ratios dependent on the equiv. of silane used. The major products were the desired 12-Pin and 15 (by *in situ* NMR spectroscopy, Fig. S34†), but conditions leading to clean formation of 12-Pin or 15 were not identified.

**Fig. 7:**
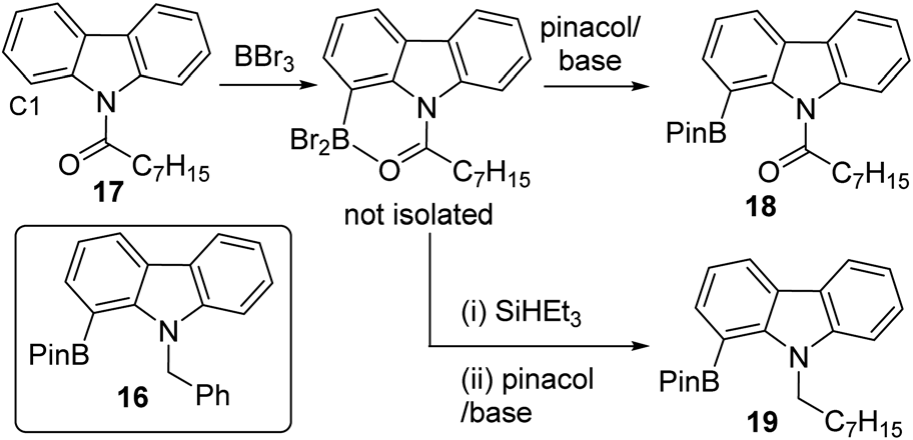
The C1 borylation–reduction of *N*-acyl carbazoles.

To expand the borylation–reduction scope it was applied in the previously reported *N*-benzoyl directed C1 borylation of carbazole.^21^ This one pot borylation–reduction process enabled formation of 16 in 61% yield (Fig. 7, inset), with 16 containing an *N*-benzyl group that can be readily removed. Carbazoles, including C1-functionalised derivatives,^24^ are prevalent in organic electronics where they are often *N*-substituted with alkyl chains to enhance solubility. Therefore, the applicability of borylation–reduction to access *N*-alkyl-1-borylated-carbazoles was assessed. N–H-carbazole was functionalised to form 17 and this derivative underwent acyl directed borylation with BBr_3_ and protection at B to afford 18 in 93% isolated yield. This in itself is notable as it is the first report of an enolisable acyl unit being successfully used in amide directed electrophilic C–H borylation to our knowledge. Applying borylation–reduction to 17 afforded 19 in 53% yield. The formation of 19 from 17 in one-pot represents a streamlined route to access useful C1-borylated-*N*-alkylated carbazoles compared to previous methods (which proceed *via* formation of the C1-brominated precursor and then lithiation–borylation).^24^

**Scheme 5:**
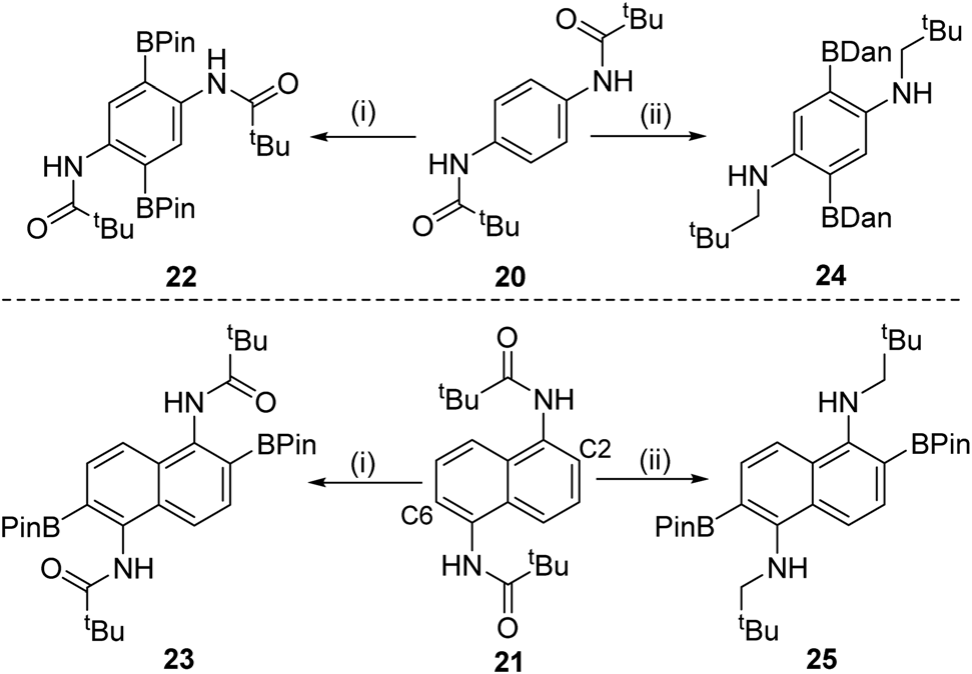
(i) Borylation with BBr_3_ followed by protection at boron; (ii) borylation with BBr_3_, reduction with SiHEt_3_ and then protection.

Doubly functionalised diamino-arenes also are useful building blocks,^25^ therefore the extension of this process to double amide directed C–H borylation products was investigated. Substrates 20 and 21 (Scheme 5) were synthesized *via* standard methodologies and found to undergo double directed electrophilic borylation using BBr_3_ to afford 22 and 23 in good isolated yields (80 and 83%, respectively). Incorporating a reduction step prior to protection at boron enabled formation of 24 and 25 in moderate yields (24 and 40%, respectively). The double borylation of the naphthyl derivative 21 at the C2/6 positions in preference to the *peri*-positions was confirmed by single crystal X-ray diffraction studies on compound 25 (see Fig. S119†). The solid-state structure of 25 is unremarkable, excluding the fact that the nitrogen atoms are pyramidalised (*e.g.* C–N–C = 115.14(7)°), something we attribute to pyramidalisation being required to permit the N–H⋯OB_Pin_ intramolecular hydrogen bond (2.024(9) Å) that is present in the structure of 25. The selective formation of the 2,6-diborylated isomers (23 and 25) is consistent with the preference for forming six membered boracycles over seven membered during electrophilic borylation (the latter would be required to borylate at the *peri* position of 21).^1*a*^

### Borylation–reduction of diaryl borane derivatives

**Fig. 8:**
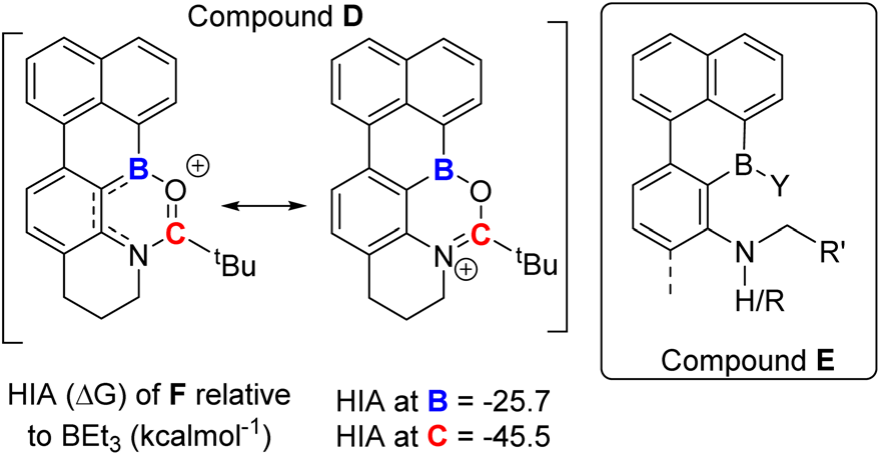
Lewis acidity of diaryl-borane derived cations.

While a range of substituents on the amide are tolerated in borylation–reduction all the examples above have very similar substituents at boron in the borenium cation being reduced, specifically in each case boron is substituted with an aryl, a bromine and a carbonyl. We were interested in determining if altering one substituent at boron in the borocation was compatible with selective amide to amine reduction. Therefore, a directed C–H borylation was targeted where a single boron centre performs two C–H borylations to generate a borocation substituted with two aryl groups (*e.g.* compound D, Fig. 8 left). If reducible this amide would form species E (right, Fig. 8) and compounds related to E are of current interest as they are B,N containing PAHs that have a reactive site at B and at N thus would facilitate further reactivity *e.g.* for making rigid “pre-organized” intramolecular FLPs^26^ or emissive B,N-PAHs.^27^

However, embedding Lewis acidic centres into rigid PAHs can dramatically alter their reactivity.^28^ Therefore the HIA was calculated at boron and carbon for D. This revealed the HIA at boron in D is significantly reduced relative to that in 1^+^ (Fig. 3), while the carbonyl carbon possesses a high HIA (Fig. 8), comparable to that in 1^+^. The disparity between the HIA values at boron and carbon in D is attributed to the *N*-(alkyl)aryl amide structure (in contrast to the *N*-dialkyl amide unit in 1^+^) which will result in the N lone pair being delocalised to some extent into the extended π system (Fig. 8, left-hand resonance structure). This is supported by the calculated structures, which revealed a longer (O)C–N bond and a shorter aryl–B bond in D relative to that in 1^+^, consistent with the relative Lewis acidity at boron and carbon in D. Regardless, the high HIA at carbon in D indicates reduction of amide to amine is feasible.

**Fig. 9:**
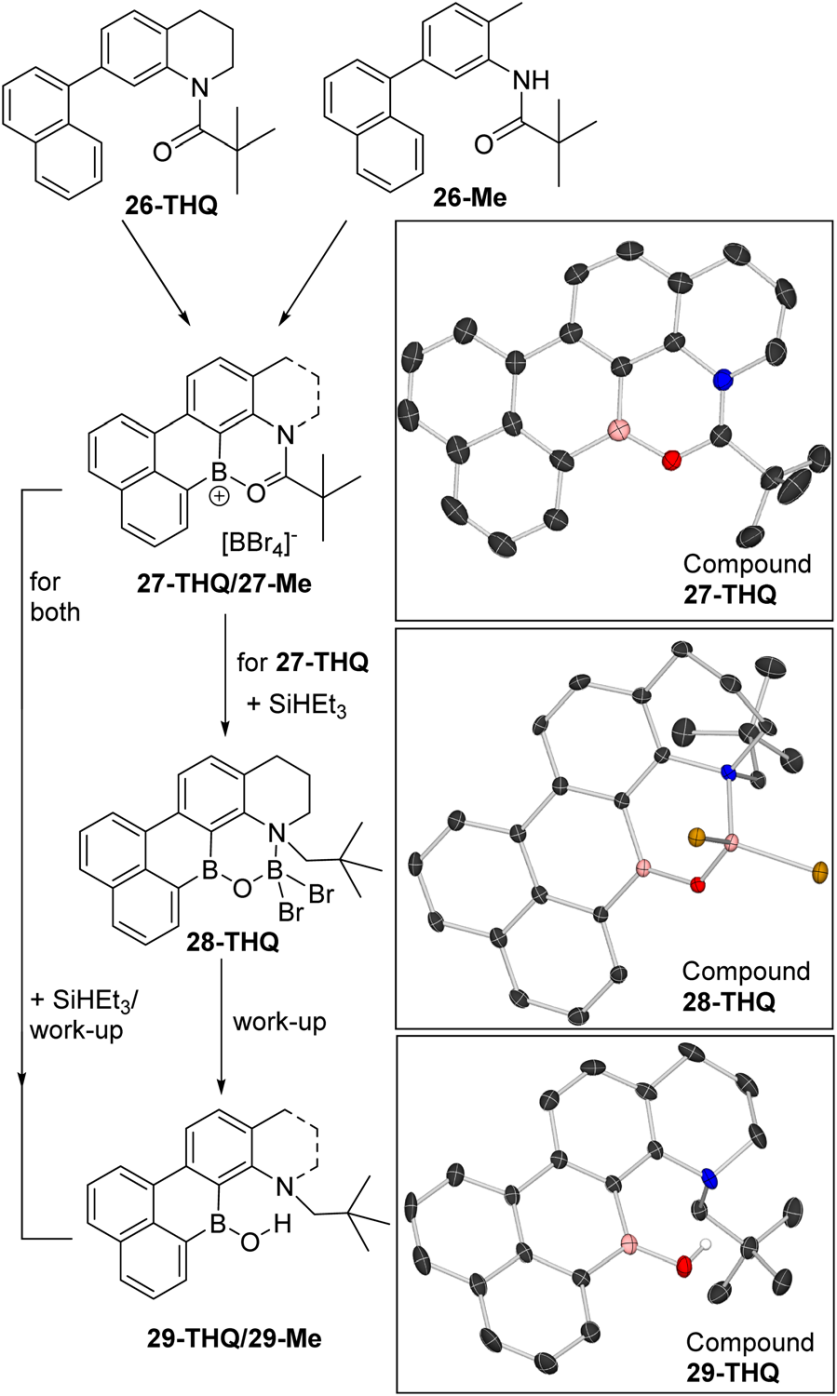
Double borylation–reduction–hydrolysis to form B,N-containing PAHs 29-THQ and 29-Me. Right, solid state structures, ellipsoids at 50% probability and most hydrogens omitted for clarity.

Two precursors, 26-THQ and 26-Me (top, Fig. 9), were synthesised in two steps from commercial precursors. Both are designed so that amide directed C–H borylation can only incorporate the boron centre in the correct position for a second C–H borylation at the naphthalene *peri* position. The latter step is expected to be facile based on our previous work.^29^ Indeed, the addition of two equiv. BBr_3_ to 26-THQ in DCM at room temperature led to the precipitation of 27-THQ, with the formulation supported by single crystal X-ray diffraction analysis (*vide infra* for discussion). Similarly, the addition of two equiv. BBr_3_ to 26-Me led to precipitation of a yellow solid, assigned as 27-Me. Both 27-THQ and 27-Me were sufficiently soluble in CD_2_Cl_2_ to produce weak NMR spectra that displayed resonances consistent with their formulation, *e.g. δ*_11B_ = +36.5 (broad) and −24.4 for 27-THQ and +36.0 (broad) and −24.4 for 27-Me. The sharp −24.4 ppm resonance is due to [BBr_4_]^−^ and confirms borenium ion formation, as expected based on the low (relative to 1^+^) Lewis acidity calculated at boron in D. The ^1^H NMR spectra for both compounds 27 contained eight 1H integral resonances in the aromatic region as expected.

The addition of two equiv. of SiHEt_3_ to suspensions of 27-THQ led to gradual dissolution and formation of a single major product containing a three and a four-coordinate boron centre (by NMR spectroscopy). This species was isolated by crystallisation, and analysis by X-ray diffraction revealed formation of 28-THQ. In 28-THQ the amide has been reduced to an amine, but the product has not undergone B–O cleavage. Instead the B and N centres are bridged by a [O–BBr_2_]^−^ moiety, presumably derived from an O–BHBr_2_ intermediate (as proposed in the conversion from 3 to C, Scheme 2). The formulation as 28-THQ from X-ray diffraction studies is fully consistent with the ^11^B NMR spectrum which contained resonances at +40.6 and +6 ppm, for a three and a four coordinate boron centre, respectively. Due to the chiral N in 28-THQ the methylene protons are diastereotopic in the ^1^H NMR spectrum, while there remain eight aromatic resonances.

Starting from 27-Me, *in situ* NMR spectroscopy revealed a complex mixture is formed post reduction with SiHEt_3_, but the mixture can be converted into the borinic acid 29-Me in 78% yield by treatment with aqueous base. Compound 29-Me displayed a *δ*_11B_ at +41.1 in the expected region, while a *δ*_1H_ singlet at +11.95 was observed for the B–OH which is consistent with a borinic acid O*H* in a rigid environment containing a BO⋯H–N hydrogen bond.^30^29-THQ can be accessed analogously from 28-THQ by aqueous basic work up, or in a one pot process directly from 27-THQ in 67% isolated yield. 29-THQ has a comparable *δ*_11B_ (+40.9 broad) and *δ*_1H_ (+11.92) for the B–OH unit. The formation of compounds 29 demonstrates that it is possible to access B/N containing PAHs using the directed borylation–reduction methodology, including an example containing the modifiable groups, N–H and a B–OH as in 29-Me.

**Fig. 10:**
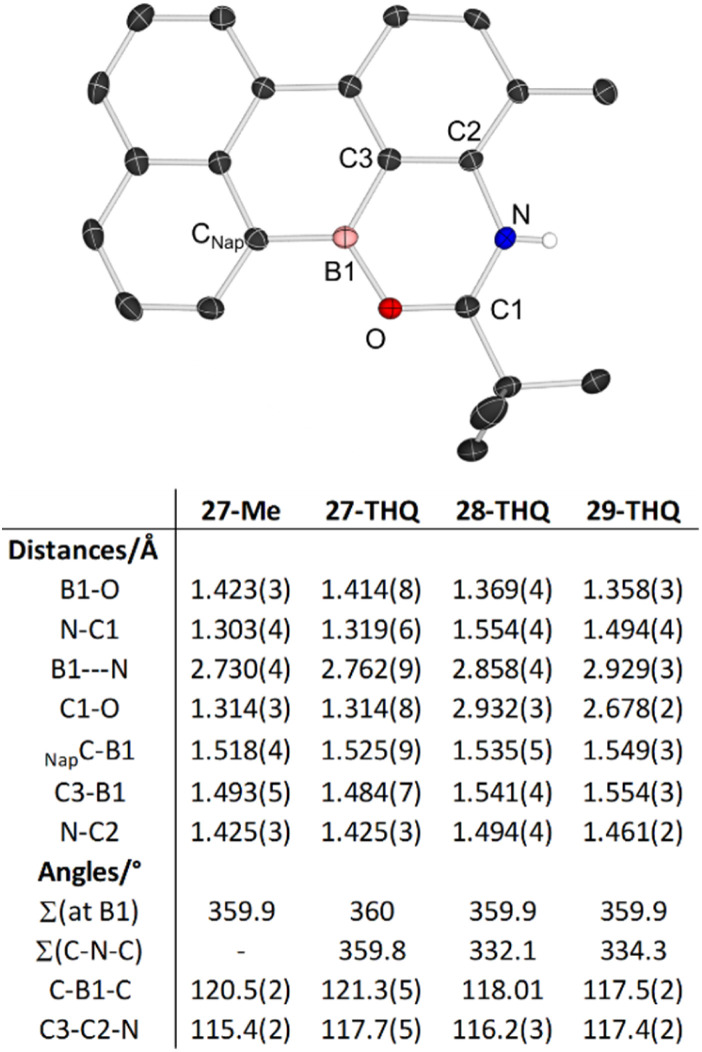
Top, the solid-state structure of the cationic portion of 27-Me, ellipsoids at 50% probability, most hydrogens omitted for clarity. Bottom, select metrics.

In addition to 27–29-THQ, 27-Me also was crystallised and analysed by X-ray diffraction studies (Fig. 10, top). Analysis of the key metrics for 27-Me revealed them to be closely comparable to 27-THQ (Fig. 10 bottom), therefore the structural discussion only focuses on the 27–29-THQ series. In all the structures the boron centre is effectively trigonal planar, with the shortest anion⋯cation contact in 27-THQ long at Br⋯B = 3.610(6) Å. 27-THQ can be viewed as having a core that is a main group analogue of perylene and the all sp^2^ pentacyclic core in 27-THQ is effectively planar (max. deviation from the plane of the pentacycle = 0.12 Å), with the geometry at N effectively trigonal planar. This is in contrast to the geometry at N in 28- and 29-THQ, with N significantly pyramidalised in both. While this is expected for 28-THQ, for 29-THQ the N is pyramidalised due to the N⋯H–OB interaction. Other noteworthy metrics include: (i) the B–O distance being longest in the datively bonded system, 27-THQ; (ii) significant (0.17 Å) variation in the *trans*-annular B⋯N distances, indicating some flexibility in this system despite its fused nature; (iii) the B1–C3 distance being significantly shorter in cationic 27-THQ, whereas the C_Nap_–B distances varies to a much lesser degree across the series. This indicates some delocalisation from the N to the Lewis acidic boron centre in 27-THQ. Finally, the effect on stability (to protodeboronation) of embedding the boron centre into a fused amino-substituted PAH was assessed. Reactivity studies confirmed that 27-THQ and 27-Me are significantly more robust towards protodeboronation than the borinic acid Ph_2_BOH and the non-fused 1-BPin-2-NR_2_-C_6_H_4_ systems. For example, in contrast to the latter two compounds 27-THQ does not react on prolonged exposure to basic or acidic aqueous solutions (by NMR spectroscopy).

In conclusion, this work demonstrates that amides can function as modifiable directing groups in electrophilic C–H borylation. Post borylation using BBr_3_, the addition of an hydrosilane to the crude reaction mixture enables reduction of the amide to an amine while maintaining the C–B bond(s). This process proceeds *via* borenium cations that are Lewis acidic at both boron and the carbonyl carbon, but hydride transfer occurs selectively to carbon (in the absence of significant bulk on the amide). Post reduction, the products are useful as the C–B unit can be oxidised *in situ* to the respective phenol or protected at boron to give synthetically ubiquitous aryl boronate esters (as BPin or BDan derivatives). Notably, this amide directed electrophilic borylation–reduction process represents a simple route to form aryl boranes (*e.g.* compound 19) that can be made currently only *via* convoluted routes. By applying borylation–reduction to a range of substrates an initial mechanistic picture has emerged that indicates a stepwise reduction, where the second reduction step proceeds not from a hydrosilane, but from a HBBr_2_ species made *in situ*. Notably, amide reduction is applicable to a range of amide→B containing borenium cations, with substituent variation tolerated at both the amide and around boron. The latter enables borylation–reduction to be used to access novel B,N-functionalised PAHs that contain reactive functionalities on both N and B. This will facilitate further functionalisation and thus enable application of these materials in FLP catalysis and as emissive organic materials, both topics currently under exploration in our laboratory.

## Data availability

The data supporting this article has been uploaded as part of the ESI.†

## Author contributions

MI, SI and MU conceived the research concept and aims and analysed all data. SI and MU performed the majority of the synthetic work and the majority of the analytical components of this project. IN, ZW, HJ, and CM also performed the synthesis and characterisation of a number of compounds reported in this manuscript. GN and MU collected and solved all the crystal structures. KY performed the computational analysis. Combined, MI, MU, SI and GC drafted, reviewed and edited the manuscript.

## Conflicts of interest

There are no conflicts to declare.

## Supplementary Material

SC-014-D2SC06483A-s001

SC-014-D2SC06483A-s002
