# Supplementary material for: Amides as modifiable directing groups in electrophilic borylation
Source: Chem Sci. 2023 Mar 15;14(14):3865–72. doi: 10.1039/d2sc06483a (PMC10074396; doi:10.1039/d2sc06483a)
Supplement: SC-014-D2SC06483A-s001 [file SC-014-D2SC06483A-s001.pdf]

Electronic Supplementary Material (ESI) for Chemical Science.
This journal is © The Royal Society of Chemistry 2023

Supporting information for

Amides as Modifiable Directing Groups in Electrophilic Borylation

S. A. Iqbal[a‡], M. Uzelac[a‡], I. Nawaz[a,b], Z. Wang[a], T. H. Jones[a], K. Yuan[a], C. R. P. Millet[a], G. S. Nichol,[a] G. Abbas Chotana[b] and M. J. Ingleson*[a]

a School of Chemistry, University of Edinburgh, Edinburgh, EH9 3FJ, UK

b. Department of Chemistry and Chemical Engineering, Lahore University of Management Sciences, Lahore 54792, Pakistan

Contents

1 General considerations 2

2 Amide Synthesis 3

3 Borylation – reduction of 2-phenylacetamides 10

4 Borylation-Reduction of Aniline Derivatives 30

5 Borylation of Carbazole Compounds: 43

6 Double Borylation – reduction studies 50

7 Synthesis and characterisation of B,N-PAH derivatives 64

7.1 Two step synthesis of *N*-pivaloyl-7-naphthyl-1,2,3,4-tetrahydroquinoline (Compound **26-THQ)** 64

7.2 Directed borylation of *N*-pivaloyl-7-naphthyl-1,2,3,4-tetrahydroquinoline (**27-THQ**) 67

7.3 Reduction of **27-THQ** with triethylsilane to afford **28-THQ** 72

7.4 Isolation of **29-THQ** 74

7.5 Synthesis of *N*-pivaloyl-2-methyl-5-naphthylaniline (**26-Me**) 79

7.6 Directed borylation of *N*-pivaloyl-2-methyl-5-naphthylaniline (**27-Me**) 81

7.7 Reduction of **27-Me** and isolation of **29-Me** 86

8 X-Ray Crystallography 92

9 Computational details 97

10 References 111

1 General considerations

Unless otherwise indicated all manipulations were conducted under inert conditions either using standard Schlenk techniques or in a MBraun UniLab glovebox (< 0.1 ppm H_2O / O_2). All chemicals were purchased from commercial sources and used as received without further purification unless otherwise stated. BBr_3 solution (1 M in DCM) was transferred and stored in J Young tap fitted ampoules. Solvents were dried using an Innovative Technology SPS system and stored over activated molecular sieves. NMR spectra were recorded on Bruker Avance III 400 MHz and 500 MHz spectrometers. Chemical shifts are reported as dimensionless δ values and are frequency referenced relative to residual protio- impurities in the NMR solvents for ^1H and $^{13}C\{^1H\}$ respectively, while ^{11}B and ^{19}F shifts are referenced relative to external $BF_3{\cdot}Et_2O$ and hexafluorobenzene, respectively. Coupling constants J are given in Hertz (Hz) as positive values regardless of their real individual signs. The multiplicity of the signals is indicated as "s", "d", "t" "q" "pent", "sept" or "m" for singlet, doublet, triplet, quartet, pentet, septet or multiplet, respectively.

Column chromatography was performed on 40-63 µm silica gel manually or using a CombiFlash NextGen 300+ Autocolumn system. Electrospray ionization (ESI) measurements were performed at the Scottish Instrumentation and Resource Centre for Advanced Mass Spectrometry (SIRCAMS) based in the School of Chemistry at the University of Edinburgh. High resolution mass spectra were recorded on a Bruker Daltonics 12T SolariX Fourier Transform Ion Cyclotron Resonance Mass Spectrometer (FT-ICR-MS). Elemental analyses were carried out by Elemental Analysis Ltd., measured in duplicate.

2 Amide Synthesis

Amide synthesis

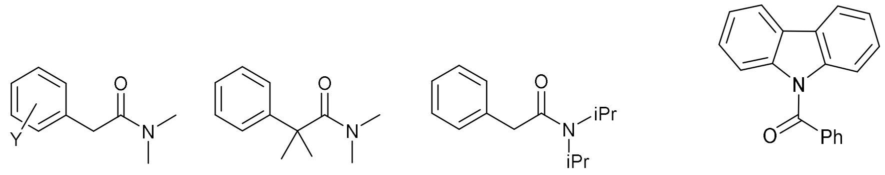

S1 **S2** **S3** **S4**

Amides **S1a-d,**[1] **S2**[1] and **S3**[1] and **S4**[2] were prepared according to previously reported procedures.

Synthesis of N-acylated substrates: General procedure 1

To an ampoule, triethylamine (1.5 eq) was added to the solution of respective amine (1 eq) in dry DCM. To this stirred solution was added dropwise the acyl chloride (1.2 eq) at 0°C and the mixture was left stirring at room temperature (for up to two days). Upon reaction completion (monitored by TLC), all volatiles were removed under vacuum. The reaction mixture was extracted with DCM (×3), and combined organic fractions were washed with brine, dried over $MgSO_4$, filtered, concentrated under vacuum, and purified by column chromatography to afford the desired product.

Synthesis of N-acylated substrates: General procedure 2

To an ampoule, sodium hydride in mineral oil (1.5 eq) was added to the solution of the amine (1 eq) in dry THF (20 mL) and stirred for 10 minutes at 0 °C on an ice bath. The clear solution obtained was transferred to a solution of acyl chloride (1.2 eq) in dry THF (10 mL) at 0 °C and allowed to stir at room temperature. On completion (monitored by TLC), all volatiles were removed under vacuum. The reaction mixture was extracted with DCM (×3), and combined organic fractions were washed with brine, dried over $MgSO_4$, filtered, concentrated under vacuum, and purified by column chromatography to afford the product.

_N,N'_-(1,4-phenylene)bis(2,2-dimethylpropanamide)

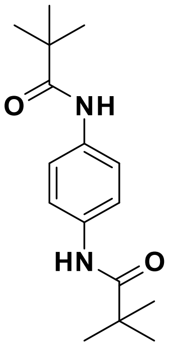

The title compound was prepared following general procedure 1 using *p*-phenylenediamine (1.08 g, 10 mmol, 1 eq), Et_3N (3.3 mL, 24 mmol, 2.4 eq), and DCM (40 mL). To a stirred solution was then added pivaloyl chloride (2.7 mL, 22 mmol, 2.2 eq). The crude reaction mixture obtained was purified by column chromatography on silica gel (petroleum ether/ ethyl acetate = 9:1) to afford the product as a white crystalline solid (2.6 g, 94 %). [Acc. Mass] Calculated for $[M+H]^+$: 277.1924, found $[M+H]^+$ 277.1911.

^{1}H NMR (500 MHz, $CDCl_3$, 298 K): δ 7.49 (s, 4H), 7.29 (br s, 2H), 1.31 (s, 18H). **^{13}C{^{1}H} NMR** (126 MHz, $CDCl_3$, 298 K): δ 176.6, 134.4, 120.7, 39.7, 27.7.

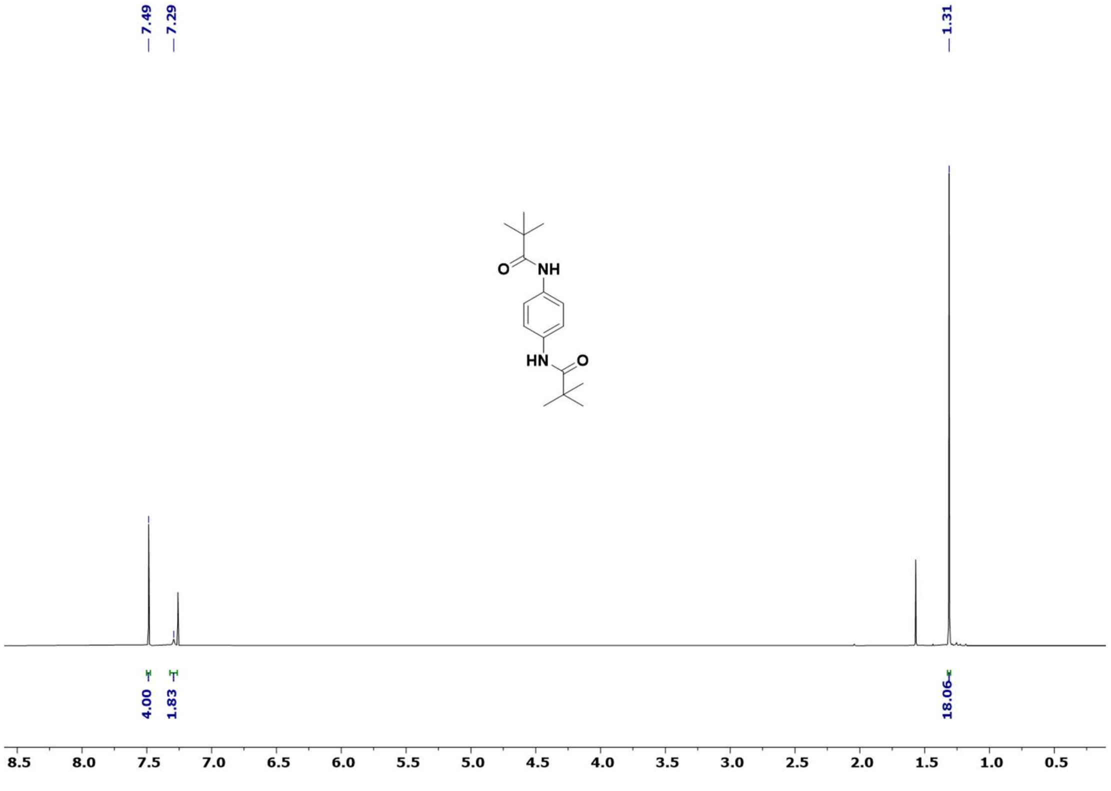

Figure S1. ^{1}H NMR spectrum in $CDCl_3$.

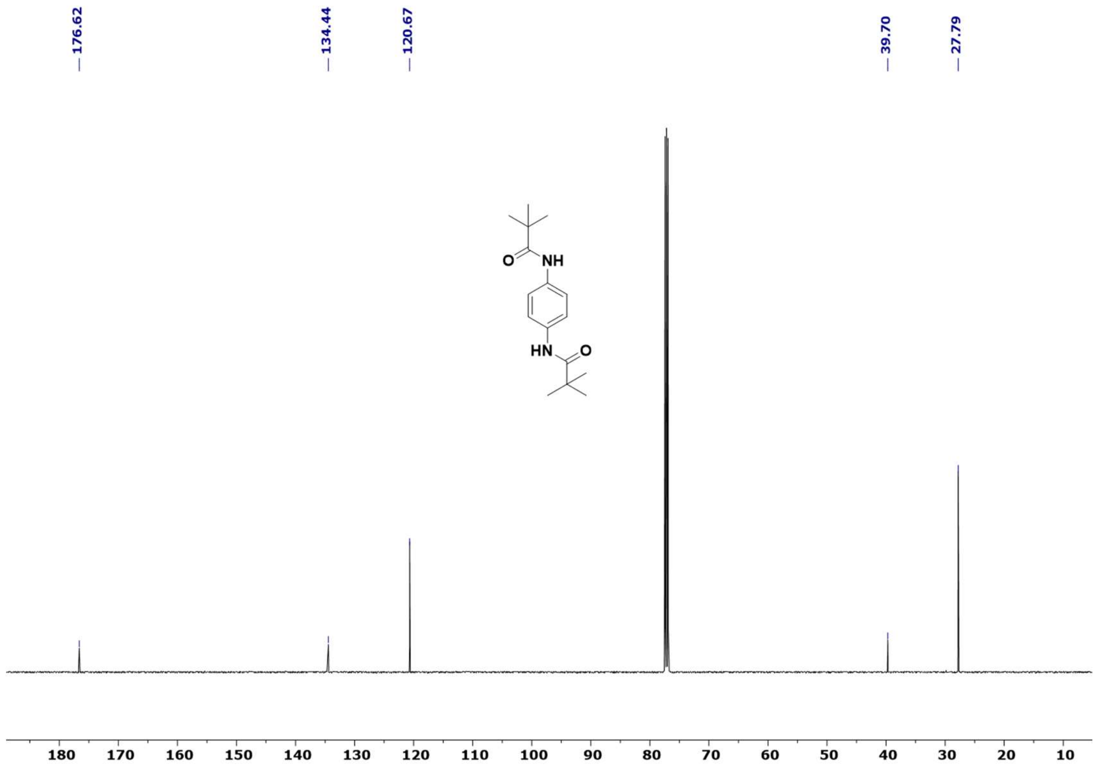

Figure S2. $^{13}C\{^1H\}$ NMR spectrum in $CDCl_3$.

***N,N'*-(naphthalene-1,5-diyl)bis(2,2-dimethylpropanamide)**

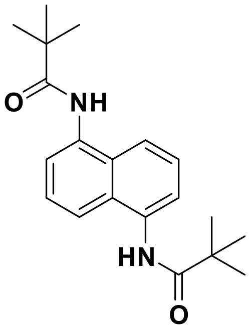

The title compound was prepared following general procedure 1 using 1,5-diamminonapthalene (1.58 g, 10 mmol, 1 eq), Et_3N (3.3 mL, 24 mmol, 2.4 eq), and DCM (40 mL). To a stirred solution was then added pivaloyl chloride (2.7 mL, 22 mmol, 2.2 eq). Upon completion, monitored by TLC, the reaction mixture was evaporated, and washed several times with water, to remove triethylammonium salt to get the pure product as a white solid (2.71 g, 83 %). [Acc. Mass] Calculated for $[M+H]^+$: 327.20887, found $[M+H]^+$ 327.20670.

^{1}H NMR (500 MHz, $CDCl_3$, 298 K): δ 8.04 (d, *J*=7.4 Hz, 2H), 7.80 (br s, 2H), 7.63 (d, *J*=8.5 Hz, 2H), 7.53 (t, *J*=8.0 Hz, 2H), 1.45 (s, 18 H). **^{13}C{^{1}H} NMR** (126 MHz, $CDCl_3$, 298 K): δ 177.1, 133.4, 128.0, 126.4, 120.9, 117.5, 40.1, 28.0.

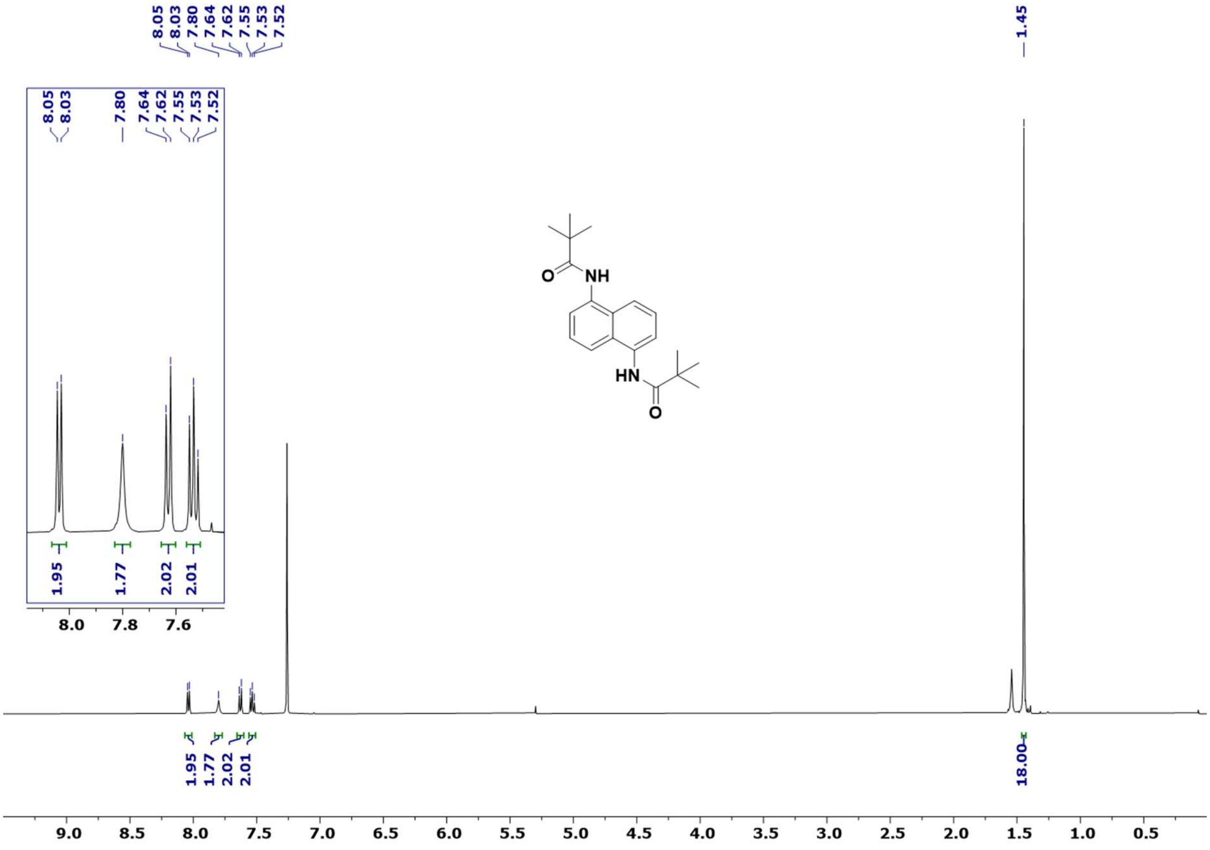

Figure S3. ^{1}H NMR spectrum in $CDCl_3$.

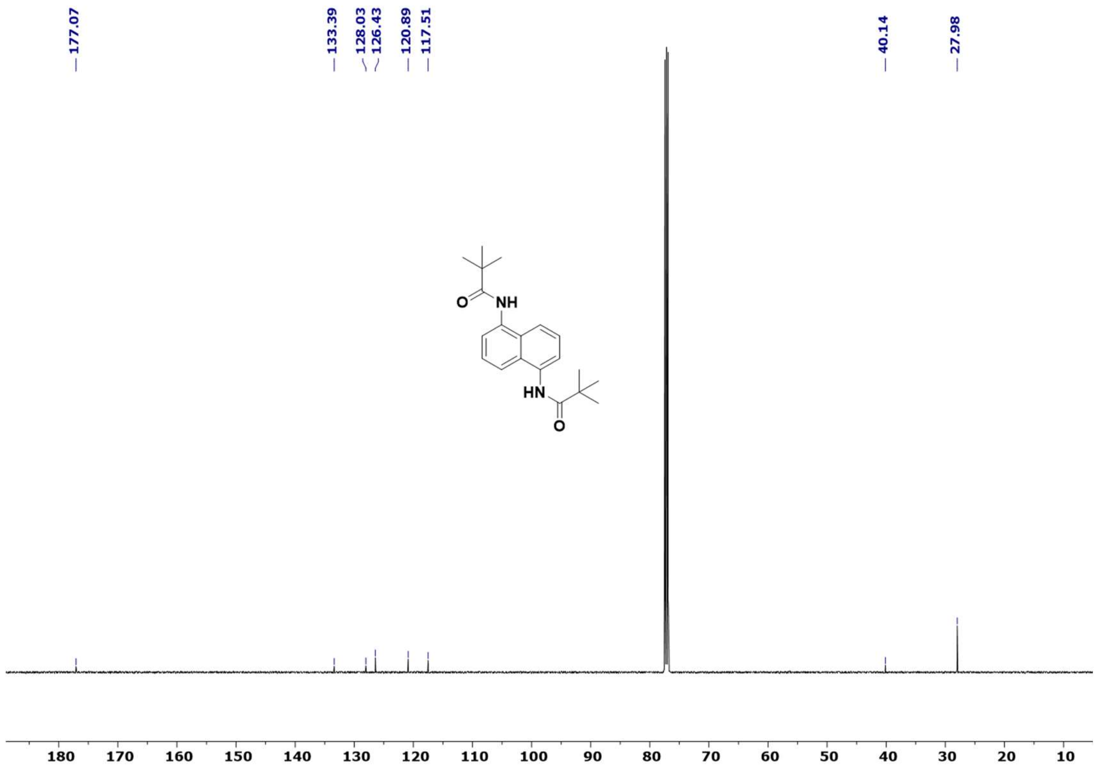

Figure S4. $^{13}C\{^1H\}$ NMR spectrum in $CDCl_3$.

1-(9H-carbazol-9-yl)octan-1-one (1c)

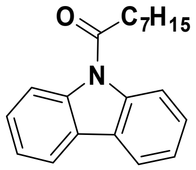

The title compound was prepared following general procedure 2 using carbazole (836 mg, 5 mmol, 1 eq), 90 % dry suspension of sodium hydride in mineral oil (180 mg, 7.5 mmol, 1.5 eq), and dry THF (20 mL). The stirred solution was then added to the solution of octanoyl chloride (1.0 mL, 6 mmol, 1.2 eq) in dry THF (10 mL). The crude product obtained was purified by column chromatography on silica gel (petroleum ether) to afford the product as a white crystalline solid (661 mg, 45 %).

^{1}H NMR (500 MHz, $CDCl_3$, 298 K): δ 8.23 (d, *J*=8.4 Hz, 2H), 8.02-7.98 (m, 2H), 7.51-7.46 (m, 2H), 7.39 (td, *J*=7.5, 0.9 Hz, 2H), 3.17-3.12 (m, 2H), 1.97-1.90 (m, 2H), 1.54-1.47 (m, 2H) 1.44-1.37 (m, 2H), 1.36-1.29 (m, 4H), 0.93-0.88 (m, 3H). **^{13}C{^{1}H} NMR** (126 MHz, $CDCl_3$, 298 K): δ 173.6, 138.7, 127.4, 126.5, 123.6, 119.9, 116.6, 39.4, 31.9, 29.4, 29.3, 24.9, 22.8, 14.2.

Analytical data are in accordance with the literature report.[3]

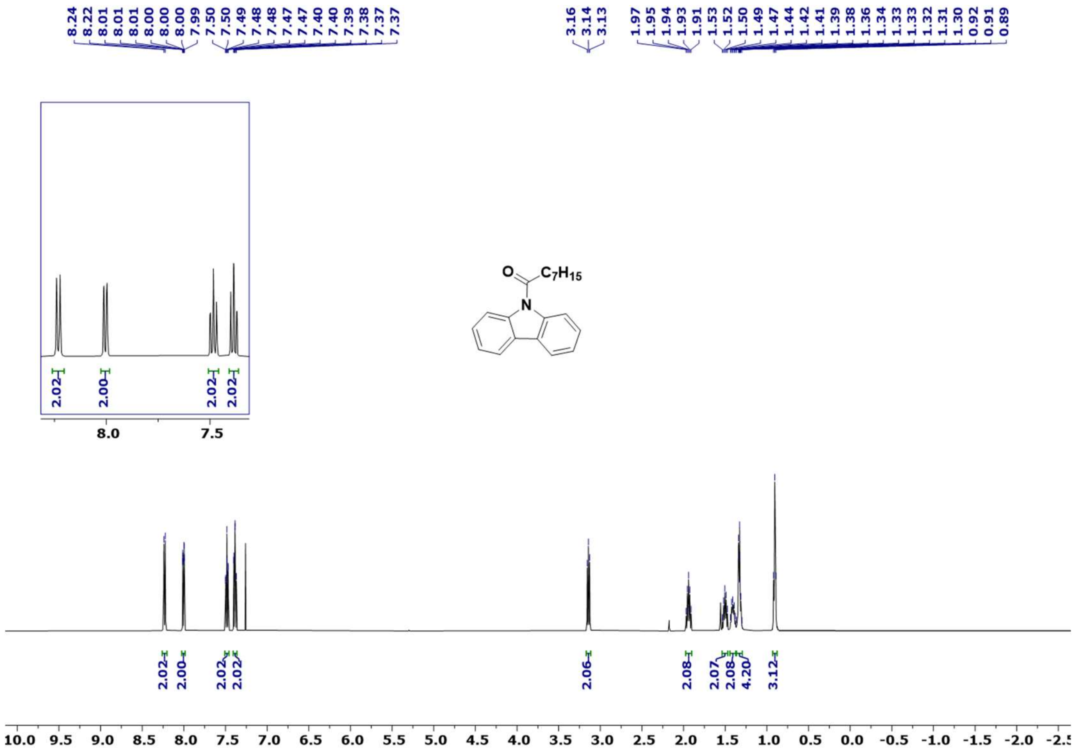

Figure S5. ^{1}H NMR spectrum in $CDCl_3$.

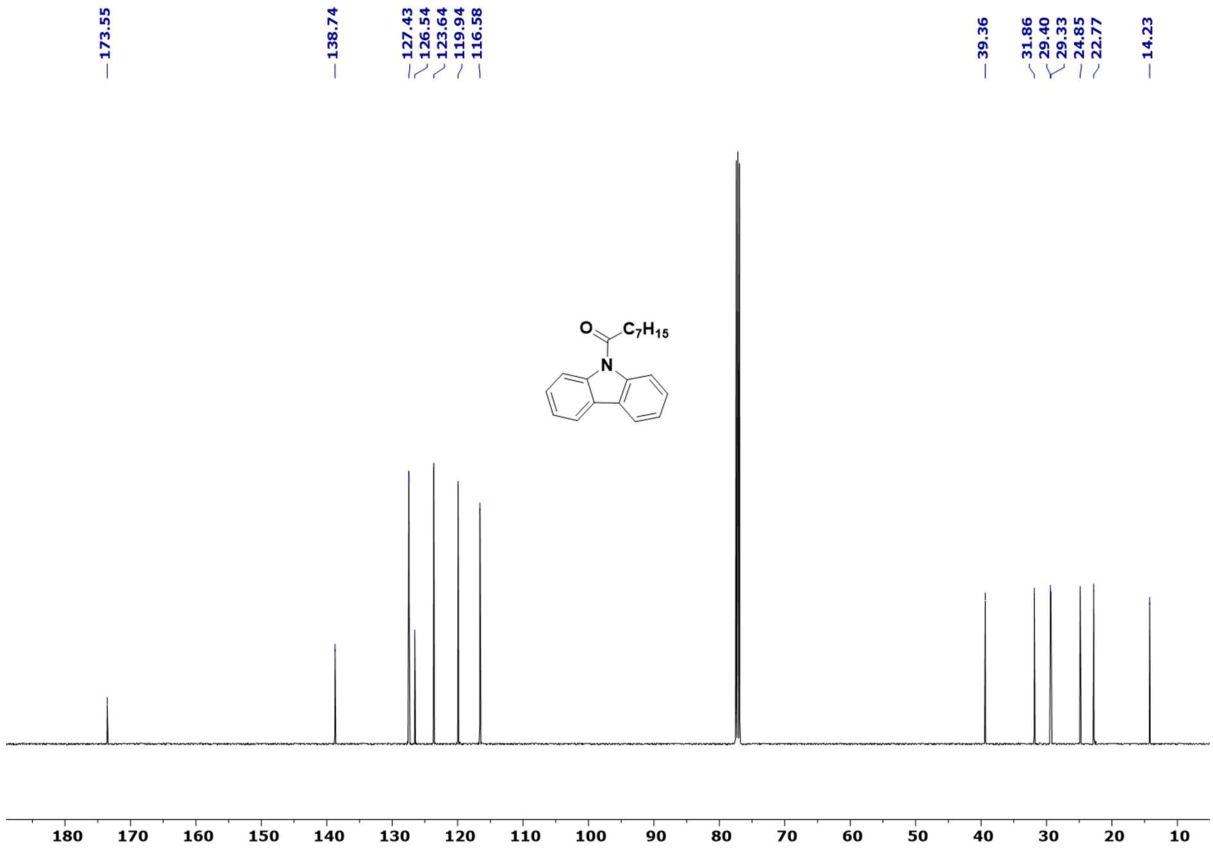

Figure S6. $^{13}C\{^1H\}$ NMR spectrum in $CDCl_3$.

3 Borylation – reduction of 2-phenylacetamides

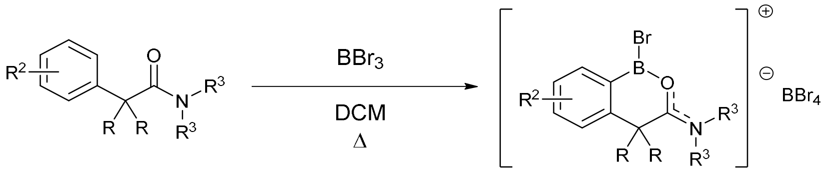

Ortho borylation of the phenyl acetylamides to generate **1[BBr₄]**, **4** and **8** was conducted according to our previously reported procedure.[1]

Reduction of 1[BBr₄]: *in-situ* monitoring

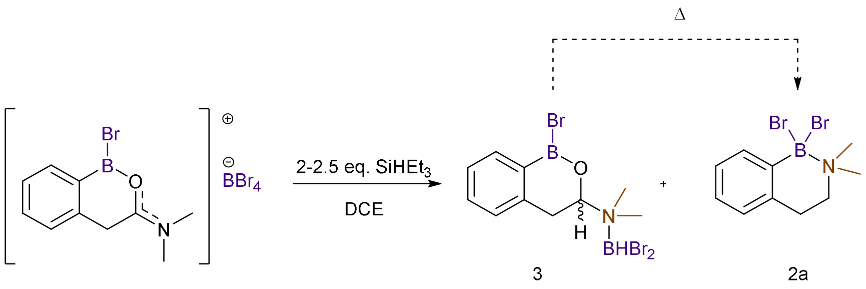

To an NMR tube fitted with a J. Young valve was added **1[BBr₄]** (0.1 mmol, 0.059 g) in a glove box. To the tube was added anhydrous DCE (1 mL), the tube was sealed and shaken to form a brown solution. To this tube was added $SiHEt_3$ (0.25 mmol, 40 μL) to form a colourless solution. The reaction mixture was monitored at intervals at room temperature and at 60 °C.

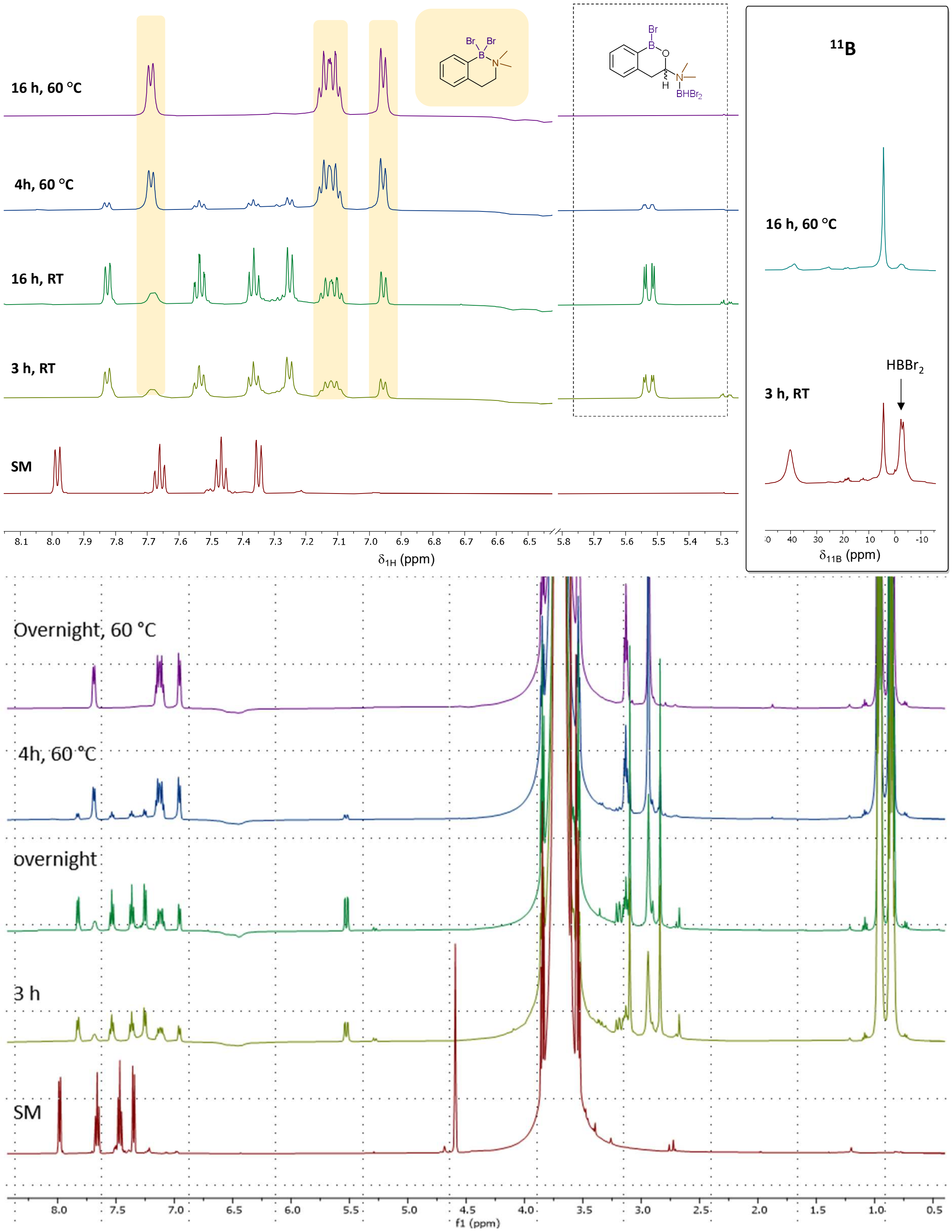

Figure S7. Top, stacked *in-situ* ^{1}H and ^{11}B (inset) NMR spectra showing conversion from **1[BBr4]** to **2a/3** and then completely to **2a** in dichloroethane. Bottom, full spectrum of the same monitoring reactions – note growth and disappearance of resonances at 3.1 ppm for the diastereotopic CH_2 protons in **3**.

Altering the silane source (which had negligible effect on selectivity).

Table S1: Comparison between various hydrosilanes. Number of equivalents (n) and time are reported as those where the best results were obtained. All reactions conducted at room temperature. Ratio of products determined by *in-situ* ^{1}H NMR spectroscopy. **SM=starting material**. **HA=hemiaminal (3)**. **AB=amine-borane (2a)**.

Silane	n	Time	Conversion (%)[a] 1[BBr_4]:**3**:**2a** SM:**HA**:**AB**
$SiHEt_3$	2	3 h	0:**66**:**34**
	1.6	1 h	30:**52**:**19**
$SiHPh_3$	1	10 min	69:**24**:**7**
	2[b]	6 h	0:**60**:**40**
SiH_2Ph_2	1	2 h	0:**66**:**34**
$SiHMe_2Ph$	2	3 h	0:**64**:**36**

[a] Conversion ratios stated at point selectivity could be conclusively determined by 1H NMR spectroscopy. [b] 2nd equivalent added after stirring with 1 equivalent for 16 h.

Isolation of amine boranes

Compound 2a

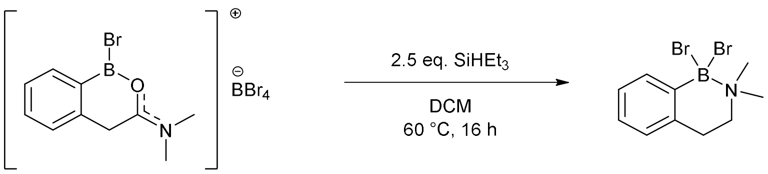

Compound **1[BBr_4]** (0.059 g, 0.1 mmol) was added to an NMR tube fitted with a J. Young valve. The solid was dissolved in DCM (0.7 mL) and $SiHEt_3$ (2.5 eq. 40 μL) was added, the tube was sealed and heated to 60 °C for 16 h during which a white precipitate formed. The precipitate was removed by filtration of the reaction mixture through a 0.22 μm filter syringe and the product extracted from the remaining solids with DCM (1 mL). The combined filtrates were concentrated under vacuum and the product dried to give **2a** (0.026 g, 83%) as a white solid.

^{1}H NMR (400 MHz, CD_2Cl_2) δ 7.77 (d, J = 7.1 Hz, 1H), 7.27 – 7.14 (m, 2H), 7.07 – 6.99 (m, 1H), 3.62 (t, J = 6.5 Hz, 2H), 3.21 (t, J = 6.5 Hz, 2H), 3.03 (s, 6H). **^{11}B NMR** (128 MHz, CD_2Cl_2) δ 4.5. **^{13}C{^{1}H} NMR** (126 MHz, CD_2Cl_2) δ 135.3, 133.2, 128.2, 127.4, 126.8, 58.1, 49.3, 27.5. **[Acc. Mass]** calcd for [M]=$C_{10}H_{14}BBr_2N$: 316.9581, found 316.9592.

Compound 2a: 2-step-1-pot synthesis

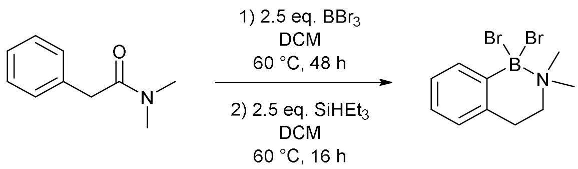

Compound **S1** (0.024 g, 0.15 mmol) was added to an NMR tube fitted with a J. Young valve. The solid was dissolved in DCM (0.4 mL) and BBr_3 (1M in DCM, 2.5 eq., 0.38 mL) was added. The tube was sealed, and the reaction mixture heated at 60 °C for 48 h. The solvents/volatiles were removed under vacuum and the product dried. The borylated species was dissolved in DCM (1.1 mL) and $SiHEt_3$ (2.5 eq., 60 μL) was added, the tube was sealed and heated to 60 °C for 16 h during which a white precipitate formed. The precipitate was removed by filtration of the reaction mixture through a 0.22 μm filter syringe and the product extracted from the remaining solids with DCM (1 mL). The combined filtrates were concentrated under vacuum and the product dried to give **2a** (0.039 g, 81%) as a white solid.

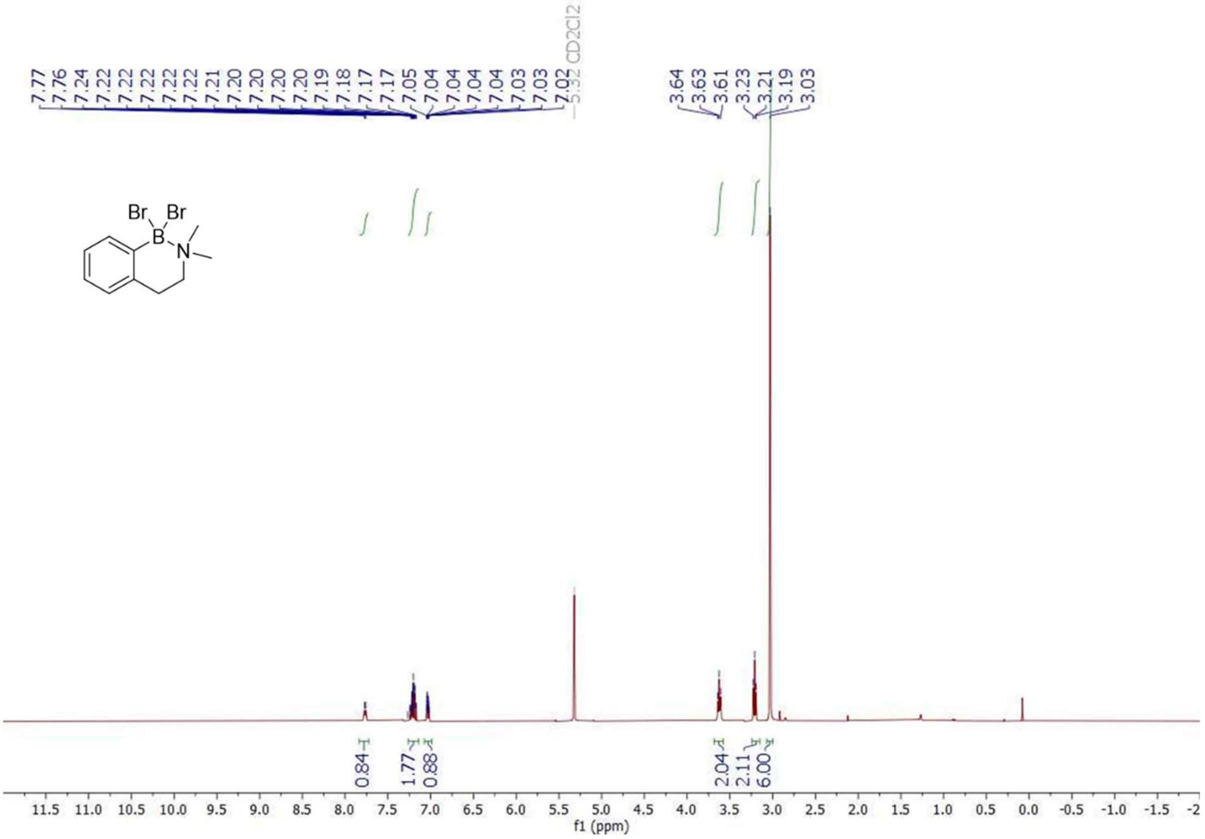

Figure S8. ^{1}H NMR spectrum of compound **2a** in CD_2Cl_2.

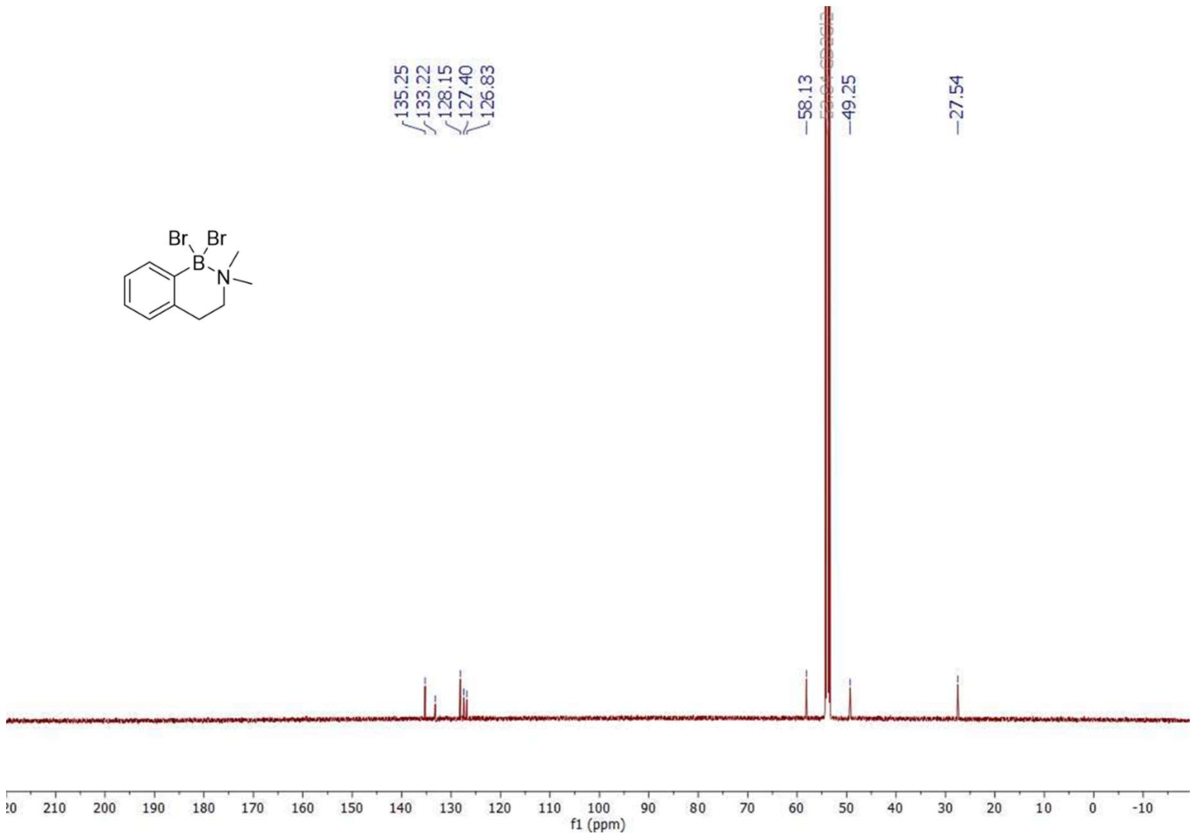

Figure S9. $^{13}C\{^{1}H\}$ NMR spectrum of compound **2a** in CD_2Cl_2.

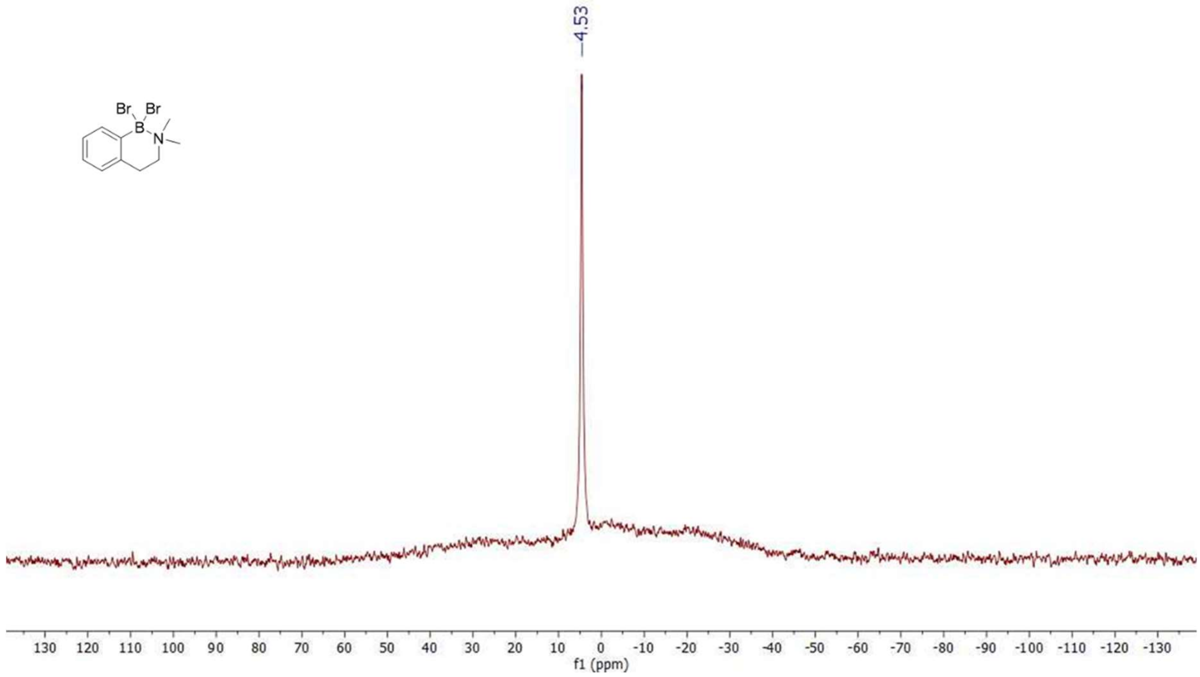

Figure S10. ^{11}B NMR spectrum of compound **2a** in CD_2Cl_2.

Compound 2b

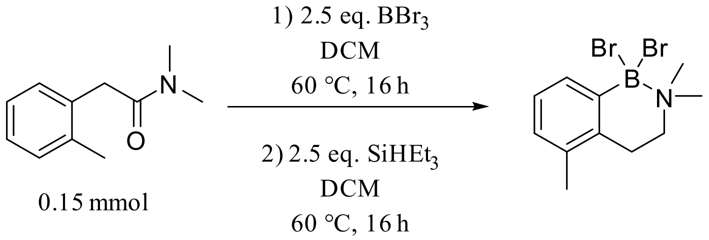

To an NMR tube fitted with a J-Youngs valve was added N,N-dimethyl-2-phenylacetamide (0.024 g, 0.15 mmol) which was dissolved in DCM (0.4 mL). BBr_3 (1M in DCM, 0.38 mL, 0.375 mmol) was added and the tube was sealed and heated at 60 °C for 16 hours. The solvents were removed under vacuum and the product dried. The solid was dissolved in DCM (1.1 mL) and $SiHEt_3$ (60 µL, 0.375 mmol) was added. The tube was sealed and heated at 60 °C for 16 hours. White precipitate formed, which was removed by filtration through 0.22 µm filter syringe. The tube was extracted by DCM (1 mL) and all solutions were combined. The solution was dried under vacuum to give the desired product (0.046 g, 92%) as a white solid without any further purification.

^{1}H NMR (500 MHz, $CDCl_3$) δ 7.75 (d, J = 7.7 Hz, 1H), 7.19 (t, J = 7.6 Hz, 1H), 7.06 (d, J = 7.4 Hz, 1H), 3.80 – 3.57 (t, J = 6.6 Hz, 2H), 3.06 (s, 6H), 3.04 (t, J = 6.5 Hz, 2H), 2.20 (s, 3H). **^{11}B NMR** (160 MHz, $CDCl_3$) δ 4.7. **^{13}C{^{1}H} NMR** (126 MHz, $CDCl_3$) δ 134.1, 133.2, 131.0, 129.6, 126.9, 57.8, 49.0, 25.5, 19.7. **[Acc. Mass]** calcd for [M] = $C_{11}H_{16}BBr_2N$: 330.97371, found 330.97484.

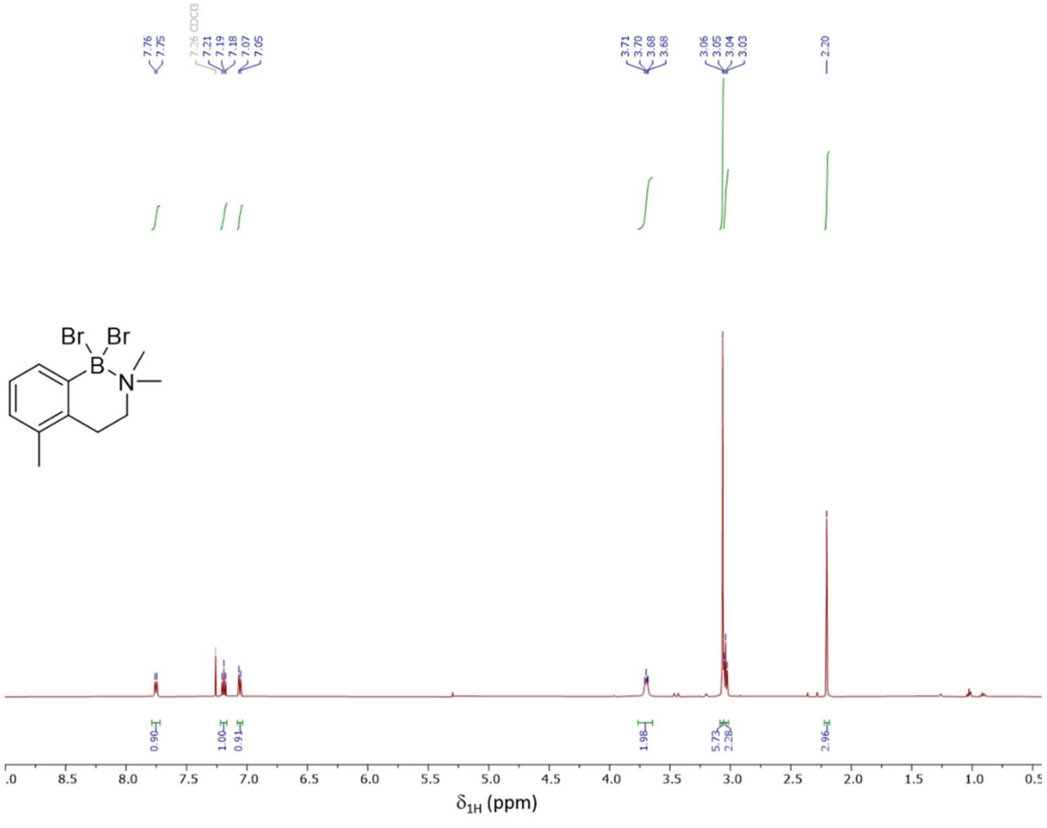

Figure S11. ^{1}H NMR spectrum of compound **2b** in $CDCl_3$.

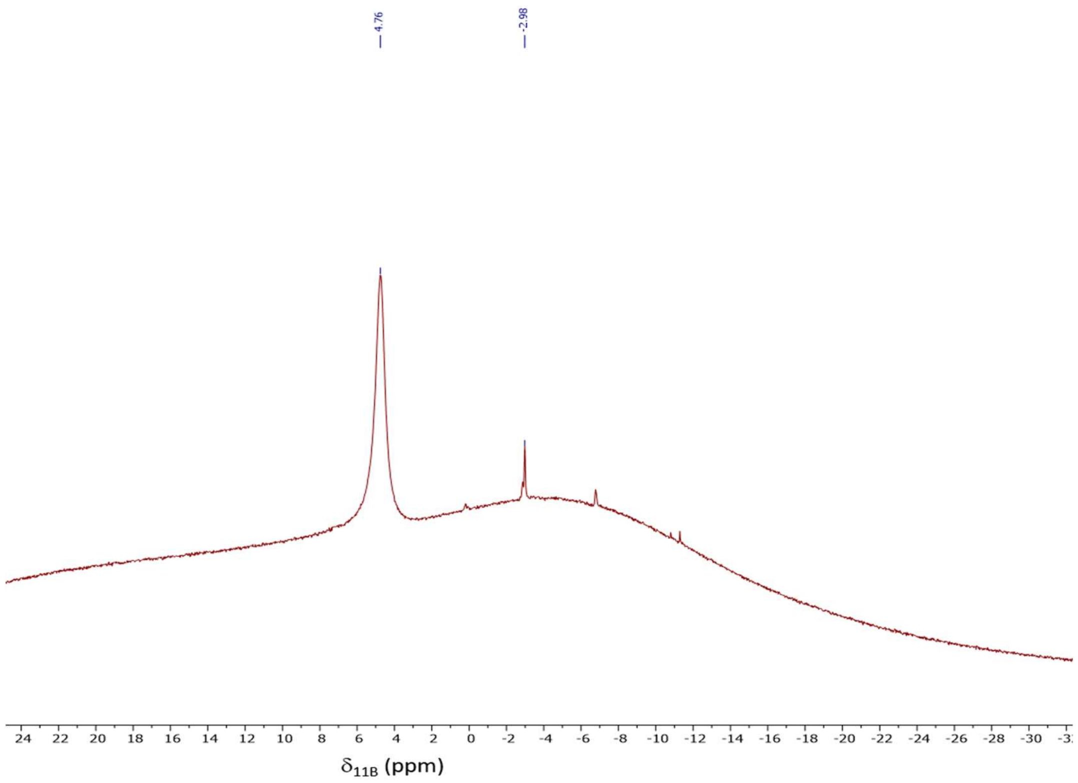

Figure S12. ^{11}B NMR spectrum of compound **2b** in $CDCl_3$.

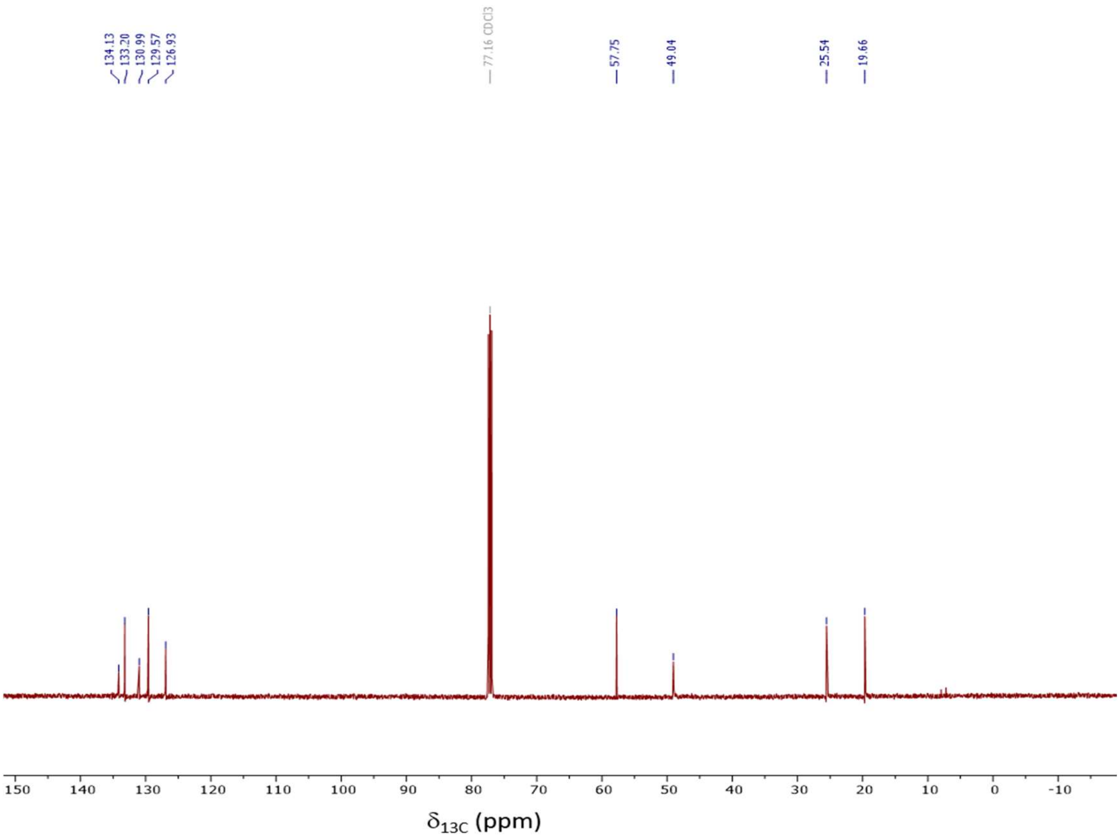

Figure S13. $^{13}C\{^1H\}$ NMR spectrum of compound **2b** in $CDCl_3$.

Compound 2c

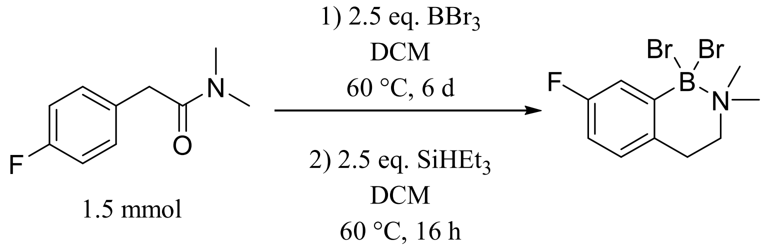

To an ampule fitted with a J-Youngs valve was added 4-fluorophenylacetyl chloride (0.27 g, 1.5 mmol) which was dissolved in DCM (7.5 mL). BBr_3 (0.36 mL, 3.75 mmol) was added and the ampule was sealed and heated at 60 °C for 6 days (note borylation times can be reduced using higher boiling solvents and higher temperatures). The solvents were removed under vacuum and the product dried. The solid was dissolved in DCM (11 mL) and $SiHEt_3$ (0.60 mL, 3.75 mmol) was added. The ampule was sealed and heated at 60 °C for 16 hours. White precipitate formed which was removed by filtration through a 0.22 µm filter syringe. The ampule was extracted by DCM (3 x 2 mL) and all solutions were combined. The solution was dried under vacuum and the product was washed with pentane (5 mL stirred for 30 minutes). The pentane was decantedand the remaining solid was dried to give a pale-yellow product (0.427 g, 85%).

^{1}H NMR (500 MHz, $CDCl_3$) δ 7.52 (dd, J = 10.0, 2.8 Hz, 1H), 7.00 – 6.95 (m, 1H), 6.87 (td, J = 8.4, 2.8 Hz, 1H), 3.64 (t, J = 6.5 Hz, 2H), 3.19 (t, J = 6.5, 2H), 3.05 (s, 6H). **^{11}B NMR** (160 MHz, $CDCl_3$) δ 3.9. **^{13}C{^{1}H} NMR** (126 MHz, $CDCl_3$) δ 162.0 (d, J = 245.1 Hz), 128.9 (d, J = 7.4 Hz), 127.7 (d, J = 2.9 Hz), 120.9 (d, J = 20.4 Hz), 115.5 (d, J = 22.2 Hz), 57.9, 49.1, 26.7. **[Acc. Mass]** calcd for [M]=$C_{10}H_{13}BBr_2FN$:, found. 334.94863, found 334.94784

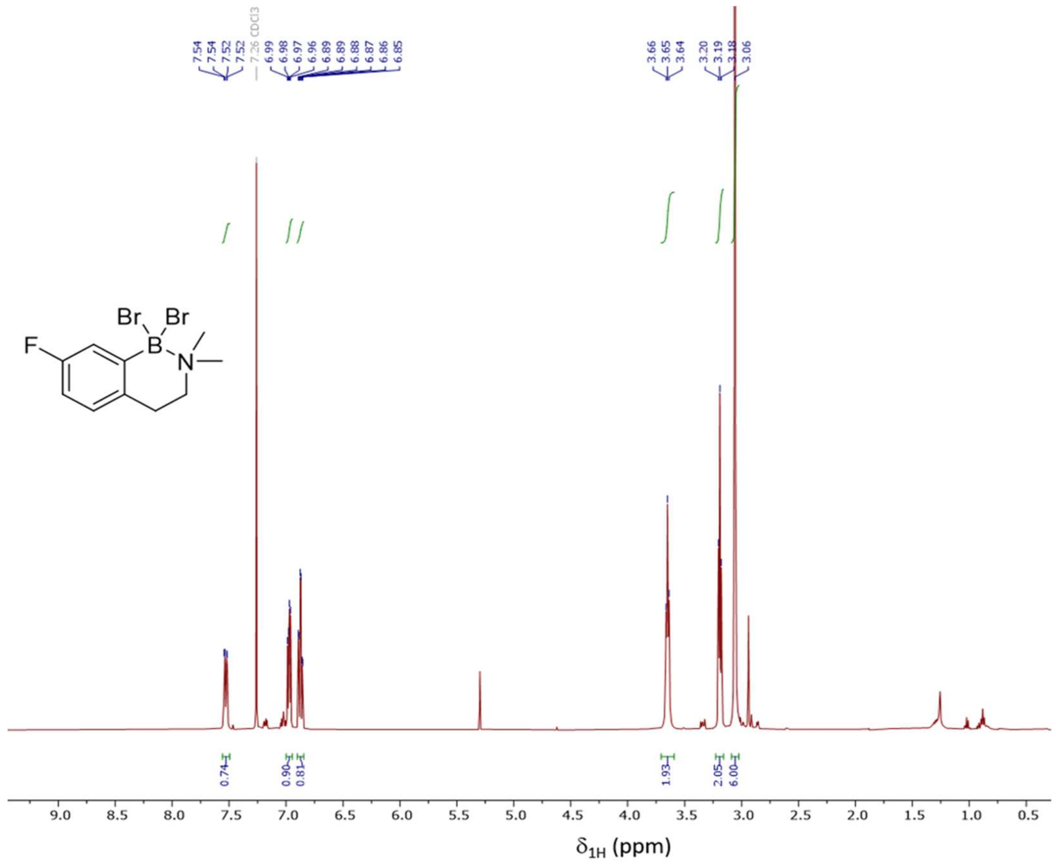

Figure S14. ^{1}H NMR spectrum of compound **2c** in $CDCl_3$.

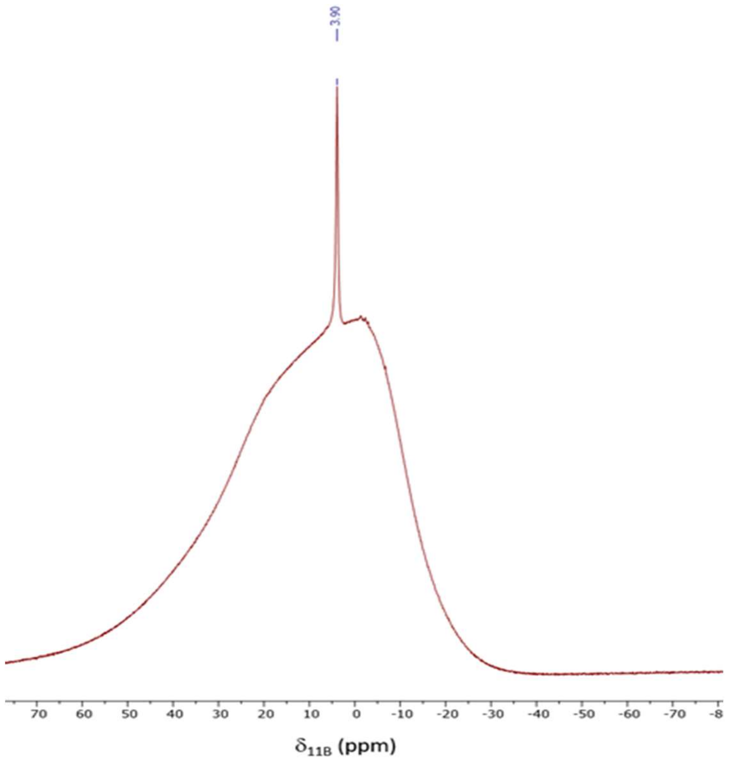

Figure S15. ^{11}B NMR spectrum of compound **2c** in $CDCl_3$.

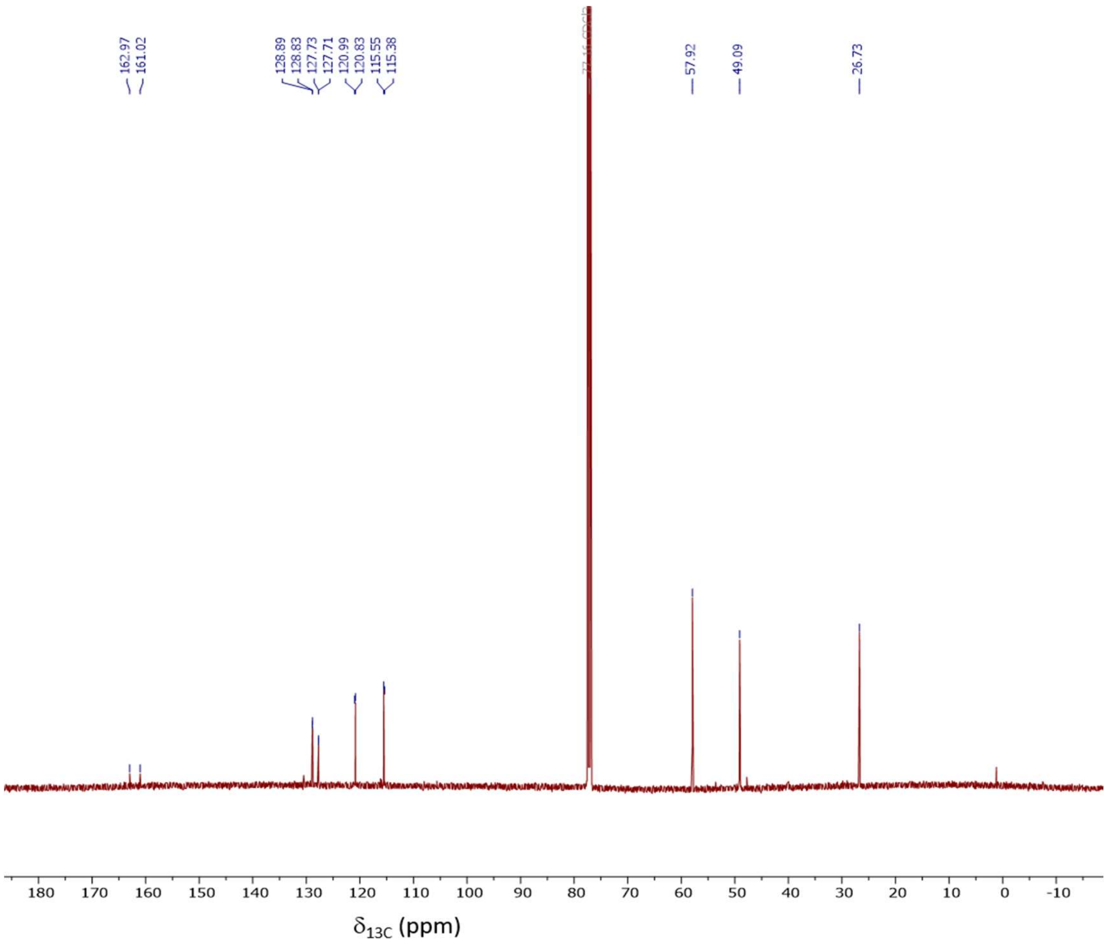

Figure S16. $^{13}C\{^{1}H\}$ NMR spectrum of compound **2c** in $CDCl_3$.

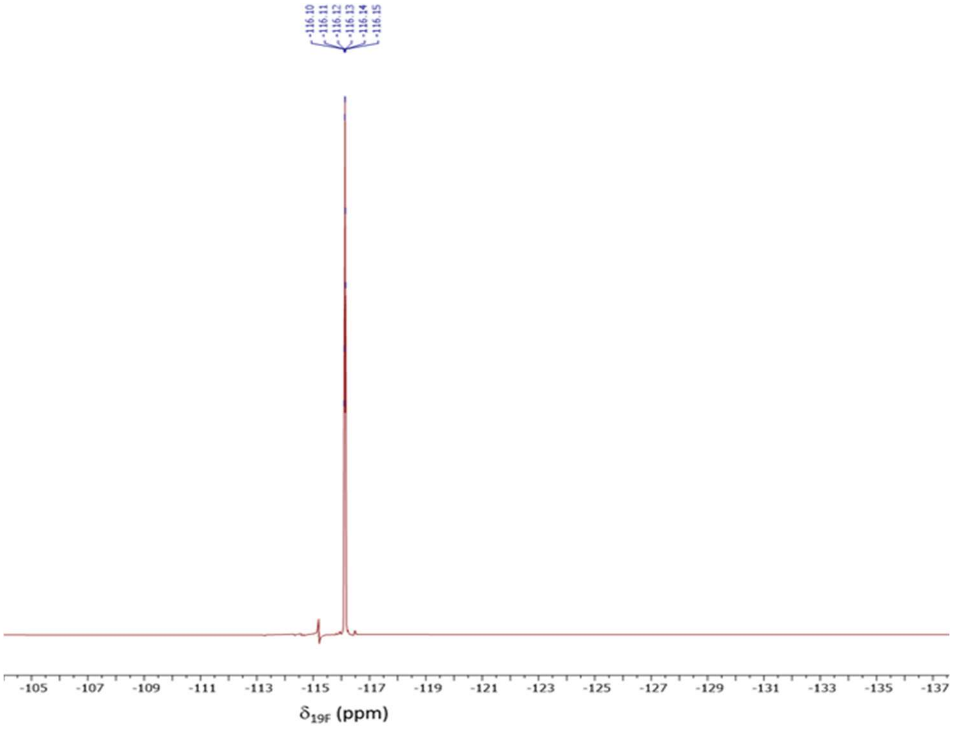

Figure S17. $^{19}F\{^{1}H\}$ NMR spectrum of compound **2c** in $CDCl_3$.

Compound 2d

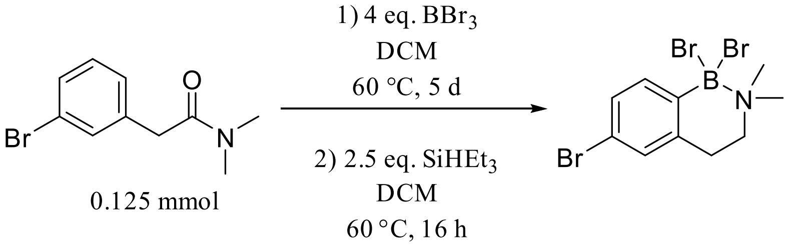

To an NMR tube fitted with a J-Youngs valve was added 2-(3-bromophenyl)-N,N-dimethylacetamide (0.030 g, 0.125 mmol) which was dissolved in DCM (0.35 mL). BBr_3 (1M in DCM, 0.5 mL, 0.5 mmol) was added and the tube was sealed and heated at 60 °C for 5 days. The solvents were removed under vacuum and the product dried. The solid was dissolved in DCM (1.1 mL) and $SiHEt_3$ (50 µL, 0.31 mmol) was added. The tube was sealed and heated at 60 °C for 16 hours. White precipitate formed which was removed by filtration through a 0.22 µm filter syringe. The tube was extracted by DCM (1 mL) and all solutions were combined. The solution was dried under vacuum and the solid was washed by pentane (5 mL and stirred for 30 minutes). The pentane solution was decanted and the remaining solid was dried to give a pale-yellow product (0.032 g, 64%).

^{1}H NMR (400 MHz, $CDCl_3$) δ 7.73 (d, J = 8.3 Hz, 1H), 7.39 – 7.35 (m, 1H), 7.17 (dd, J = 2.0, 1.0 Hz, 1H), 3.64 (t, J = 6.5 Hz, 2H), 3.19 (t, J = 6.5 Hz, 2H), 3.05 (s, 6H). **^{11}B NMR** (128 MHz, $CDCl_3$) δ 4.2. **^{13}C{^{1}H} NMR** (126 MHz, $CDCl_3$) δ 136.9, 134.2, 130.0, 129.6, 122.1, 57.4, 49.0, 26.9. **[Acc. Mass]** calcd for [M]=$C_{10}H_{13}BBr_3N$: 394.86857, found 394.86937.

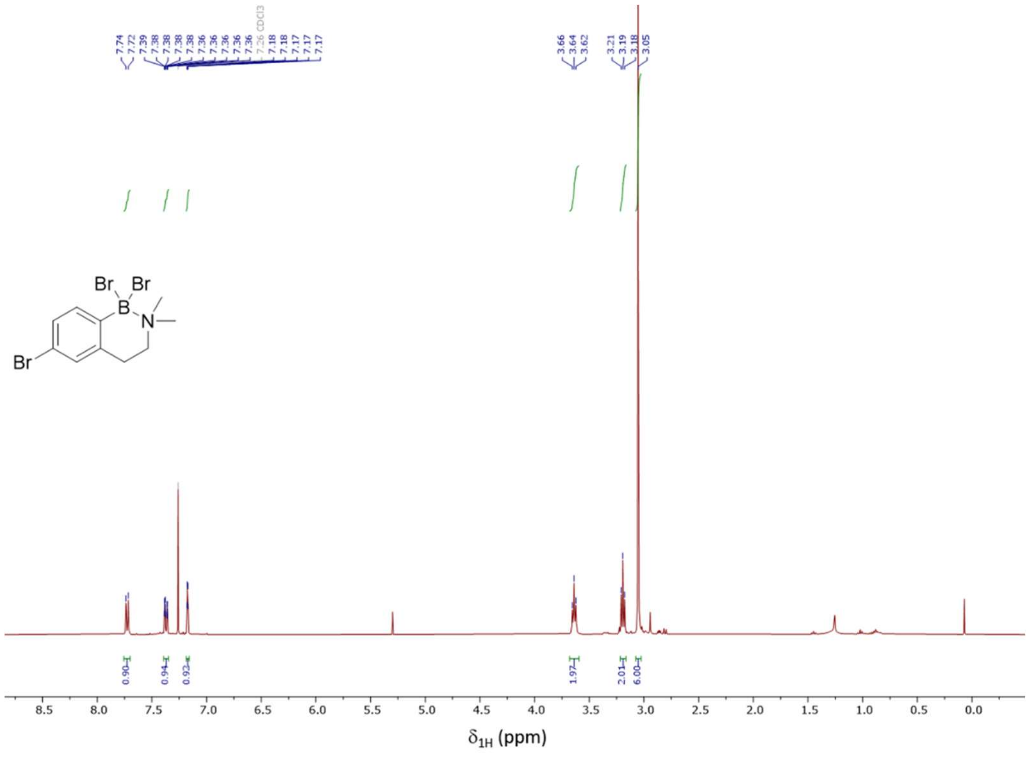

Figure S18. ^{1}H NMR spectrum of compound **2d** in $CDCl_3$.

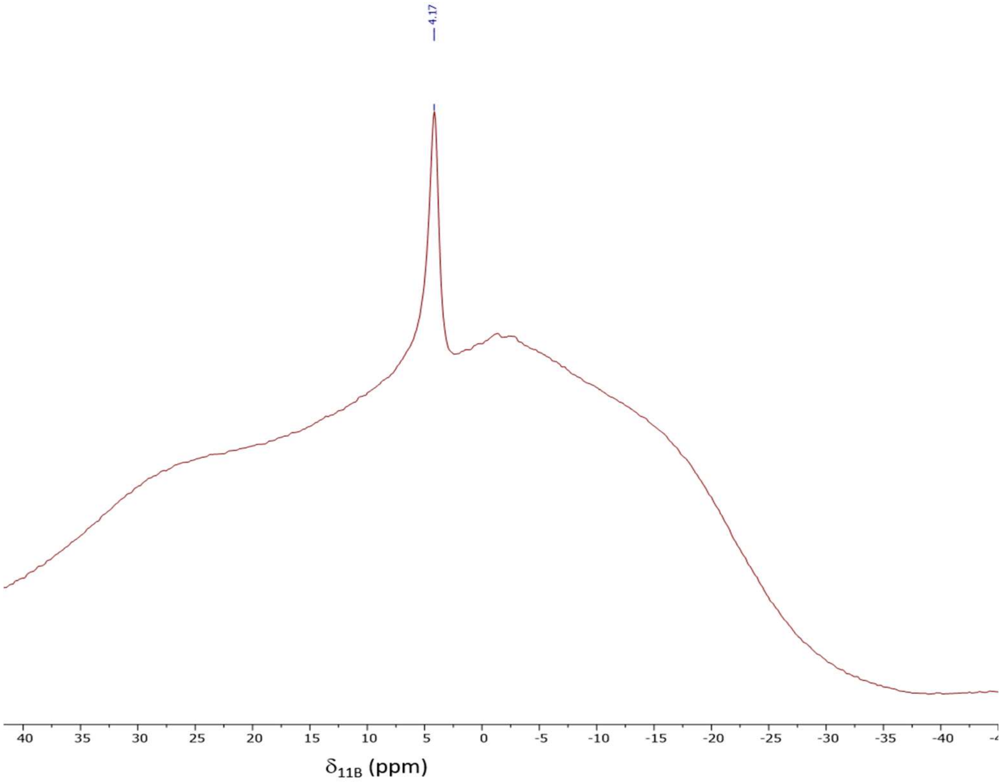

Figure S19. ^{11}B NMR spectrum of compound **2d** in $CDCl_3$.

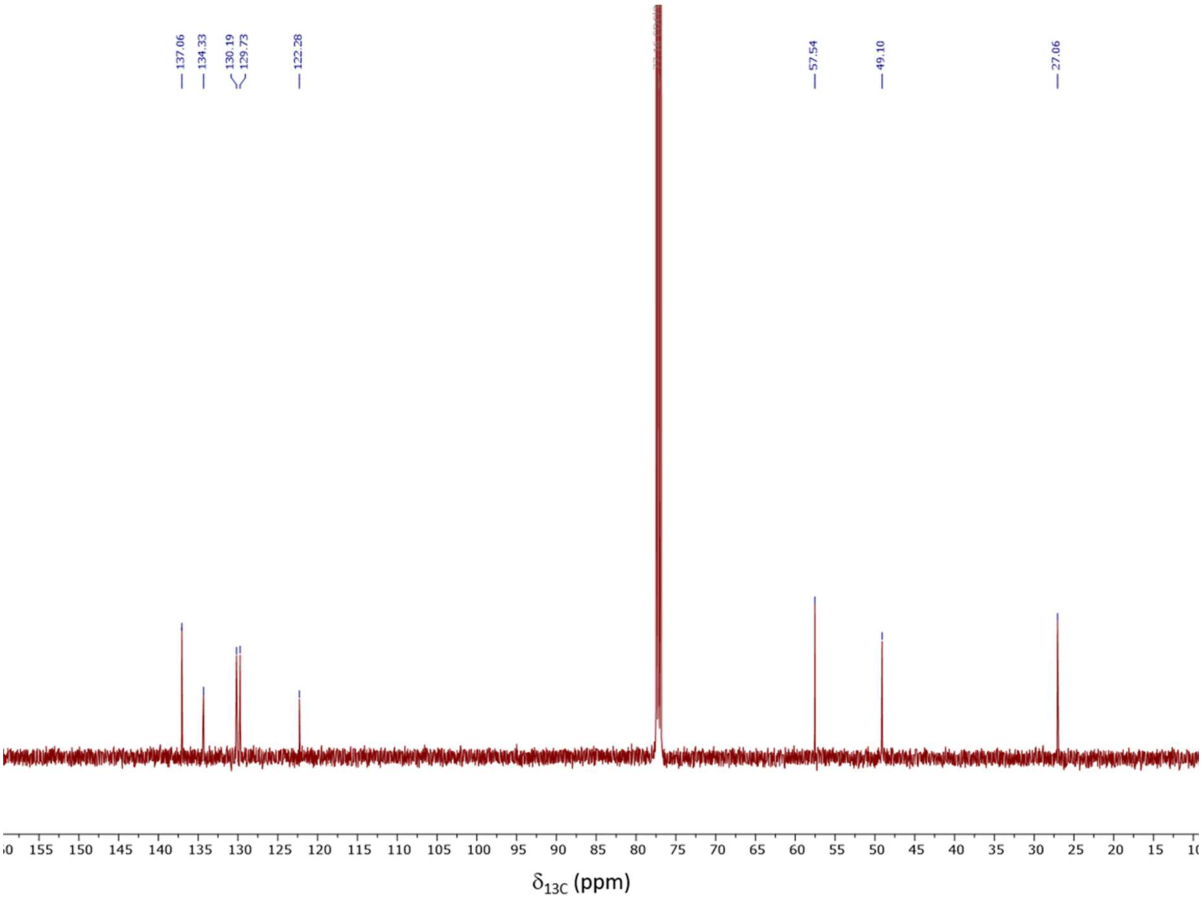

Figure S20. $^{13}C\{^{1}H\}$ NMR spectrum of compound **2d** in $CDCl_3$.

Compound 5

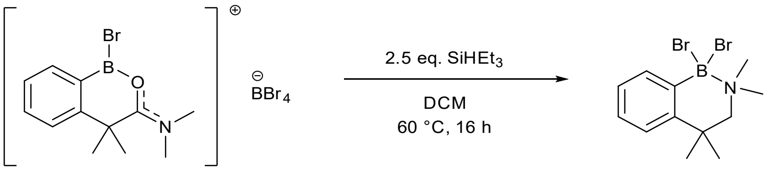

Compound **4** (0.090 g, 0.15 mmol) was added to an NMR tube fitted with a J. Young valve. To the solid was added DCM (1.7 mL) to form a suspension followed by $SiHEt_3$ (2.5 eq., 60 μL) which resulted in a clear solution. The tube was sealed and heated to 60 °C for 16 h during which a white precipitate formed. The precipitate was removed by filtration of the reaction mixture through a 0.22 μm filter syringe and the product extracted from the remaining solids with DCM (1 mL). The combined filtrates were concentrated under vacuum and the product dried to give **5** (0.0463 g, 89%) as a white solid. *(Crystals suitable for X-ray diffraction studies were grown by slow evaporation of a DCM/pentane solution of **5**)*.

^{1}H NMR (500 MHz, CD_2Cl_2) δ 7.81 (d, J = 6.8 Hz, 1H), 7.32 – 7.17 (m, 3H), 3.52 (s, 2H), 3.04 (s, 6H), 1.45 (s, 6H). **^{11}B NMR** (128 MHz, CD_2Cl_2) δ 5.0. **^{13}C{^{1}H} NMR** (126 MHz, CD_2Cl_2) δ 143.1, 134.6, 128.9, 126.6, 125.42, 70.8, 50.6, 36.2, 33.4. **[Acc. Mass].** calcd for [M]=$C_{10}H_{14}BBr_2N$: 344.9894, found 344.9894. *Product is bench stable in the solid-state but decomposes slowly when left in a wet CD_2Cl_2 solution for >24 h*

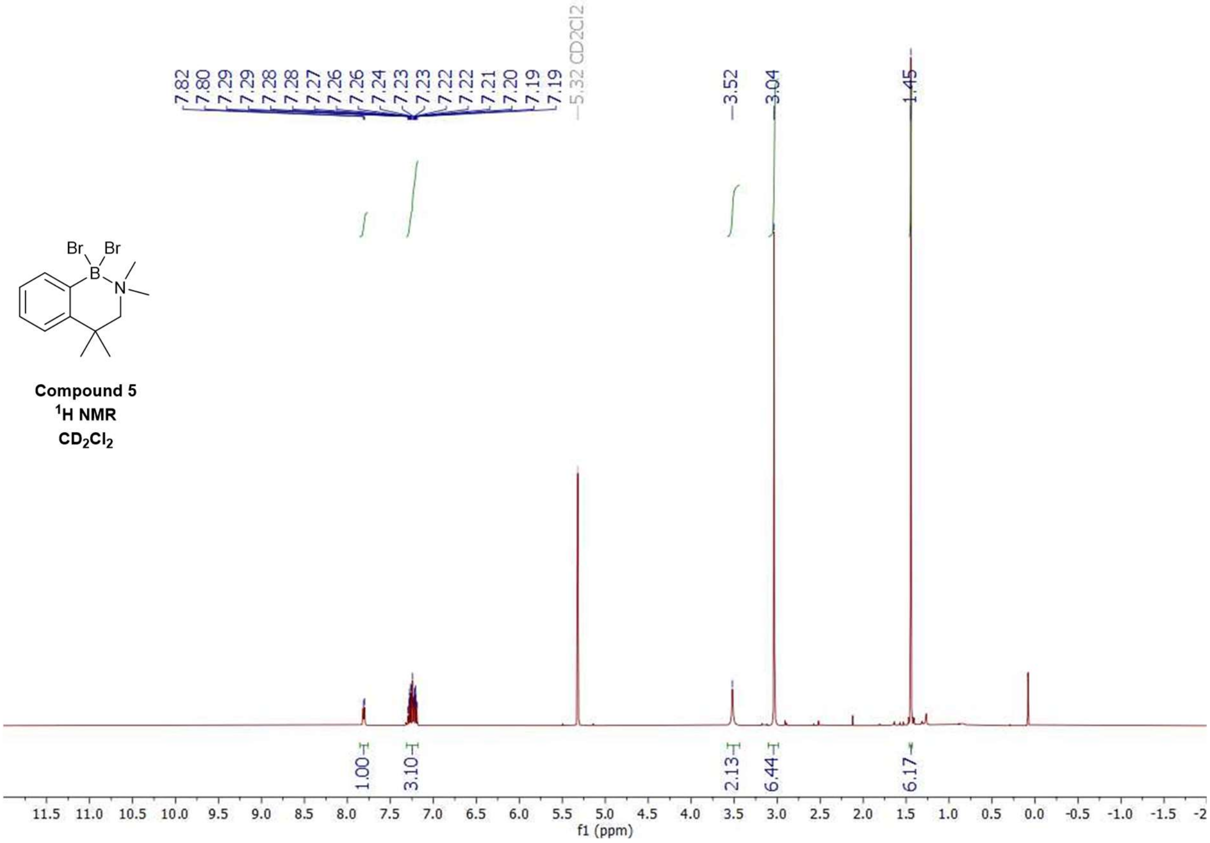

Figure S21. ^{1}H NMR spectrum of compound **5** in CD_2Cl_2.

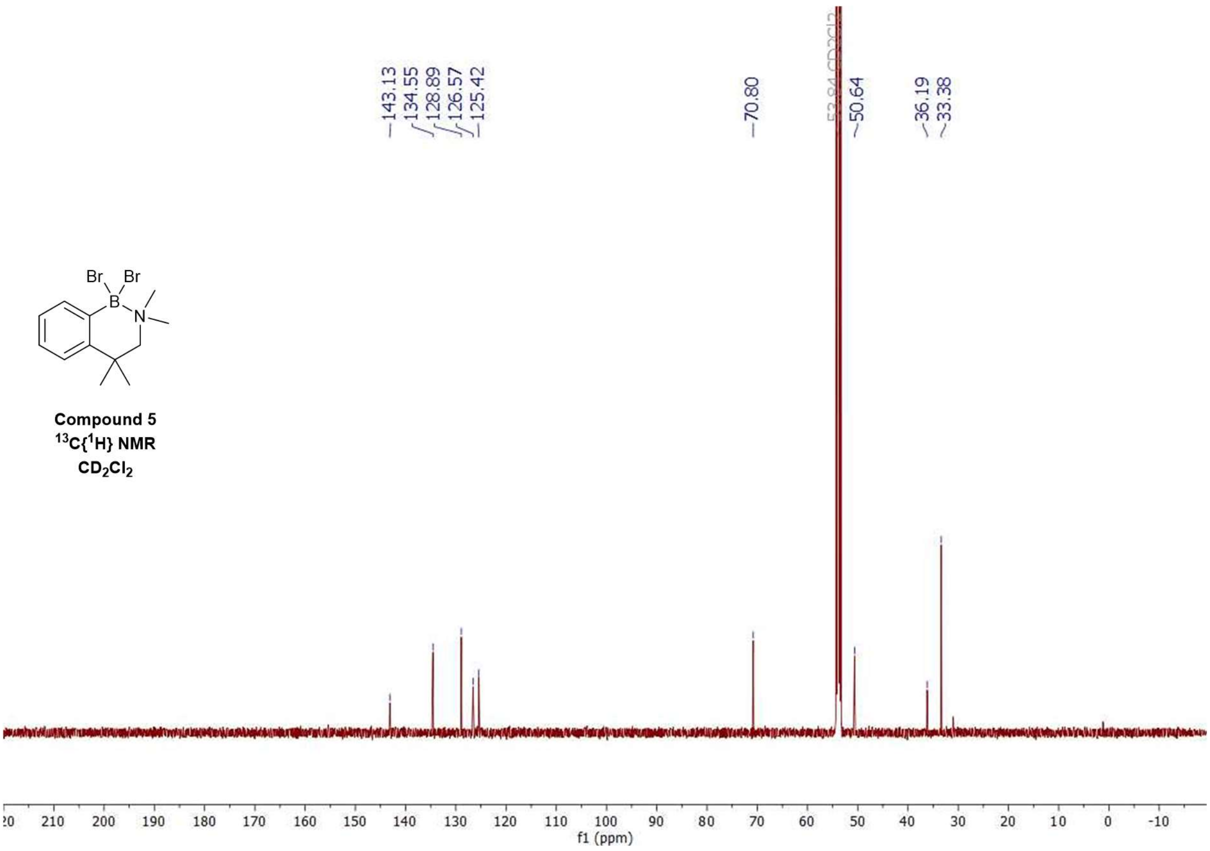

Figure S22. $^{13}C\{^{1}H\}$ NMR spectrum of compound **5** in CD_2Cl_2.

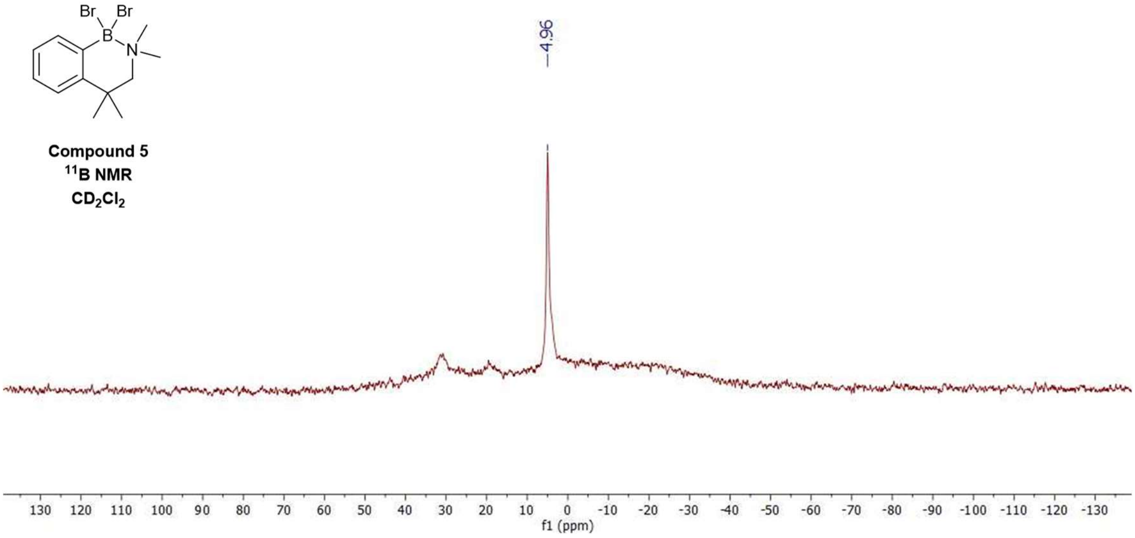

Figure S23. ^{11}B NMR spectrum of compound **5** in CD_2Cl_2. Note trace impurity at ca. +30 ppm is assigned to the product from reaction with water, presumably the boronic acid derived from hydrolysis of the B-Br bonds.

Compound 9

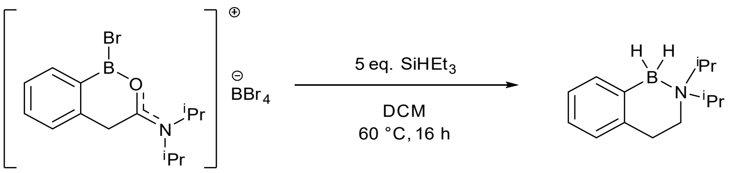

Compound **8** (0.320 g, 0.5 mmol) was added to an ampoule fitted with a J. Young valve. To the ampoule was added DCM (5 mL) and the suspension stirred while $SiHEt_3$ (0.4 mL, 5 eq.) was added dropwise. The ampoule was sealed and stirred at 60 °C for 16 h. The solvents/volatiles were removed under vacuum and the product dried to give a clear oil. The oil was redissolved in DCM (6 mL) and pentane (20 mL) was added slowly to induce precipitation. The precipitate was removed by filtration through a 0.22 μm syringe filter and the filtrate concentrated and dried under vacuum to give compound **9** (0.092 g, 84%) as a clear oil.

^{1}H{^{11}B} NMR (500 MHz, $CDCl_3$) δ 7.34 (d, J = 7.3 Hz, 1H), 7.11 (t, J = 7.3 Hz, 1H), 7.03 (t, J = 7.4 Hz, 1H), 6.97 (d, J = 7.5 Hz, 1H), 3.49 (hept, J = 6.6 Hz, 2H), 3.02 (t, J = 5.4 Hz, 2H), 2.92 (t, J = 5.4 Hz, 2H), 2.55 (br s, 2H), 1.36 (d, J = 6.6 Hz, 6H), 1.29 (d, J = 6.6 Hz, 6H). **^{11}B NMR** (160 MHz, $CDCl_3$) δ -10.41 (t, $^1J_{BH}$ = 90.05 Hz). **^{13}C{^{1}H} NMR** (126 MHz, CDCl3) δ 138.8, 132.4, 125.9, 125.4, 124.3, 59.4, 51.0, 31.4, 19.3, 18.7. **[Acc. Mass]**. calcd for [M]=$C_{14}H_{24}BN$: 217.1996, found 217.1998.

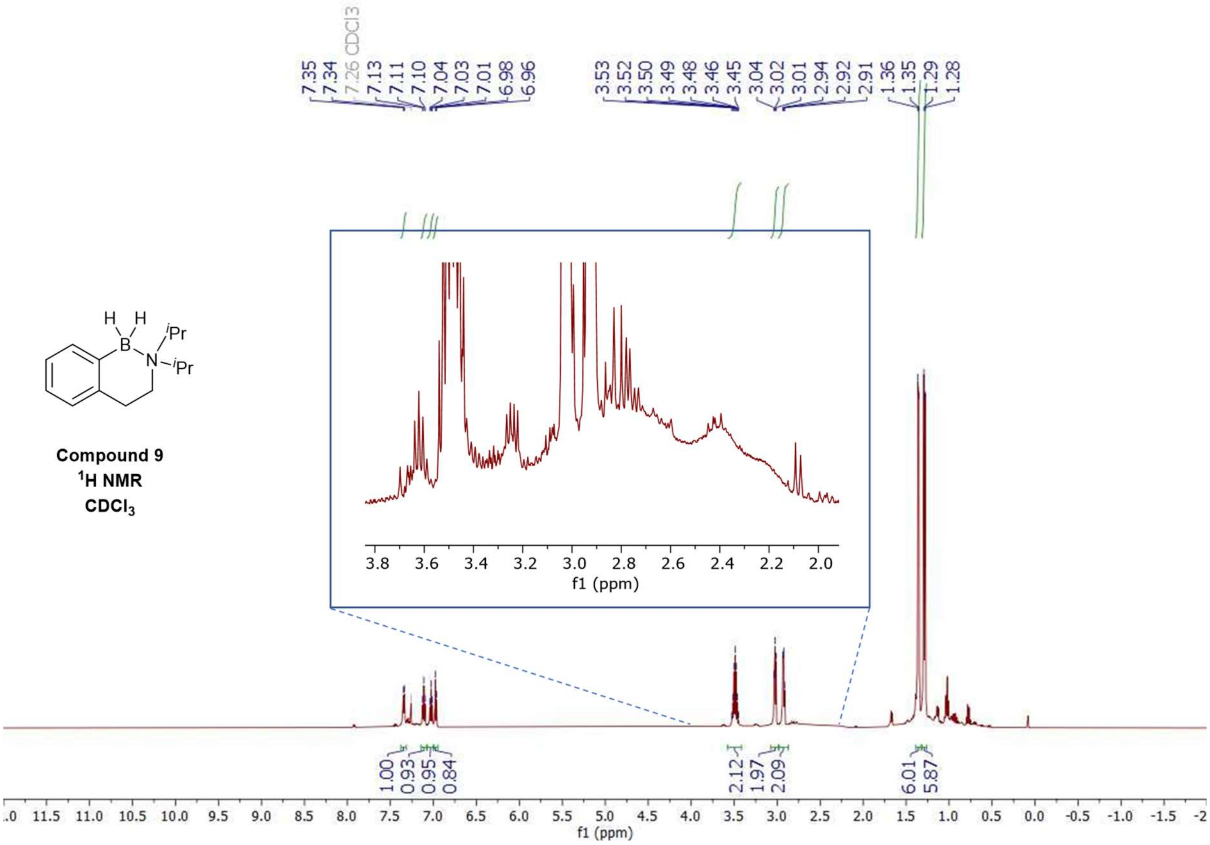

Figure S24. ^{1}H NMR spectrum of compound **9** in $CDCl_3$. The inset shows the broad B-H resonances overlapped with the aliphatic resonances.

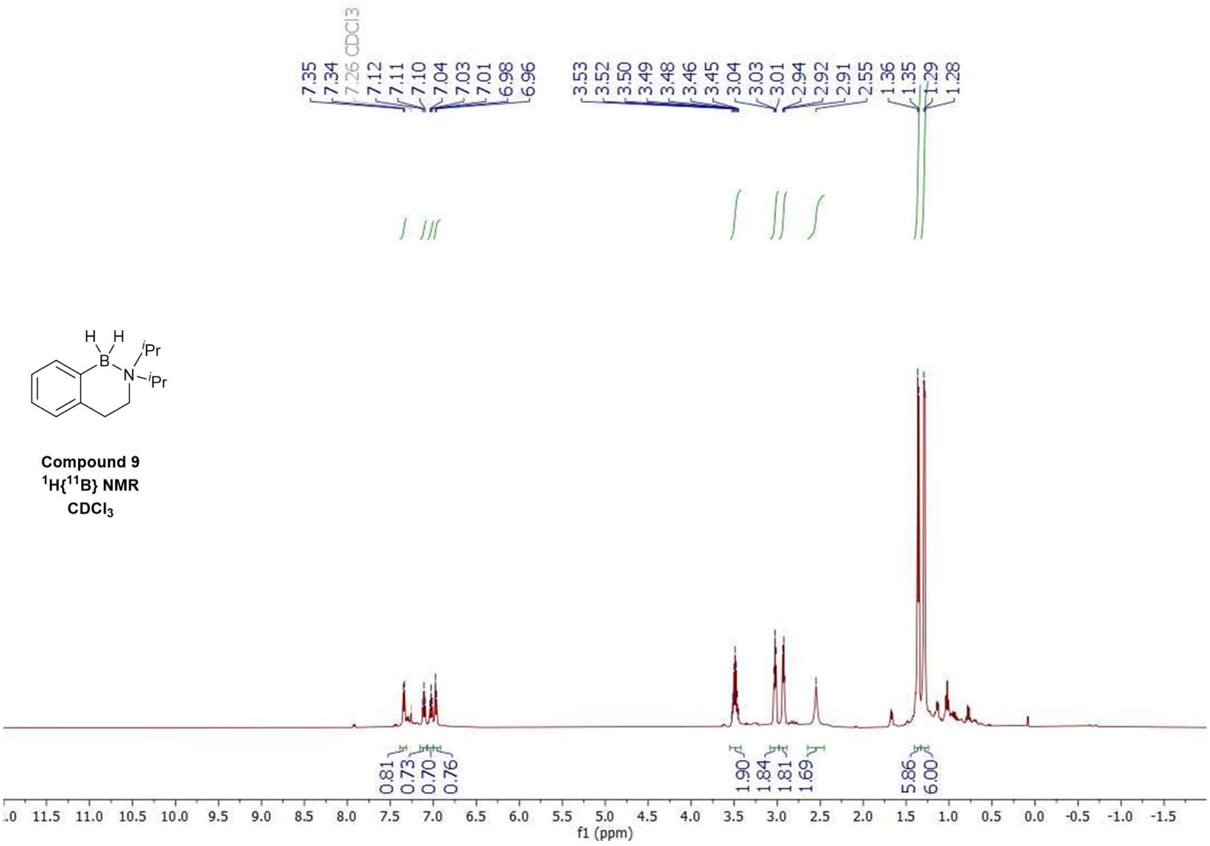

Figure S25. $^{1}H\{^{11}B\}$ NMR spectrum of compound **9** in $CDCl_3$.

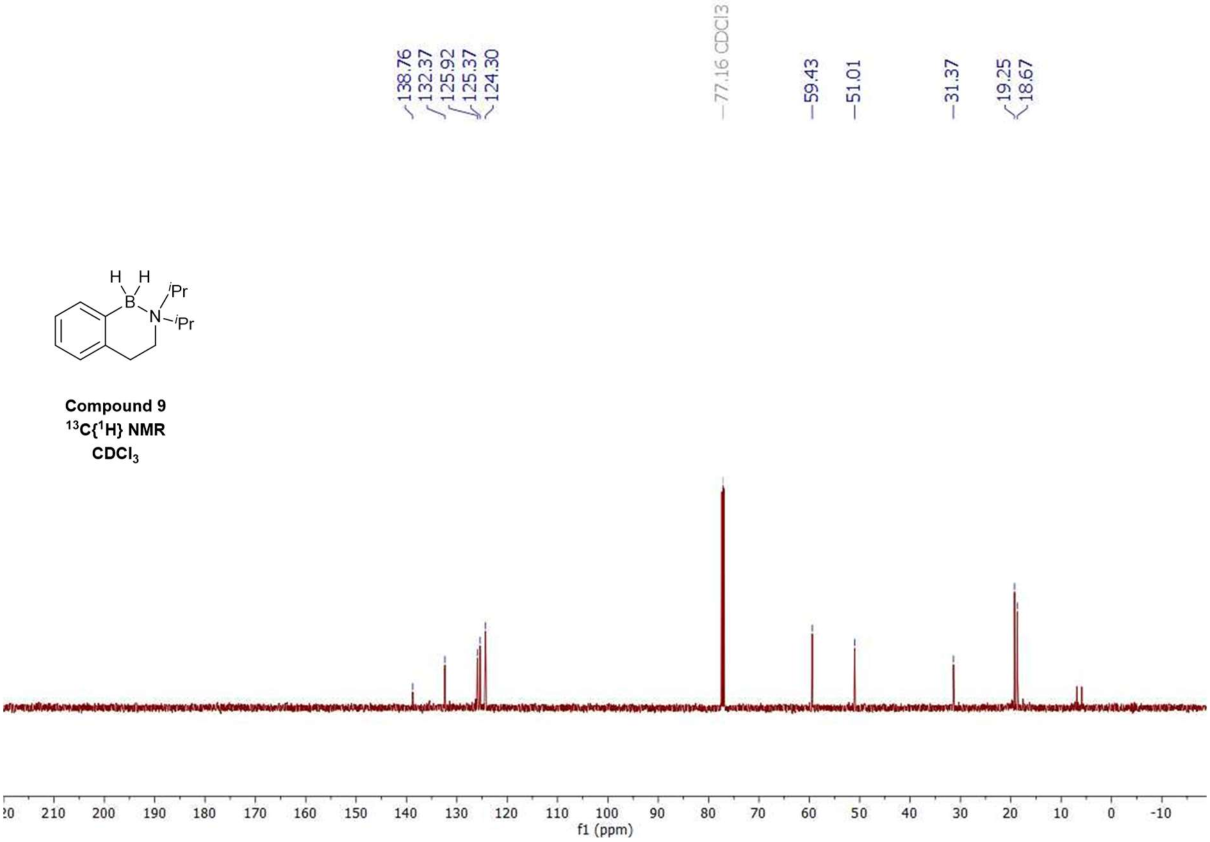

Figure S26. $^{13}C\{^{1}H\}$ NMR spectrum of compound **9** in $CDCl_3$.

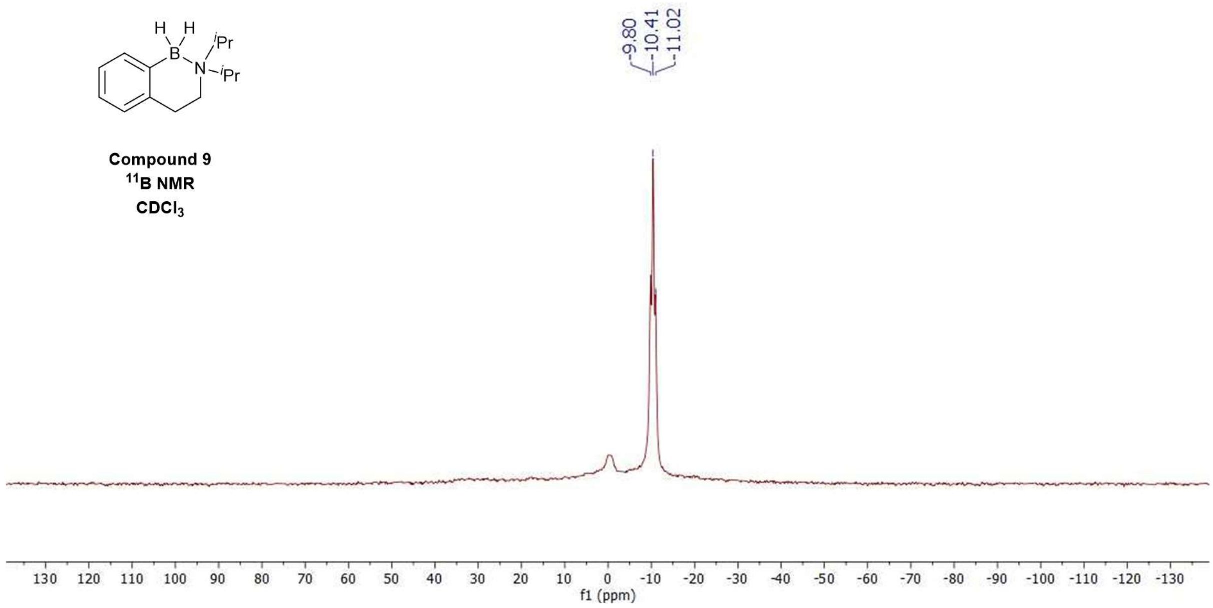

Figure S27. ^{11}B NMR spectrum of compound **9** in $CDCl_3$.

Compound 7

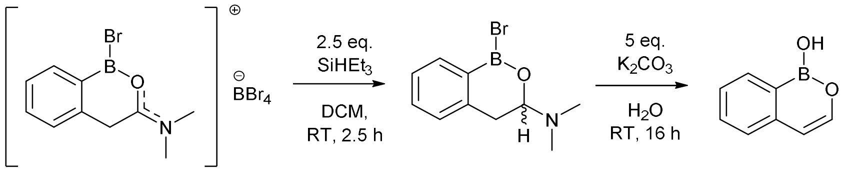

1[BBr$_4$] (0.058 g, 0.1 mmol) was added to an NMR tube fitted with a J. Youngs valve. The solid was dissolved in DCM (0.7 mL) and $SiHEt_3$ (2.5 eq. 40 μL) added, the tube was sealed and rotated at room temperature for 2.5 h after which a solution of K_2CO_3 (0.07 g, 5 eq.) in H_2O (0.6 mL) was added, the tube was shaken vigorously and rotated at room temperature for 16 h. The product was extracted with DCM. The organic fraction was dried over $MgSO_4$ and concentrated *in vacuo*. *Yield determination versus mesitylene* added *as an internal standard showed* 26% *yield*. Column chromatography on silica gel (EtOAc:PET) gave **7** (0.002 g, 14%) as a clear oil. Analytical data for **7** consistent with the literature report.[4]

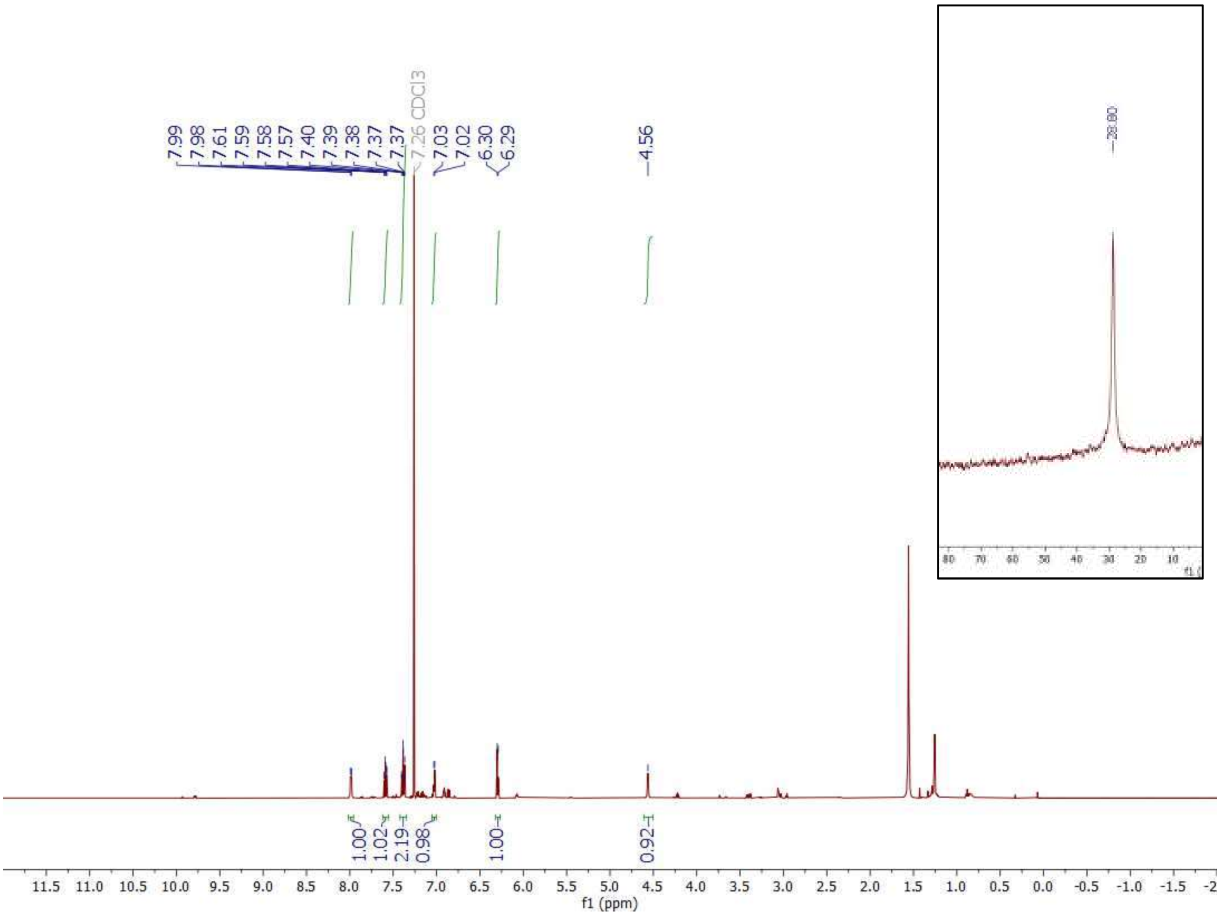

Figure S28. Crude ^{1}H NMR spectrum of **7** in $CDCl_3$. Inset the ^{11}B NMR spectrum for **7**.

Procedure for 2-(2-(dibromoboraneyl)phenyl)-*N,N*-dimethylethan-1-amine oxidation:

2-(2-(dimethylamino)ethyl)phenol (Compound 6)

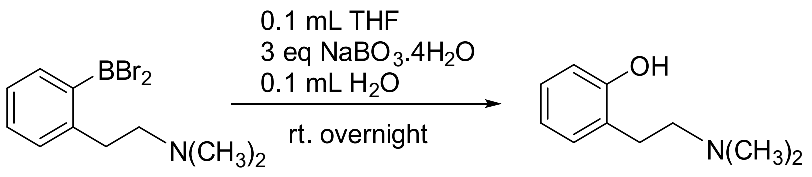

To an oven-dried ampoule 2-(2-(dibromoboraneyl)phenyl)-*N,N*-dimethylethan-1-amine (10 mg, 0.03 mmol, 1 eq), and dry THF (0.1 mL) was added. To which $NaBO_3{\cdot}4H_2O$ (14 mg, 0.09 mmol, 3 eq) was added followed by water (0.1 mL). The reaction mixture was allowed to stir at room temperature overnight. The mixture was dried over $MgSO_4$, filtered, and NMR spectra were taken. The NMR data showed quantitative conversion into desired phenol product as the only species present. **[Acc. Mass]** Calculated for $[M+H]^+$: 166.1226, found $[M+H]^+$ 166.1220.

^{1}H NMR (500 MHz, $CDCl_3$, 298 K): δ 7.12 (td, J=7.7, 1.7 Hz, 1H), 7.01 (dd, J=7.4, 1.7 Hz, 1H), 6.89 (dd, J=8.0, 1.2 Hz, 1H), 6.75 (td, J=7.3, 1.2 Hz, 1H), 2.91 (dd, J=6.8, 4.7 Hz, 2H), 2.81 (dd, J=6.8, 4.7 Hz, 2H), 2.51 (s, 6H). **^{13}C{^{1}H} NMR** (126 MHz, $CDCl_3$, 298 K): δ 156.8, 130.9, 128.6, 126.3, 119.4, 117.6, 59.7, 44.5, 30.7.

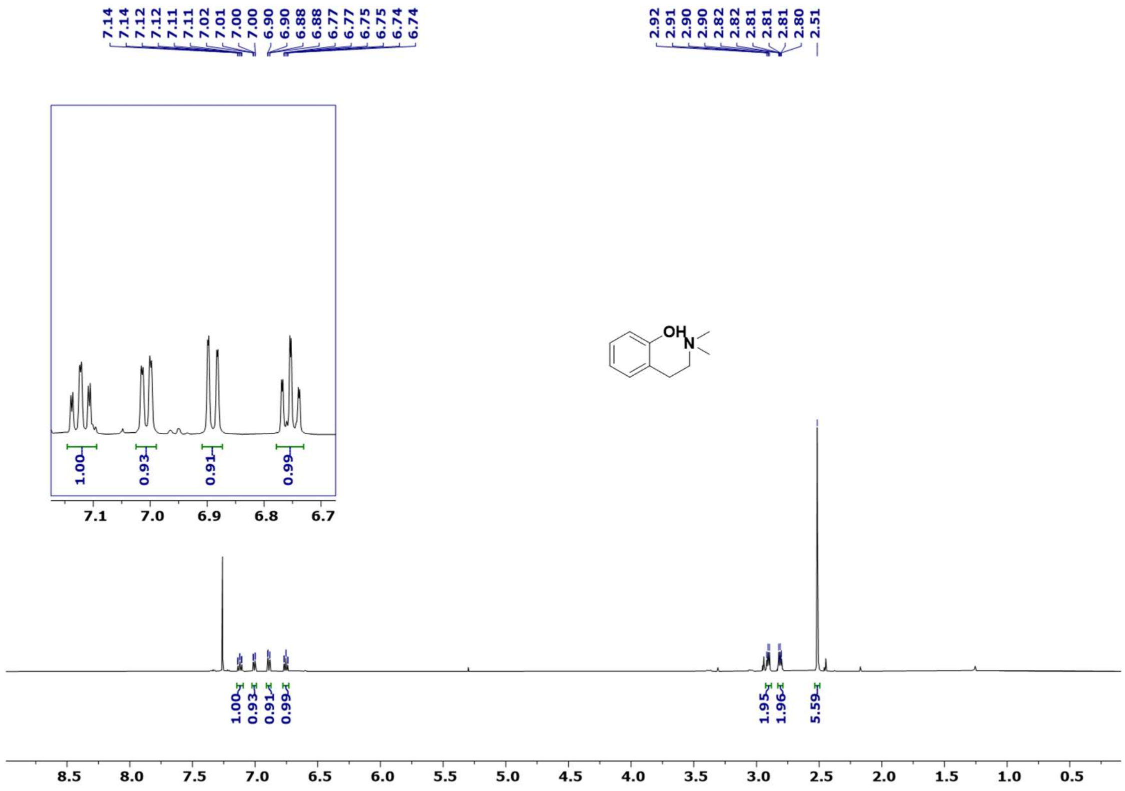

Figure S29. ^{1}H NMR spectrum of **6** in $CDCl_3$ – without any additional purification.

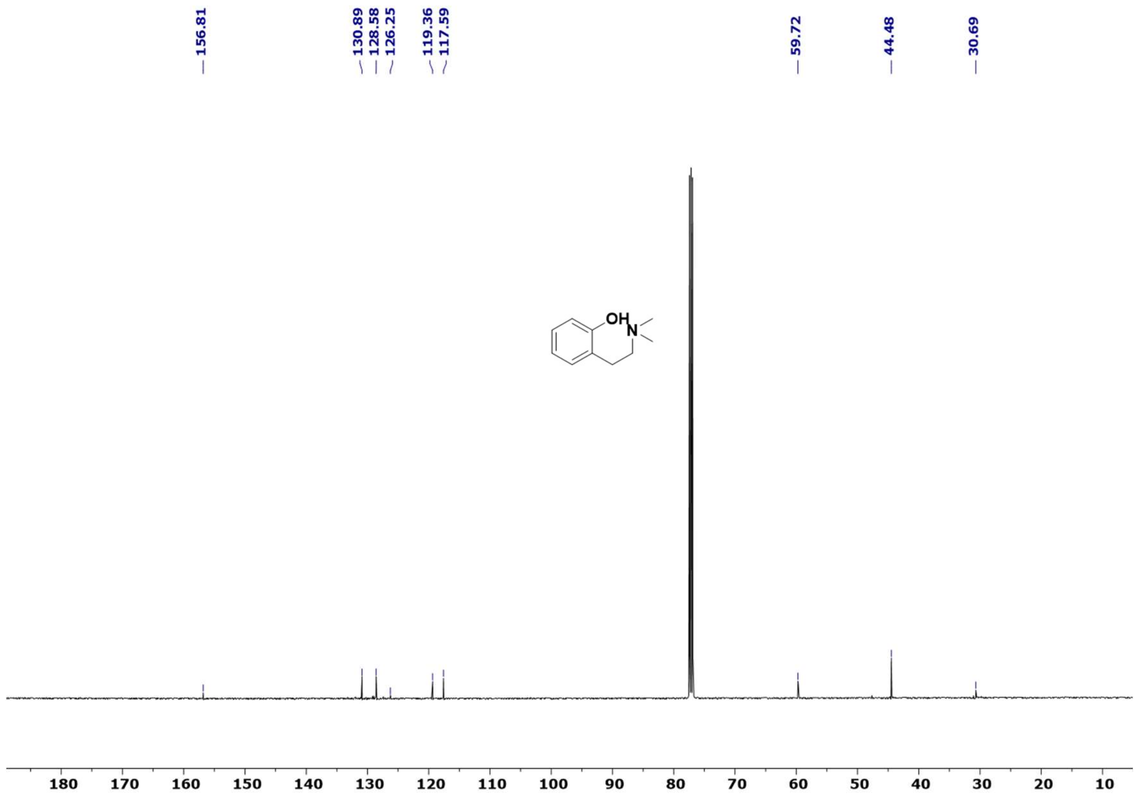

Figure S30. $^{13}C\{^{1}H\}$ NMR spectrum of **6** without any additional purification in $CDCl_3$.

4 Borylation-Reduction of Aniline Derivatives

The synthesis of *ortho* borylated anilines, **10-Br** and **S5**, was based on our previously reported procedure.[2]

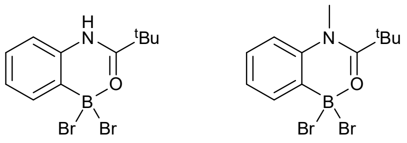

10-Br **S5**

Attempted direct reduction of **10-Br**

Multiple attempts were made to reduce **10-Br** directly, this involved varying the equivalents of silane and reaction duration, however, unreacted amide was always present in these reactions (vide infra). The addition of an internal standard (CH_2Br_2) enabled estimation of conversions in these reactions. Note attempts to isolate **11-Pin** and **12-Pin** from silane by-product was not possible in our hands.

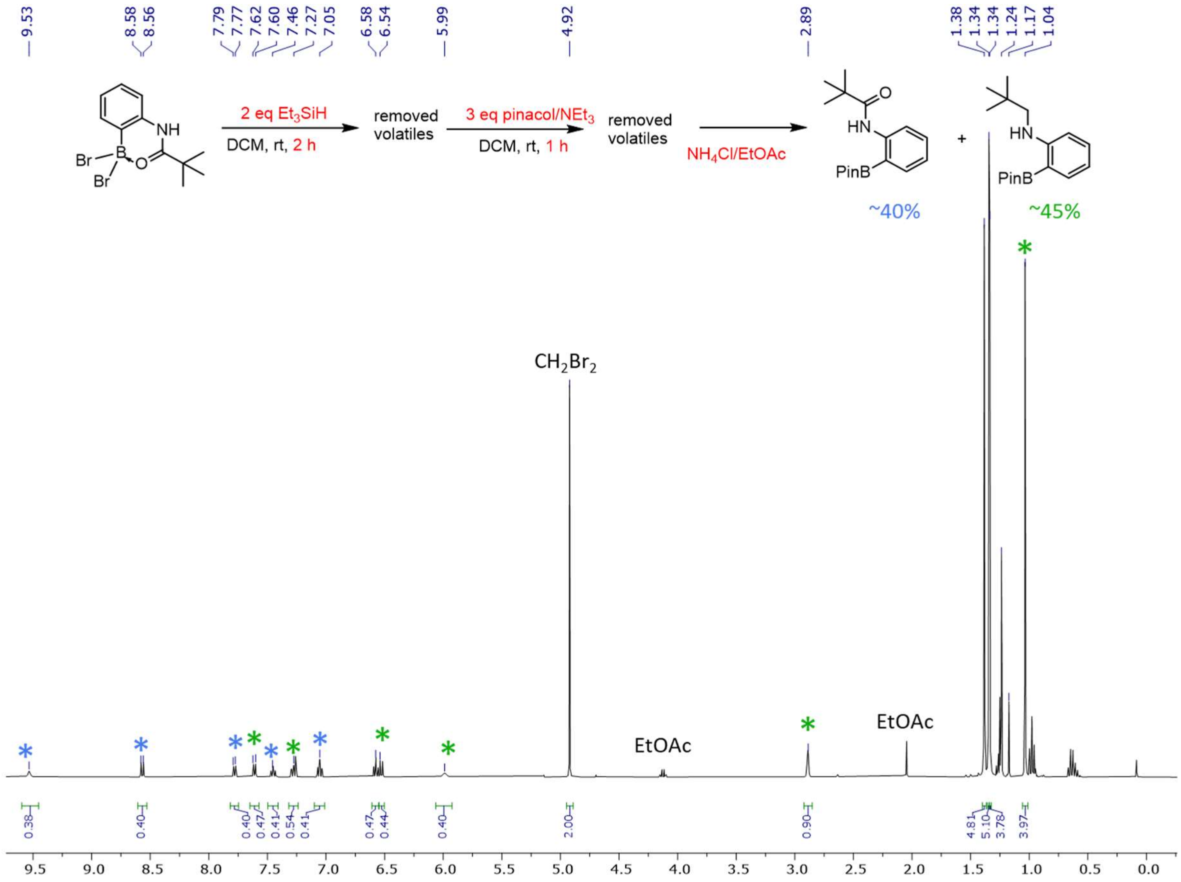

Figure S31. In-situ ^{1}H NMR spectrum of the crude reaction mixture from the reduction of **10-Br**.

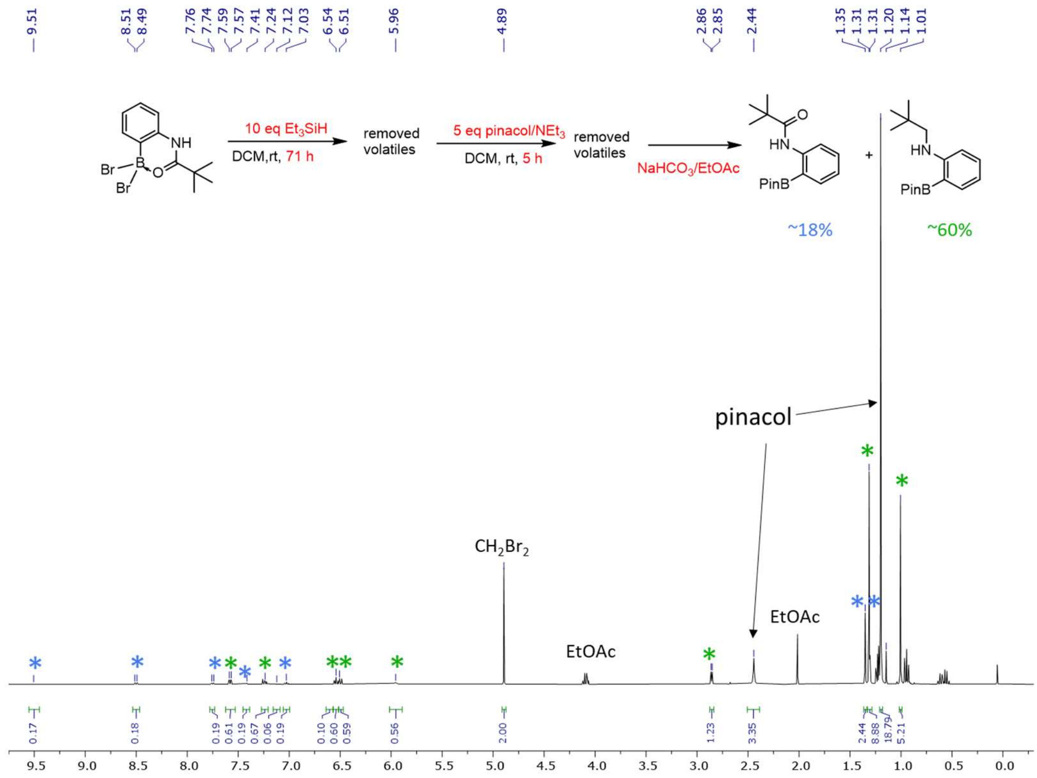

Figure S32. In-situ ^{1}H NMR spectrum of the crude reaction mixture from the reduction of **10-Br** using a large excess of hydrosilanes and long reaction time.

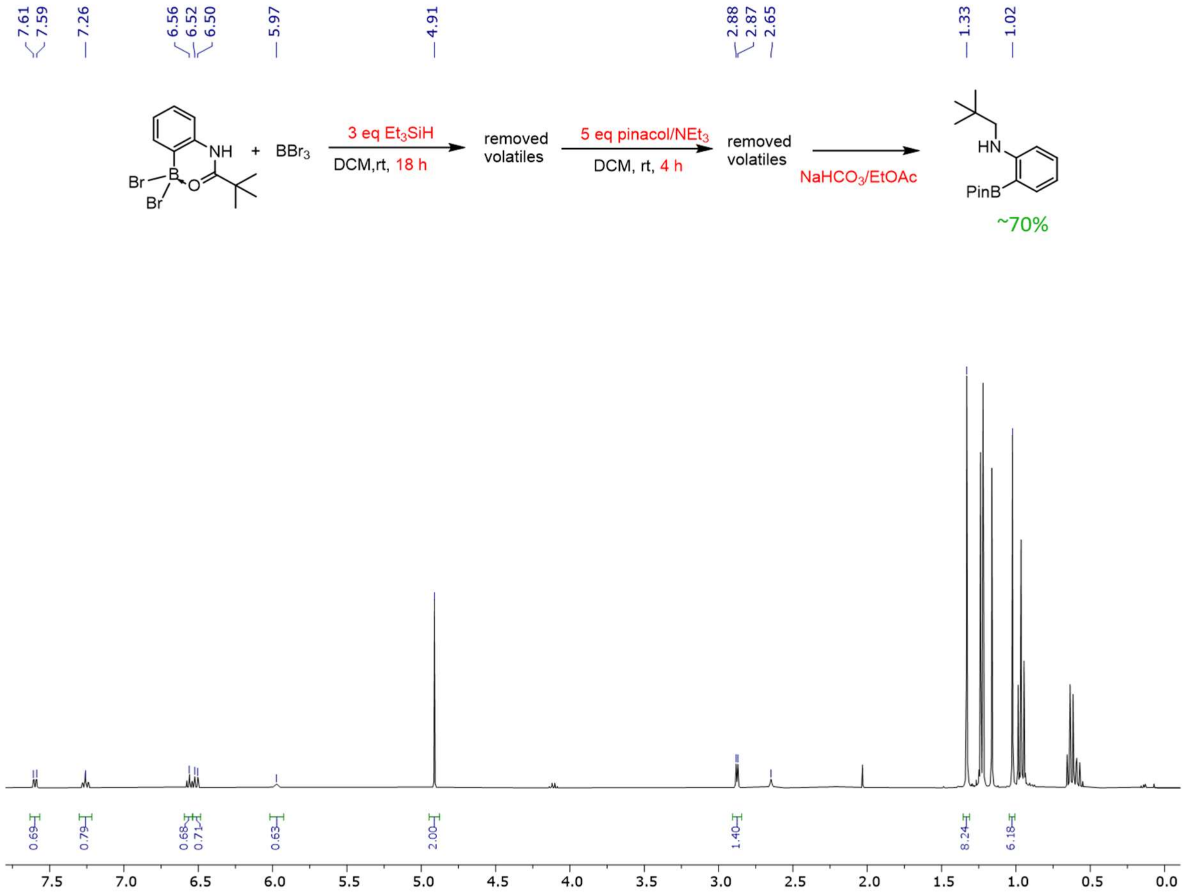

Figure S33. In-situ ^{1}H NMR spectrum of the crude reaction mixture (with CH_2Br_2 added) from the reduction of **10[BBr4]**.

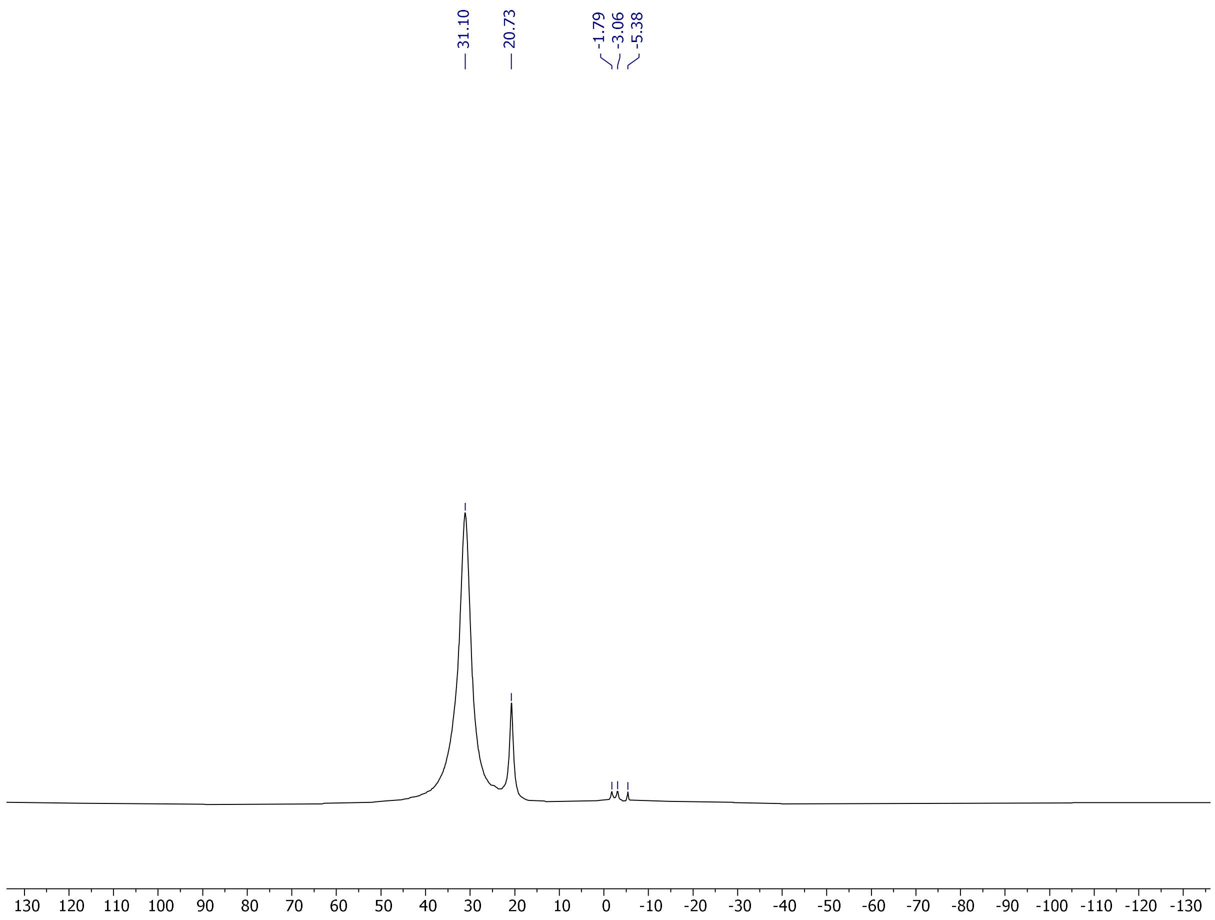

Figure S34. In-situ ^{11}B NMR spectrum of the crude reaction mixture (with CH_2Br_2 added) from the reduction of **10[BBr$_4$]**.

Compound 11-Dan

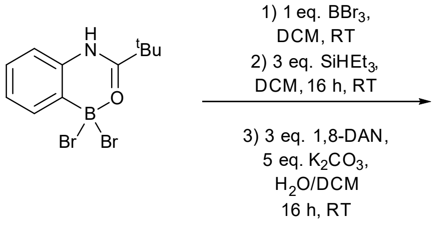

10-Br (0.035g, 0.1 mmol) was added to an ampoule fitted with a J. Young valve. The ampoule was evacuated and refilled with N_2. To this was added DCM (2 mL) followed by BBr_3 1M in heptanes (0.1 mmol, 0.1 mL) – to convert it to **10[BBr$_4$]**. To the stirring solution was added $SiHEt_3$ (0.3 mmol, 48 μL), the ampoule was sealed and stirred overnight at room temperature. The volatiles were removed, and the crude intermediate was dried under vacuum. To the ampoule was added a biphasic solution of K_2CO_3 (0.5 mmol, 0.069 g) in H_2O (0.8 mL) and 1,8-diaminonaphtalene (0.3 mmol, 0.047 g) in DCM (0.8 mL). The solution was stirred vigorously for 16 h. The contents of the reaction were diluted with DCM (20 mL) and dried over $MgSO_4$, filtered and volatiles were removed *in vacuo* and the yield was calculated by NMR spectroscopy to be 44% using CH_2Br_2 as an internal standard. The crude product was purified by flash chromatography on silica-gel (DCM/PET) to give the pure product (0.008 g, 24%).

^{1}H NMR (500 MHz, $CDCl_3$) δ 7.38 (dd, *J* = 7.2, 1.7 Hz, 1H), 7.30 (m, 1H), 7.14 (dd, *J* = 8.3, 7.2 Hz, 2H), 7.06 (dd, *J* = 8.3, 1.0 Hz, 2H), 6.79 (bs, 1H), 6.70 (s, 1H), 6.42 – 6.33 (m, 2H), 6.18 (s, 2H), 4.15 (s, 1H), 2.91 (s, 2H), 1.05 (s, 9H). **^{11}B NMR** (160 MHz, $CDCl_3$) δ 30.0. **^{13}C{^{1}H} NMR** (126 MHz, $CDCl_3$) δ 152.3, 141.1, 136.5, 133.4, 131.5, 127.8, 120.0, 118.1,117.8, 110.9, 106.2, 56.3 31.7, 28.1. **[Acc. Mass]**. calcd for $C_{21}H_{24}BN_3$ = 329.2058, found = 329.2066.

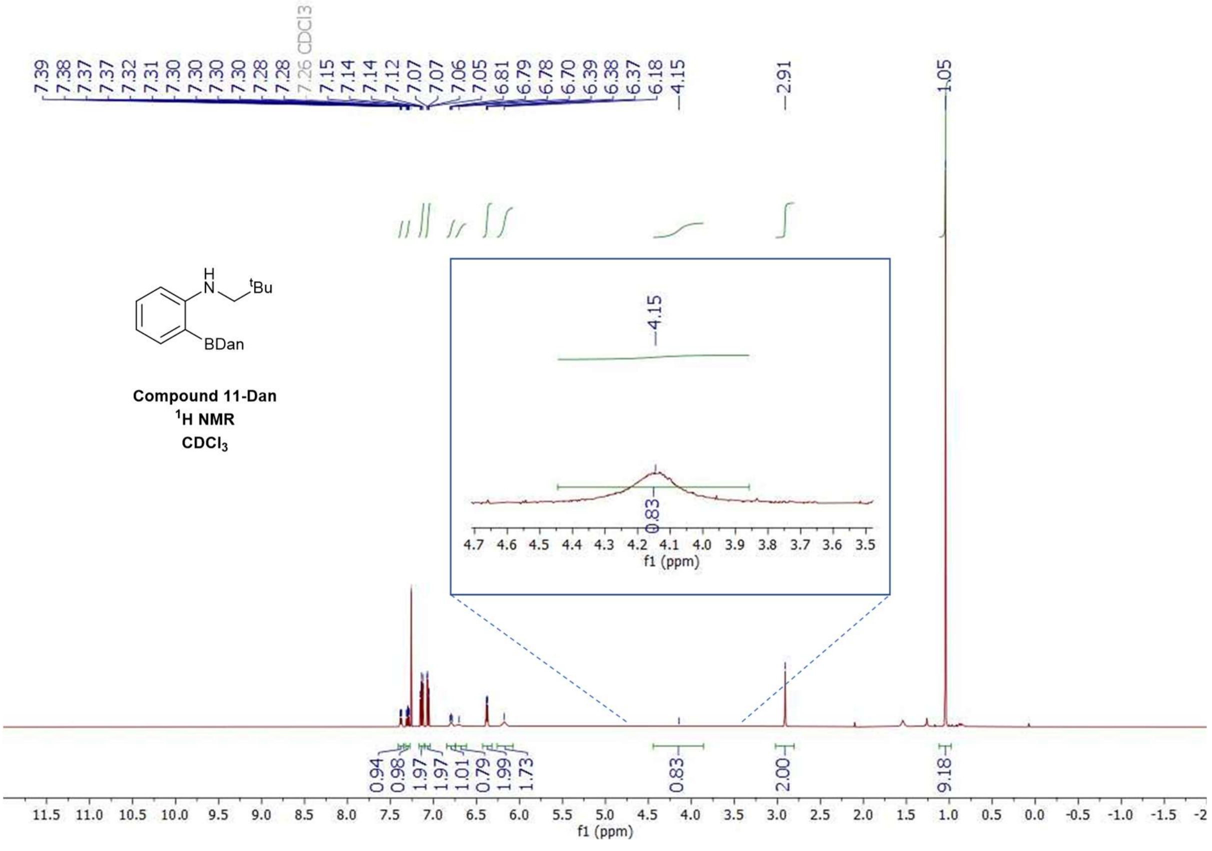

Figure S35. ^{1}H NMR spectrum of compound **11-Dan** in $CDCl_3$.

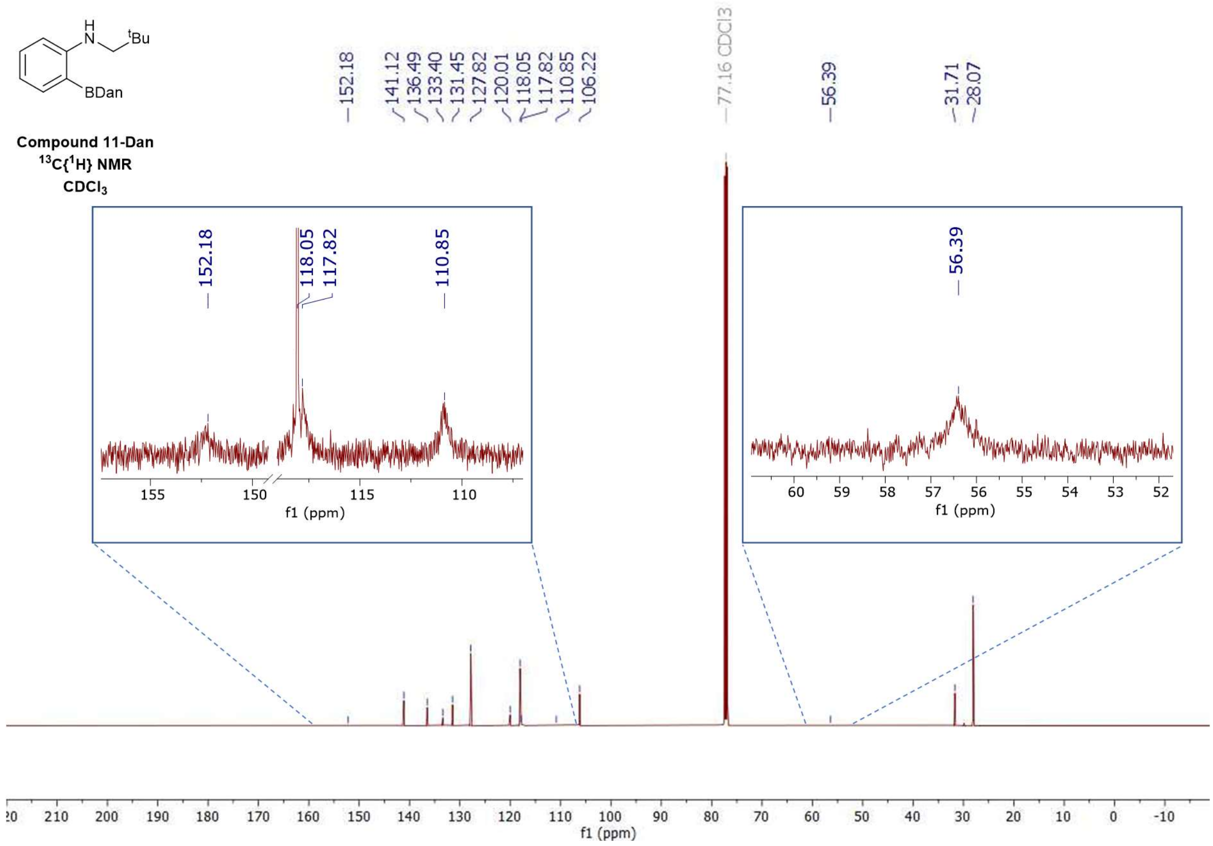

Figure S36. $^{13}C\{^1H\}$ NMR spectrum of compound **11-Dan** in $CDCl_3$.

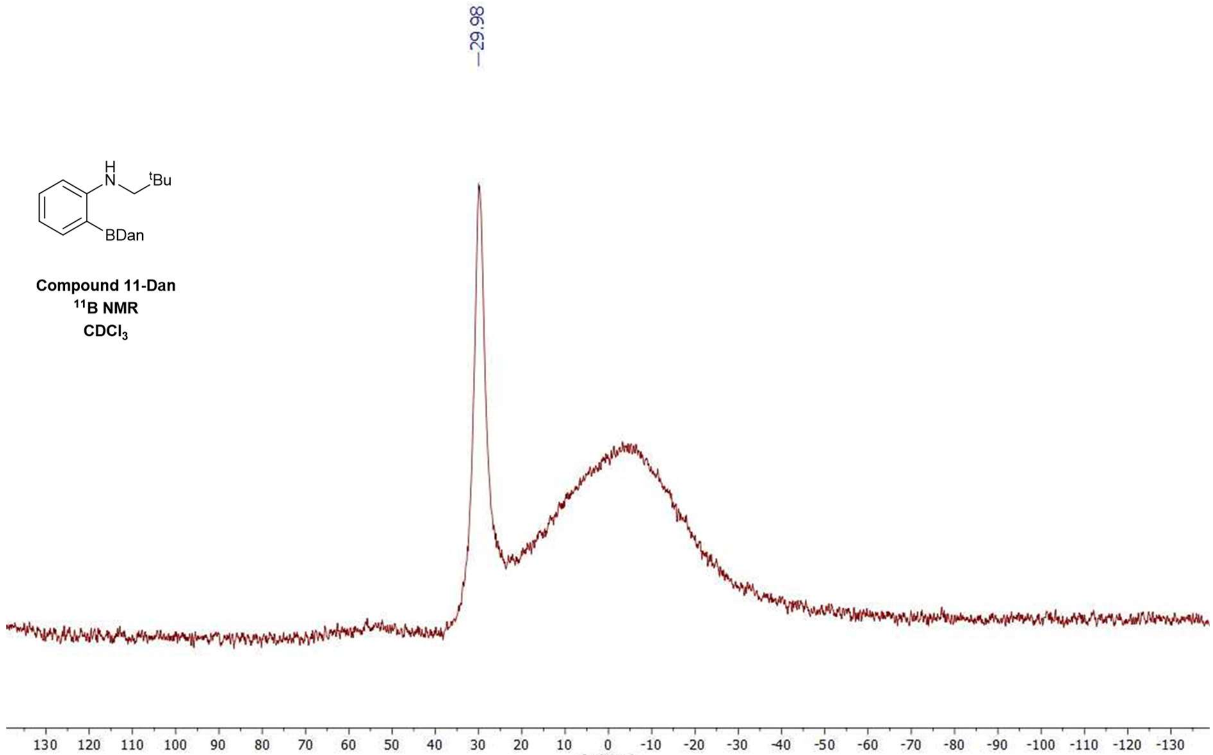

Figure S37. ^{11}B NMR spectrum of compound **11-Dan** in $CDCl_3$.

Compound 12

Note in this case, overnight with 3 eq. of hydrosilanes is sufficient to reduce the amide in the neutral species, **S5**. Though the borenium analogue of **S5** (formed by addition of an additional equivalent of BBr_3) undergoes full reduction of the amide to the amine within 2 h at room temperature using 2 equiv. $HSiEt_3$, indicating the borenium is still more reactive.

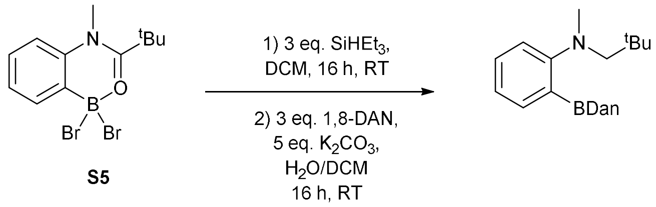

Compound **S5** (0.108g, 0.3 mmol) was added to an ampoule fitted with a J. Young valve. The ampoule was evacuated and refilled with N_2. To this was added DCM (5 mL) followed $SiHEt_3$ (0.9 mmol, 0.15 mL) dropwise, the ampoule was sealed and stirred overnight at room temperature. The volatiles were removed, and the crude intermediate was dried under vacuum. The residue was redissolved in DCM (4 mL) and a biphasic solution of K_2CO_3 (0.15 mmol, 0.207 g) in H_2O (1 mL) and 1,8-diaminonaphtalene (0.9 mmol, 0.14 g) in DCM (1 mL). The solution was stirred vigorously for 16 h. The contents of the reaction were diluted with DCM (20 mL) and dried over $MgSO_4$, filtered and volatiles were removed *in vacuo* and the yield was calculated by NMR to be 76% using 1,2-dibromoethane as an internal standard. The crude product was purified by flash chromatography on silica-gel (DCM/PET) to give the pure product (0.023 g, 22%).

^{1}H NMR (500 MHz, $CDCl_3$) δ 7.54 (dd, *J* = 7.4, 1.7 Hz, 1H), 7.38 (m, 1H), 7.27 (d, *J* = 8.5 Hz, 1H), 7.14 (dd, *J* = 8.3, 7.3 Hz, 2H), 7.08 (td, *J* = 7.3, 1.1 Hz, 1H), 7.04 (dd, *J* = 8.3, 1.0 Hz, 2H), 6.99 (s, 2H), 6.39 (dd, *J* = 7.3, 1.0 Hz, 2H), 3.04 (s, 2H), 2.89 (s, 3H), 1.01 (s, 9H). **^{11}B NMR** (160 MHz, $CDCl_3$) δ 29.7. **^{13}C{^{1}H} NMR** (126 MHz, $CDCl_3$) δ 161.0, 141.8, 136.6, 133.4, 131.0, 127.8, 122.6, 121.0, 120.1, 117.4, 105.7, 67.7, 50.0, 34.2, 29.0. **[Acc. Mass]**. calcd for $C_{22}H_{26}BN_3$ = 343.2214, found = 343.2226.

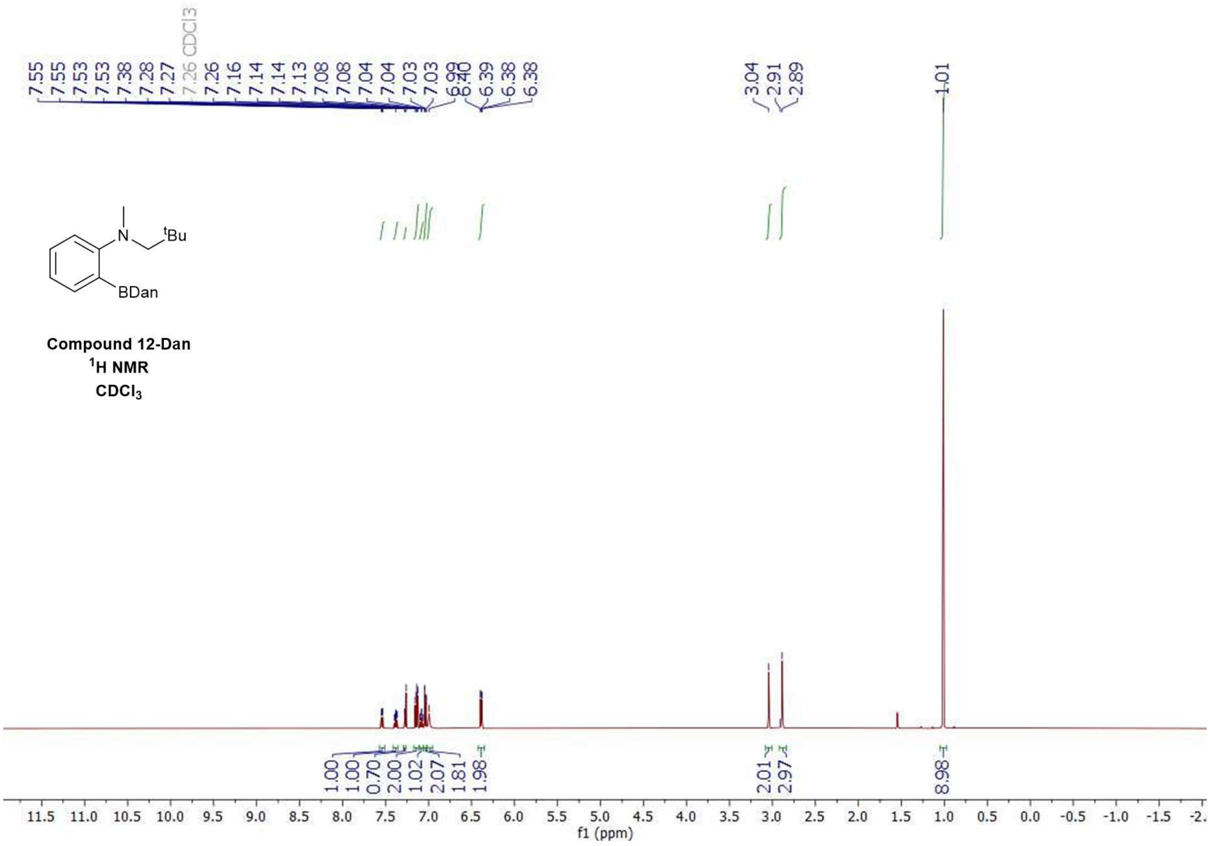

Figure S38. ^{1}H NMR spectrum of compound **12** in $CDCl_3$.

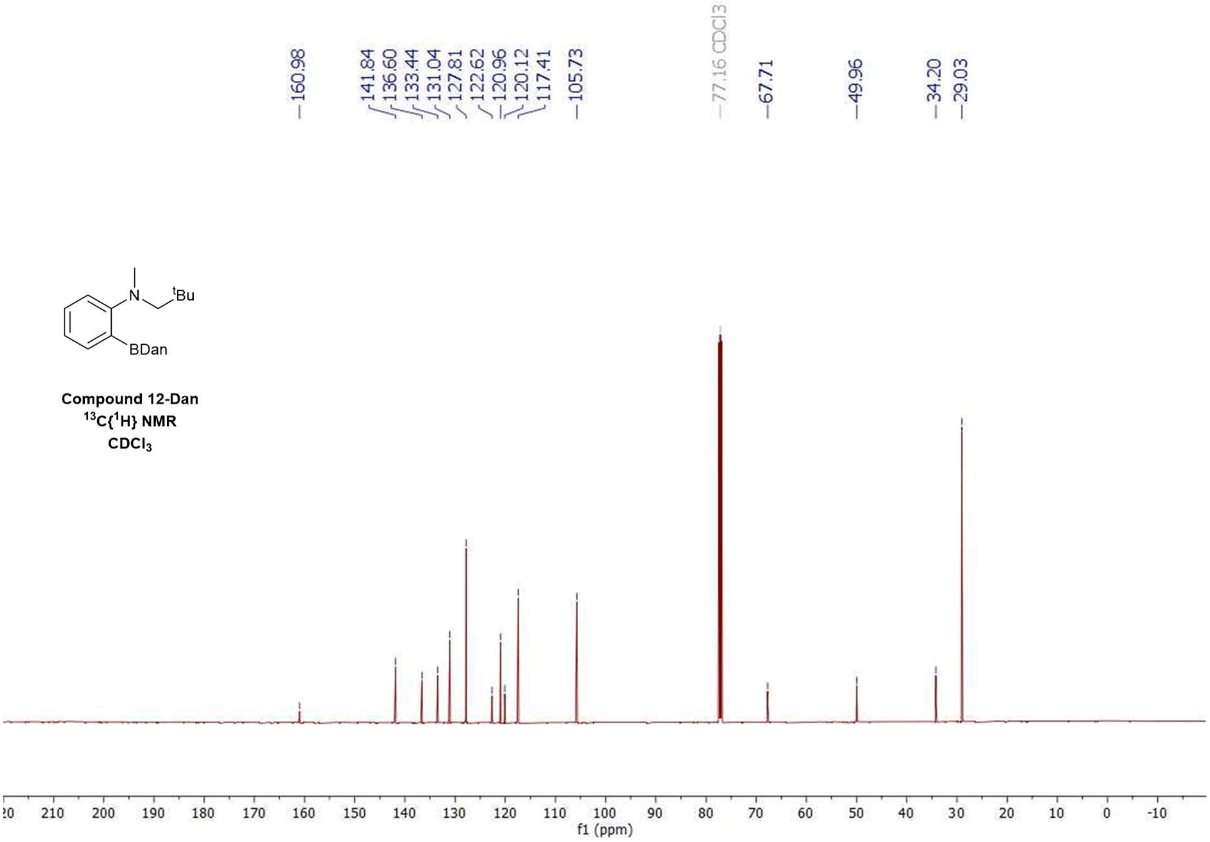

Figure S39. $^{13}C\{^{1}H\}$ NMR spectrum of compound **12** in $CDCl_3$.

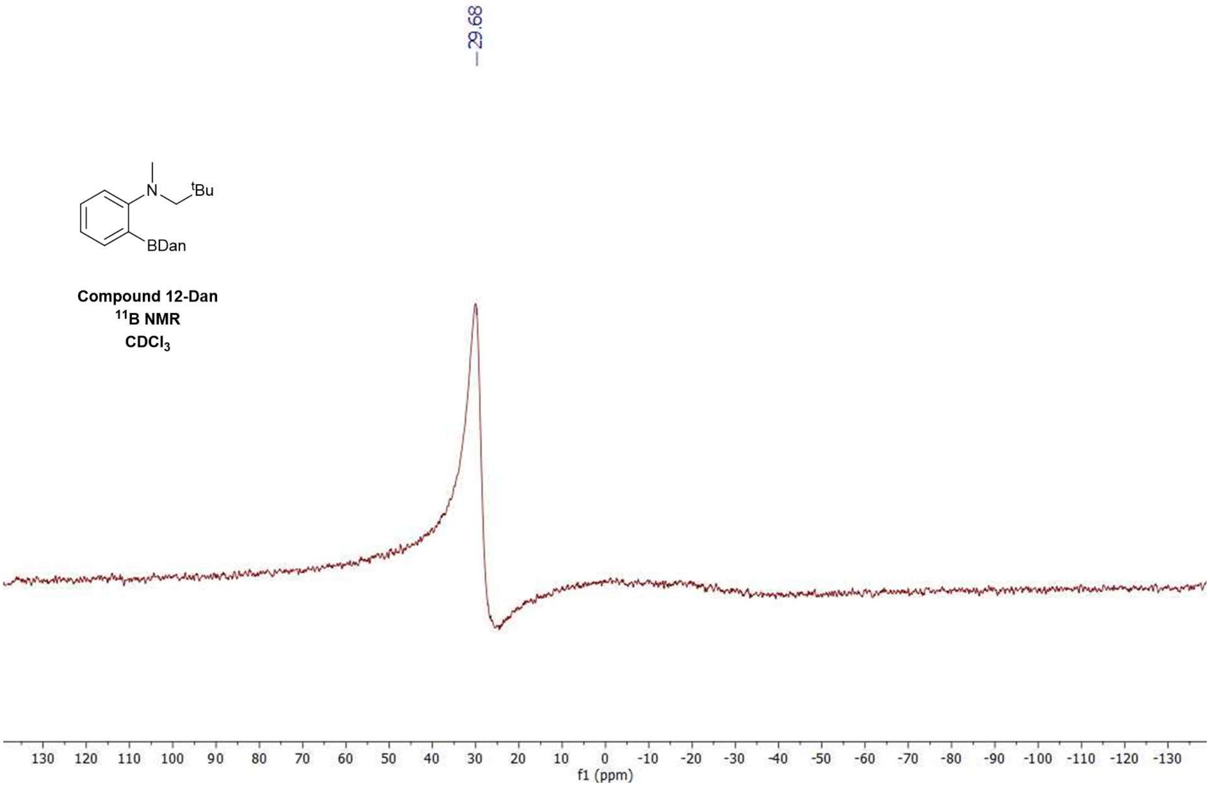

Figure S40. ^{11}B NMR spectrum of compound **12** in $CDCl_3$.

Compound 13

***N*-benzyl-2-(1H-naphtho[1,8-de][1,3,2]diazaborinin-2(3H)-yl)aniline (3c)**

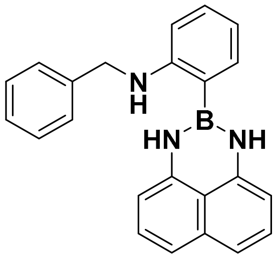

Compound **13** was prepared using benzanilide (138 mg, 0.7 mmol, 1 eq) dissolved in DCM (0.5 mL). To the stirred solution was added BBr_3 (1M solution in DCM) (0.8 mL, 0.8 mmol, 1.1 eq) and heated at 60 °C (in a sealed ampoule) for 18 h. The white suspension obtained was evaporated and dried under vacuum to get a dry solid. Dry DCM (1 mL) and Et_3SiH (0.3 mL, 1.5 mmol, 2.2 eq) were then added and the mixture was stirred at room temperature overnight. The clear solution obtained was evaporated and dried under a vacuum, to which the biphasic solution of DAN (133 mg, 0.8 mmol, 1.2 eq in 0.5 mL DCM) and K_2CO_3 (676 mg, 4.9 mmol, 7 eq in 1 mL H_2O) was added and stirred at room temperature overnight. $MgSO_4$ was added to the reaction mixture diluted with DCM and left for an hour. The mixture was filtered, and volatiles removed under vacuum to get crude product which was purified by column chromatography on silica gel (petroleum ether/ DCM = 8:2) to get a maroon sticky solid (120 mg, 49%). **[Acc. Mass]** Calculated for $C_{23}H_{21}B_1N_3$ $[M+H]^+$: 350.1823, found $[M+H]^+$ 250.1827.

^{1}H NMR (500 MHz, $CDCl_3$, 298 K): δ 7.41-7.35 (m, 5H), 7.32-7.26 (m, 2H), 7.14 (dd, *J*=8.3, 7.3 Hz, 2H), 7.06 (dd, *J*=8.3, 1.0 Hz, 2H), 6.86-6.81 (m, 1H), 6.70 (d, *J*=8.2 Hz, 1H), 6.35 (dd, *J*=7.2, 1.0 Hz, 2H), 6.10 (br s, 2H), 4.47 (br s, 1H), 4.38 (s, 2H). **^{13}C{^{1}H} NMR** (126 MHz, $CDCl_3$, 298 K): δ 151.2, 141.0, 139.3, 136.4, 133.3, 131.3, 128.9, 127.7, 127.5, 120.0, 118.3, 118.3, 118.1, 112, 106.2, 99.0. **^{11}B NMR** (160 MHz, CD_2Cl_2, 298 K): δ 30.1.

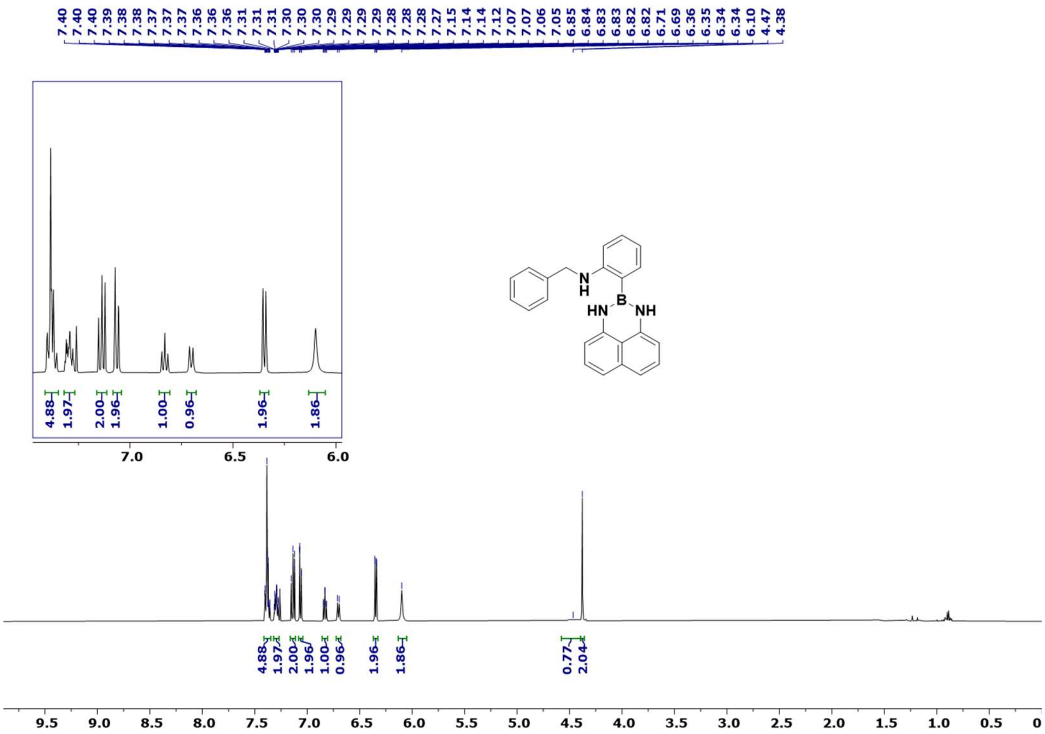

Figure S41. ^{1}H NMR spectrum of **13** in $CDCl_3$.

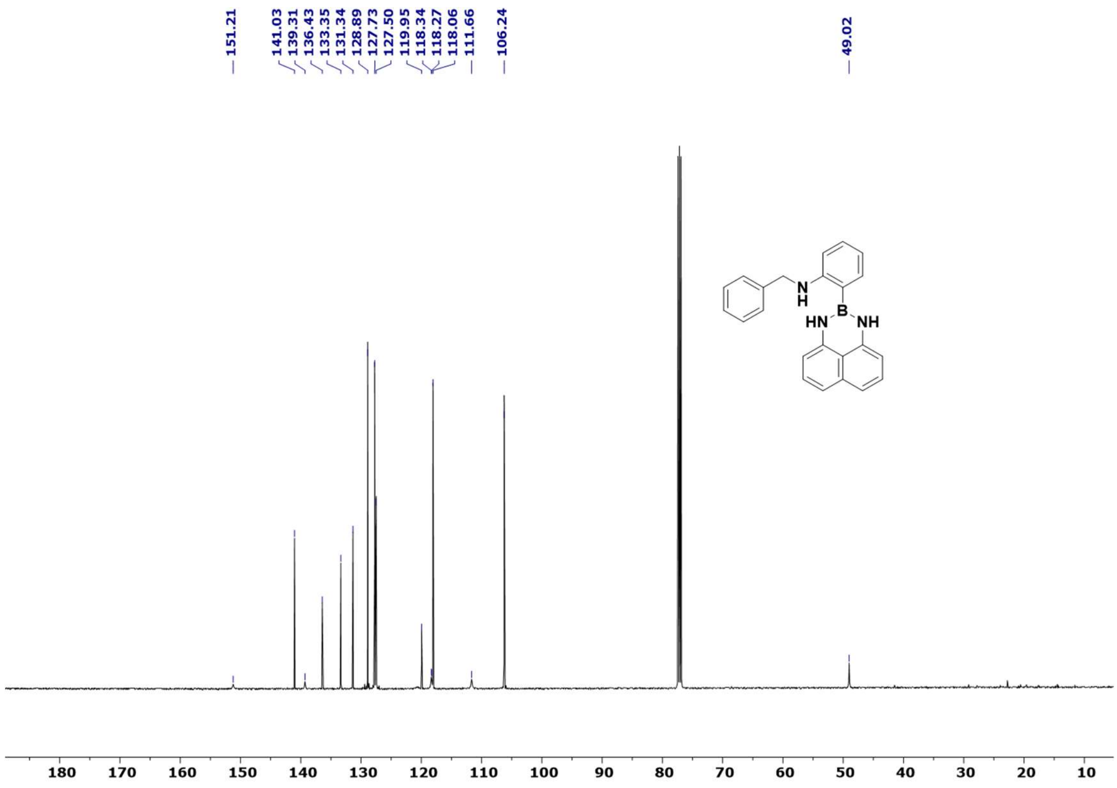

Figure S42. $^{13}C\{^{1}H\}$ NMR spectrum of **13** in $CDCl_3$.

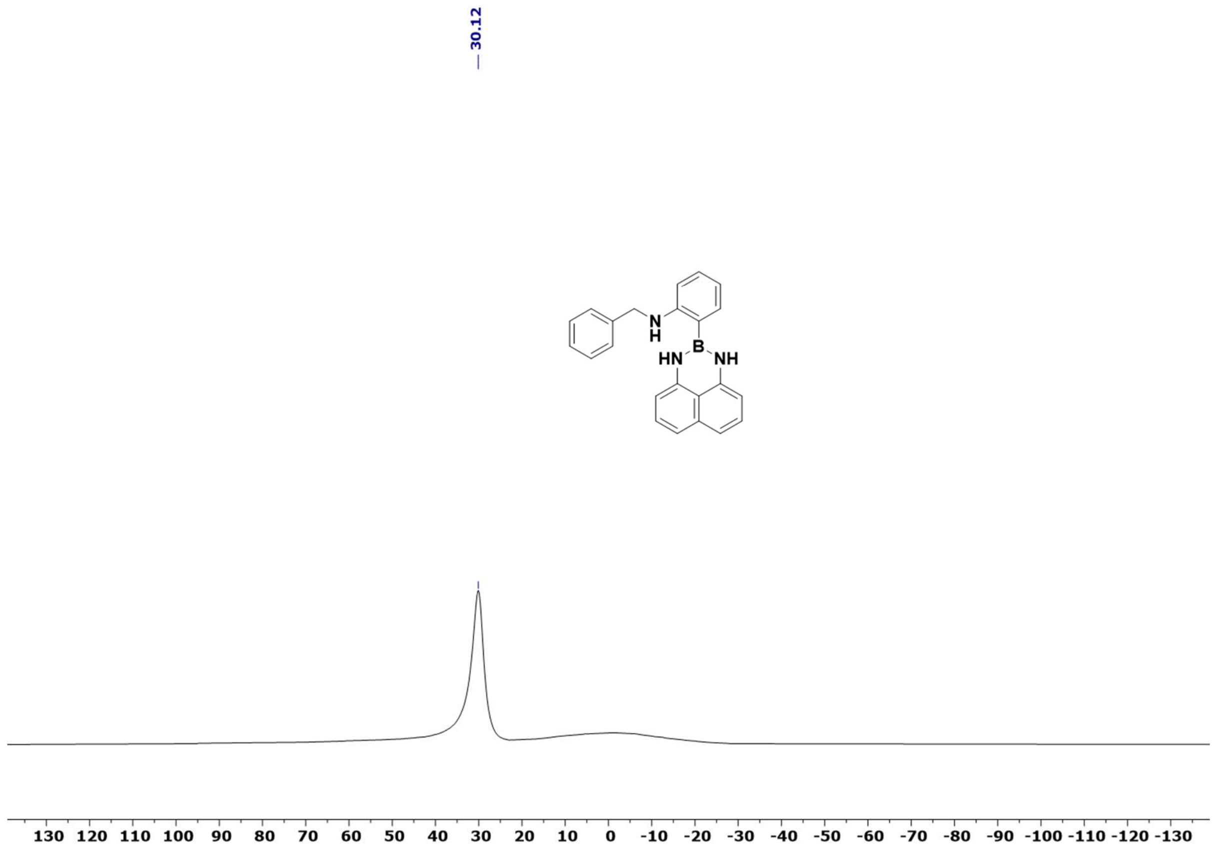

Figure S43. ^{11}B NMR spectrum of **3c** in $CDCl_3$.

Attempted reduction of Compound 14 using literature conditions [5]

To a dried NMR tube fitted with a J. Young valve was added zinc acetate (10 mol%, 2 mg), dry THF (0.5 mL) and $(EtO)_3SiH$ (55 µL, 3 eq). Compound **14** (32 mg, 0.1 mmol, 1 eq) was then added and the tube rotated at room temperature overnight. NMR spectra taken showed no reaction progress, so the reaction mixture was heated at 40 °C for 23 h. After that solvent was evaporated to get crude NMR in $CDCl_3$ which showed a 56 % formation of "deprotected" product (**15**)[6] with 44 % of unreacted starting material (**14**). Attempts to drive this reaction to completion using a larger excess of silane failed as this did not lead to formation of only **15** but led to multiple species, as evidenced by the ^{1}H NMR spectra.

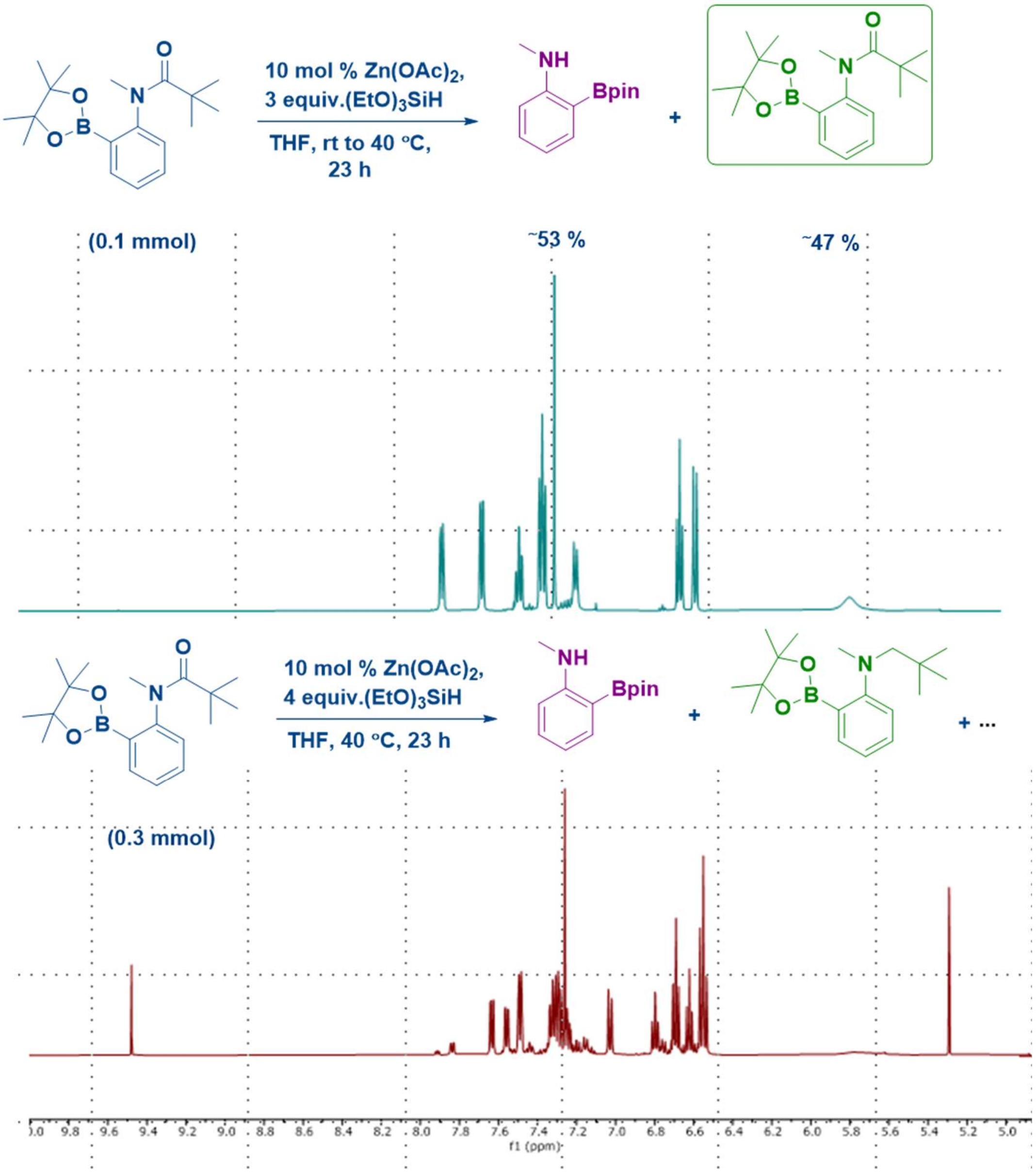

Figure S44. ^{1}H NMR spectra of the attempted zinc catalysed reduction of **14**, top with 3 equiv. hydrosilane, bottom 4 equiv. hydrosilane.

For comparison 12-Pin made via our methodology.

12-Pin can be made in good in-situ conversion (vs CH_2Br_2 added as an internal standard), however, it cannot be isolated cleanly from the silane by-products, while it proved sensitive to column

chromatography. For reference the crude ^{1}H NMR spectrum of **12-Pin** is provided below to confirm that the zinc catalysed approach does afford some of this product.

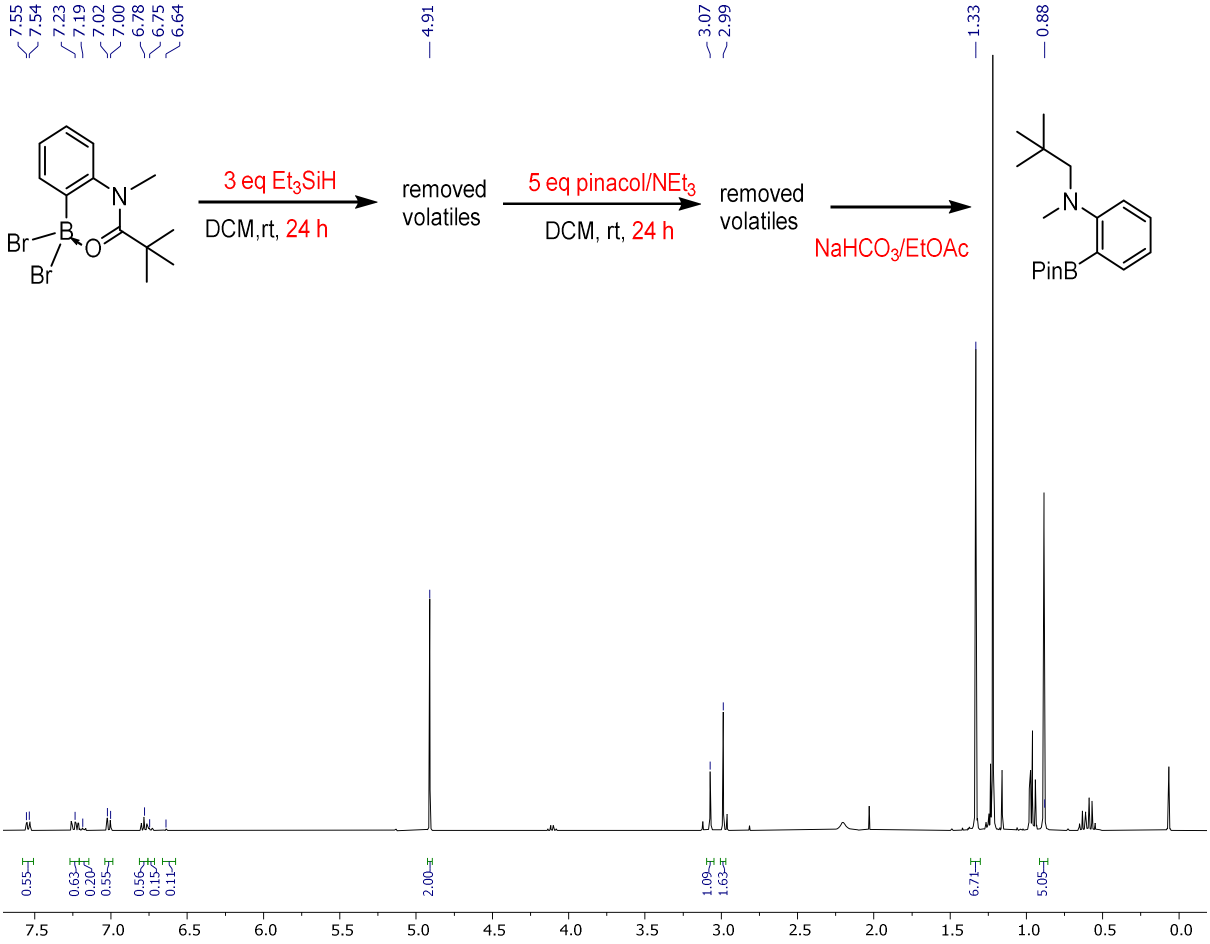

Figure S45. ^{1}H NMR spectrum of crude **12-Pin**.

5 Borylation of Carbazole Compounds:

Compound 16

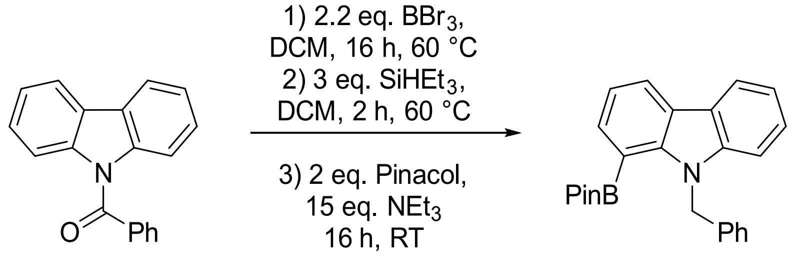

S2 **16**

Amide **S2** (0.054 g, 0.2 mmol) was added to an NMR tube fitted with a J. Young valve. The tube was evacuated and refilled with N_2. The solid was dissolved in DCM (1 mL) and BBr_3 1M in DCM (0.44 mmol, 0.44 mL) was added. The tube was sealed and heated at 60 °C for 16 h. The solvents/volatiles were removed under vacuum and the intermediate was dried. The solid was redissolved in DCM (1 mL) and $SiHEt_3$ (0.6 mmol, 96 μL) was added, the reaction was heated at 60 °C for 2 hours before all solvents/volatiles were removed under vacuum. DCM (1 mL) was added followed by pinacol (0.4 mmol, 0.05 g) and NEt_3 (3 mmol, 0.42 mL). The tube was sealed and stirred at room temperature for 16 hours. The crude product was then directly purified by flash chromatography on silica gel (DCM/PET) to give the pure product (0.023 g, 61%).

^{1}H NMR (500 MHz, $CDCl_3$) δ 8.27 (dd, *J* = 7.7, 1.4 Hz, 1H), 8.12 (dt, *J* = 7.7, 1.1 Hz, 1H), 7.95 (dd, *J* = 7.2, 1.4 Hz, 1H), 7.37 (m, 1H), 7.31 – 7.25 (m, 2H), 7.25 – 7.13 (m, 4H), 7.02 (m, 2H), 6.02 (s, 2H), 1.20 (s, 12H). **^{11}B NMR** (160 MHz, $CDCl_3$) δ 31.5. **^{13}C{^{1}H} NMR** (126 MHz, $CDCl_3$) δ 144.6, 141.4, 138.6, 135.0, 128.5, 126.8, 126.1, 125.9, 123.5, 123.4, 123.3, 119.9, 119.5, 118.7, 109.7, 84.1, 48.1, 24.7. **[Acc. Mass]**. Calcd for $C_{25}H_{26}BNO_2Na$ = 406.1949, found = 406.1942.

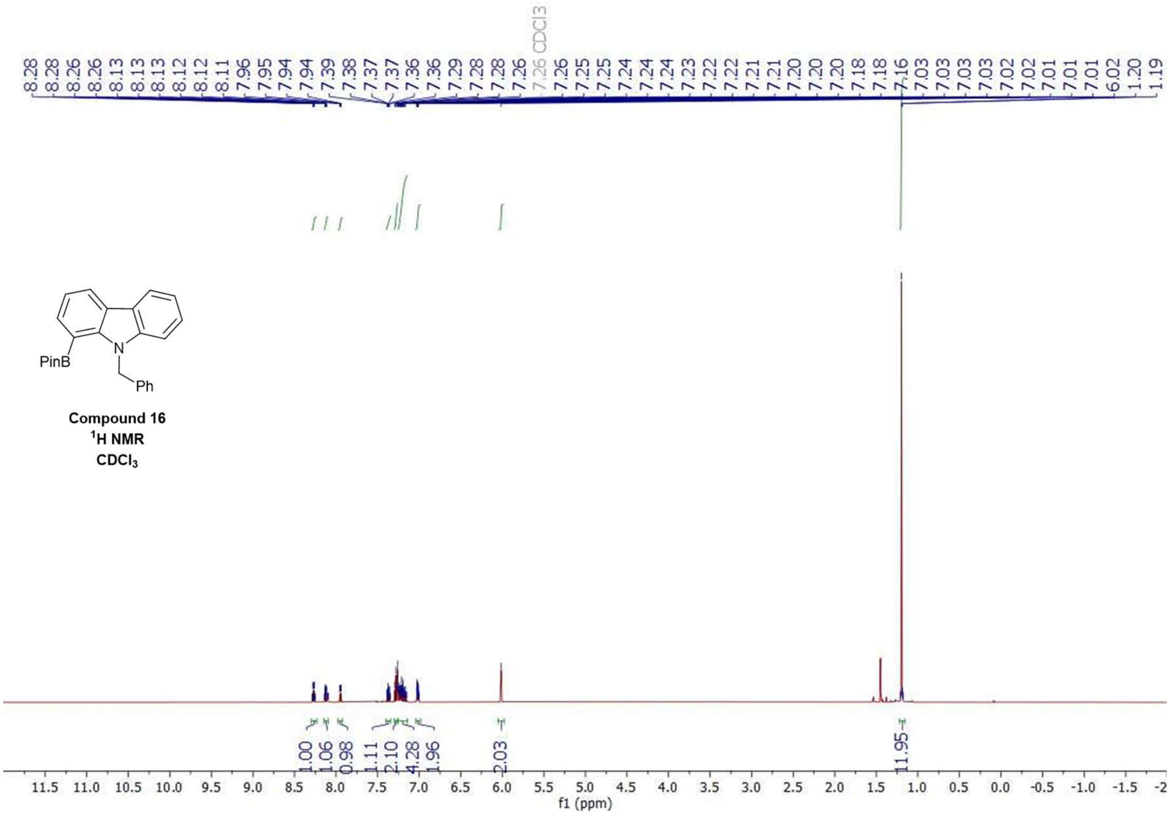

Figure S46. ^{1}H NMR spectrum of compound **16** in $CDCl_3$.

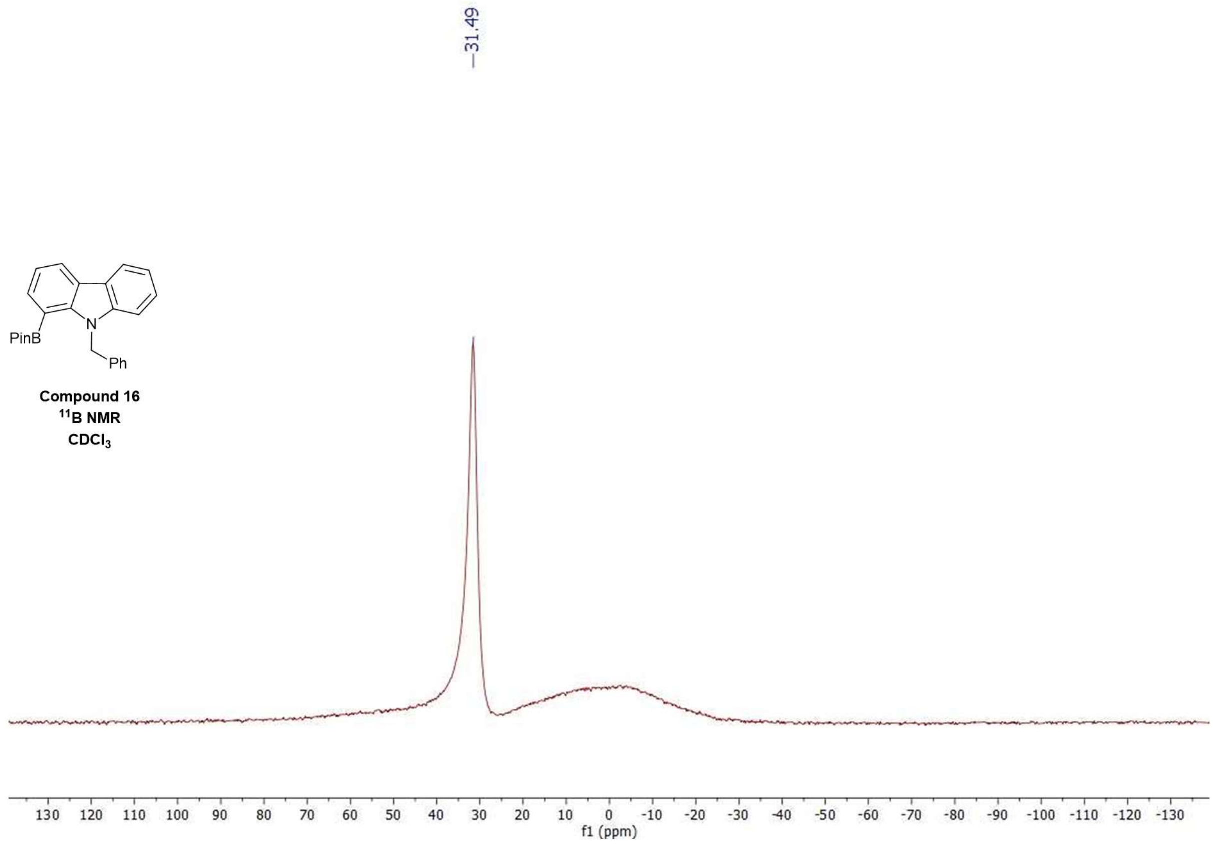

Figure S47. ^{11}B NMR spectrum of compound **16** in $CDCl_3$.

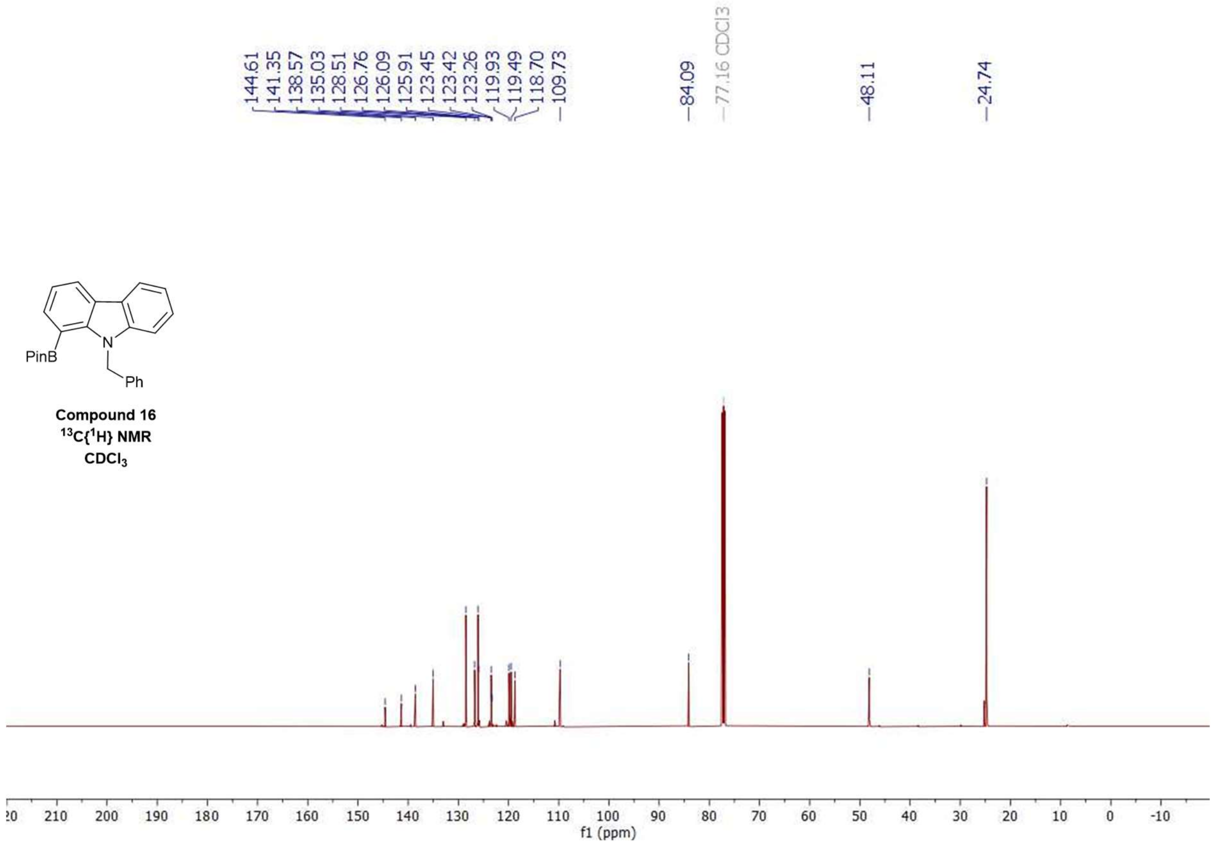

Figure S48. $^{13}C\{^{1}H\}$ NMR spectrum of compound **16** in $CDCl_3$.

1-(1-(4,4,5,5-tetramethyl-1,3,2-dioxaborolan-2-yl)-9H-carbazol-9-yl)octan-1-one

Compound 18

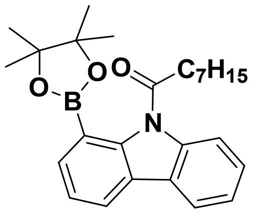

Compound **18** was prepared using **17** (147 mg, 0.5 mmol, 1 eq) dissolved in DCM (1 mL). To the stirred solution was added BBr_3 (1M solution in DCM) (0.6 mL, 0.6 mmol, 1.2 eq) and heated at 60 °C (in a sealed ampoule) for 16 h. A biphasic solution of pinacol (71 mg, 0.5 mmol, 1.1 eq in 0.5 mL DCM) and K_2CO_3 (249 mg, 1.8 mmol, 3.5 eq in 1 mL H_2O) was added and stirred at room temperature overnight. $MgSO_4$ was added to the reaction mixture diluted with DCM and left for an hour. The mixture was filtered, evaporated and dried under vacuum at 60 °C to remove excess pinacol to get the pure product (196 mg, 93%). **[Acc. Mass]** Calculated for $[M+H]^+$: 420.2705, found $[M+H]^+$ 420.2718.

^{1}H NMR (500 MHz, $CDCl_3$, 298 K): δ 7.99 (dd, *J*=7.7, 1.4 Hz, 1H), 7.89 (dd, *J*=7.6, 1.3 Hz, 1H), 7.71 (d, *J*=8.3 Hz, 1H), 7.63 (dd, *J*=7.3, 1.3 Hz, 1H), 7.48-7.38 (m, 3H), 3.17 (t, *J*=7.3 Hz, 2H), 1.94 (p, *J*=7.4 Hz, 2H), 1.55-1.48 (m, 2H), 1.45 (s, 12H), 1.43-1.37 (m, 2H), 1.35-1.30 (m, 4H), 0.93-0.88 (m, 3H). **^{13}C{^{1}H}** NMR (126 MHz, $CDCl_3$, 298 K): δ 173.7, 140.8, 137.0, 132.2, 128.6, 125.4, 124.6, 124.5, 121.0, 119.6, 115.6, 82.6, 37.7, 31.8, 29.2, 25.8, 24.3, 22.8, 14.2. **^{11}B NMR** (160 MHz, CD_2Cl_2, 298 K): δ 21.8.

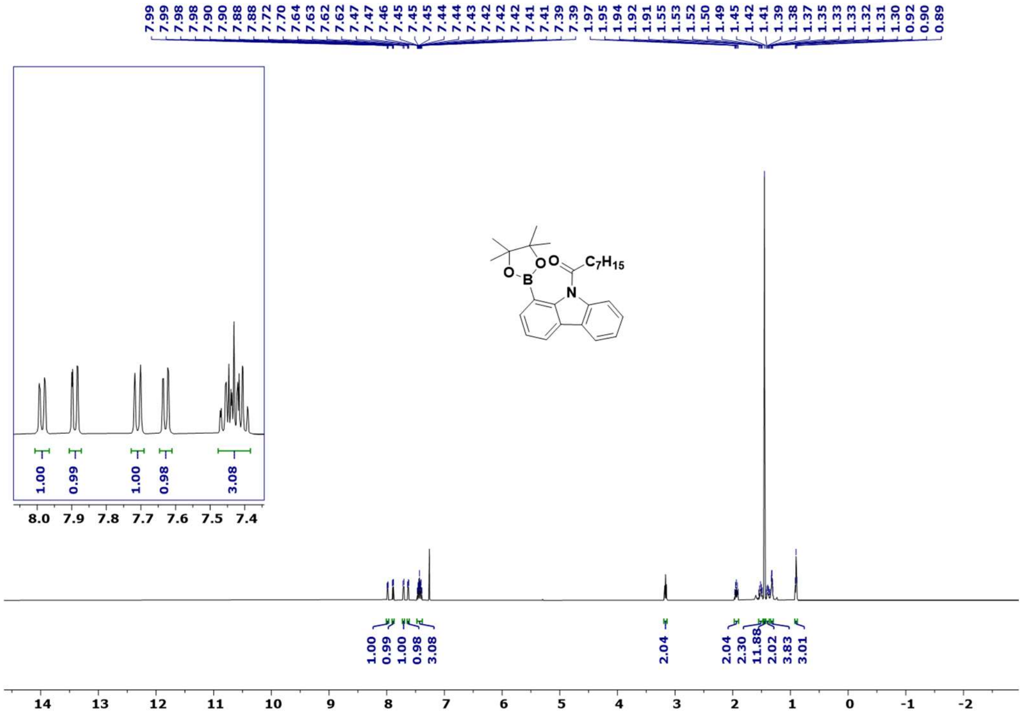

Figure S49. ^{1}H NMR spectrum of **18** in $CDCl_3$.

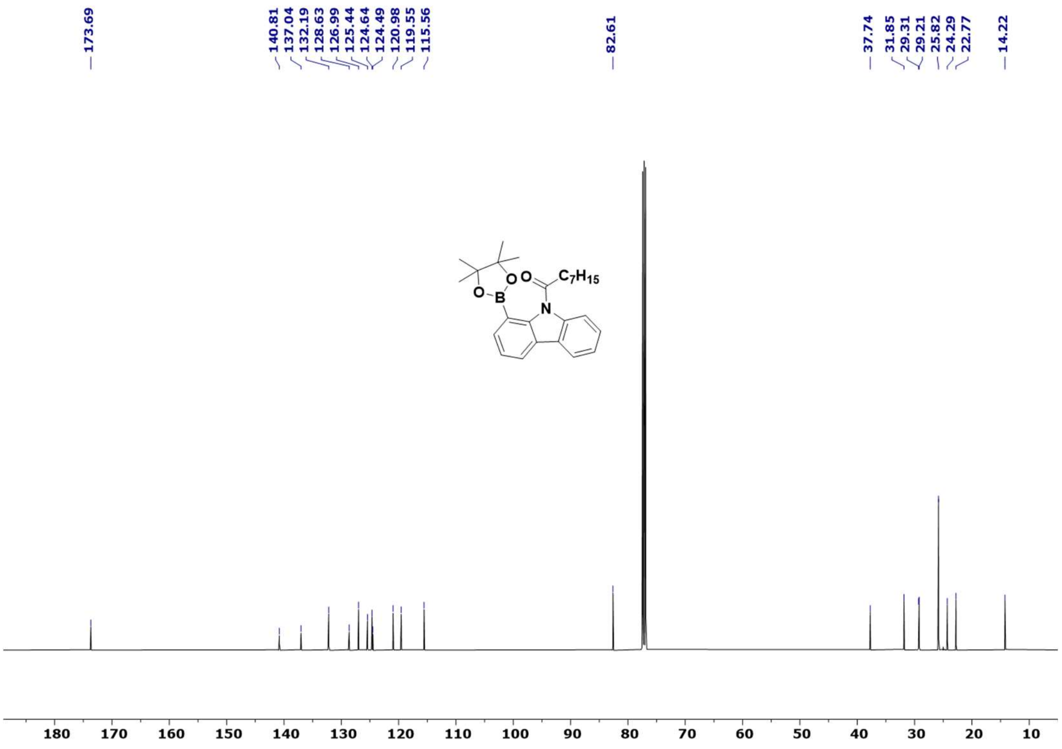

Figure S50. $^{13}C\{^{1}H\}$ NMR spectrum of **18** in $CDCl_3$.

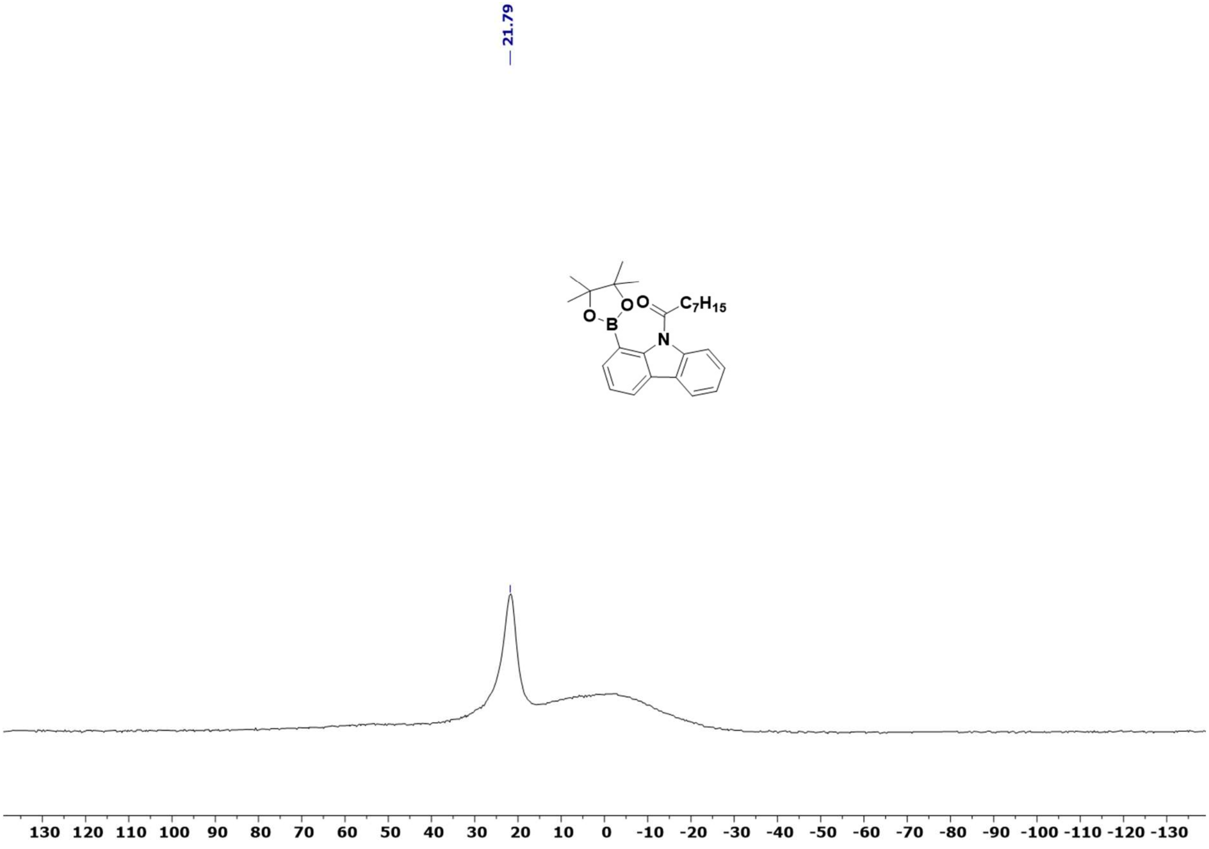

Figure S51. ^{11}B NMR spectrum **18** in $CDCl_3$.

9-octyl-1-(4,4,5,5-tetramethyl-1,3,2-dioxaborolan-2-yl)-9H-carbazole

Compound 19

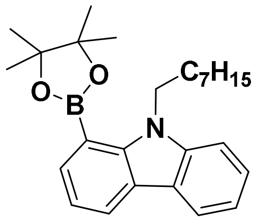

Compound **19** was prepared using **17** (110 mg, 0.37 mmol, 1 eq) dissolved in DCM (0.5 mL). To the stirred solution was added BBr_3 (1M solution in DCM, 0.44 mL, 0.44 mmol, 1.2 eq) and heated at 60 °C (in a sealed ampoule) for 18 h. The solution obtained was evaporated and dried under vacuum to get a dry solid. Dry DCM (1 mL) and Et_3SiH (0.12 mL, 0.74 mmol, 2.0 eq) were added and stirred at room temperature overnight. The clear yellow solution obtained was evaporated and dried under vacuum. To the residue the biphasic solution of pinacol (52 mg, 0.44 mmol, 1.2 eq in 0.5 mL DCM) and K_2CO_3 (179 mg, 1.3 mmol, 3.5 eq in 1 mL H_2O) was added and stirred at room temperature overnight. $MgSO_4$ was added to the reaction mixture diluted with DCM and left for an hour. The mixture was filtered, and volatiles removed under vacuum to get crude product which was purified by cold hexane washing (hexane dissolved the desired product but left silicon containing impurities behind) to get the product as a white solid (80 mg, 53%). %). **[Acc. Mass]** Calculated for $[M+H]^+$: 406.2912, found $[M+H]^+$ 406.2903.

^{1}**H NMR** (500 MHz, $CDCl_3$, 298 K): δ 8.24 (dd, *J*=7.6, 1.4 Hz, 1H), 8.12 (dt, *J*=7.7, 1.0 Hz, 1H), 7.97 (dd, *J*=7.3, 1.4 Hz, 1H), 7.50-7.43 (m, 2H), 7.23-7.22 (m, 2H), 4.74-4.67 (m, 2H), 1.87-1.79 (m, 2H), 1.47 (s, 12H), 1.41-1.21 (m, 12H)[a], 0.91-0.88 (m, 3H). ^{13}**C{^{1}H} NMR** (126 MHz, $CDCl_3$, 298 K): δ 143.6, 141.1, 134.9, 125.6, 123.4, 123.3, 123.1, 120.0, 119.0, 118.2, 109.3, 84.1, 44.3, 32.0, 29.8, 29.4, 27.3, 25.1, 22.8, 14.2. ^{11}**B NMR** (160 MHz, CD_2Cl_2, 298 K): δ 31.8.

(a) slightly increased integration value corresponds to minor pinacol impurity overlapping with this resonance.

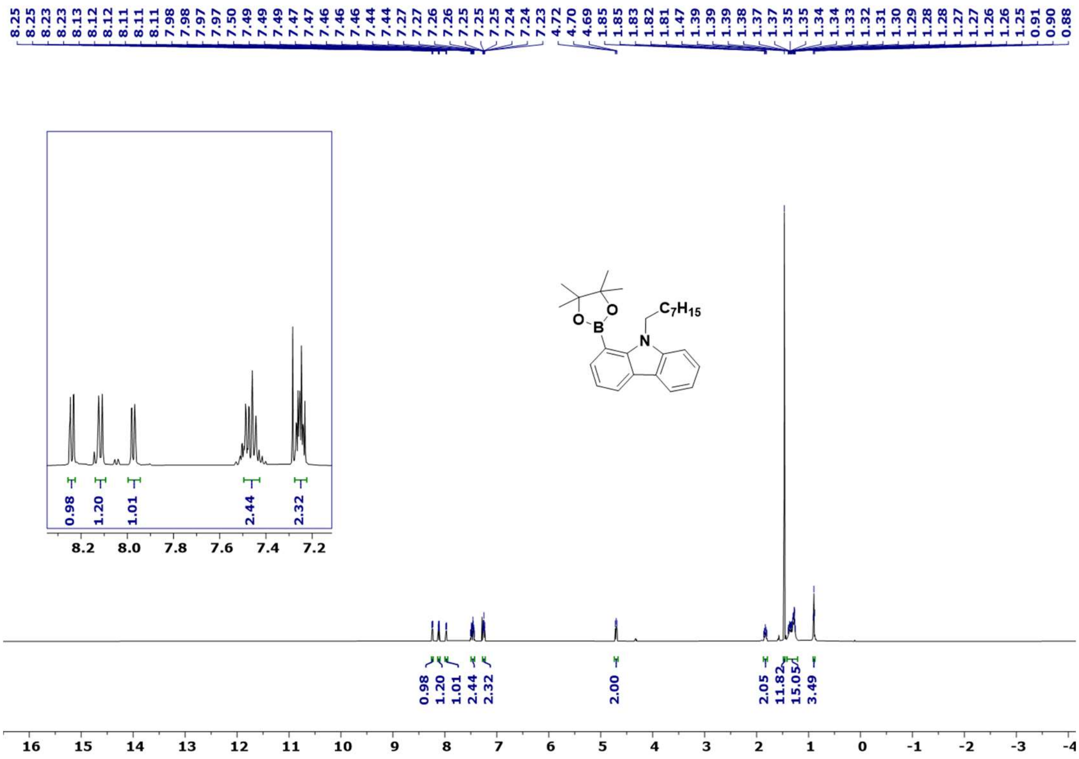

Figure S52. ^{1}H NMR spectrum of **19** in $CDCl_3$.

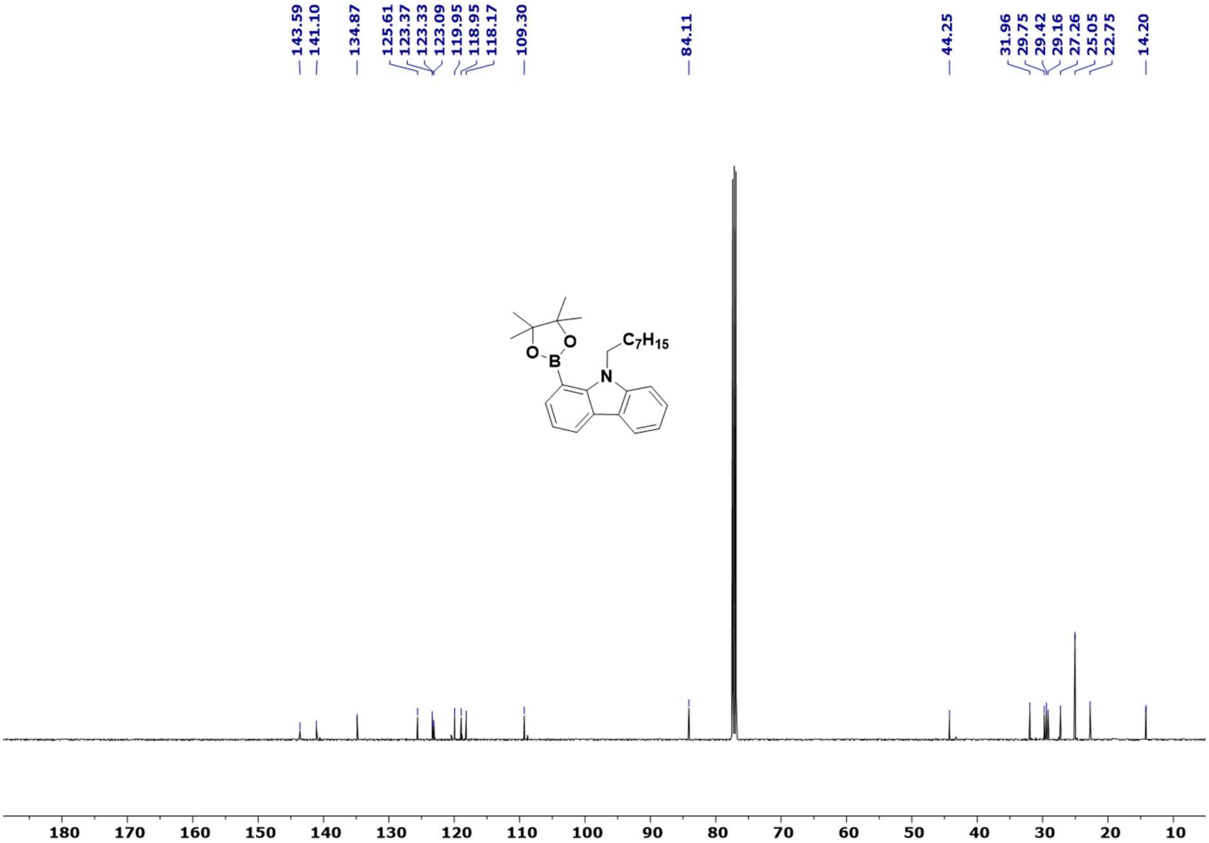

Figure S53. $^{13}C\{^1H\}$ NMR spectrum of **19** in $CDCl_3$.

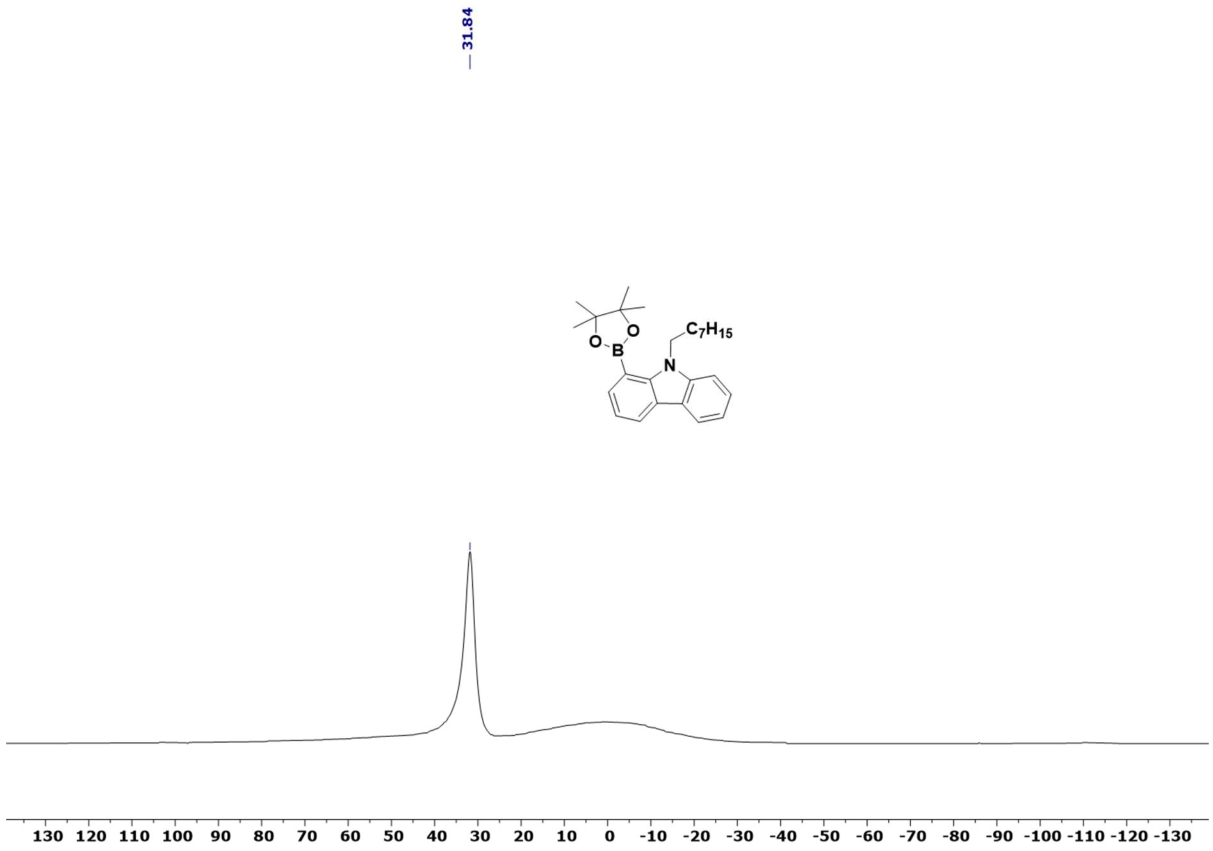

Figure S54. ^{11}B NMR spectrum of **19** in $CDCl_3$.

6 Double Borylation – reduction studies

1,1,7,7-tetrabromo-3,9-di-tert-butyl-1,4,7,10-tetrahydro-1l4,2l3,7l4,8l3-naphtho[1,8-cd:5,4-c'd']bis([1,6,2]oxazaborepine)

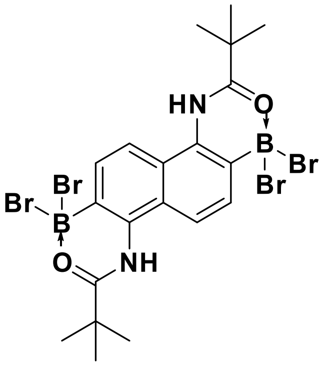

An oven-dried ampoule was charged with **21** (1.63 g, 5 mmol, 1 eq), followed by addition of dry DCM (15 mL) and BBr_3 (1M solution in heptane, 15 mL, 15 mmol, 3 eq). Sealed ampoule was a heated at 60 °C for 65h. Obtained white suspension was then evaporated under vacuum to get a dry white solid (3.1 g, 93%). Because of the very low solubility in appropriate organic solvents (e.g. halocarbons), we could not perform representative spectral analysis.

N,N'-(4,8-bis(4,4,5,5-tetramethyl-1,3,2-dioxaborolan-2-yl)naphthalene-1,5-diyl)bis(2,2-dimethylpropanamide)

Compound 23

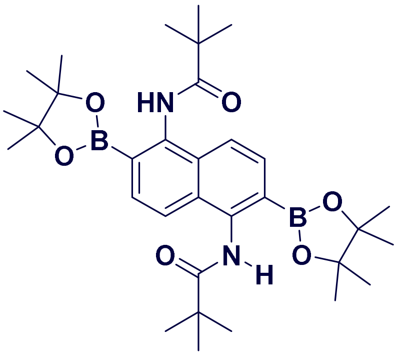

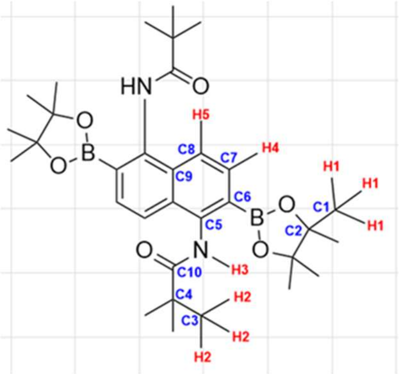

An oven-dried ampoule was charged with the solid from the above reaction (333 mg, 0.5 mmol, 1 eq) followed by the addition of dry DCM (1 mL). The biphasic solution of pinacol (130 mg, 1.1 mmol, 2.2 eq in 0.5 mL DCM) and K_2CO_3 (484 mg, 3.5 mmol, 7 eq in 1 mL H_2O) was then added and stirred at room temperature overnight. $MgSO_4$ was added to the reaction mixture diluted with DCM and left for an hour. The reaction mixture was then filtered, evaporated, and dried under vacuum at 60 °C to remove excess pinacol to get the pure product (240 mg, 83%).

[Acc. Mass] Calculated for $[M+H]^+$: 579.3771, found $[M+H]^+$ 579.3796. **^{1}H NMR** (500 MHz, $CDCl_3$, 298 K): δ 8.85 (br s, 2H, H3), 7.83 (d, *J*=8.4 Hz, 2H, H4), 7.53 (d, *J*=8.4 Hz, 2H, H5), 1.47 (s, 18H, H2), 1.37 (s, 24H, H1). **^{13}C{^{1}H} NMR** (126 MHz, $CDCl_3$, 298 K): δ 178.1 (C10), 134.0 (C9), 130.8 (C7), 125.7 (C6),

125.0 (C5), 117.6 (C8), 82.0 (C2), 39.4 (C4), 27.3 (C3), 25.0 (C1). **^{11}B NMR** (160 MHz, CD_2Cl_2, 298 K): δ 17.3.

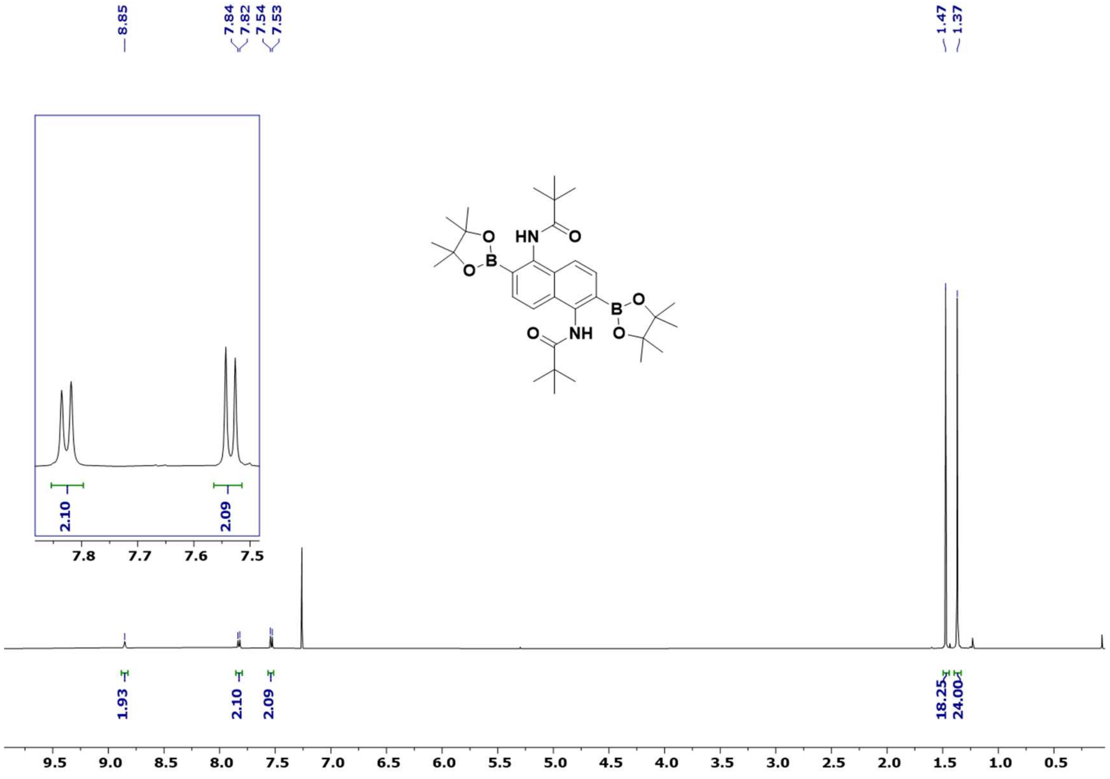

Figure S55. ^{1}H NMR spectrum of **23** in $CDCl_3$.

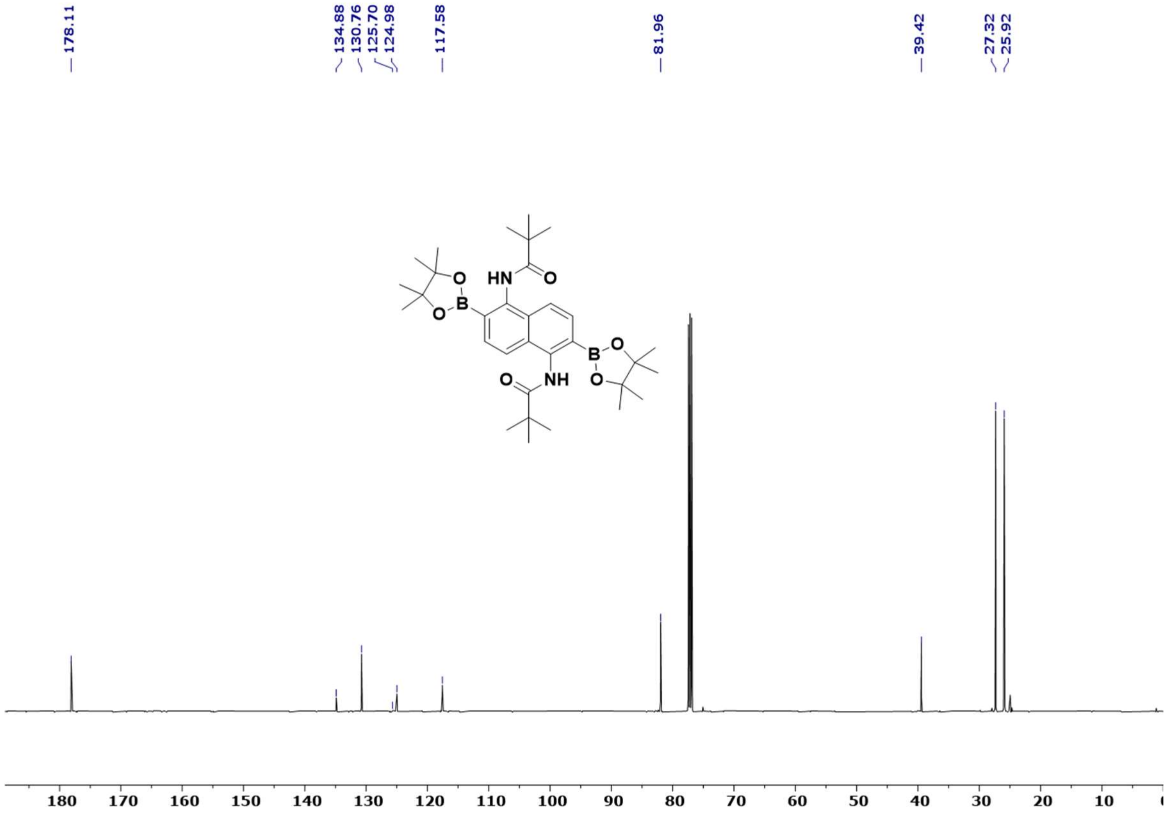

Figure S56. $^{13}C\{^1H\}$ NMR spectrum of **23** in $CDCl_3$.

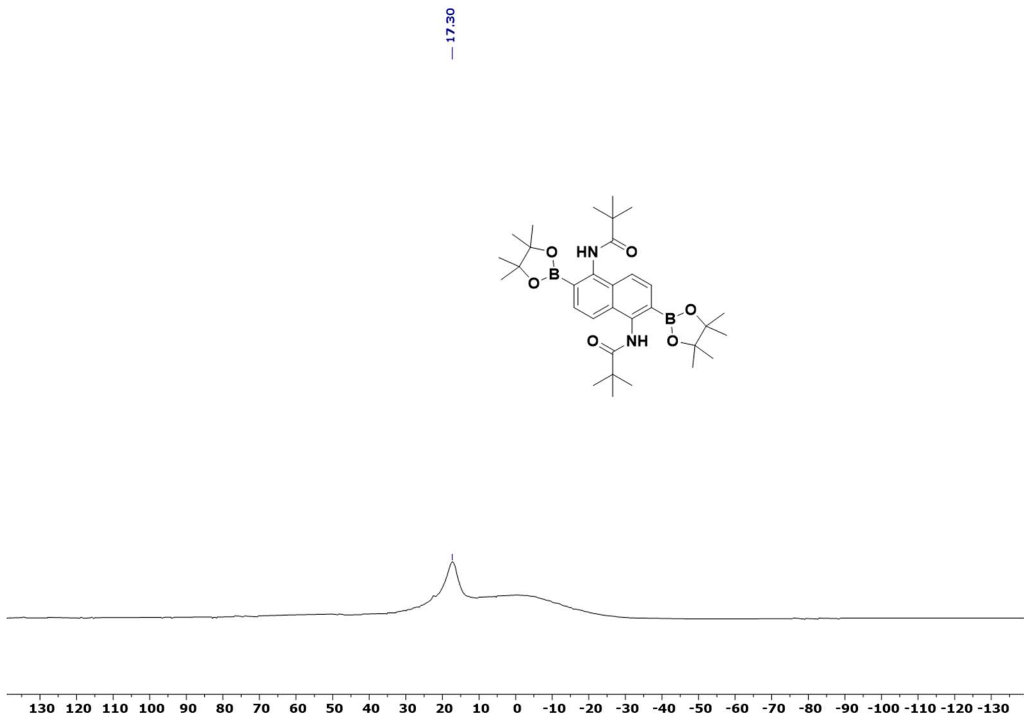

Figure S57.1 ^{11}B NMR spectrum of **23** in $CDCl_3$.

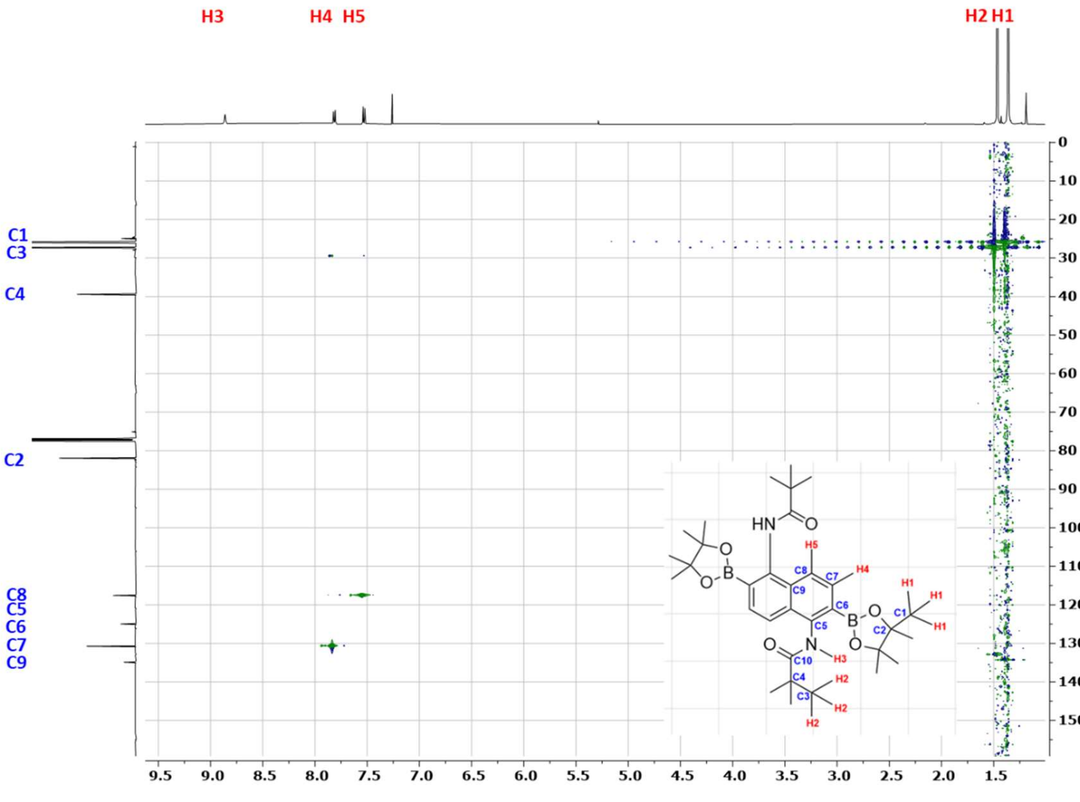

Figure S58. ^{1}H – ^{13}C{^{1}H} HSQC NMR spectrum of **23** in $CDCl_3$.

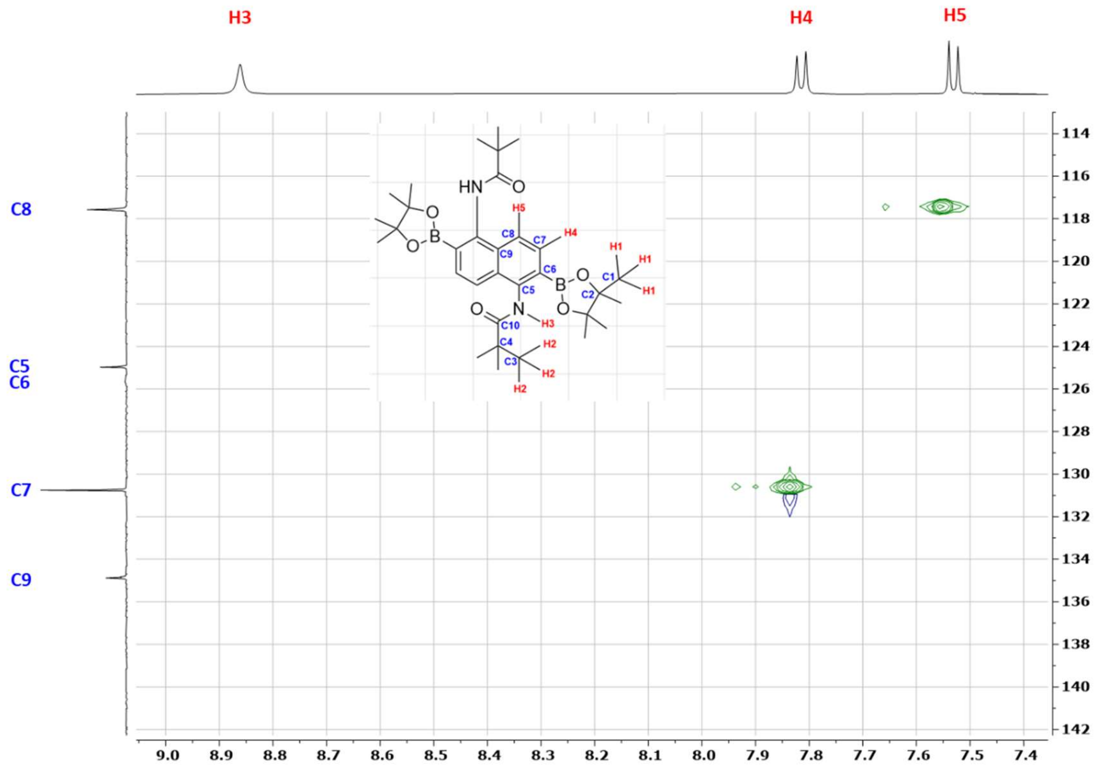

Figure S59. $^1H - ^{13}C\{^1H\}$ HSQC NMR spectrum of **23** in $CDCl_3$.

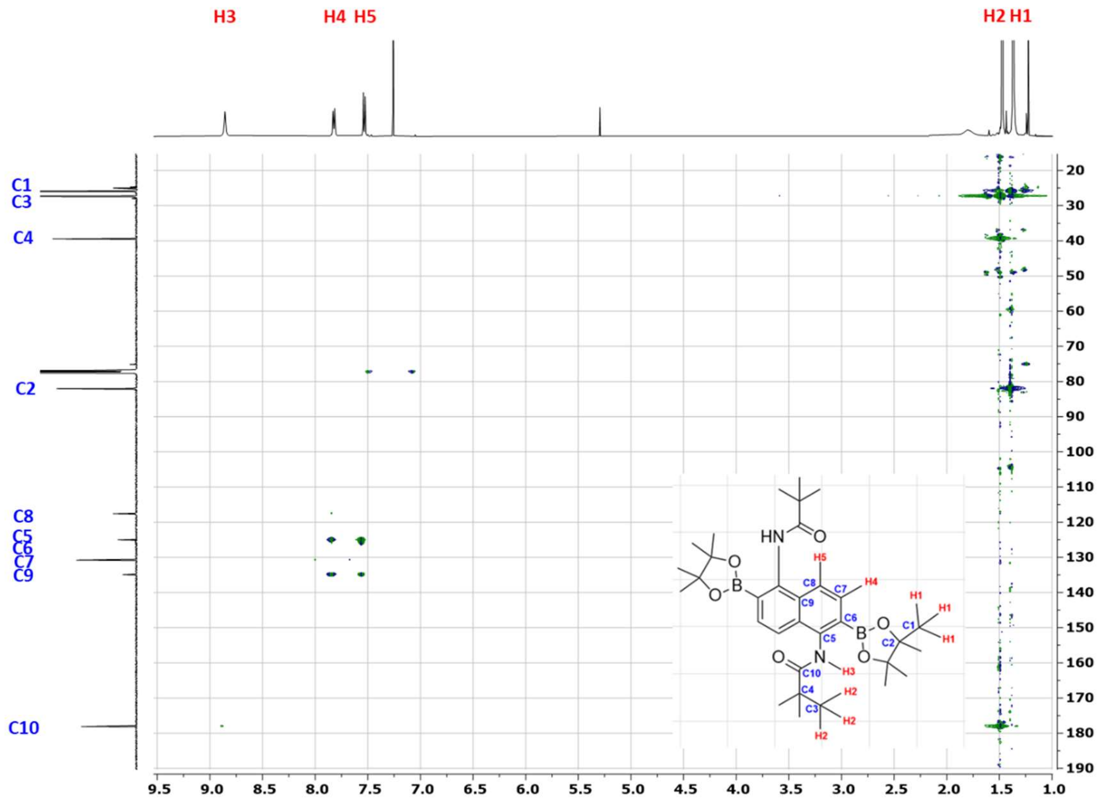

Figure S60. $^1H - ^{13}C\{^1H\}$ HMBC NMR spectrum of **23** in $CDCl_3$.

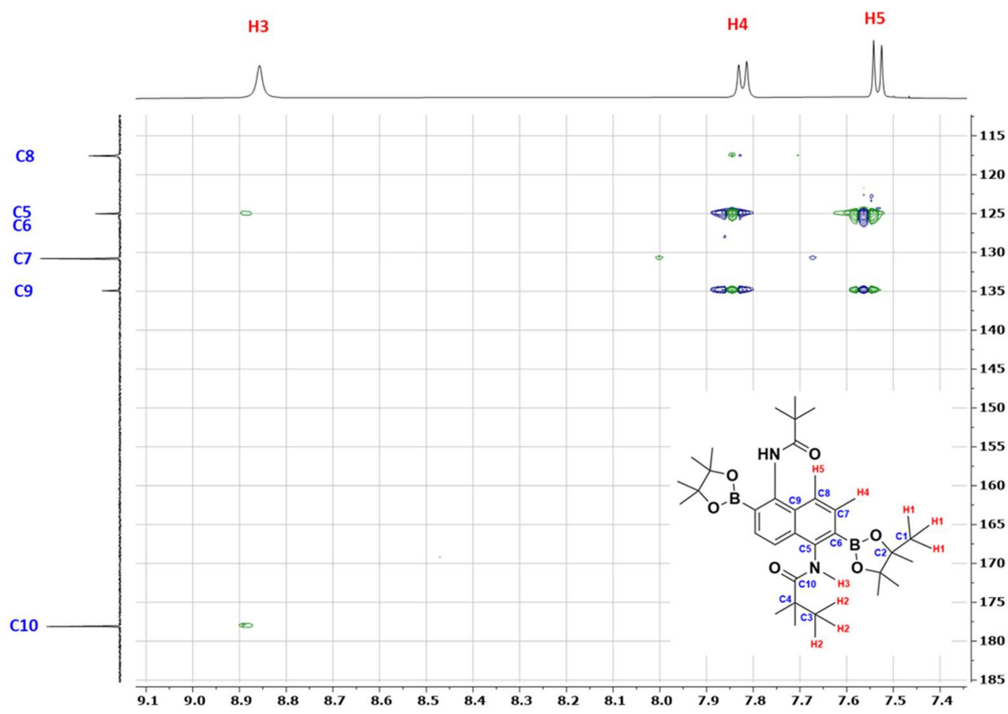

Figure S61. ^{1}H – $^{13}C\{^{1}H\}$ HMBC NMR spectrum of **23** in $CDCl_3$.

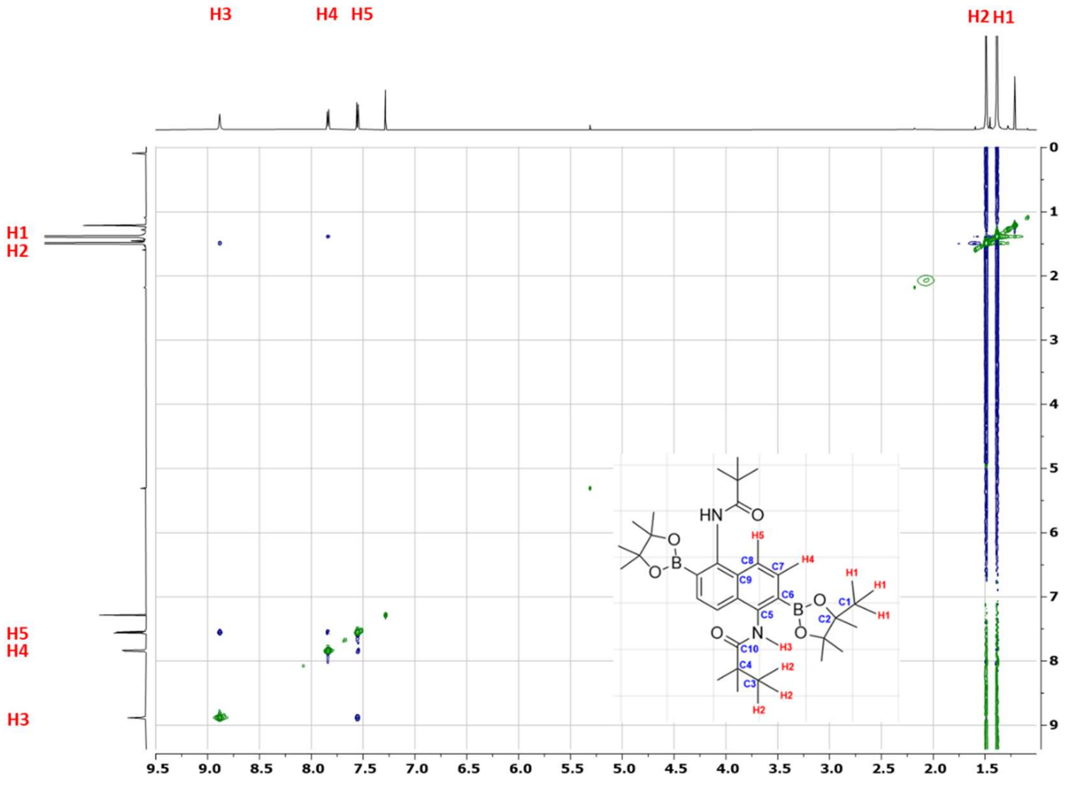

Figure S62. ^{1}H – $^{13}C\{1H\}$ NOESY NMR spectrum of **23** in $CDCl_3$.

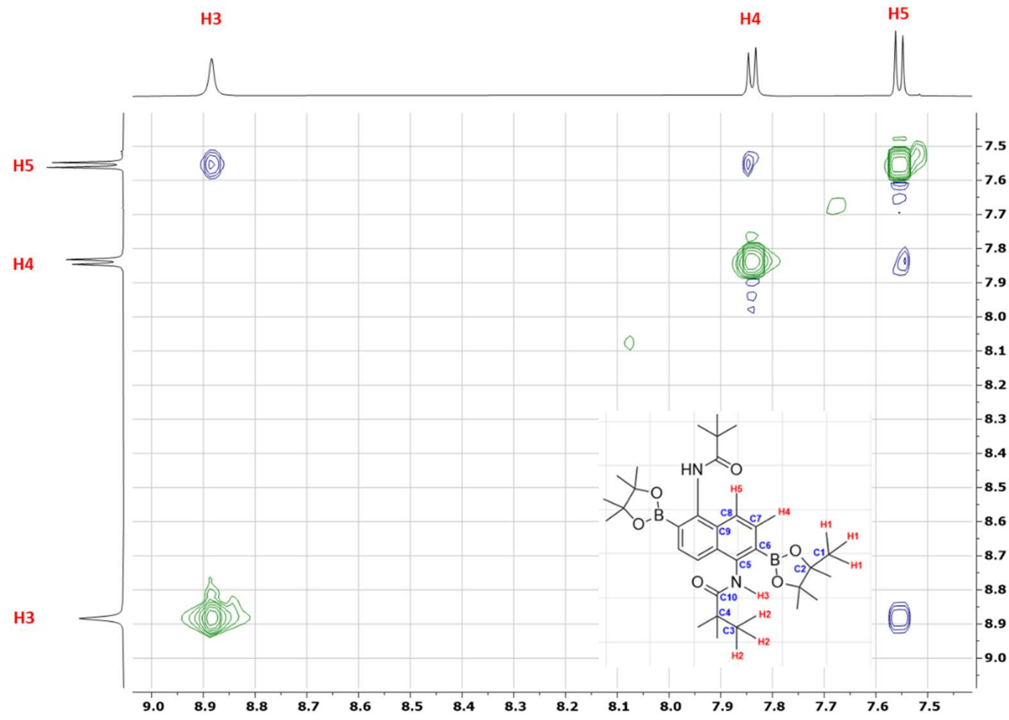

Figure S63. ^{1}H – $^{13}C\{^{1}H\}$ HMBC NMR spectrum of **23** in $CDCl_3$.

***N,N'*-(2,5-bis(4,4,5,5-tetramethyl-1,3,2-dioxaborolan-2-yl)-1,4-phenylene)bis(2,2-dimethylpropanamide)**

Compound 22

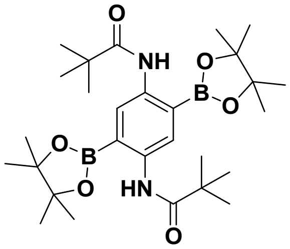

Compound **22** was prepared using the respective amide (138 mg, 0.5 mmol, 1 eq) dissolved in DCM (3 mL). To the stirred solution was added BBr_3 (1M solution in heptane, 1.5 mL, 1.5 mmol, 3 eq) and heated at 60 °C (in a sealed ampoule) for 65 h. The biphasic solution of pinacol (130 mg, 1.1 mmol, 2.2 eq in 0.5 mL DCM) and K_2CO_3 (484 mg, 3.5 mmol, 7 eq. in 1 mL H_2O) was added and stirred at room temperature overnight. $MgSO_4$ was added to the reaction mixture diluted with DCM and left for an hour. Filtered, evaporated, and dried under vacuum at 60 °C to remove excess pinacol to get the pure product (211 mg, 80%). **[Acc. Mass]** Calculated for $[M+H]^+$: 329.3615, found $[M+H]^+$ 329.3614.

^{1}H NMR (500 MHz, $CDCl_3$, 298 K): δ 9.43 (br s, 2H), 8.92 (s, 2H), 1.36 (s, 24H), 1.32 (s, 18H). ^{13}C{^{1}H} NMR (126 MHz, $CDCl_3$, 298 K): δ 177.0, 140.0, 126.4, 120.9[a] 84.6, 40.1, 27.8, 25.1. **^{11}B NMR** (160 MHz, CD_2Cl_2, 298 K): δ 30.4.

[a]120.9 (C-B) signal was too weak to be observed by ^{13}C{^{1}H} NMR, its assignment was possible by ^{1}H – ^{13}C{^{1}H} HMBC experiment.

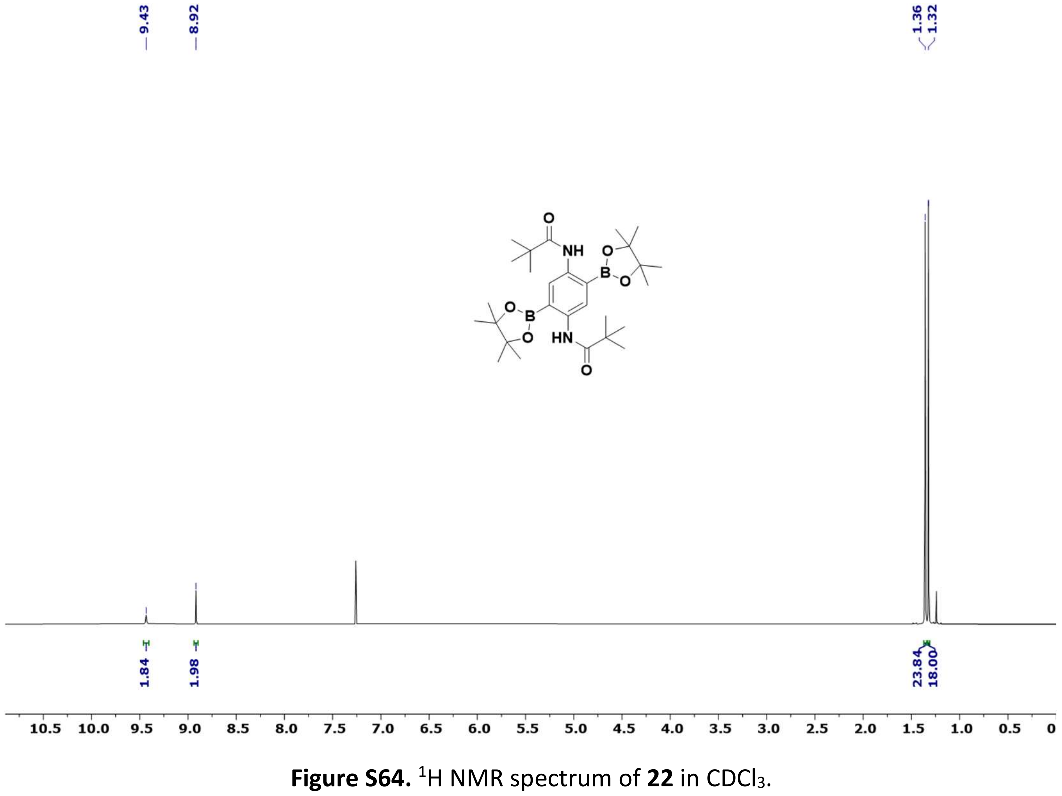

Figure S64. ^{1}H NMR spectrum of **22** in $CDCl_3$.

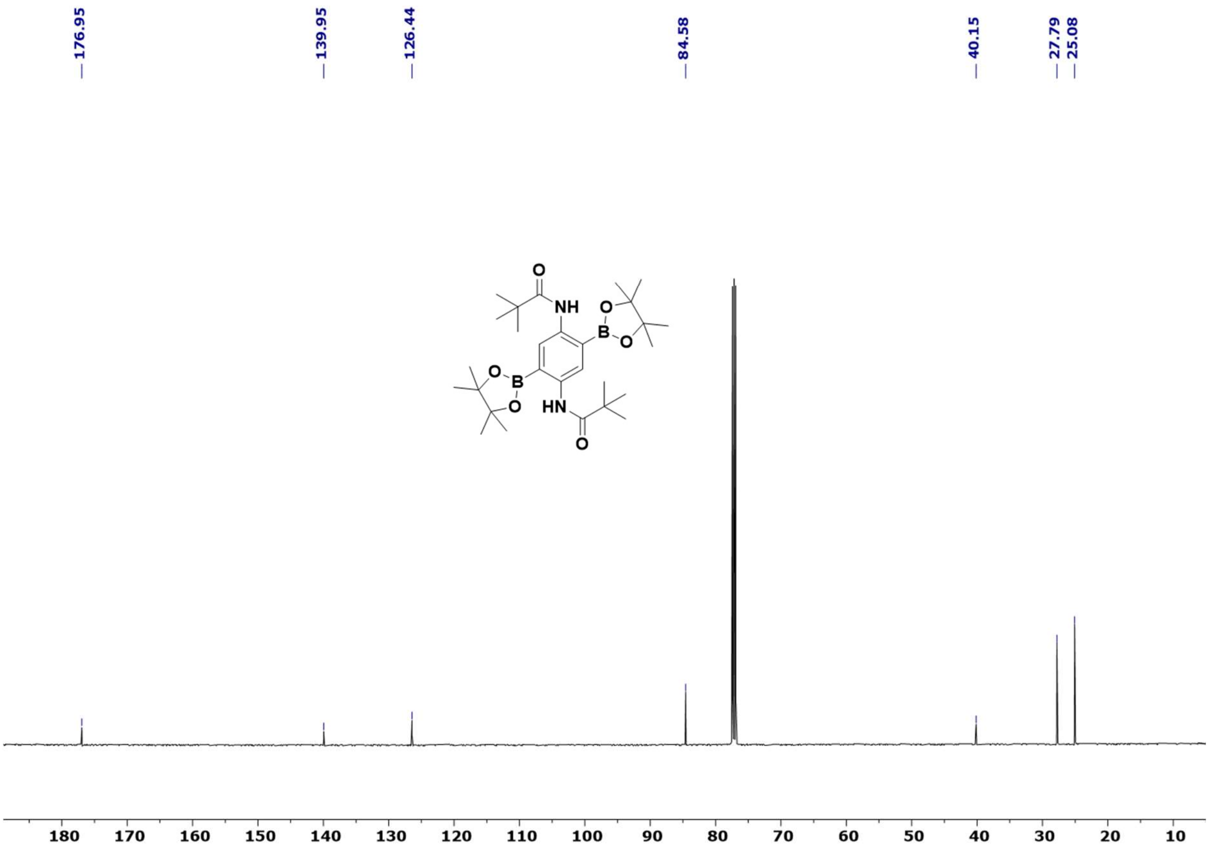

Figure S65. $^{13}C\{^{1}H\}$ NMR spectrum of **22** in $CDCl_3$.

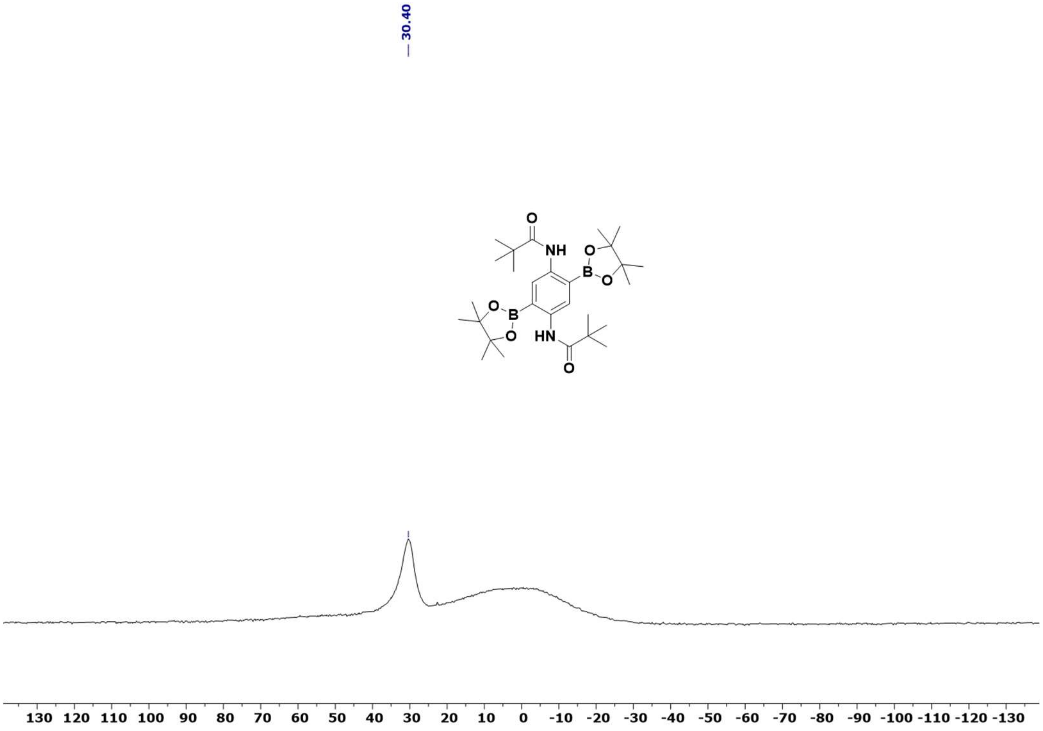

Figure S66. ^{11}B NMR spectrum of **22** in $CDCl_3$.

2,5-bis(1H-naphtho[1,8-de][1,3,2]diazaborinin-2(3H)-yl)-N1,N4-dineopentylbenzene-1,4-diamine

Compound 24

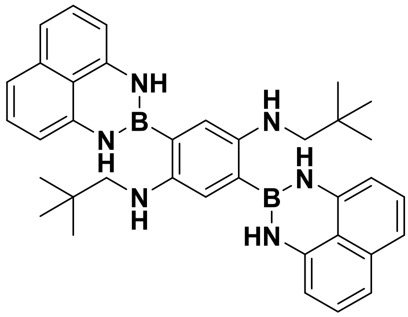

Compound **24** was prepared directly from the starting amide (138 mg, 0.5 mmol, 1 eq) dissolved in DCM (3 mL). To the stirred solution was added BBr_3 (1M solution in DCM) (2 mL, 2 mmol, 4 eq) and heated at 60 °C (in a sealed ampoule) for 65 h. To obtained white suspension was then added Et_3SiH (0.4 mL, 2.5 mmol, 5 eq) and stirred at room temperature overnight. The clear solution obtained was evaporated under vacuum to get a dry solid. The biphasic solution of 1,8-diaminonapthalene (348 mg, 1.1 mmol, 2.2 eq in 0.5 mL DCM) and K_2CO_3 (484 mg, 3.5 mmol, 7 eq in 1 mL H_2O) was added and stirred at room temperature overnight. $MgSO_4$ was added to the reaction mixture diluted with DCM and left for an hour. The reaction mixture was filtered, and volatiles removed under vacuum to get crude product which was purified by column chromatography on silica gel (petroleum ether/ DCM = 8:2) to get maroon solid (70 mg, 24%). **[Acc. Mass]** Calculated for $[M+H]^+$: 580.3652, found $[M+H]^+$ 580.3668.

^{1}H NMR (500 MHz, $CDCl_3$, 298 K): δ 7.15 (t, *J*=7.7 Hz, 4H), 7.07 (d, *J*=8.2 Hz, 4H), 6.82 (br s, 2H), 6.40 (d, *J*=7.3 Hz, 4H), 6.38 (br s, 4H), 3.51 (br s, 2H), 2.89 (br s, 4H), 1.06 (s, 18 H). **^{13}C{^{1}H} NMR** (126 MHz, $CDCl_3$, 298 K): δ 141.1, 136.5, 127.8, 126.4, 120.1, 118.0, 111.7, 106.2, 53.6, 31.7, 28.1. **^{11}B NMR** (160 MHz, CD_2Cl_2, 298 K): δ 30.4.

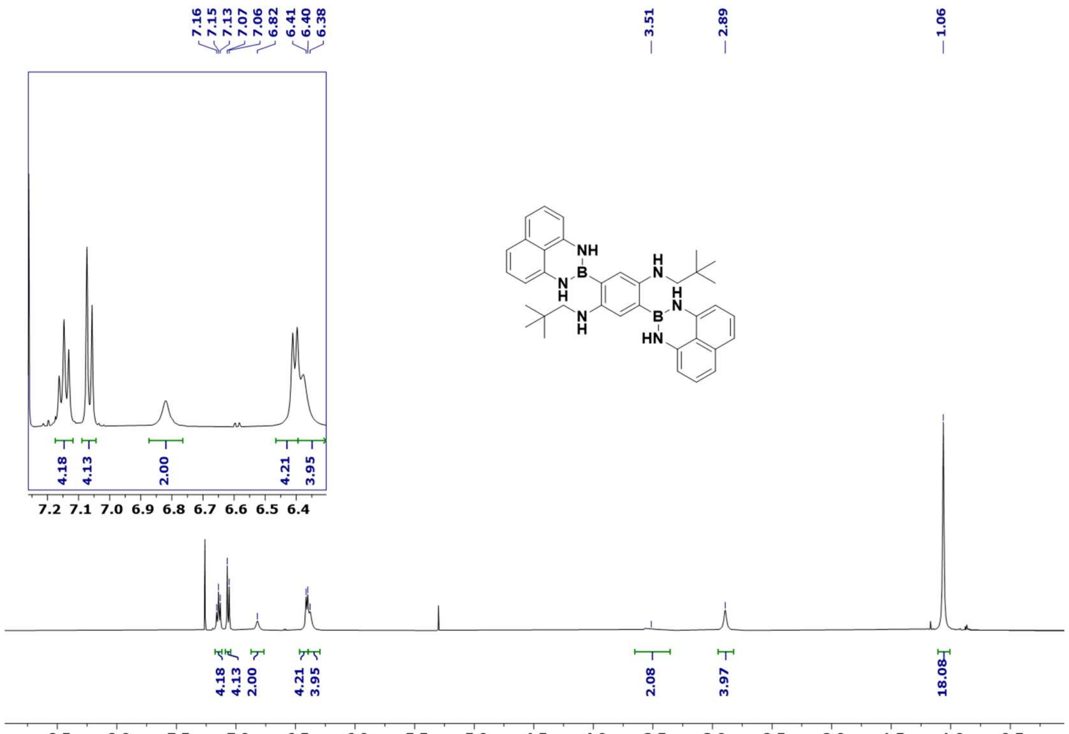

Figure S67. ^{1}H NMR spectrum of **24** in $CDCl_3$.

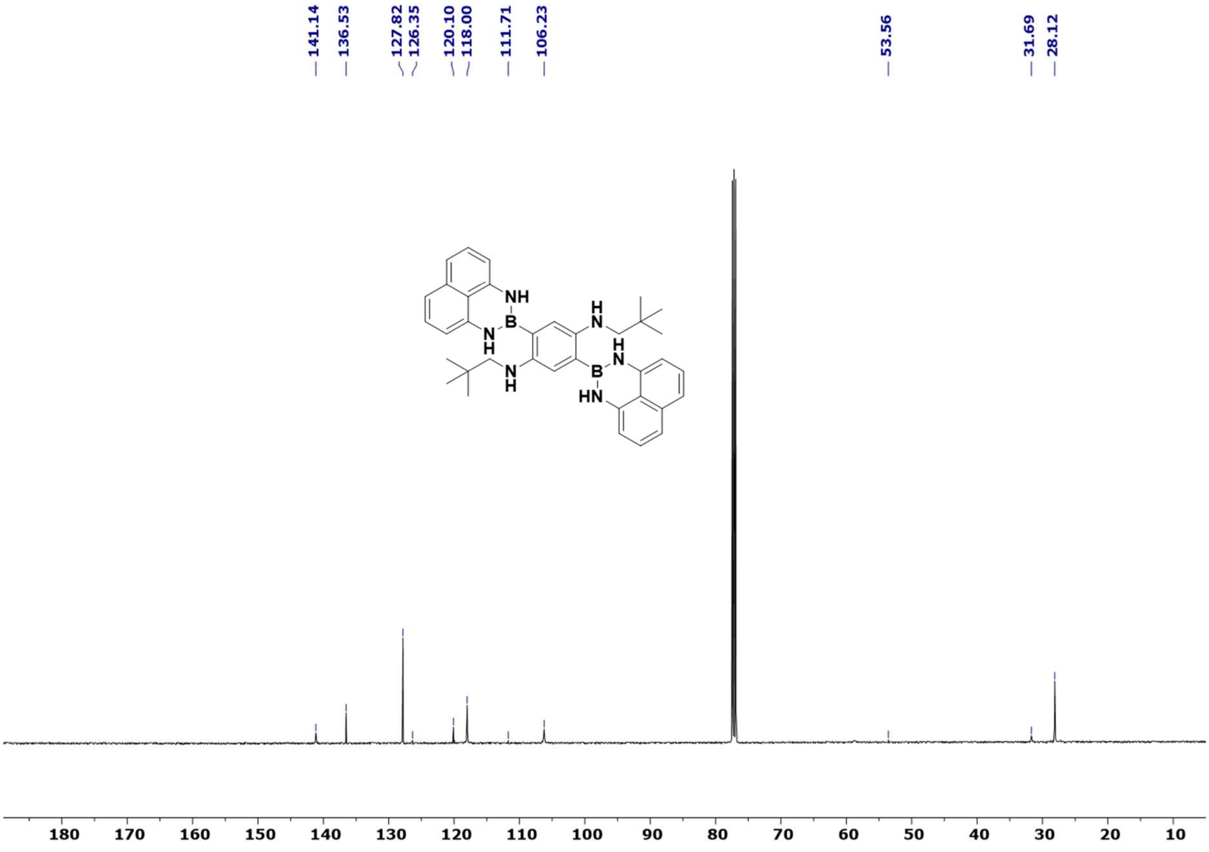

Figure S68. $^{13}C\{^1H\}$ NMR spectrum of **24** in $CDCl_3$.

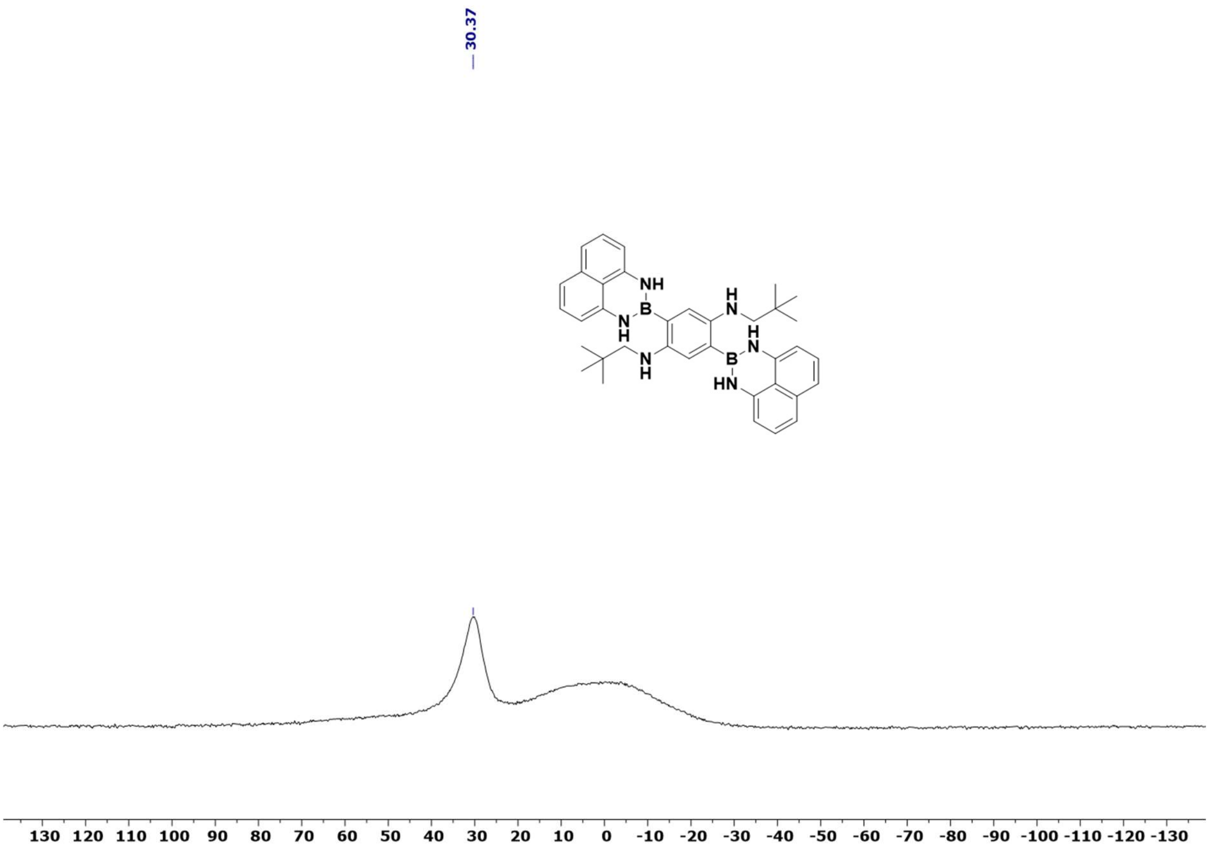

Figure S69. ^{11}B NMR spectrum of **24** in $CDCl_3$.

N1,N5-dineopentyl-4,8-bis(4,4,5,5-tetramethyl-1,3,2-dioxaborolan-2-yl)naphthalene-1,5-diamine

Compound 25

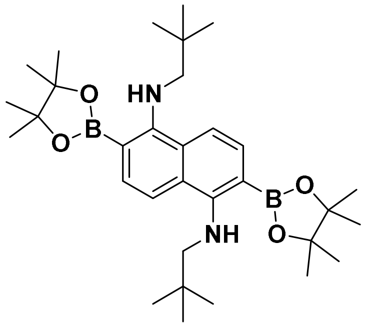

Compound **25** was prepared directly from the respective amide (333 mg, 0.5 mmol, 1 eq) dissolved in DCM (3 mL). To the stirred solution was added BBr_3 (1M solution in DCM) (1 mL, 2 mmol, 2 eq) and heated at 60 °C (in a sealed ampoule) for 18h. To obtained white suspension was then added Et_3SiH (0.4 mL, 2.5 mmol, 5 eq) and stirred at room temperature overnight. The clear solution obtained was evaporated under vacuum to get a dry solid. The biphasic solution of pinacol (130 mg, 1.1 mmol, 2.2 eq in 0.5 mL DCM) and K_2CO_3 (484 mg, 3.5 mmol, 7 eq in 1 mL H_2O) was added and stirred at room temperature overnight. $MgSO_4$ was added to the reaction mixture diluted with DCM and left for an hour. The mixture was then filtered, evaporated, and dried under vacuum. DCM and hexane (1:1) were then added to the crude material and the sample was left in the fridge overnight to get the off white crystalline solid. Filtration and washing with hexane afforded the pure product (112 mg, 40 %). **[Acc. Mass]** Calculated for $[M+H]^+$: 551.4186, found $[M+H]^+$ 551.4188.

^{1}H NMR (500 MHz, $CDCl_3$, 298 K): δ 7.73 (d, *J*=8.5 Hz, 2H), 7.61 (d, *J*=8.5 Hz, 2H), 5.55 (br s, 2H), 3.03 (s, 4H), 1.37 (s, 24H), 1.06 (s, 18H). **^{13}C{^{1}H} NMR** (126 MHz, $CDCl_3$, 298 K): δ 157.0, 131.0, 130.1, 117.8, 113.3, 83.8, 63.8, 33.0, 27.8, 25.1. **^{11}B NMR** (160 MHz, CD_2Cl_2, 298 K): δ 31.7.

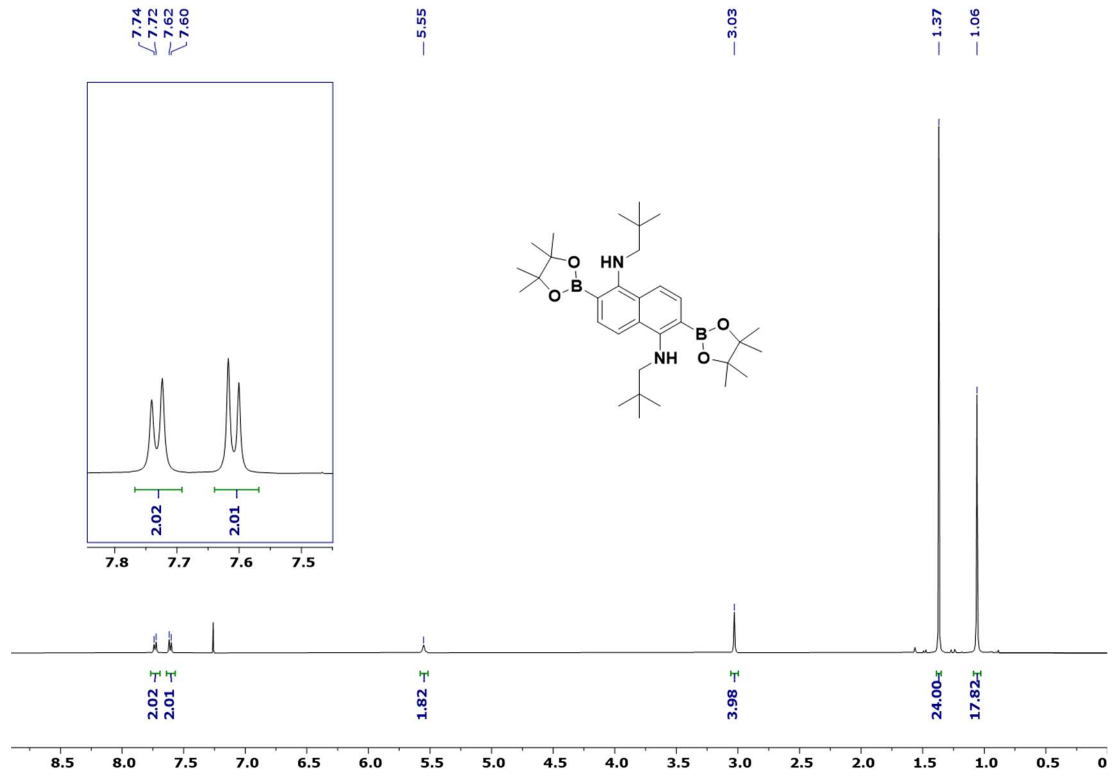

Figure S70. ^{1}H NMR spectrum of **25** in $CDCl_3$.

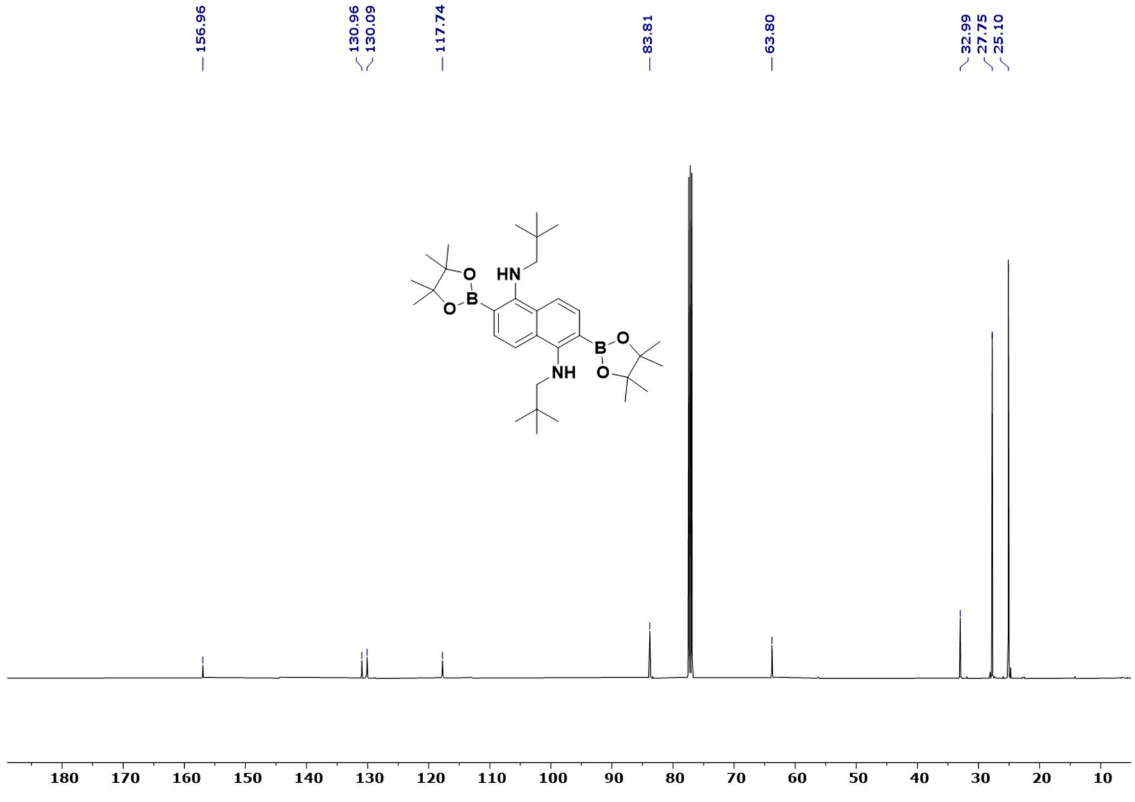

Figure S71. $^{13}C\{^{1}H\}$ NMR spectrum of **25** in $CDCl_3$.

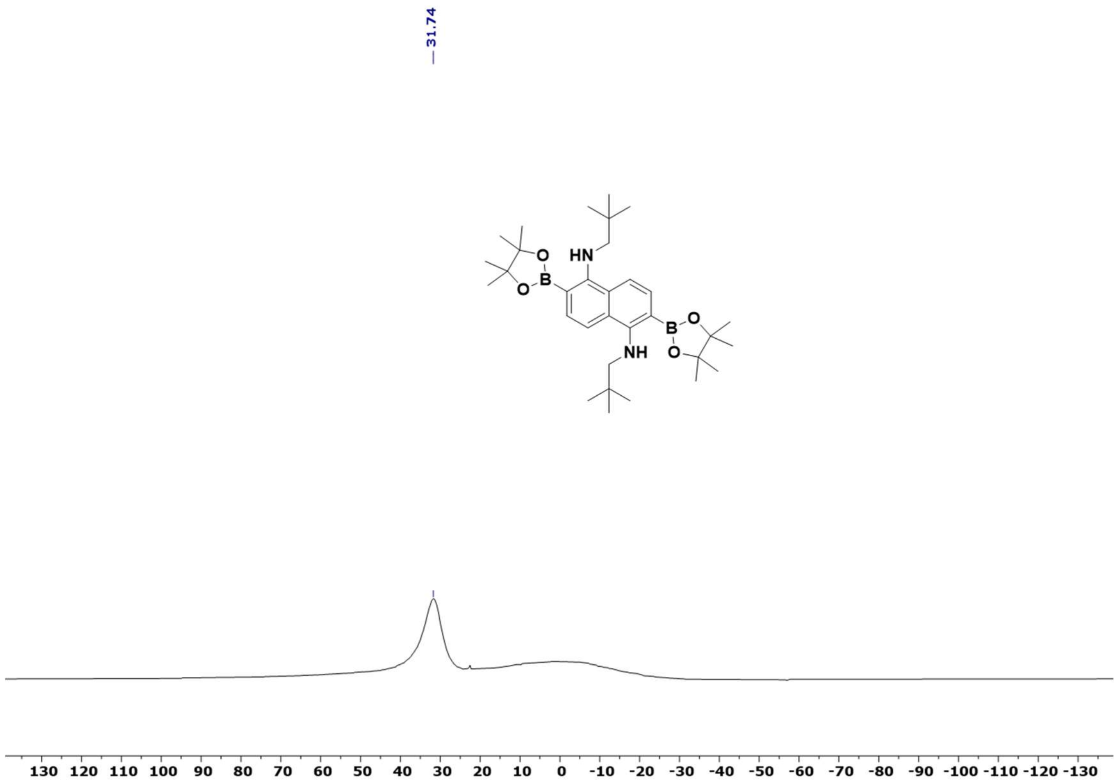

Figure S72. ^{11}B NMR spectrum of **25** in $CDCl_3$.

7 Synthesis and characterisation of B,N-PAH derivatives

7.1 Two step synthesis of *N*-pivaloyl-7-naphthyl-1,2,3,4-tetrahydroquinoline (Compound 26-THQ)

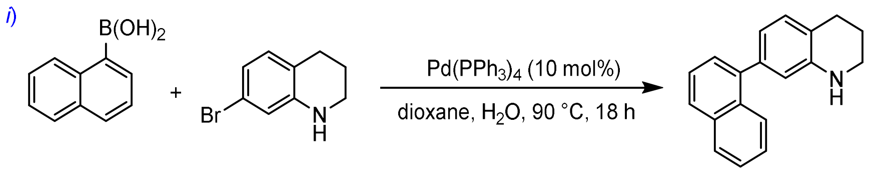

7-naphthyl-1,2,3,4-tetrahydroquinoline

7-bromo-1,2,3,4-tetrahydroquinoline (553 mg, 2.6 mmol, 1 eq), naphthalene-1-boronic acid (516 mg, 3.0 mmol, 1.1 eq) and sodium carbonate (826 mg, 7.8 mmol, 3 eq) were loaded into a Schlenk tube and suspended in a mixture of 1,4-dioxane (16 mL) and water (4 mL). Obtained suspension was degassed under the stream of argon for 30 min. Then tetrakis(triphenylphosphine)palladium(0) (300 mg, 0.26 mmol, 0.1 eq) was added and the mixture was heated at 90 °C for 18 h. Once cooled to room temperature, the reaction mixture was extracted with ethyl acetate and washed with sodium carbonate and brine. The organic fractions were dried over magnesium sulfate and purified by column chromatography on silica gel (petroleum ether/DCM = 8:2) to give 7-naphthyl-1,2,3,4-tetrahydroquinoline as light yellow oil (598 mg, 89 % yield).

^{1}H NMR (500 MHz, $CDCl_3$, ppm): 2.03(q, 2H, CH_2), 2.87(t, 2H, CH_2), 3.37(t, 2H, CH_2), 6.64(s, 1H, C-H), 6.76(d, 1H, C-H), 7.07(d, 1H, C-H), 7.41(m, 1H, C-H), 7.42(m, 1H, C-H), 7.46(m, 1H, C-H), 7.49 (m, 1H, C-H), 7.82(d, 1H, C-H), 7.88 (d, 1H, C-H), 8.02(d, 1H, C-H). **^{13}C{^{1}H} NMR** (500 MHz, $CDCl_3$, ppm): 22.07, 26.71, 42.24, 117.09, 120.69, 121.82, 125.48, 125.78, 125.95, 126.46, 126.78, 127.48, 128.28, 129.57, 131.81, 133.90, 139.61, 140.47, 142.79

[Acc. Mass] Calculated $[M+H]^+$: 260.1434 gmol^{-1}, Observed $[M+H]^+$ 260.1443 gmol^{-1}

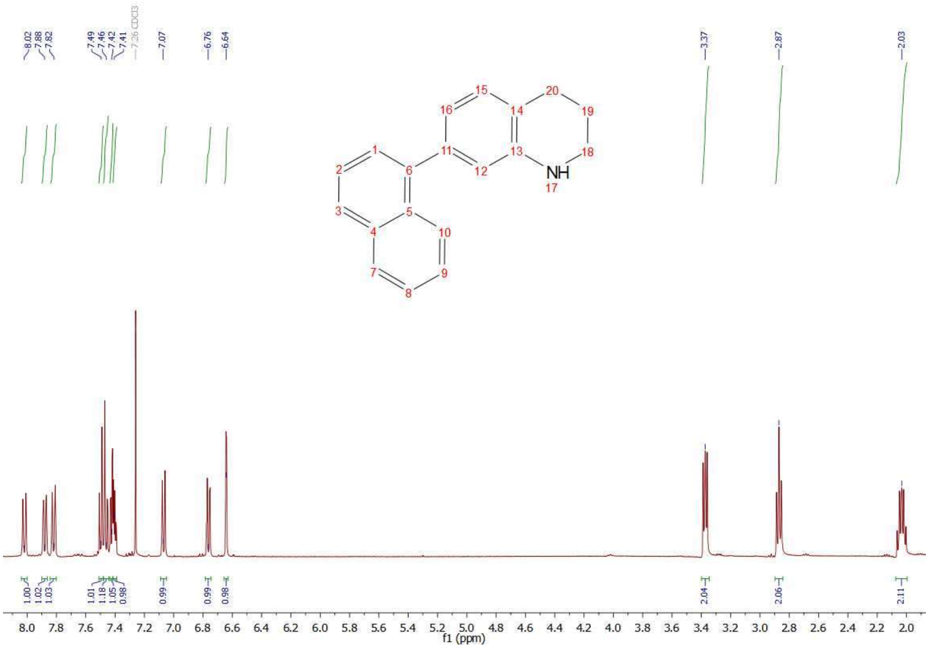

Figure S73. ^{1}H NMR spectrum of 7-naphthalen-1-yl-1,2,3,4-tetrahydroquinoline in $CDCl_3$.

ii)

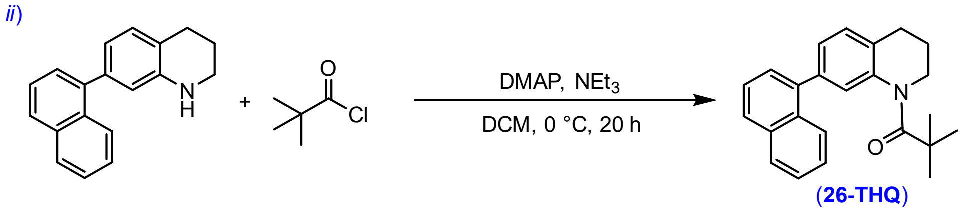

Obtained 7-naphthyl-1,2,3,4-tetrahydroquinoline (446 mg, 1.7 mmol, 1 eq) was loaded into a Schlenk tube, followed by 4-N,N-dimethylaminopyridine (42 mg, 0.34 mmol, 0.2 eq) and dissolved in dry DCM (4 mL). Dry triethylamine (0.36 mL, 2.6 mmol, 1.5 eq) was added and the solution was cooled to 0 °C (ice/water bath). Pivaloyl chloride (0.25 mL, 2 mmol, 1.2 eq) was dropwise added and the mixture was stirred at 0 °C for 10 min, and then overnight at room temperature. All volatiles were removed under vacuum and the crude was extracted with ethyl acetate and washed with ammonium chloride solution. The combined organic layers were dried over magnesium sulfate and purified by column chromatography on silica gel (petroleum ether/ ethyl acetate = 9:1) to afford N-pivaloyl-7-naphtyhyl-1,2,3,4-tetrahydroquinoline (**26-THQ**) as off-white solid (440 mg, 75 % yield).

^{1}H NMR (500 MHz, $CDCl_3$, ppm): 1.32(s, 9H, $C(CH_3)_3$), 2.08(q, 2H, CH_2), 2.89(t, 2H, CH_2), 3.87(t, 2H, CH_2), 7.22(m, 1H, C-H), 7.23(m, 1H, C-H), 7.43(m, 1H, C-H), 7.46(m, 1H, C-H), 7.49(m, 1H, C-H), 7.51(m, 1H, C-H), 7.54(d, 1H, C-H), 7.83(d, 1H, C-H), 7.89(d, 1H, C-H), 7.96(d, 1H, C-H). **^{13}C{^{1}H} NMR** (500 MHz, $CDCl_3$, ppm): 24.31, 26.17, 29.09, 40.51, 45.52, 125.48, 125.83, 126.14, 126.27, 126.87, 127.12, 127.47, 127.70, 128.34, 128.53, 130.61, 131.83, 133.91, 138.28, 139.94, 140.94, 178.38

[Acc. Mass] Calculated $[M+H]^+$: 343.2010 $gmol^{-1}$, Observed $[M+H]^+$ 343.1974 $gmol^{-1}$

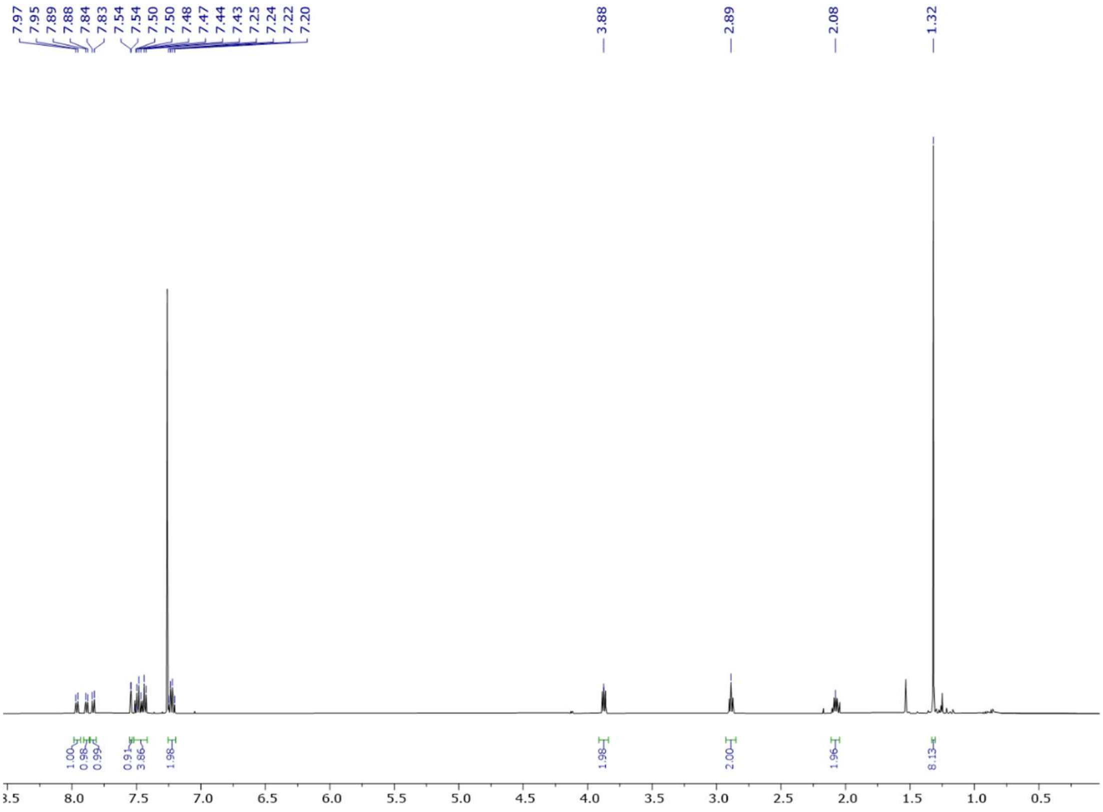

Figure S74. ^{1}H NMR spectrum of **26-THQ** in $CDCl_3$ at room temperature.

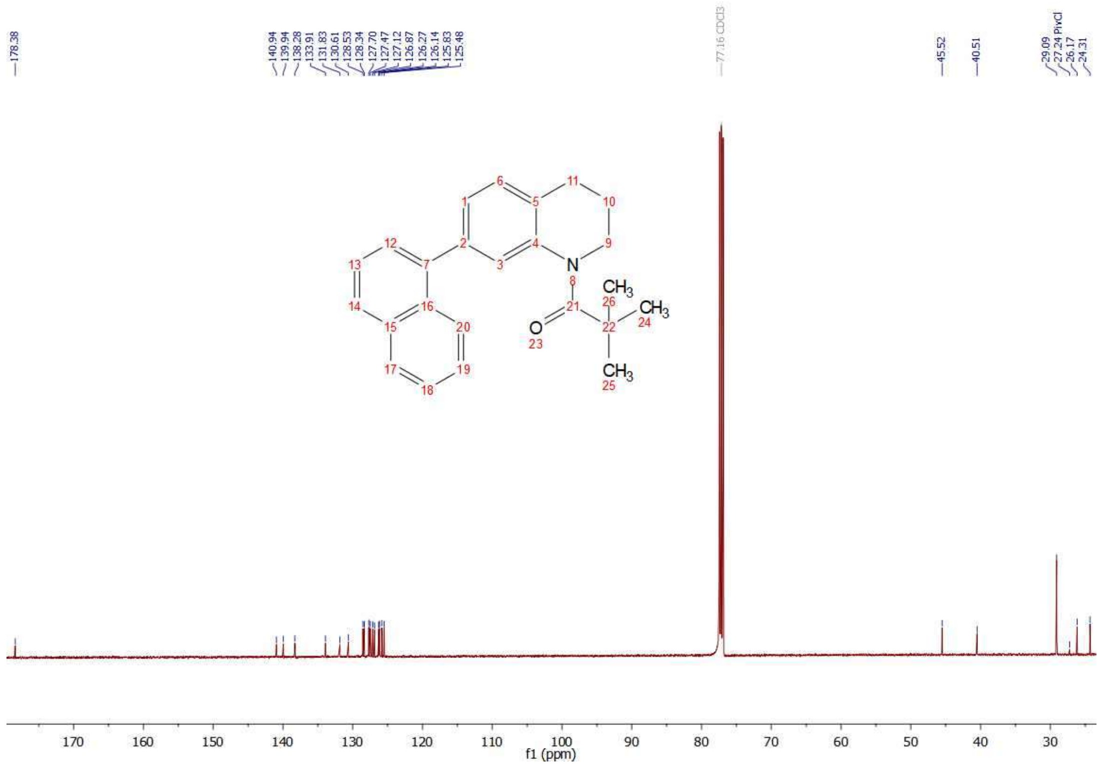

Figure S75. $^{13}C\{^{1}H\}$ NMR spectrum of **26-THQ** in $CDCl_3$ at room temperature.

7.2 Directed borylation of *N*-pivaloyl-7-naphthyl-1,2,3,4-tetrahydroquinoline (27-THQ)

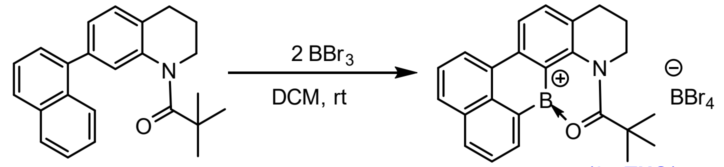

(27-THQ)

BBr_3 1M in DCM (2.6 mL, 2.6 mmol, 2 eq) was added to a stirred solution of *N*-pivaloyl-7-naphthyl-1,2,3,4-tetrahydroquinoline (0.44 g, 1.3 mmol, 1 eq) in dry DCM (5 mL). The reaction mixture was stirred for 10 min and then left to stand at room temperature overnight affording light yellow crystals of [**(27-THQ)·DCM**] (821 mg, 84 % yield). **[Acc. Mass]** Calculated for cation $C_{24}H_{23}BNO$ $[M]^+$: 352.1867, found $[M+H]^+$ 352.1854. Note the BBr_4^- peak was also observed in the negative mode.

^{1}H NMR (500 MHz, CD_2Cl_2, 298 K): δ 8.68 (dd, *J*=7.7, 1.2 Hz, 1H,CH_{N2}), 8.58 (dd, *J*=6.9, 1.4 Hz, 1H, CH_{N8}), 8.53 (d, *J*=8.2 Hz, 1H, CH_{Q6}), 8.36 (dd, *J*=8.1, 1.4 Hz, 1H, CH_{N6}), 8.16 (dd, *J*=8.1, 1.1 Hz, 1H, CH_{N4}), 7.89 (dd, *J*=8.3, 1.0 Hz, 1H, CH_{Q5}), 7.84 (dd, *J*=8.1, 6.9 Hz, 1H, CH_{N7}), 7.80 (t, *J*=7.8 Hz, 1H, CH_{N3}), 4.73 (m, 1H, Q1-CH_2), 3.22 (t, *J* = 6.1 Hz, 2H, Q3-CH_2), 2.45 (m, 2H, Q2-CH_2), 1.87 (s, 9H, CH_3) ppm.**^{13}C{^{1}H} NMR** (126 MHz, CD_2Cl_2, 298 K): δ 176.8 (C_{P3}), 141.3(C_{Q7}), 137.7 (C_{Q9}), 137.3 (C_{Q5}), 137.1 (C_{N6}), 136.0 (C_{N8}), 133.9 (C_{N5}), 133.6 (C_{N10}), 133.0 (C_{N4}), 129.8(C_{N1}), 127.9 (C_{Q4}), 127.2 (C_{N2}), 127.0 (C_{N3}), 126.7 (C_{N7}), 125.3 (C_{Q6}), 51.8 (Q1-CH_2), 42.6 (P2-$C(CH_3)_3$), 28.8 (P1-C($CH_3)_3$), 26.5 (Q3-CH_2), 21.6 (Q2-CH_2) ppm. Resonances for C_{N9} and C_{Q8} were not detected in ^{13}C{^{1}H} due to quadrupolar effects, but a cross-peak in ^{13}C{^{1}H} – ^{13}C{^{1}H} HMBC spectrum at 122.5 ppm can be assigned to C_{Q8}. **^{11}B NMR** (160 MHz, CD_2Cl_2, 298 K): δ 36.53, -24.47 ppm.

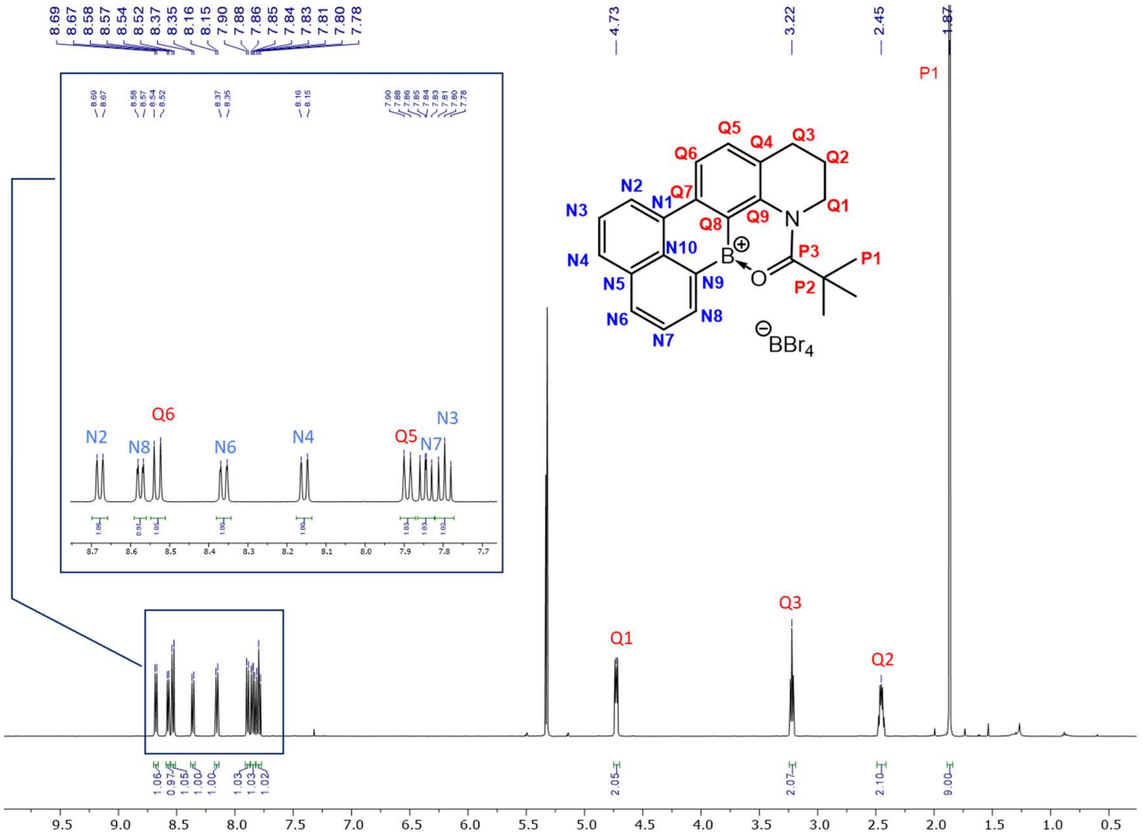

Figure S76. ^{1}H NMR of **27-THQ** in CD_2Cl_2 at room temperature.

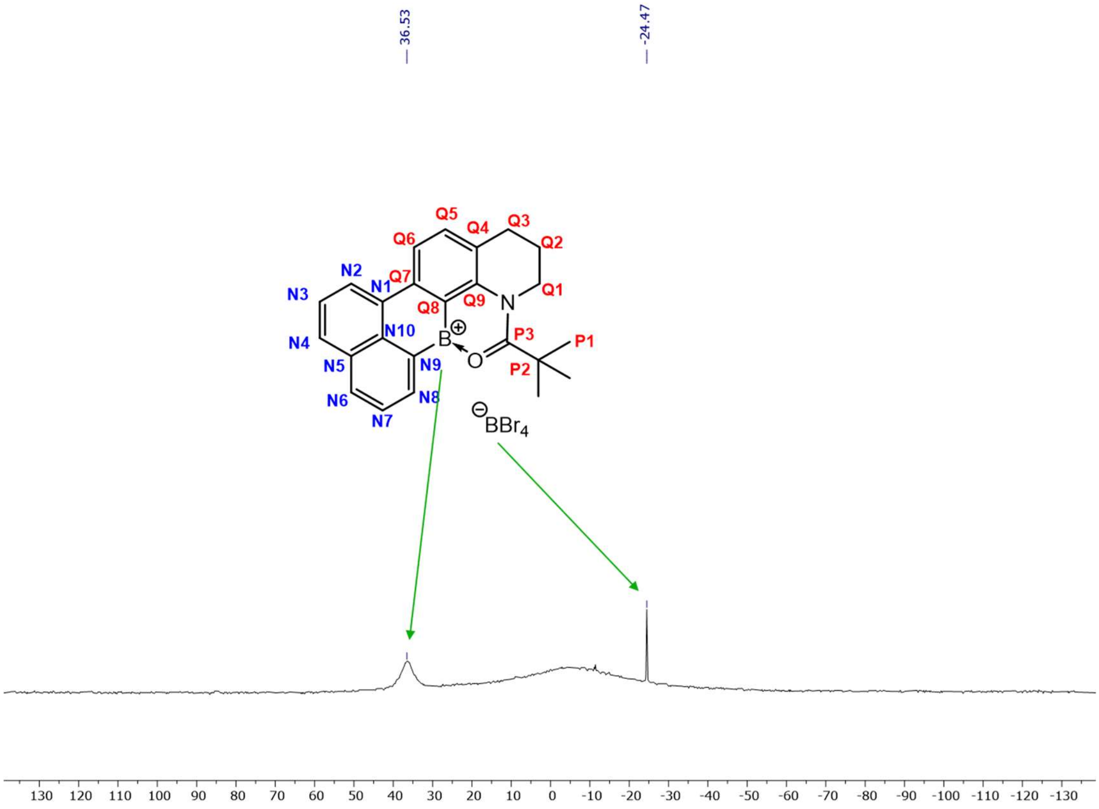

Figure S77. ^{11}B NMR of **27-THQ** in CD_2Cl_2 at room temperature.

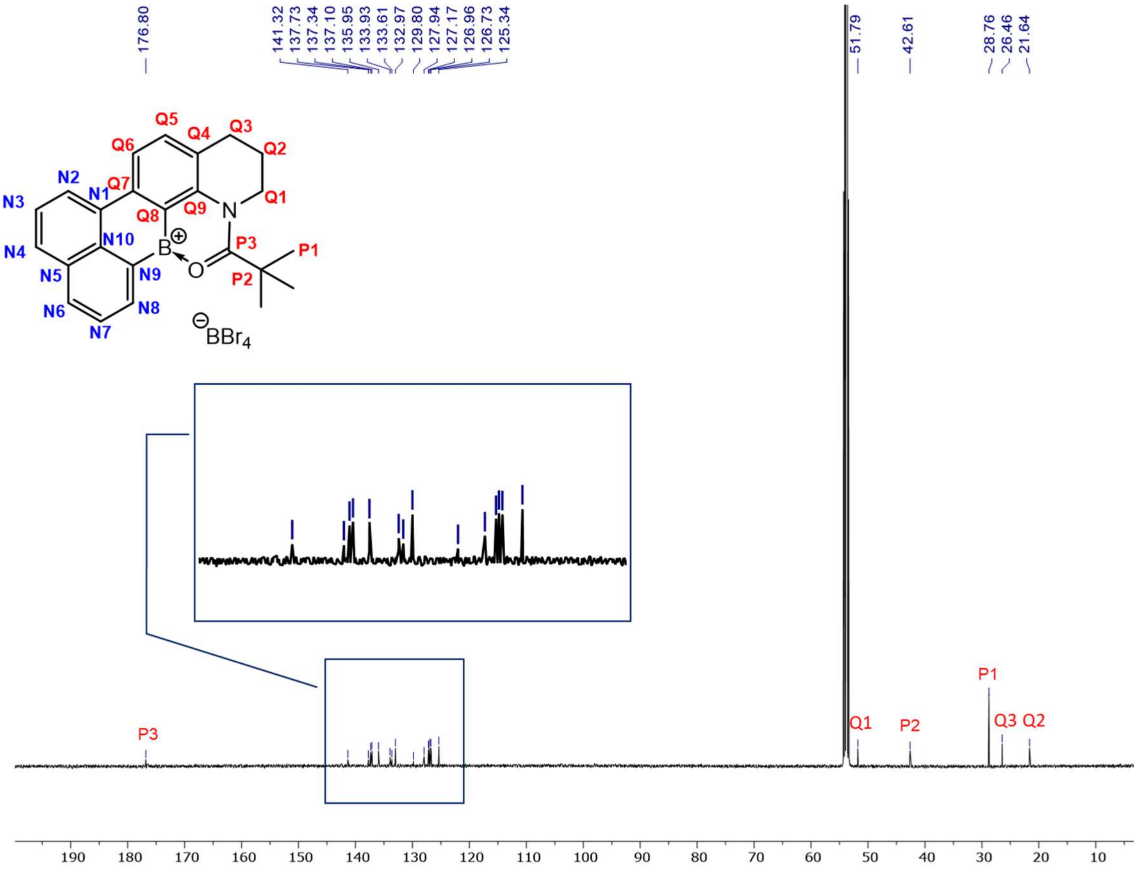

Figure S78. $^{13}C\{^1H\}$ NMR of **27-THQ** in CD_2Cl_2 at room temperature.

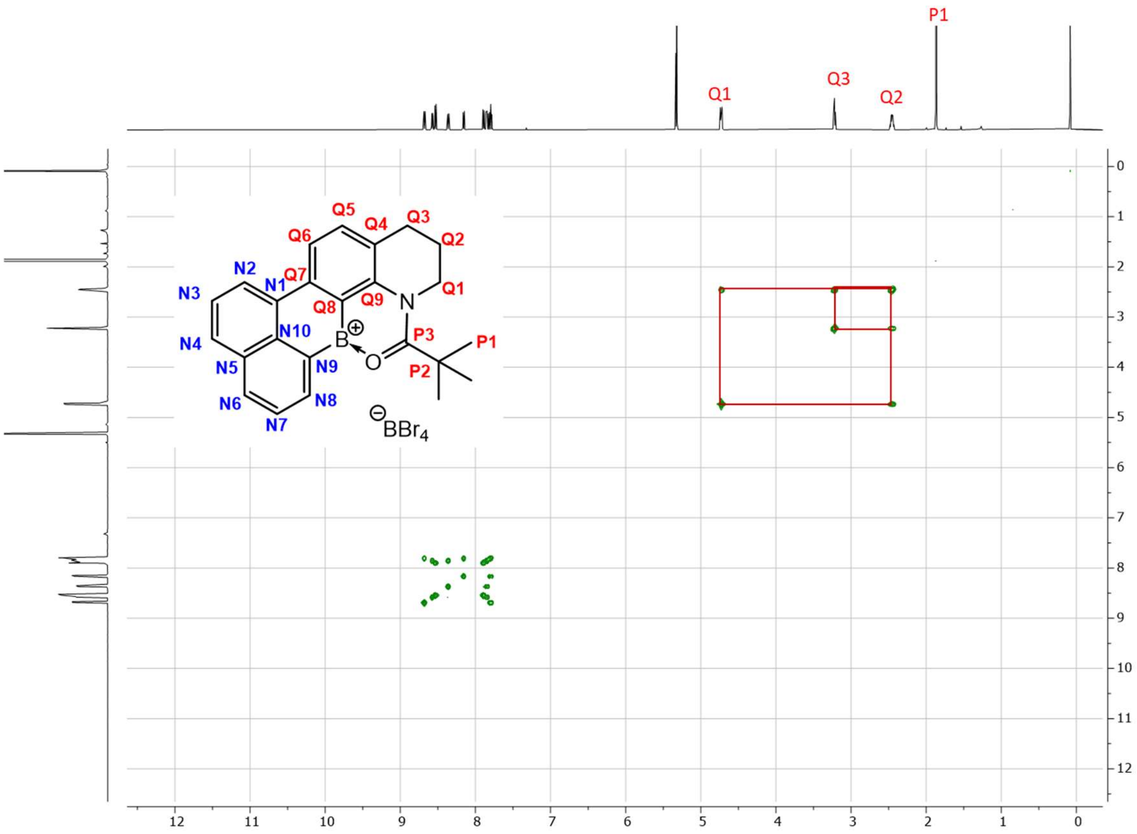

Figure S79. ^1H – ^1H COSY NMR spectrum of **27-THQ** in CD_2Cl_2 at room temperature.

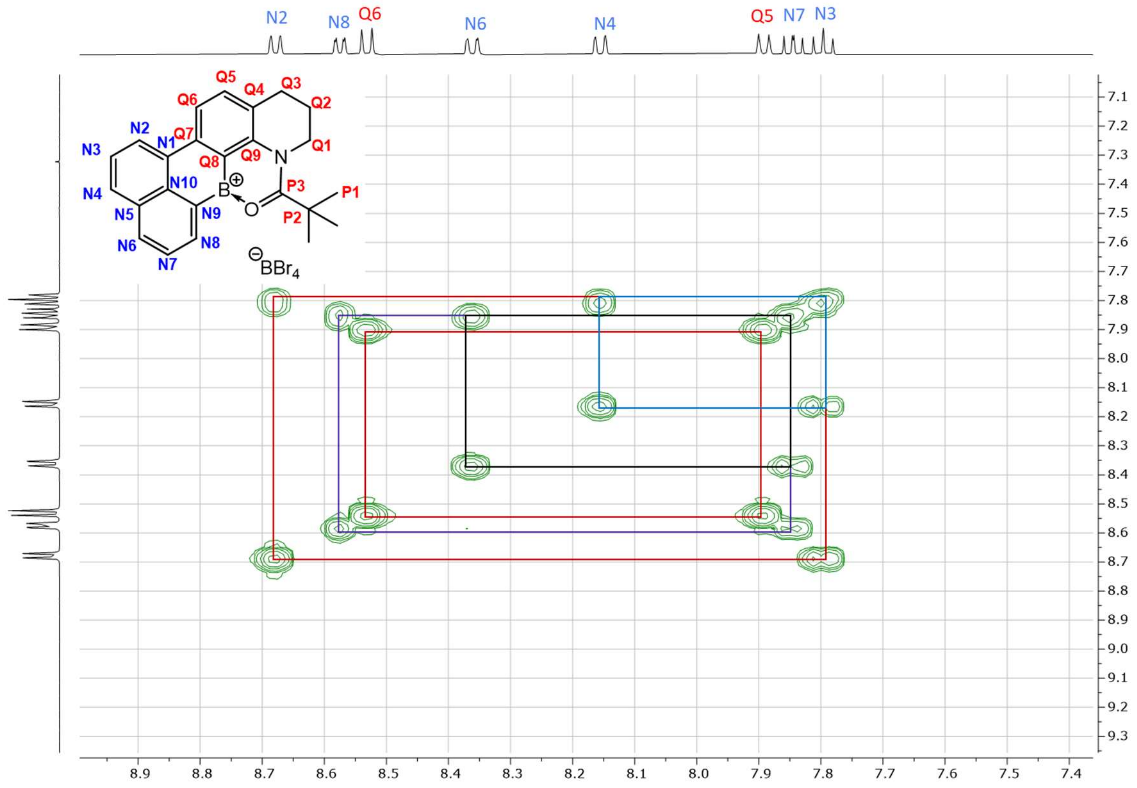

Figure S80. ^1H – ^1H COSY NMR spectrum of **27-THQ** in CD_2Cl_2 at room temperature.

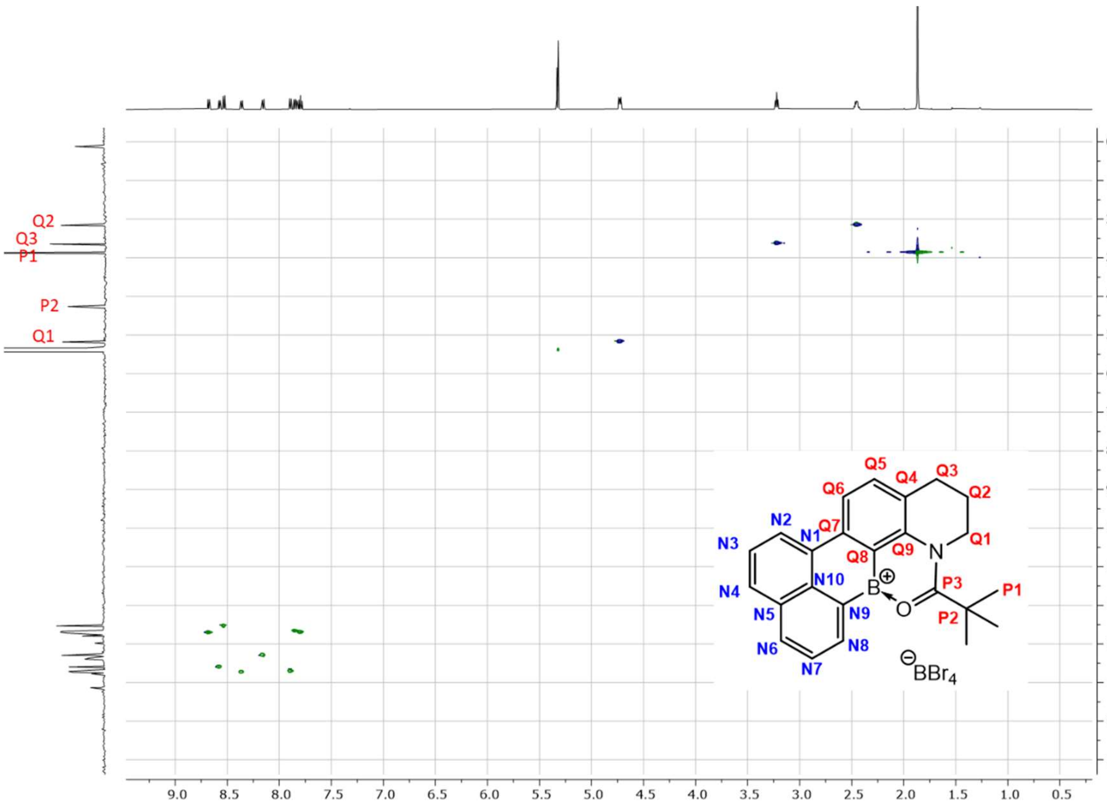

Figure S81. ^1H – $^{13}C\{^1H\}$ HSQC NMR spectrum of **27-THQ** in CD_2Cl_2 at room temperature.

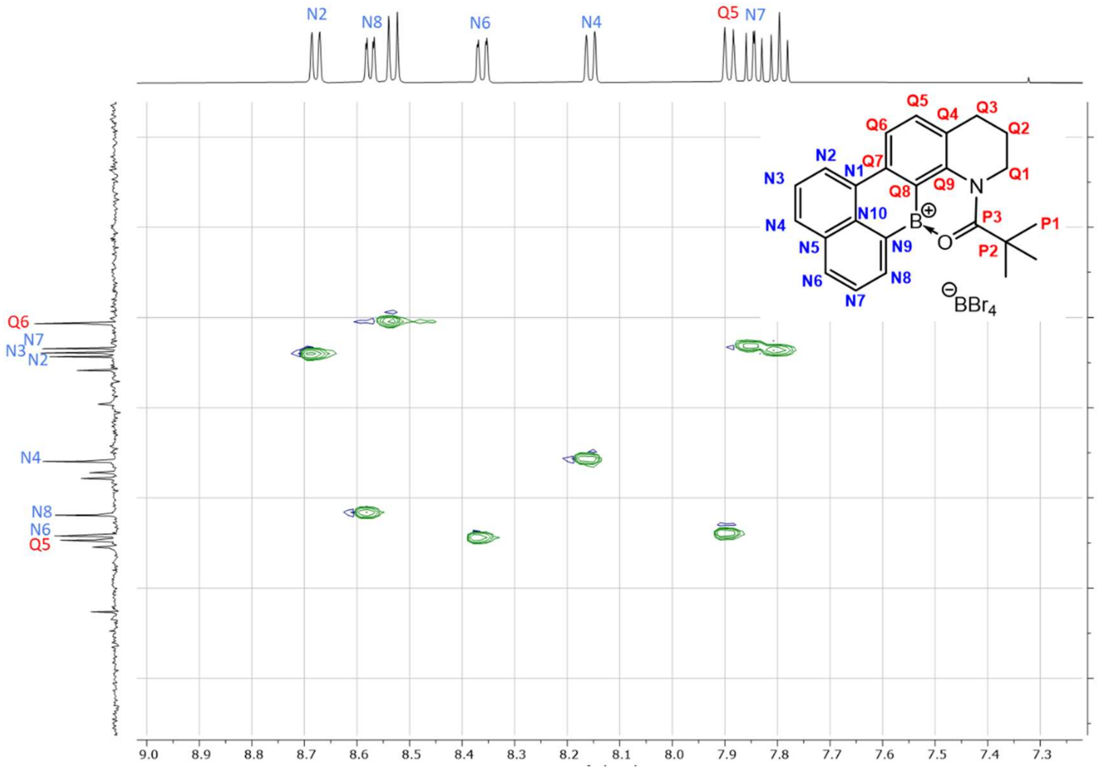

Figure S82. ^1H – $^{13}C\{^1H\}$ HSQC NMR spectrum of **27-THQ** in CD_2Cl_2 at room temperature.

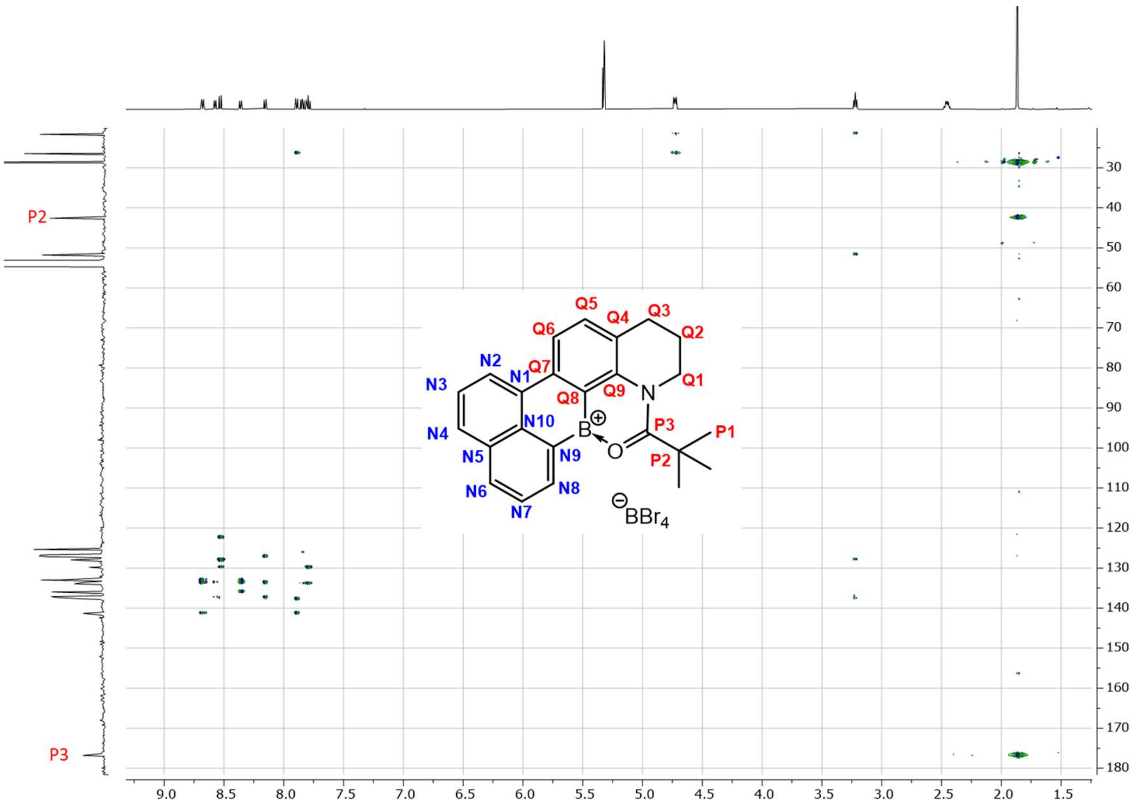

Figure S83. ^{1}H – $^{13}C\{^{1}H\}$ HMBC NMR spectrum of **27-THQ** in CD_2Cl_2 at room temperature.

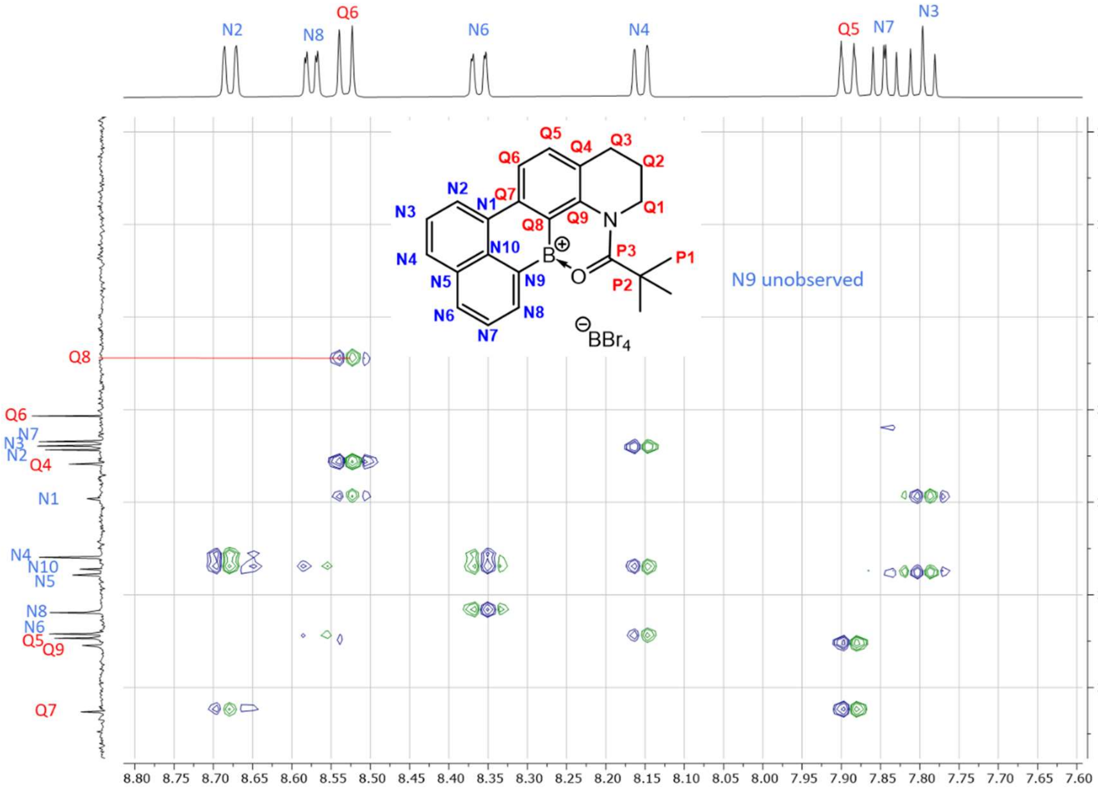

Figure S84. ^{13}C – $^{13}C\{^{1}H\}$ HMBC NMR spectrum of **27-THQ** in CD_2Cl_2 at room temperature.

7.3 Reduction of 27-THQ with triethylsilane to afford 28-THQ

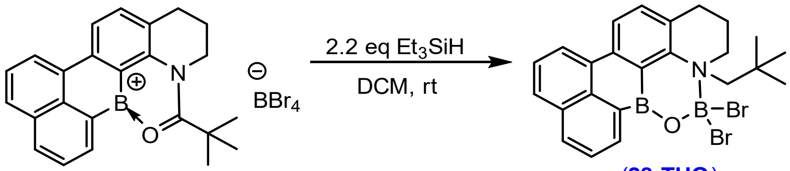

To a suspension of **27-THQ·DCM** (173 mg, 0.22 mmol, 1 eq) in DCM (5 mL) triethylsilane (76 µL, 0.48 mmol, 2.2 eq) was added. The mixture was stirred for 2 h at room temperature until a clear dark yellow solution was obtained. All volatiles were removed under vacuum, and benzene (5 mL) was added which induced precipitation of white solid. The liquid phase was decanted and white precipitate washed with more benzene (3 mL). Once again the liquid was decanted and the white solid dried under vacuum affording **28-THQ·C_6H_6** (98 mg, 73 % yield). Crystals suitable for X-ray diffraction were grown from a solution in DCM layered with benzene at room temperature. Anal. Calc. for $C_{36}H_{37}B_2Br_2NO$: C, 63.48; H, 5.48; N, 2.06; found: C, 63.25; H, 5.74; N, 2.99. The microanalysis data corresponds to **28-THQ** with two molecules of benzene.

^{1}H NMR (500 MHz, $CDCl_3$, 298 K): δ 6.68 (dd, *J*=6.8, 1.5 Hz, 1H,Ar-C*H*), 8.49 (dd, *J*=7.7, 1.1 Hz, 1H, Ar-C*H*), 8.533 (d, *J*=8.3 Hz, 1H, Ar-C*H*), 8.20 (dd, *J*=8.1, 1.5 Hz, 1H, Ar-C*H*), 8.01 (dd, *J*=8.1, 1.1 Hz, 1H, Ar-C*H*), 7.76 (dd, *J*=8.1, 6.8 Hz, 1H, Ar-C*H*), 7.66 (t, *J*=7.7, 1H, Ar-C*H*), 7.49 (d, *J*=8.2 Hz, 1H, Ar-C*H*), 4.77 (m, 1H, C*H*$_2$), 3.95 (d, *J* = 14.5 Hz, 1H, C*H*$_2$), 3.78 (d, *J* = 14.5 Hz, 1H, C*H*$_2$), 3.69 (m, 1H, C*H*$_2$), 3.11 (m, 1H, C*H*$_2$), 2.95 (m, 1H, C*H*$_2$), 2.73 (m, 1H, C*H*$_2$), 2.24 (m, 1H, C*H*$_2$), 0.64 (s, 9H, C*H*$_3$) ppm. **^{11}B NMR** (160 MHz, $CDCl_3$, 298 K): δ 40.3, 6.1 ppm.

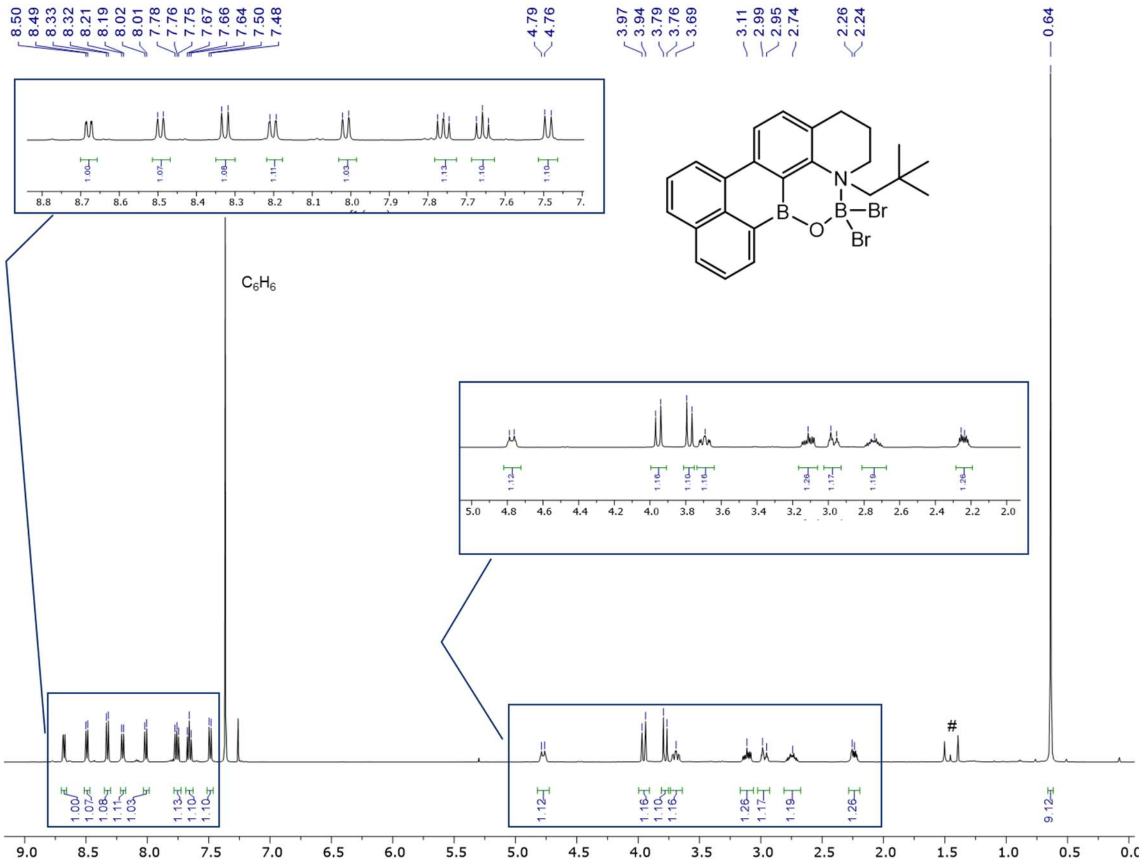

Figure S85. ^{1}H NMR spectrum of **28-THQ** in $CDCl_3$ at room temperature. # denotes unidentified impurity.

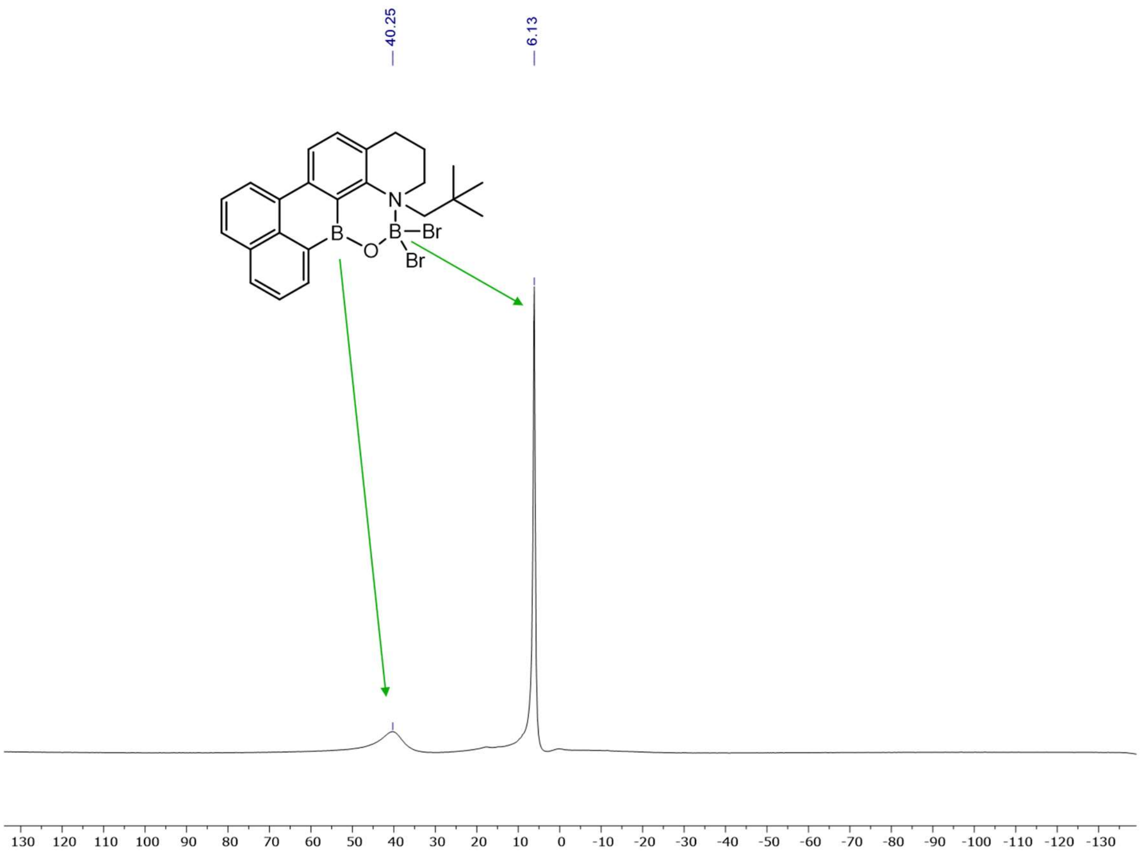

Figure S86. ^{11}B NMR spectrum of **28-THQ** in $CDCl_3$ at room temperature.

7.4 Isolation of 29-THQ

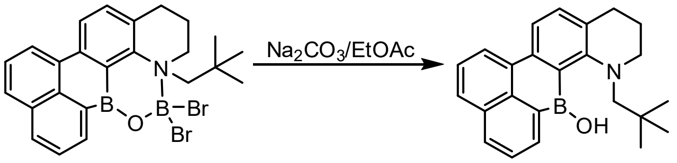

(**29-THQ**)

28-THQ·C_6H_6 (97 mg, 0.16 mmol, 1 eq) was treated with ethyl acetate and aqueous solution of sodium carbonate. Organic layer was washed with brine and dried over magnesium sulfate. Obtained bright yellow solution was evaporated to dryness and yellow solid of **29-THQ** was isolated (57 mg, quantitative). Crystals suitable for X-ray diffraction were grown from a solution in $CHCl_3$ left at +4 °C for several days.

^{1}H NMR (500 MHz, $CDCl_3$, 298 K): δ 11.9 (s, 1H, BO*H*), 8.52 (dd, *J*=6.7, 1.5 Hz, 1H,CH_{N8}), 8.37 (m, 1H, CH_{N2}), 8.09 (m, 2H, CH_{Q6} and CH_{N6} overlapping), 7.91 (dd, *J*=8.0, 1.1 Hz, 1H, CH_{N4}), 7.69 (dd, *J*=8.1, 6.8 Hz, 1H, CH_{N7}), 7.59 (dd, *J*=8.1, 7.5 Hz, 1H, CH_{N3}), 7.32 (dd, *J*=8.2, 0.9 Hz, 1H, CH_{Q5}), 3.65 (br s, 1H, CH_{Q1}), 3.19 (br s, 1H, CH_{Q1}), 2.97 (m, 3H, Q3-CH_2 and CH_{P3} overlapping), 2.39 (br s, 1H, CH_{Q2}), 1.75 (br s, 1H, CH_{Q2}), 1.15 (s, 9H, CH_3) ppm. **^{13}C{^{1}H} NMR** (126 MHz, $CDCl_3$, 298 K): δ 157.5 (C_{Q9}), 142.7(C_{Q7}), 133.5(C_{N10}), 133.3(C_{N8}), 133.2 (C_{N6}), 133.1 (C_{N5}), 133.0 (C_{Q5}), 132.4 (C_{N1}), 130.2 (C_{Q4}), 129.5 (C_{N4}), 125.9 (C_{N7}), 125.7 (C_{N3}), 123.8(C_{N2}), 121.2 (C_{Q6}), 67.9 (P3-*C*H_2), 46.5 (Q1-*C*H_2), 32.4 (P2-*C*(CH_3)$_3$), 29.6 (P1-C(*C*H_3)$_3$), 25.9 (Q3-*C*H_2), 15.6 (Q2-*C*H_2) ppm. Resonances for C_{N9} and C_{Q8} were not detected in ^{13}C{^{1}H} due to quadrupolar effects. **^{11}B NMR** (160 MHz, $CDCl_3$, 298 K): δ 40.8 ppm.

[Acc. Mass] Calculated for $C_{24}H_{27}BNO$ $[M+H]^+$: 356.2180, found $[M+H]^+$ 356.2195.

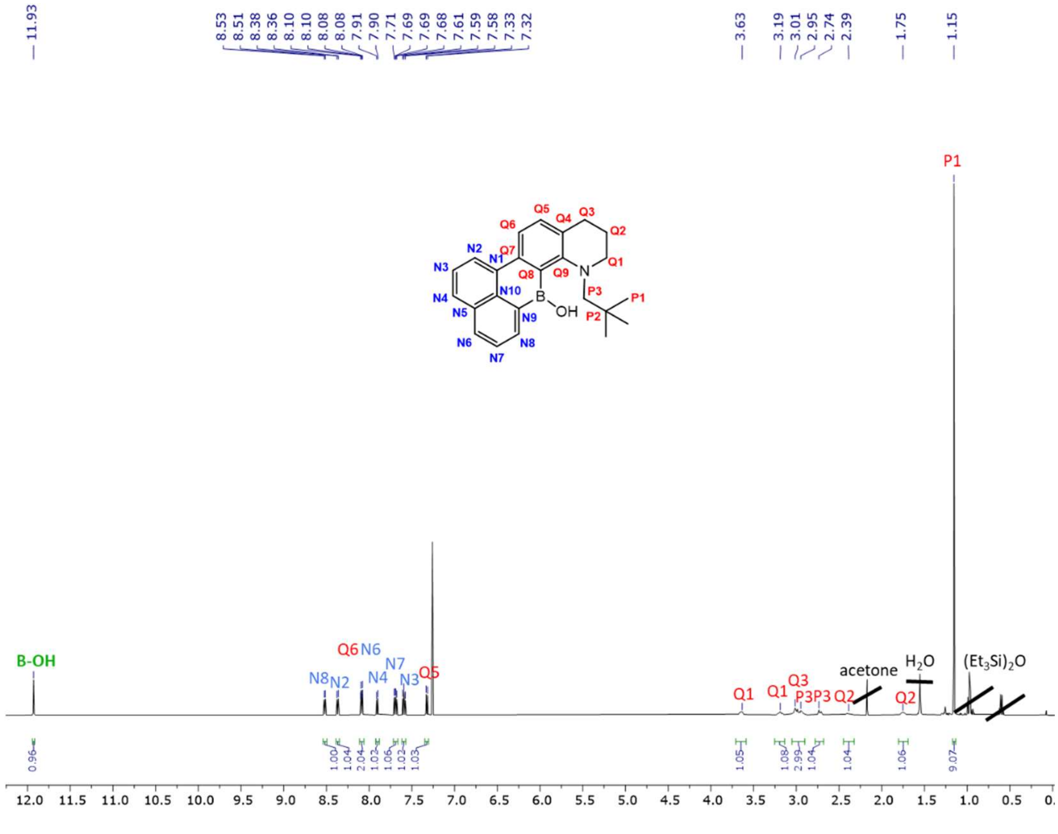

Figure S87. ^{1}H NMR spectrum of **29-THQ** in $CDCl_3$ at room temperature.

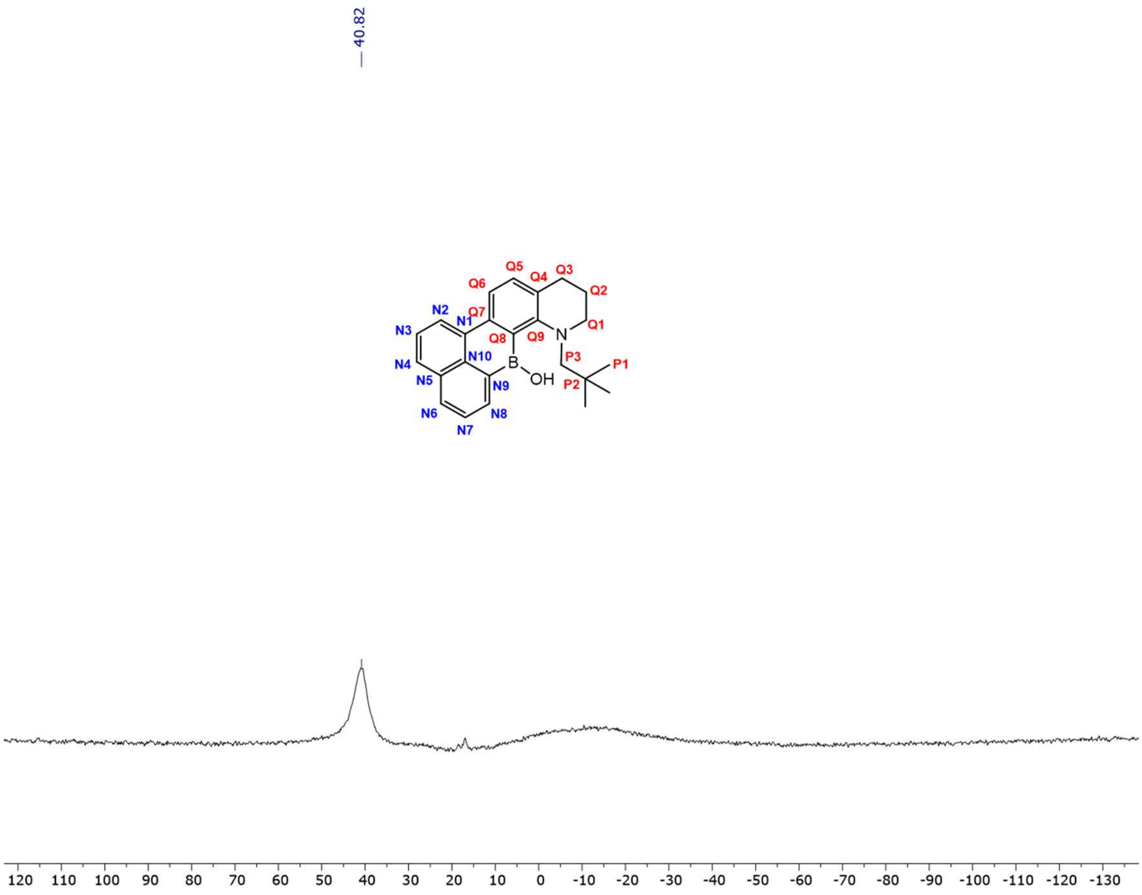

Figure S88. ^{11}B NMR spectrum of **29-THQ** in $CDCl_3$ at room temperature.

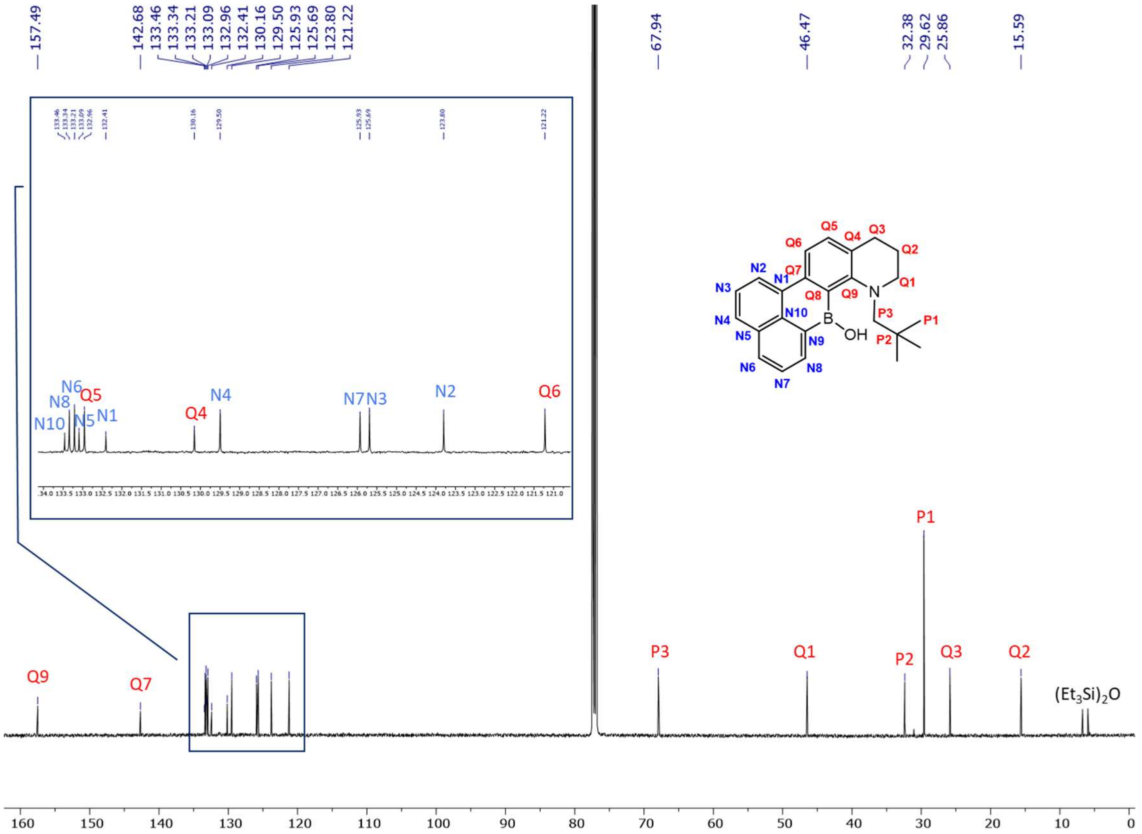

Figure S89. $^{13}C\{^1H\}$ NMR spectrum of **29-THQ** in $CDCl_3$ at room temperature.

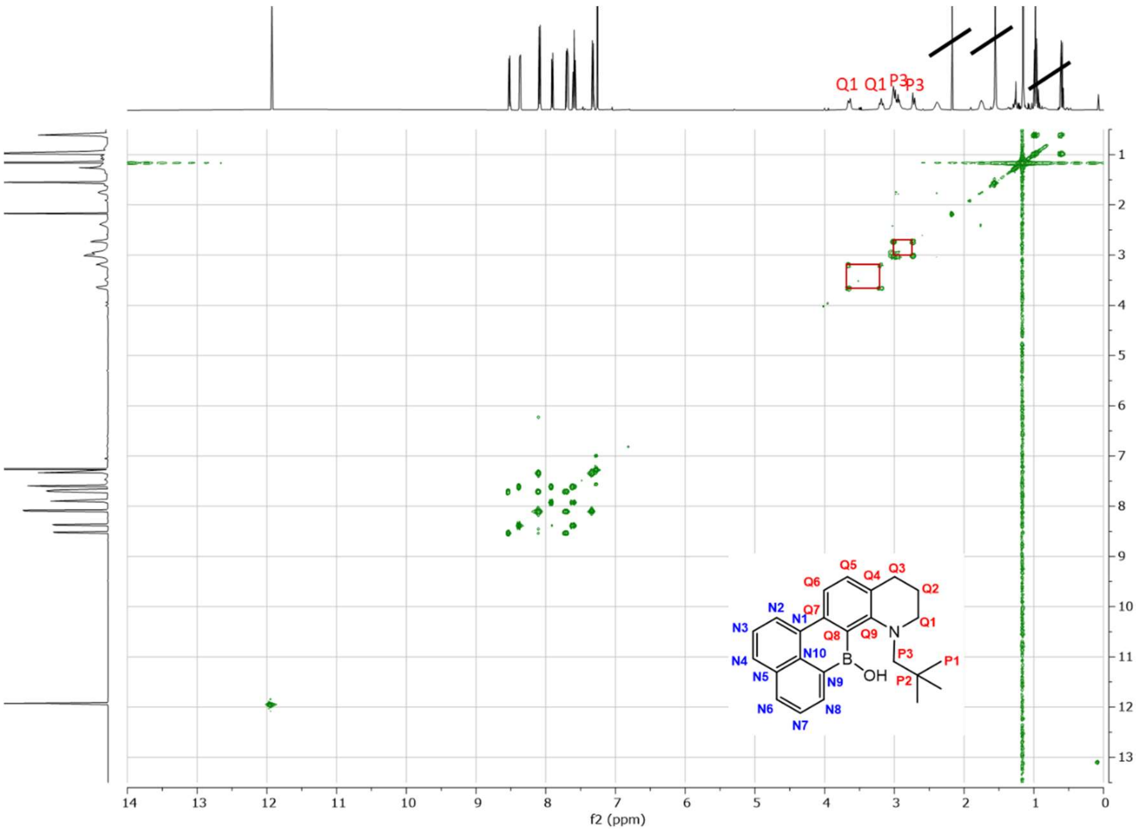

Figure S90. $^{1}H - ^{1}H$ COSY NMR spectrum of **29-THQ** in $CDCl_3$ at room temperature.

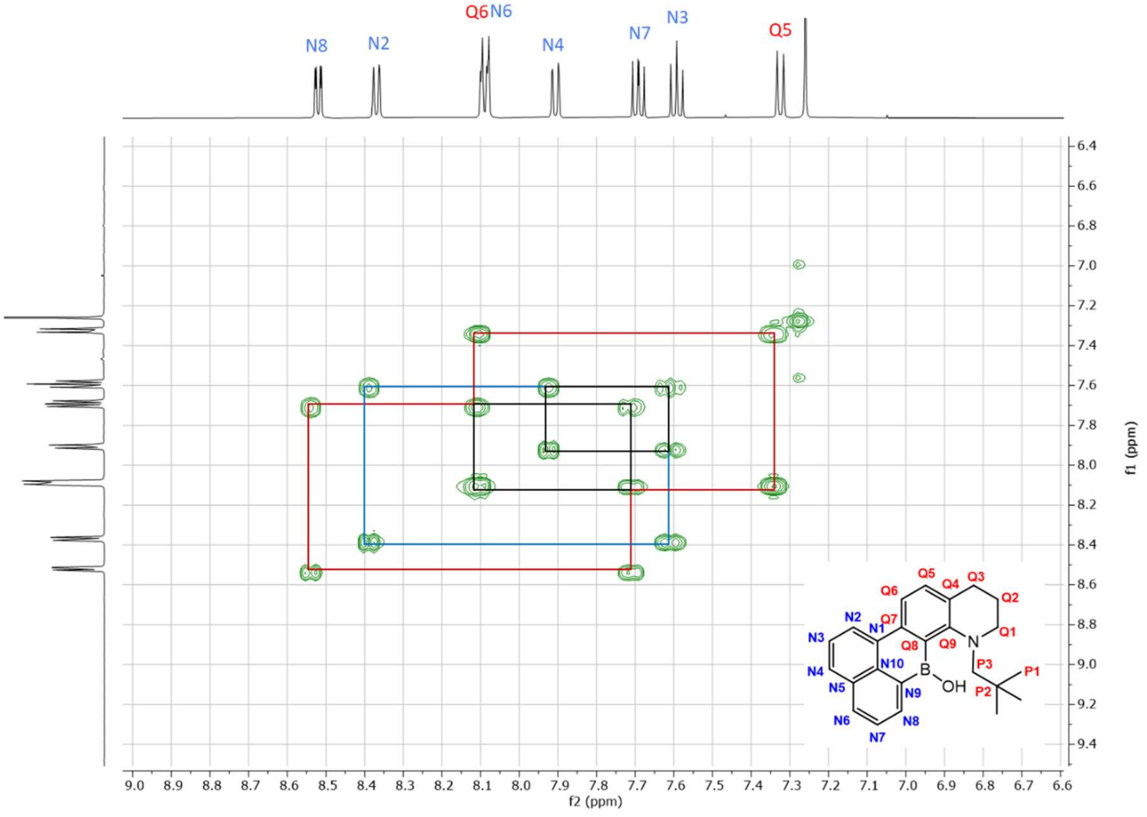

Figure S91. $^{1}H - ^{1}H$ COSY NMR spectrum of **29-THQ** in $CDCl_3$ at room temperature.

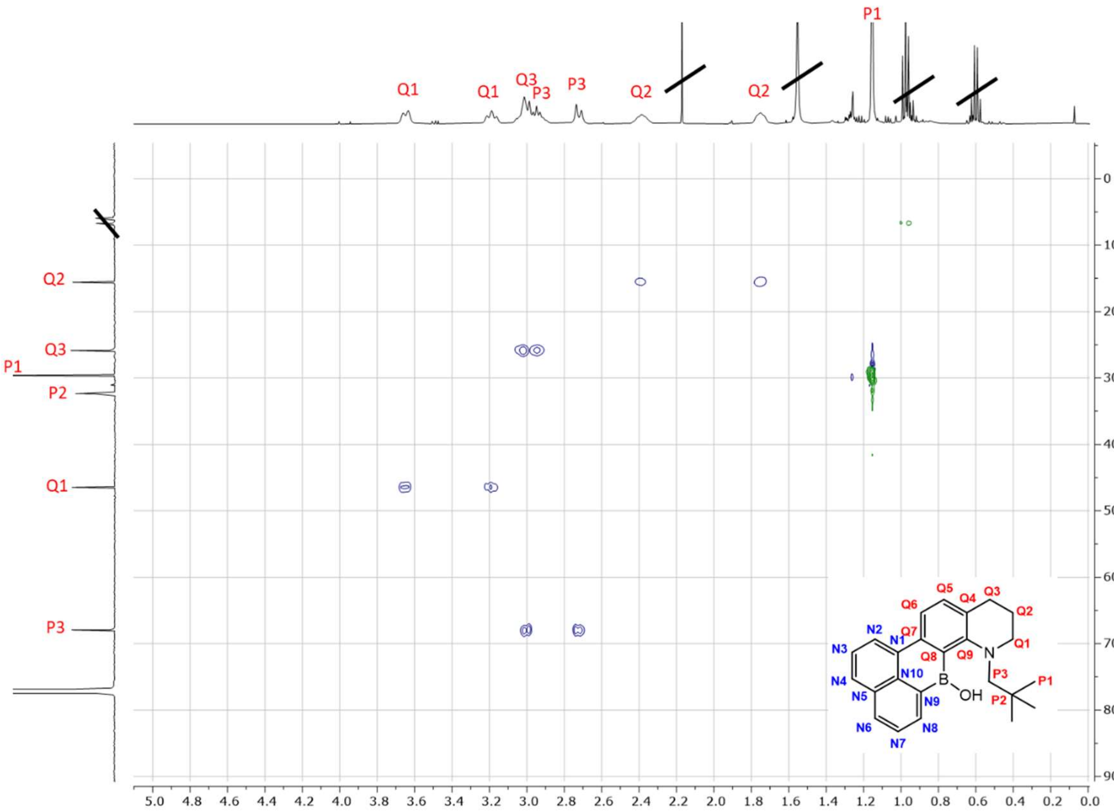

Figure S92. ^{1}H - $^{13}C\{^{1}H\}$ HSQC NMR spectrum **29-THQ** in $CDCl_3$ at room temperature.

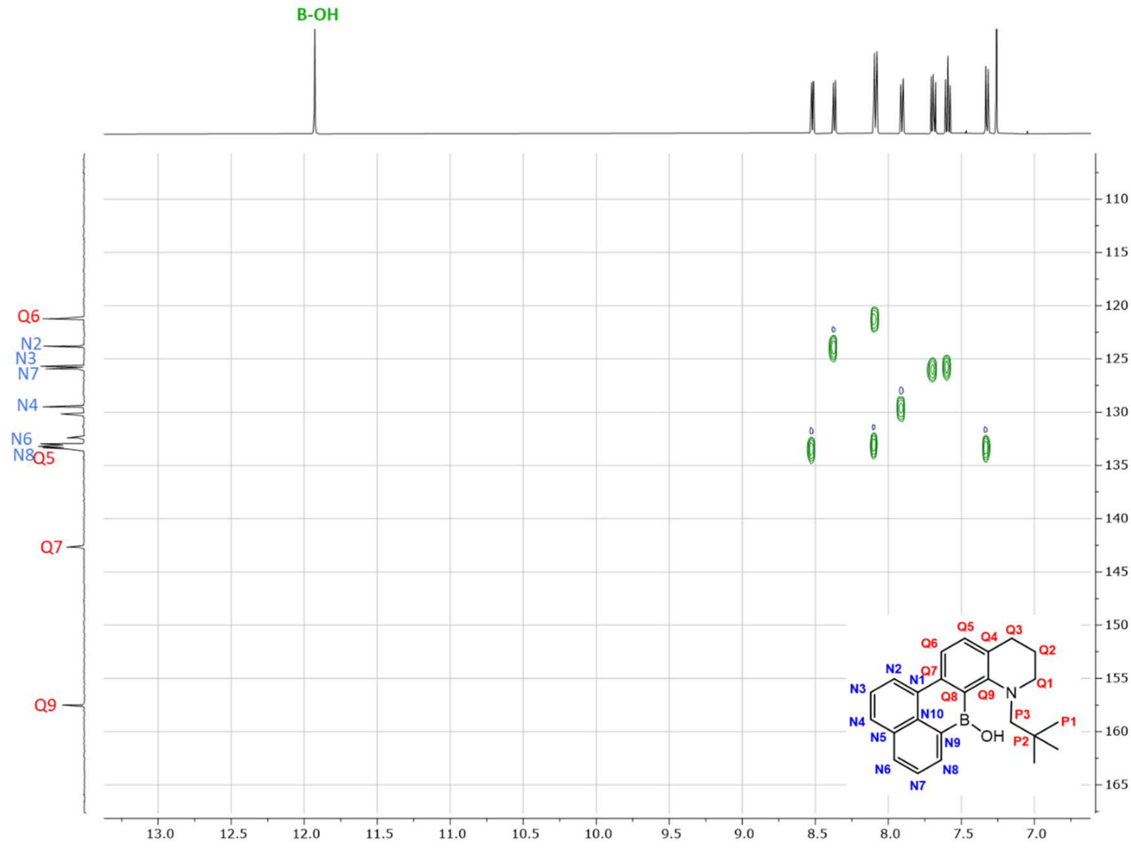

Figure S93. ^{1}H - $^{13}C\{^{1}H\}$ HSQC NMR spectrum **29-THQ** in $CDCl_3$ at room temperature.

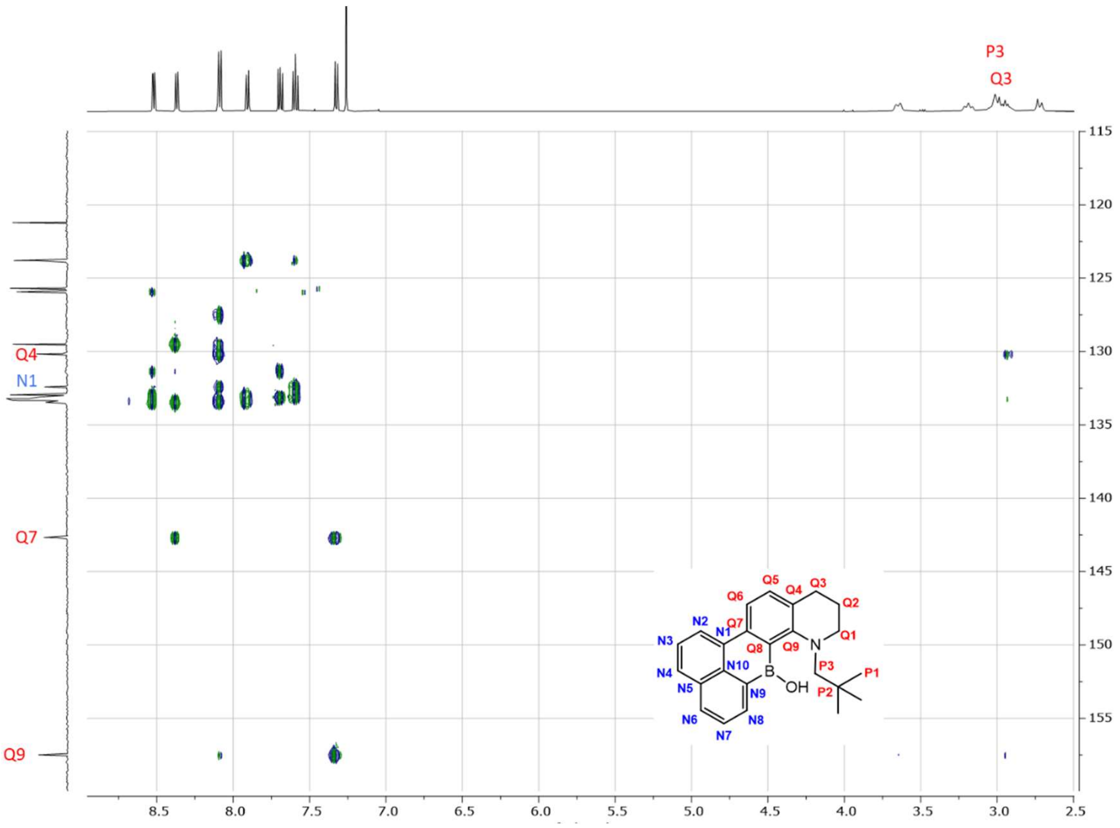

Figure S94. $^{1}H - ^{13}C\{^{1}H\}$ HMBC NMR spectrum of **29-THQ** in $CDCl_3$ at room temperature.

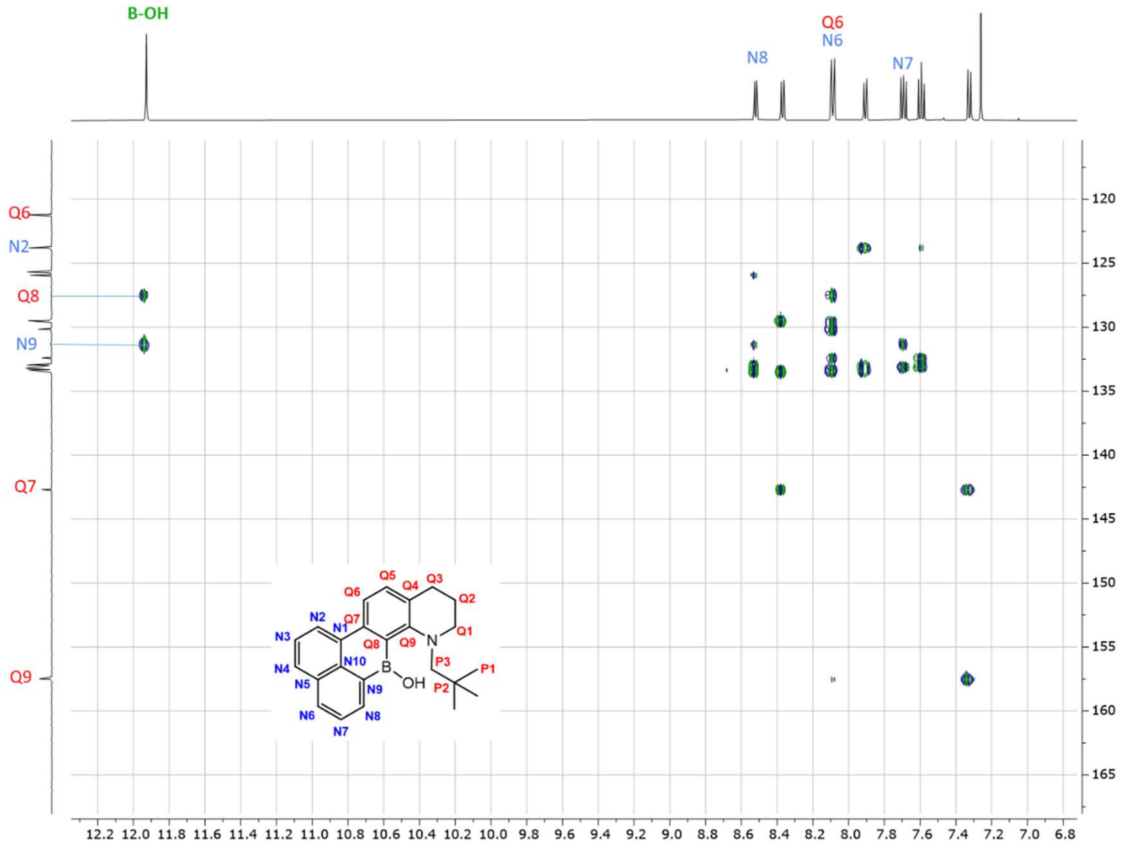

Figure S95. $^{1}H - ^{13}C\{^{1}H\}$ HMBC NMR spectrum of **29-THQ** in $CDCl_3$ at room temperature.

7.5 Synthesis of *N*-pivaloyl-2-methyl-5-naphthylaniline (26-Me)

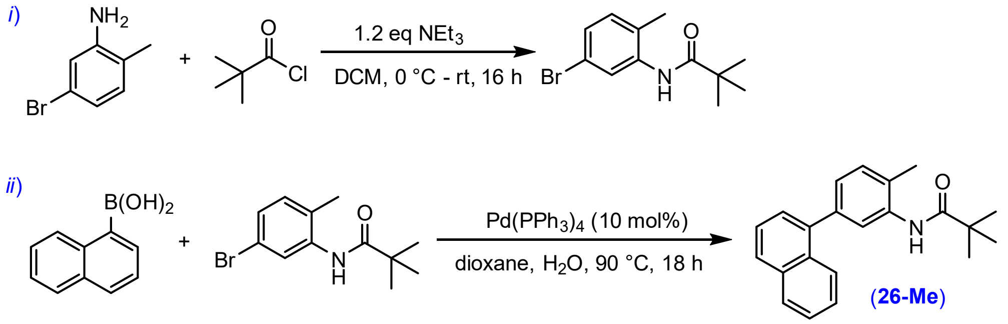

2-methyl-5-bromoaniline (1.86 g, 10 mmol, 1 eq) was loaded into a Schlenk tube and dissolved in dry DCM (30 mL). Dry triethylamine (1.67 mL, 12 mmol, 1.2 eq) was added and the solution was cooled to 0 °C (ice/water bath). Pivaloyl chloride (1.35 mL, 11 mmol, 1.1 eq) was dropwise added and the mixture was stirred at 0 °C for 10 min, and then overnight at room temperature. HCl (1M) was added to the crude mixture and the mixture was extracted with DCM (20 mL x 3), washed with brine (20 mL x 3) and dried over $MgSO_4$. Then, the extract was concentrated and *N*-(2-methyl-5-bromophenyl)pivalamide precipitated with hexane as an off-white solid (2.18 g, 81 % yield).

N-(2-methyl-5-bromophenyl)pivalamide (0.81 g, 3 mmol, 1 eq), naphthalene-1-boronic acid (0.62 g, 3.6 mmol, 1.2 eq) and sodium carbonate (1.14 g, 10.8 mmol, 3.6 eq) were loaded into a Schlenk tube and suspended in a mixture of 1,4-dioxane (20 mL) and water (5 mL). Obtained suspension was degassed under the stream of argon for 30 min. Then tetrakis(triphenylphosphine)palladium(0) (173 mg, 0.15 mmol, 0.05 eq) was added and the mixture was heated at 90 °C for 16 h. Once cooled to room temperature, the reaction mixture was extracted with ethyl acetate and washed with sodium carbonate and brine. The organic fractions were dried over $MgSO_4$ and all volatiles were removed under vacuum affording **26-Me** as an off-white solid (0.77 g, 81 % yield).

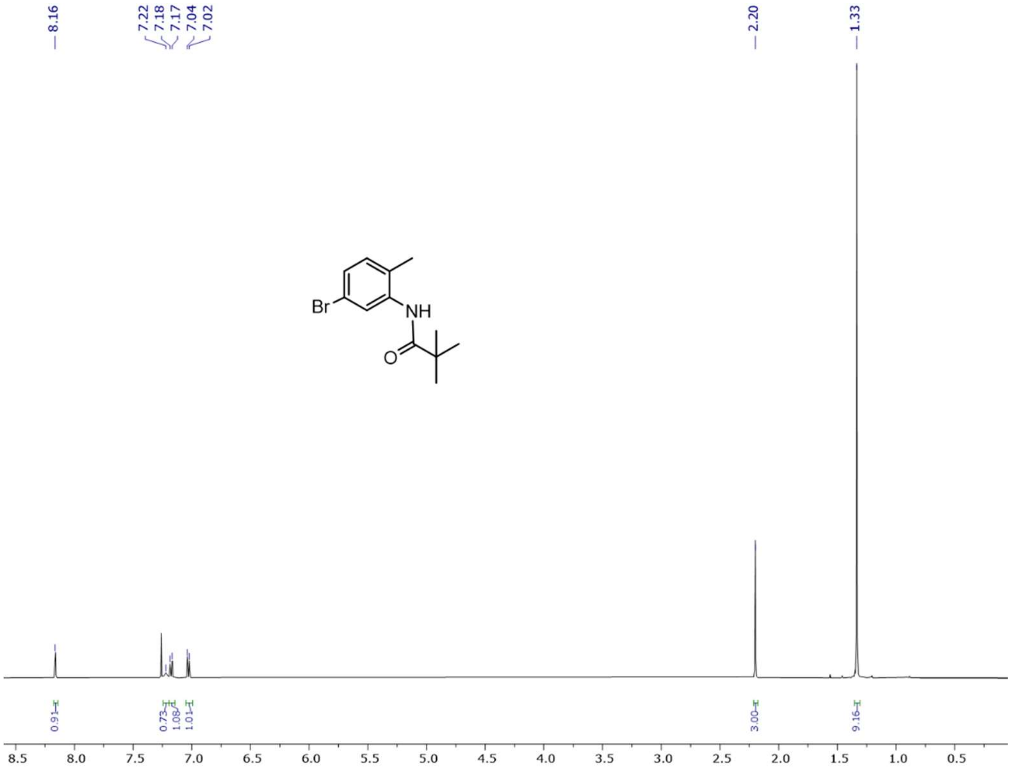

Figure S96. ^{1}H NMR spectrum of *N*-(2-methyl-5-bromophenyl)pivalamide in $CDCl_3$ at room temperature.

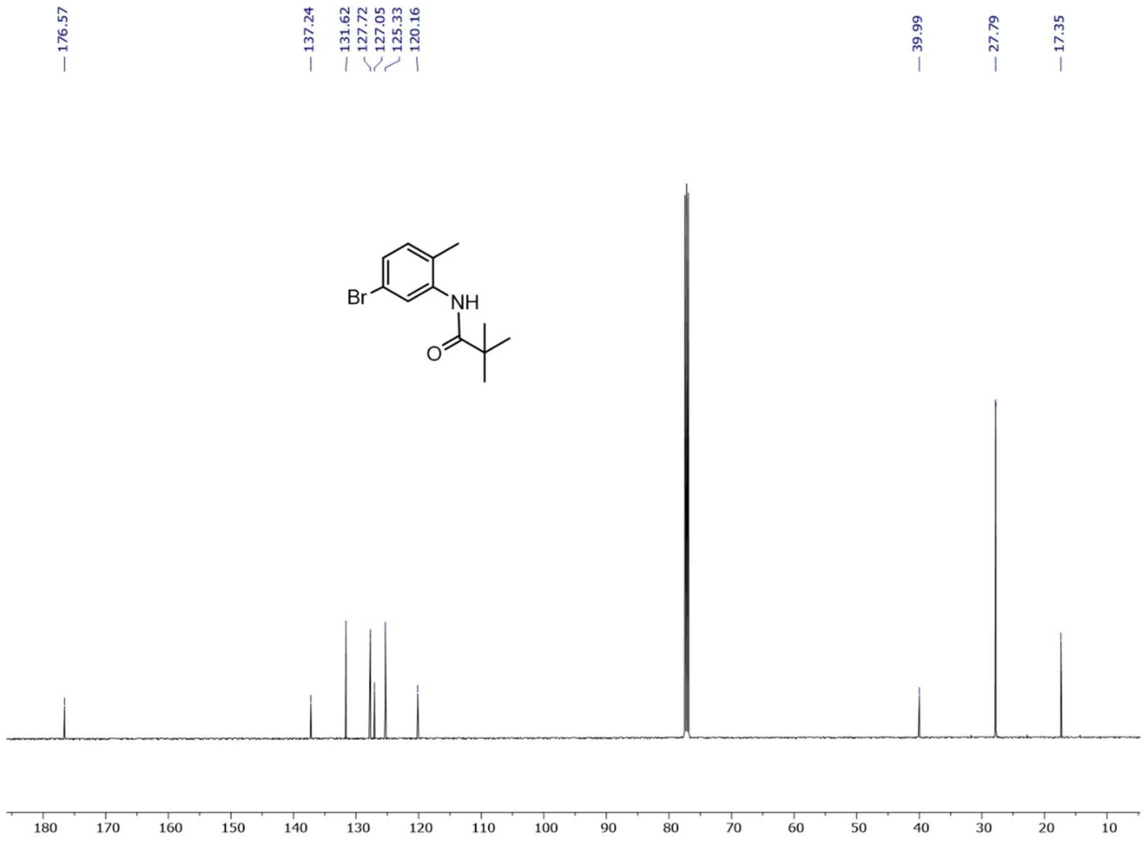

Figure S97. $^{13}C\{^1H\}$ NMR spectrum of N-(2-methyl-5-bromophenyl)pivalamide in $CDCl_3$ at room temperature.

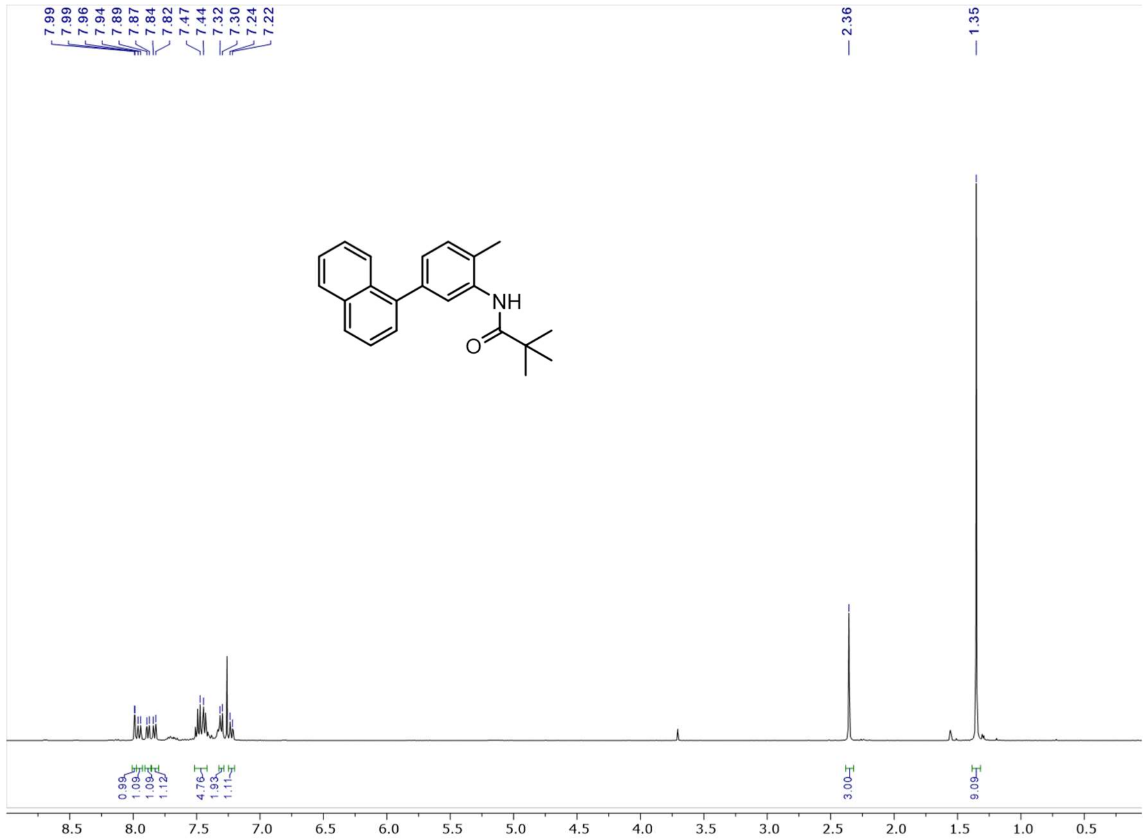

Figure S98. ^1H NMR spectrum of crude **26-Me** in $CDCl_3$ at room temperature.

7.6 Directed borylation of *N*-pivaloyl-2-methyl-5-naphthylaniline (27-Me)

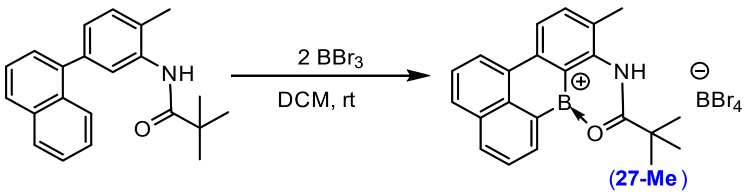

BBr_3 1M in DCM (3.6 mL, 3.6 mmol, 2 eq) was added to a stirred solution of N-pivaloyl-2-methyl-5-naphthylaniline (0.57 g, 1.8 mmol, 1 eq) in dry DCM (5 mL). The reaction mixture was stirred for 10 min and then left to stand at room temperature overnight affording light yellow solid of **27-Me** (924 mg, 78 % yield). Anal. Calc. for $C_{22}H_{21}B_2Br_4NO$: C, 40.24; H, 3.22; N, 2.13; found: C, 41.18; H, 3.16; N, 2.08.

^{1}H NMR (500 MHz, CD_2Cl_2, 298 K): δ 8.67 (dd, *J*=7.5, 1.2 Hz, 1H, CH_{N2}), 8.62 (dd, *J*=6.8, 1.4 Hz, 1H, CH_{N8}), 8.50 (d, *J*=8.2 Hz, 1H, CH_{A4}), 8.36 (dd, *J*=8.1, 1.4 Hz, 1H, CH_{N6}), 8.16 (dd, *J*=8.1, 1.1 Hz, 1H, CH_{N4}), 7.90 (dd, *J*=8.3, 0.9 Hz, 1H, CH_{A3}), 7.85 (dd, *J*=8.1, 6.8 Hz, 1H, CH_{N7}), 7.79 (t, *J*=7.8 Hz, 1H, CH_{N3}), 2.73 (s, 3H, A7-CH_3), 1.82 (s, 9H, P1-CH_3) ppm. **^{13}C{^{1}H} NMR** (126 MHz, CD_2Cl_2, 298 K): δ 180.3 (C_{P3}), 140.8 (C_{A5}), 137.7 (C_{A3}), 136.9 (C_{N6}), 135.6 (C_{N8}), 134.2(C_{N1}), 134.0 (C_{N5}), 133.5 (C_{N10}), 132.9 (C_{N4}), 129.8(C_{A2}), 126.9 (C_{N3}), 126.8 (C_{N7}), 126.6 (C_{N2}), 125.5 (C_{A4}), 124.8 (C_{A1}), 40.6 (P2-$C(CH_3)_3$), 27.1(P1-$C(CH_3)_3$), 16.1 (C_{A7}), ppm. Resonances for C_{N9} and C_{A6} were not detected in ^{13}C{^{1}H} due to quadrupolar effects.**^{11}B NMR** (160 MHz, CD_2Cl_2, 298 K): δ 36.7, -24.44 ppm.

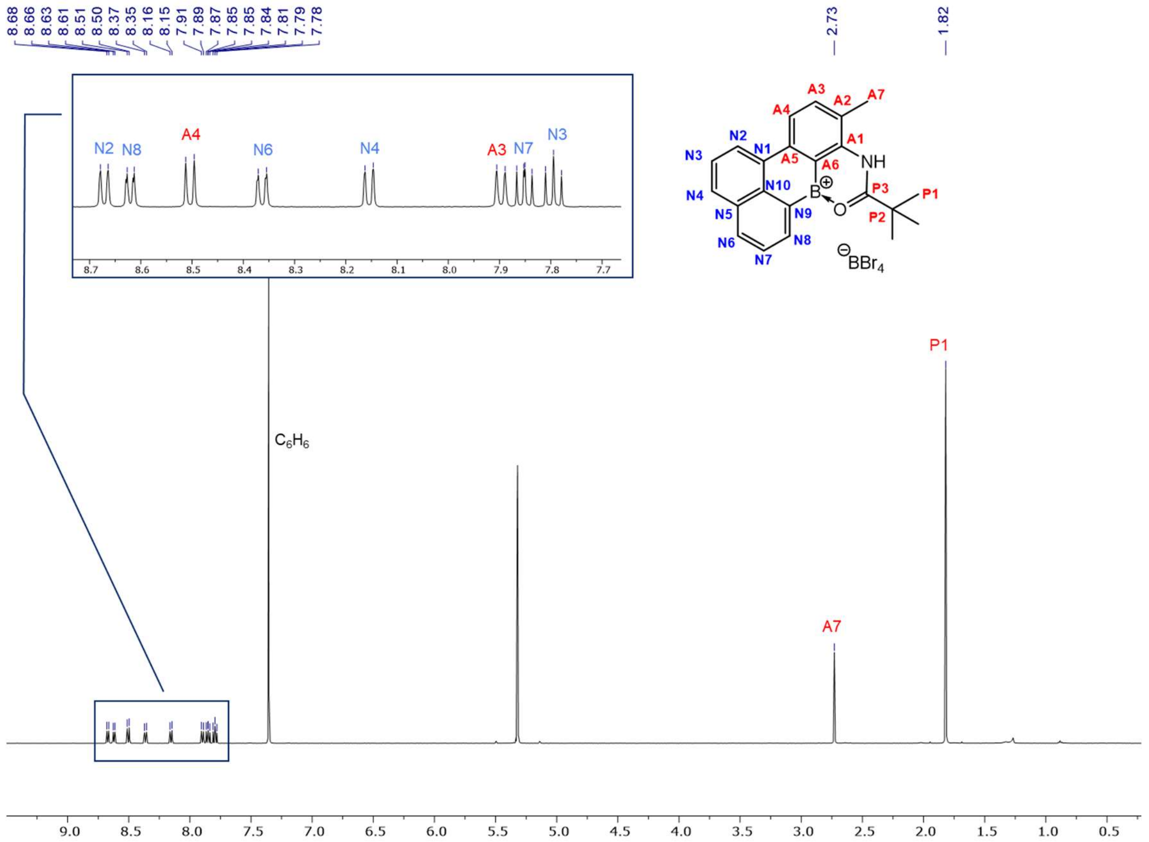

Figure S99. ^{1}H NMR spectrum of **27-Me** in CD_2Cl_2 at room temperature. Residual benzene is from washing of **27-Me** during isolation.

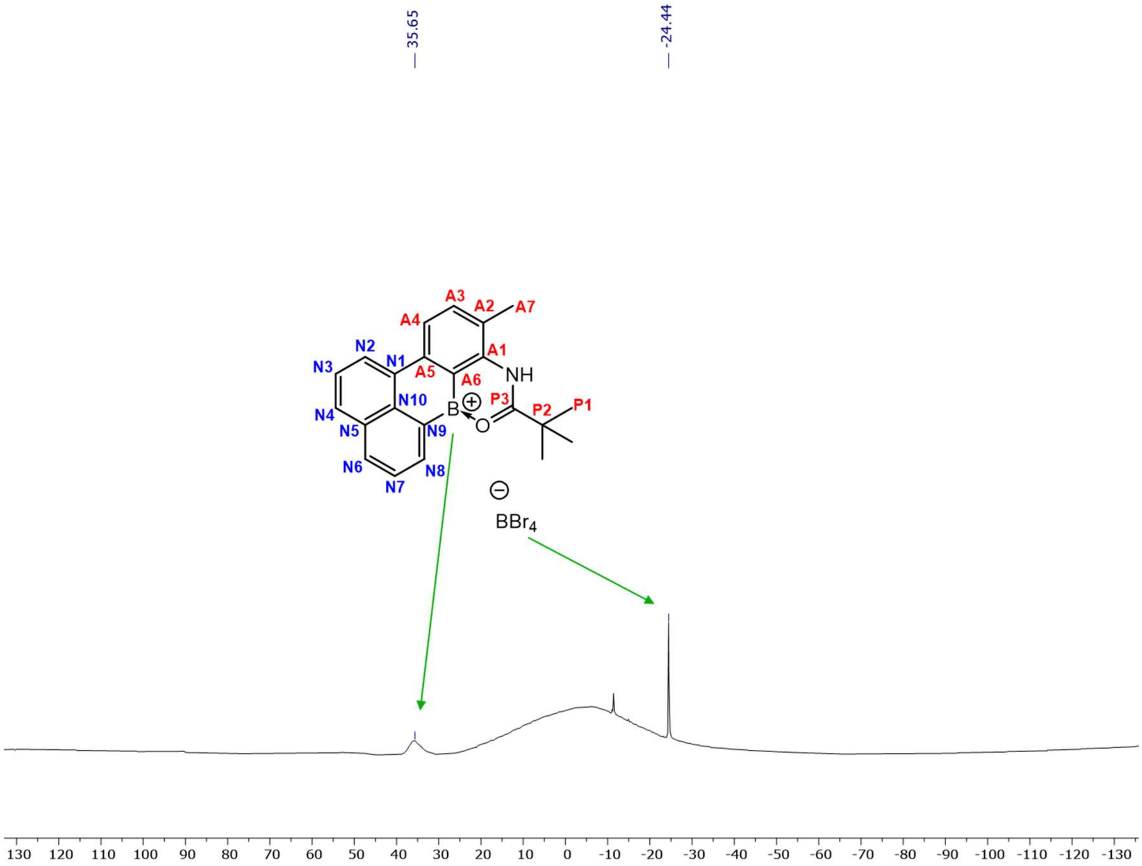

Figure S100. ^{11}B NMR spectrum of **27-Me** in CD_2Cl_2 at room temperature.

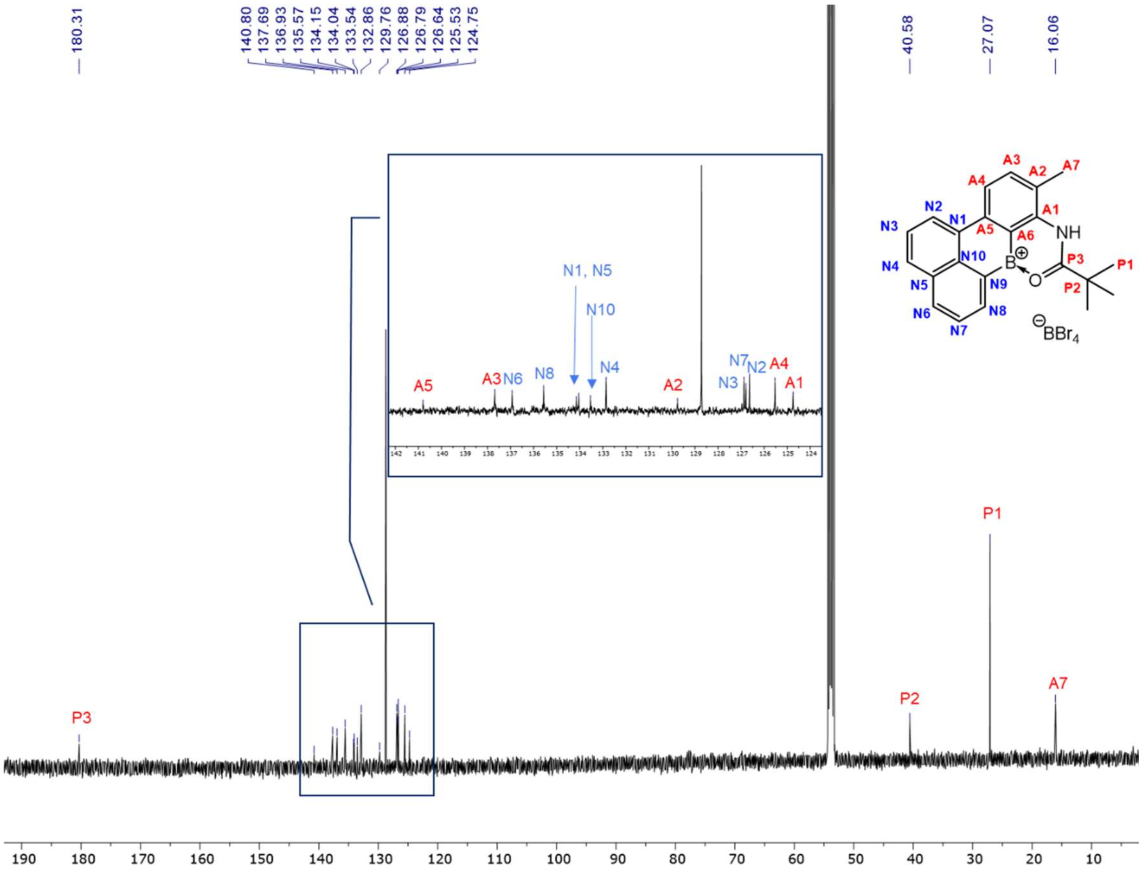

Figure S101. $^{13}C\{^1H\}$ NMR spectrum of **27-Me** in CD_2Cl_2 at room temperature.

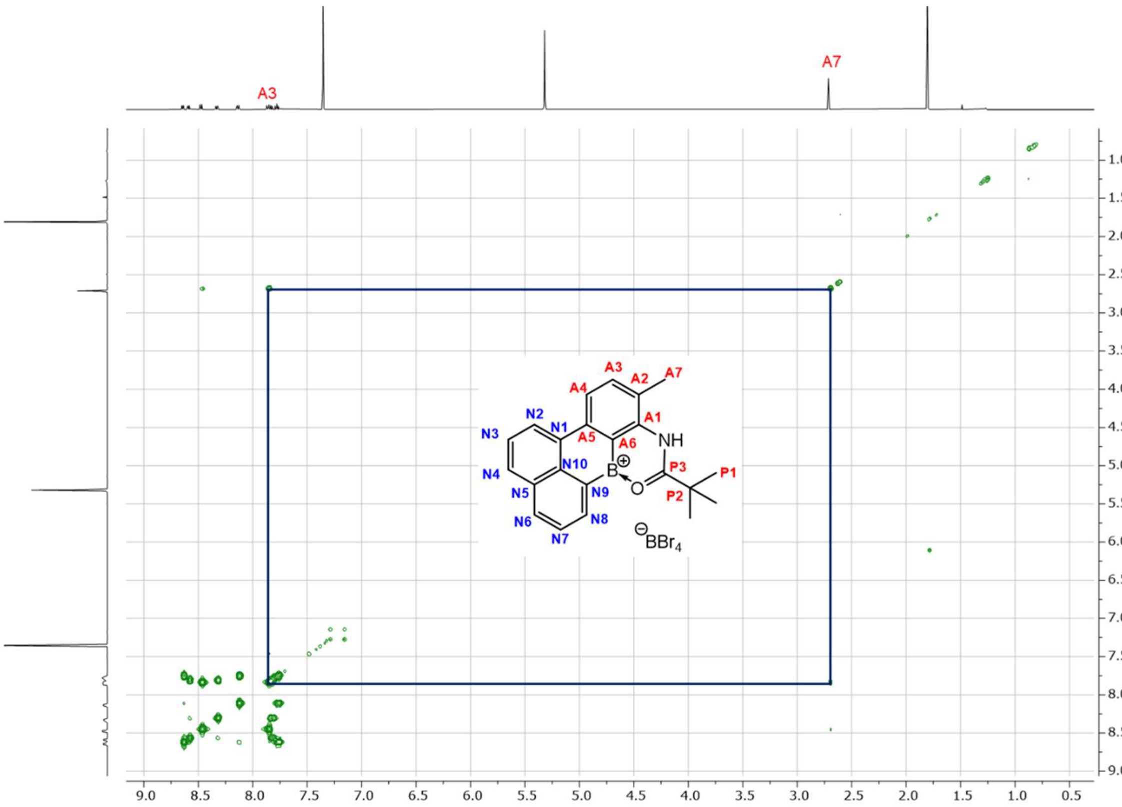

Figure S102. ^1H – ^1H COSY NMR spectrum of **27-Me** in CD_2Cl_2 at room temperature.

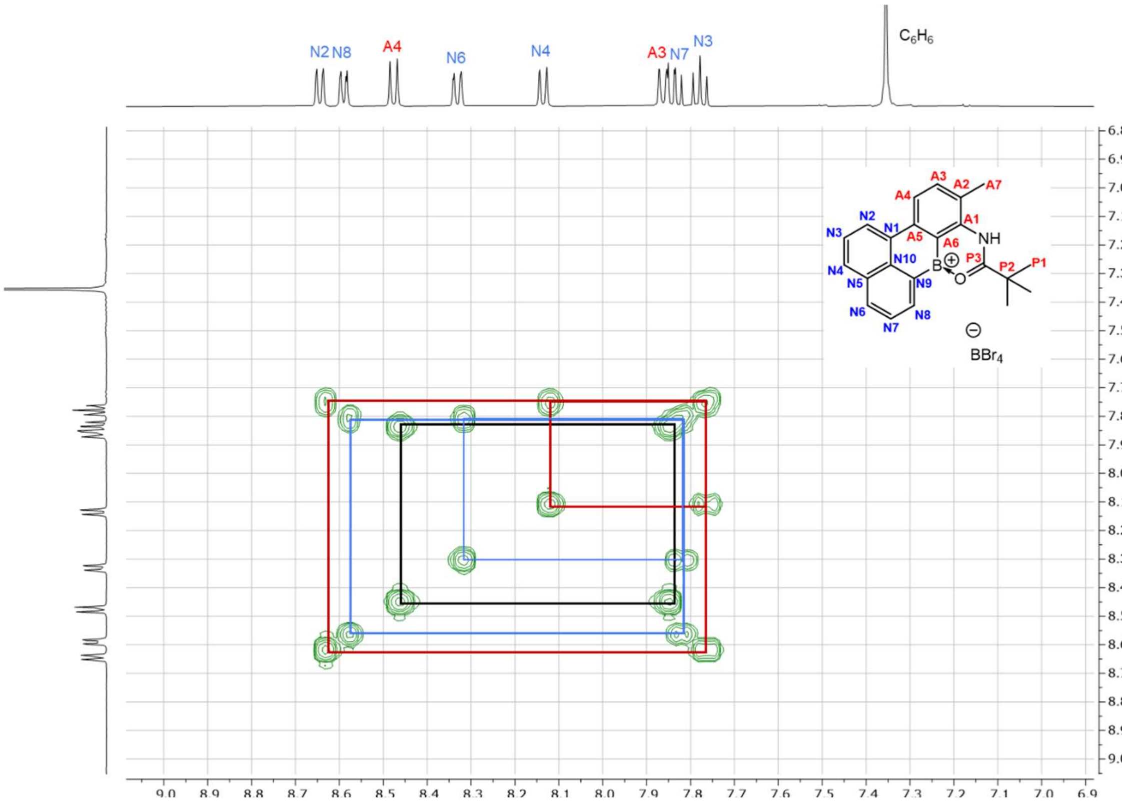

Figure S103. ^1H – ^1H COSY NMR spectrum of **27-Me** in CD_2Cl_2 at room temperature.

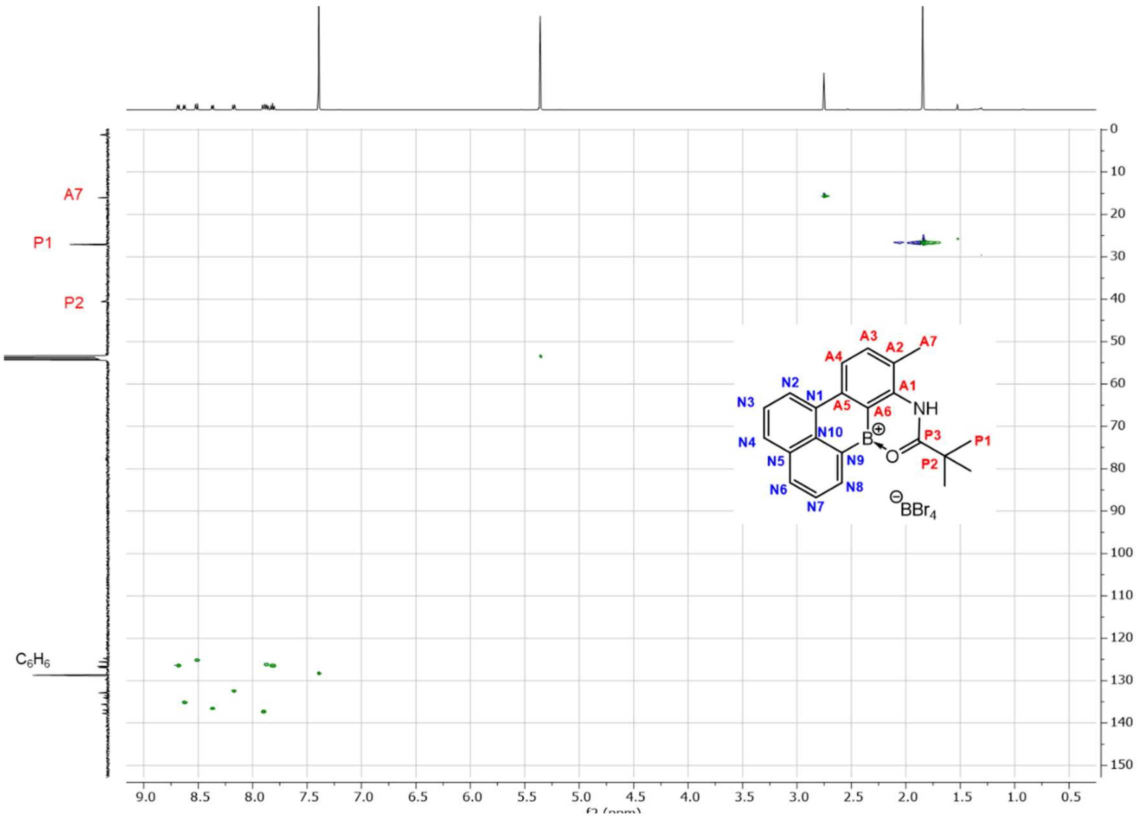

Figure S104. ^1H – $^{13}C\{^1H\}$ HSQC NMR spectrum of **27-Me** in CD_2Cl_2 at room temperature.

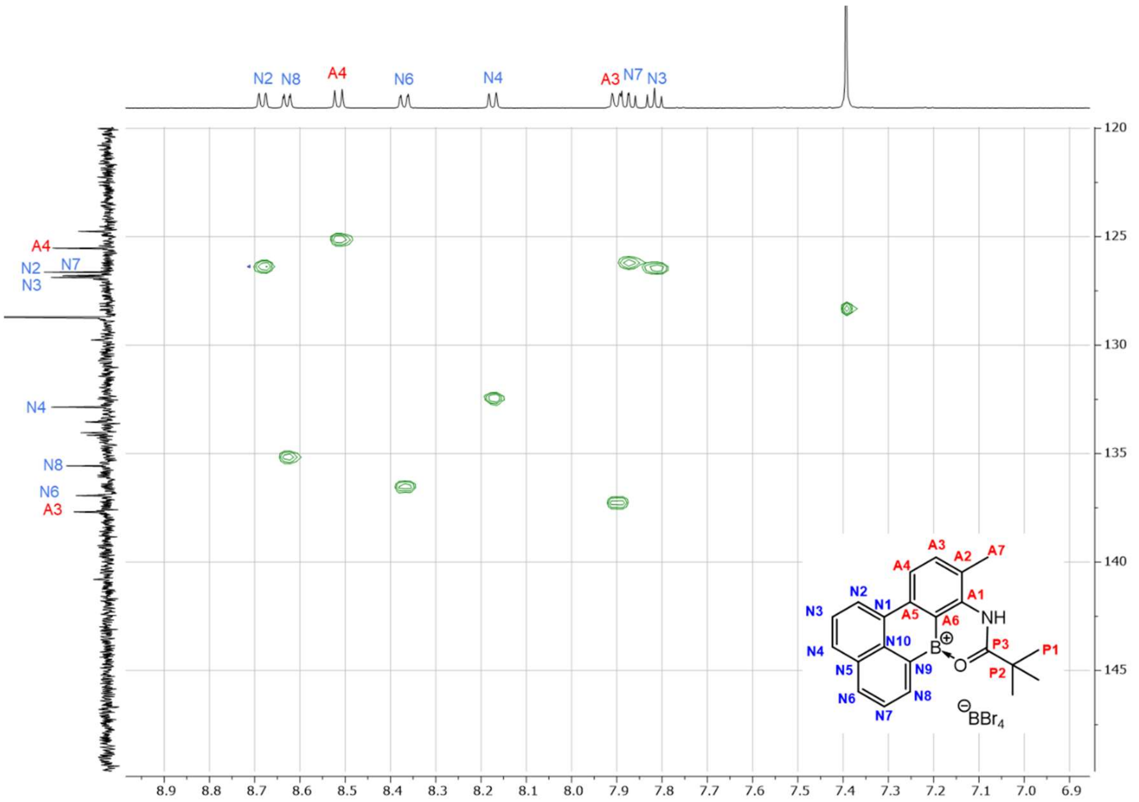

Figure S105. ^1H – $^{13}C\{^1H\}$ HSQC NMR spectrum of **27-Me** in CD_2Cl_2 at room temperature.

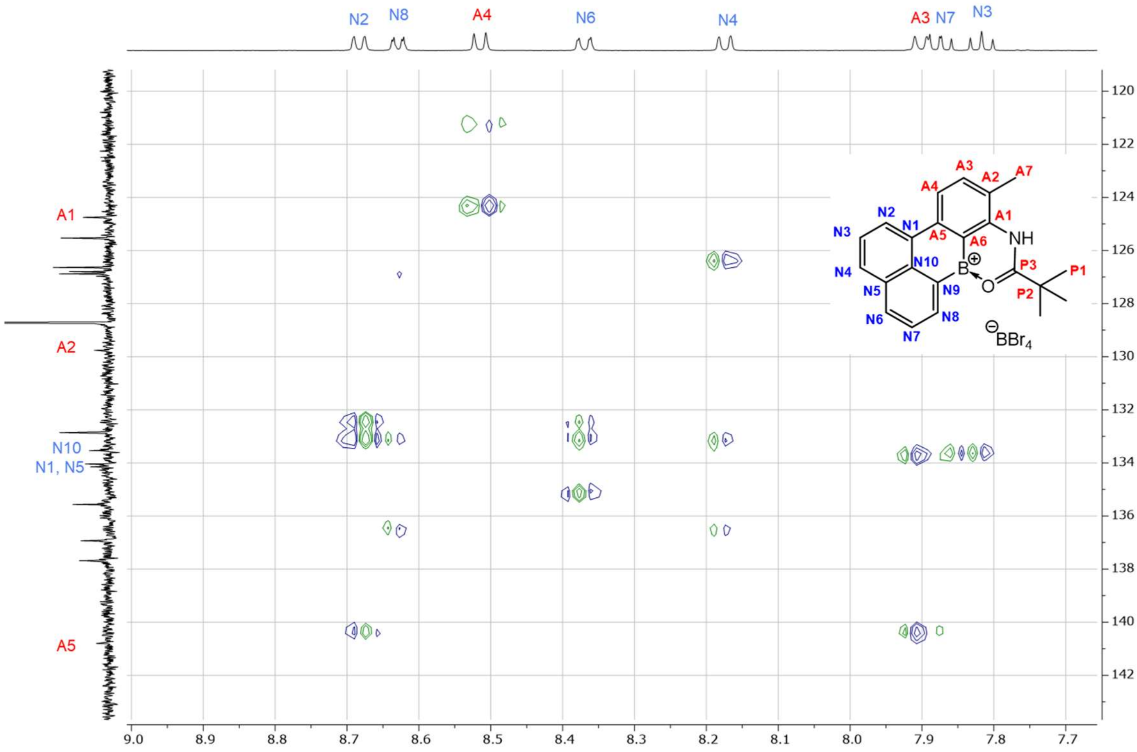

Figure S106. ^{1}H – $^{13}C\{^{1}H\}$ HMBC NMR spectrum of **27-Me** in CD_2Cl_2 at room temperature.

7.7 Reduction of 27-Me and isolation of 29-Me

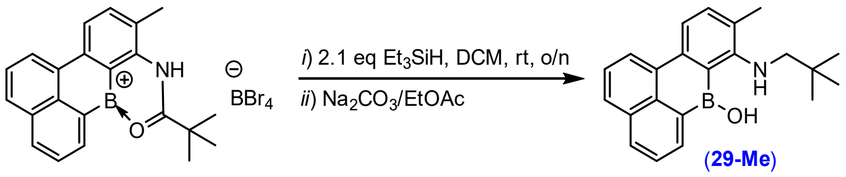

To a suspension of **27-Me** (500 mg, 0.76 mmol, 1 eq) in DCM (5 mL) triethylsilane (0.25 mL, 1.6 mmol, 2.1 eq) was added. After a couple of minutes stirring a yellow solution is formed which was further stirred overnight (20 h) again forming a suspension. All volatiles were removed under vacuum, and obtained yellow solid washed with DCM (5 mL x 2). Finally, benzene was introduced (5 mL) but no precipitation occurred. The reaction mixture was extracted with ethyl acetate and aqueous solution of sodium carbonate. Organic layer was washed with brine and dried over magnesium sulfate. Obtained bright yellow solution was evaporated to dryness and upon addition of pentane (5 mL) yellow solid of **29-Me** was precipitated (191 mg, 76 % yield).

Anal. Calc. for $C_{22}H_{24}BNO$: C, 80.26; H, 7.35; N, 4.25; found: C, 79.82; H, 7.49; N, 4.14. **[Acc. Mass]** Calculated for $[M+H]^+$: 330.20237, found $[M+H]^+$ 330.2022.

^{1}H NMR (500 MHz, $CDCl_3$, 298 K): δ 11.95(s, 1H, BO*H*), 8.54 (dd, *J*=6.8, 1.4 Hz, 1 H,CH_{N8}), 8.40 (dd, *J*=7.7, 1.1 Hz, 1 H, CH_{N4}), 8.13 (d, *J*=8.2 Hz, 1 H, CH_{A4}), 8.10 (dd, *J*=8.1, 1.4 Hz, 1 H, CH_{N6}), 7.91 (dd, *J*=8.1, 1.1 Hz, 1 H, CH_{N2}), 7.69 (dd, *J*=8.1, 6.7Hz, 1 H, CH_{N7}), 7.60 (t, *J*=7.8 Hz, 1 H, CH_{N3}), 7.44 (dd, *J*=8.2, 0.8 Hz, 1 H, CH_{A3}), 3.36 (t, *J*=7.2 Hz, 1 H, N*H*), 2.73 (d, *J*=7.2 Hz, 2 H, CH_2), 2.38 (s, 3 H, Ar-CH_3), 1.17 (s, 9 H, CH_3) ppm.**^{13}C{^{1}H} NMR** (126 MHz, $CDCl_3$, 298 K): δ 152.8 (C_{A1}), 143.1 (C_{A5}), 134.3 (C_{A3}), 133.6 (C_{N10}), 133.4 (C_{N8}), 133.2 (C_{N5}), 133.0 (C_{N6}), 132.1 (C_{N1}), 131.2 (C_{N9}), 130.5 (C_{A2}), 127.7 (C_{A6}), 129.6 (C_{N2}), 125.9 (C_{N7}), 125.6 (C_{N3}), 123.7 (C_{N4}), 121.5 (C_{A4}), 64.8 (CH_2), 31.8 ($C(CH_3)_3$), 27.8 ($C(CH_3)_3$),16.8 (Ar-CH_3) ppm. **^{11}B NMR** (160 MHz, $CDCl_3$, 298 K): δ 41.08 ppm.

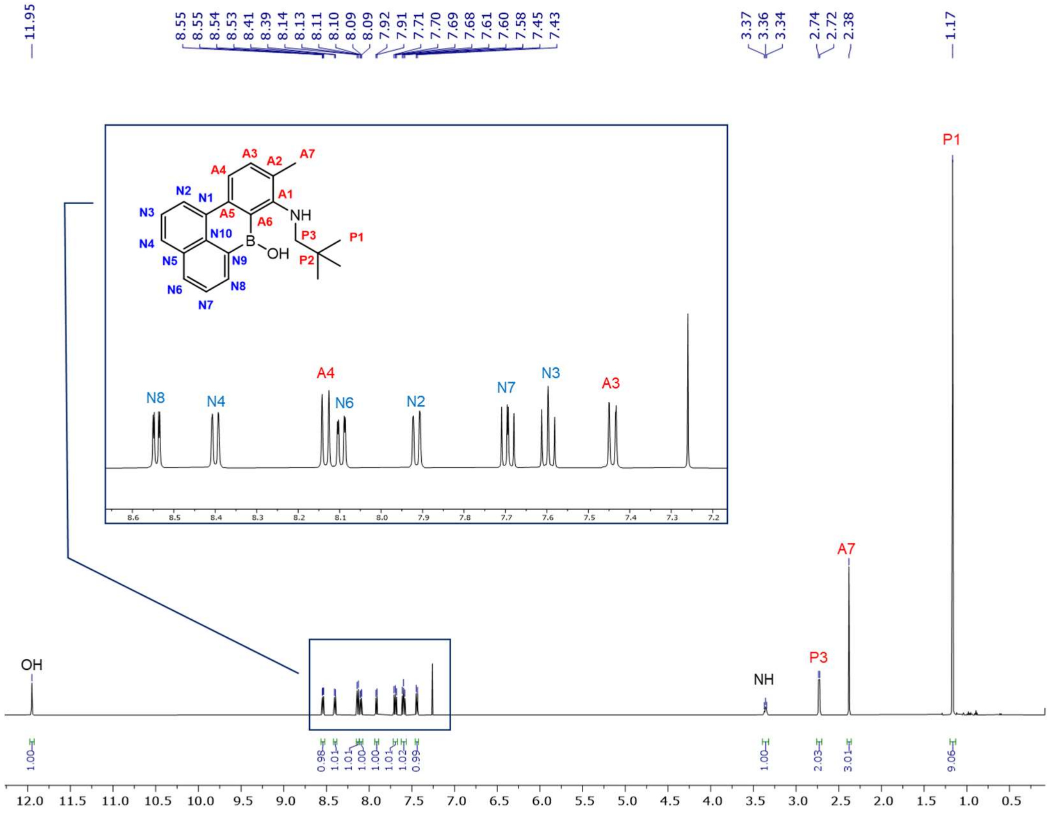

Figure S107. ^{1}H NMR spectrum of **29-Me** in $CDCl_3$ at room temperature.

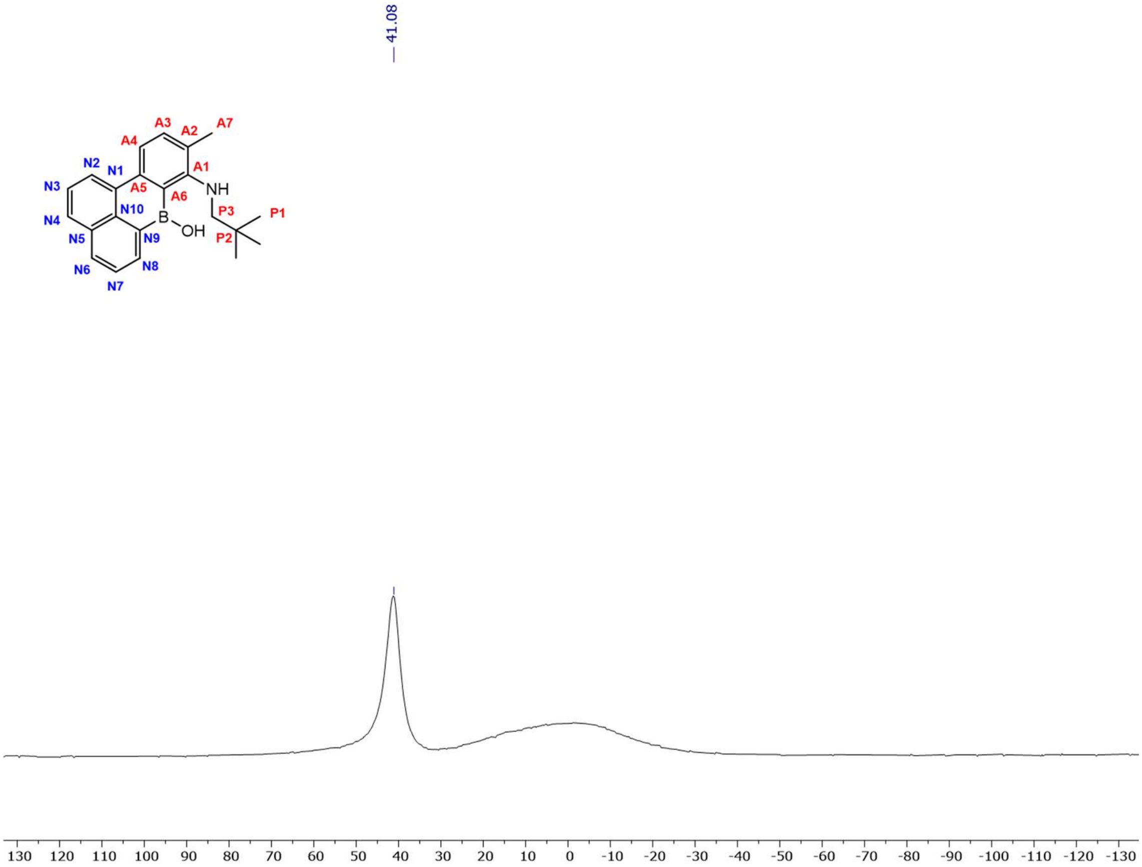

Figure S108. ^{11}B NMR spectrum of **29-Me** in $CDCl_3$ at room temperature.

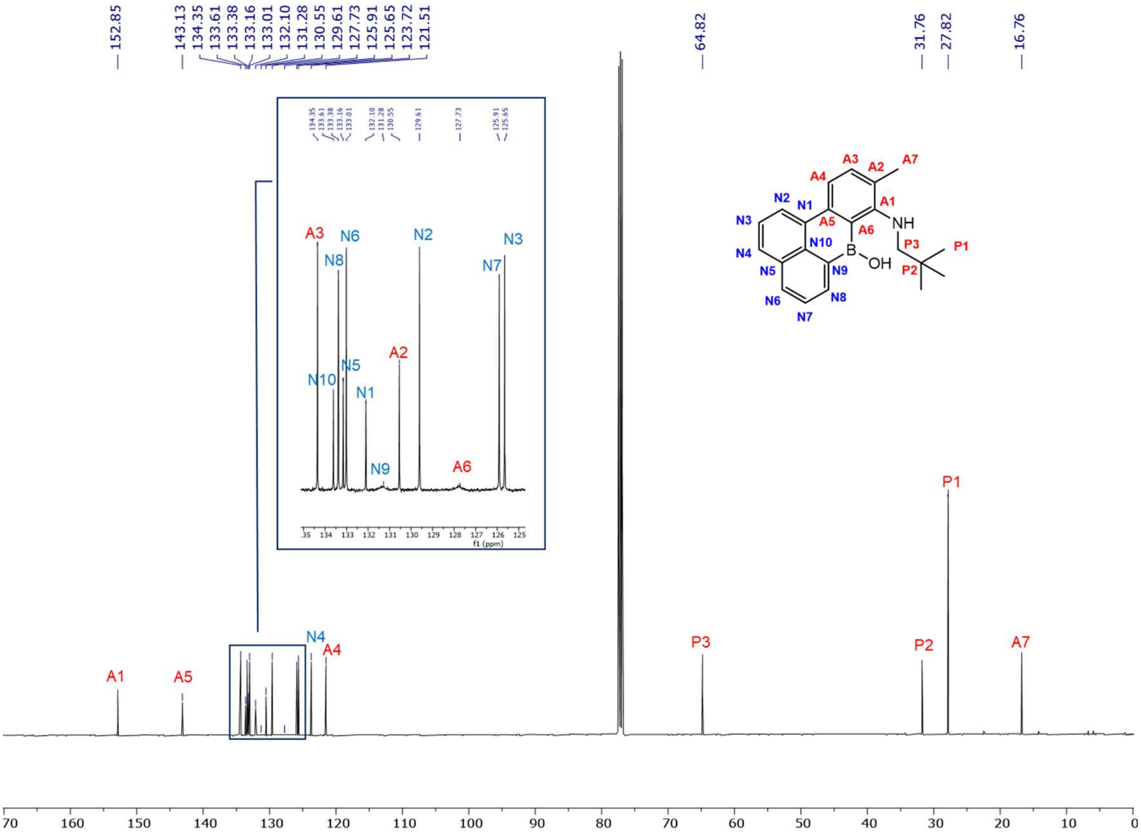

Figure S109. $^{13}C\{^{1}H\}$ NMR spectrum of **29-Me** in $CDCl_3$ at room temperature.

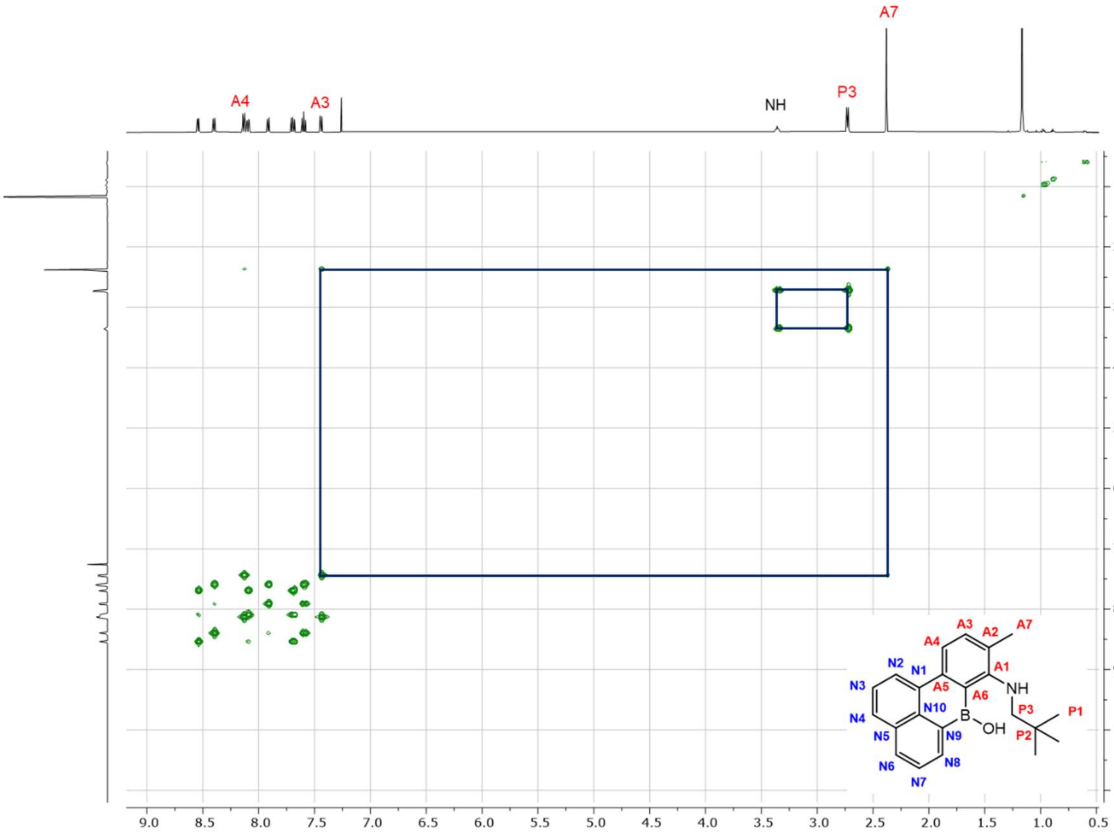

Figure S110. $^1H - ^1H$ COSY NMR spectrum of **29-Me** in $CDCl_3$ at room temperature.

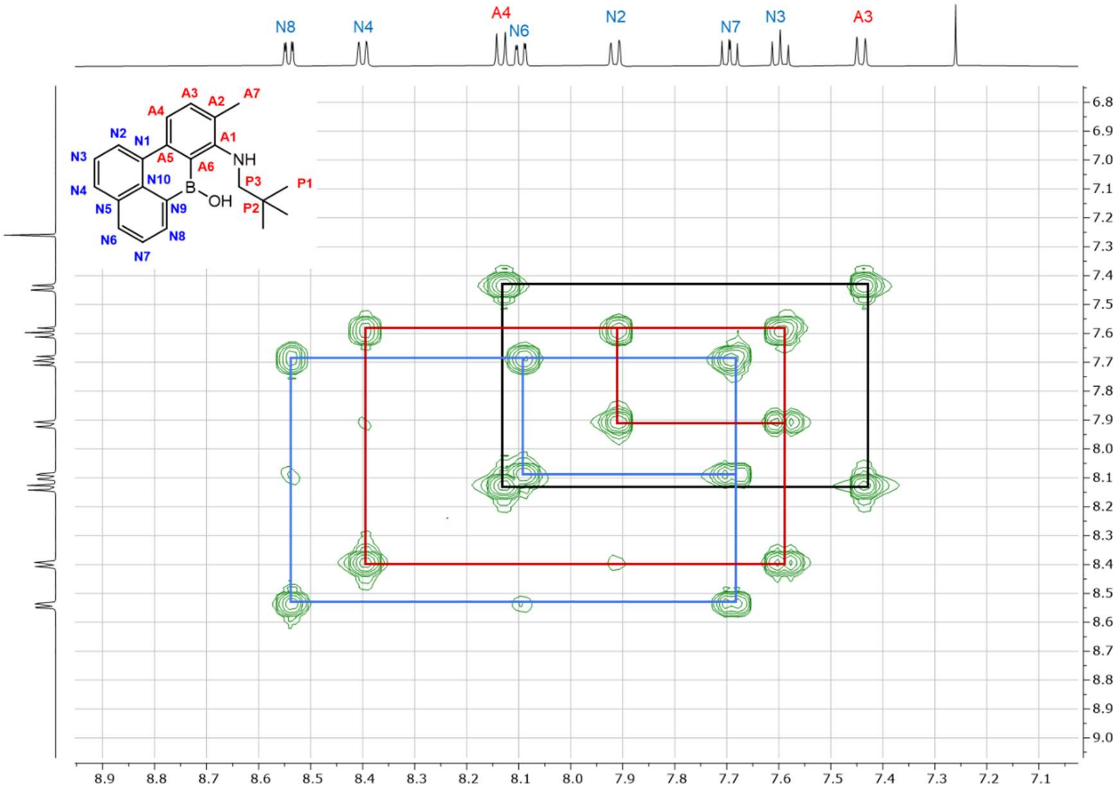

Figure S111. $^1H - ^1H$ COSY NMR spectrum of **29-Me** in $CDCl_3$ at room temperature.

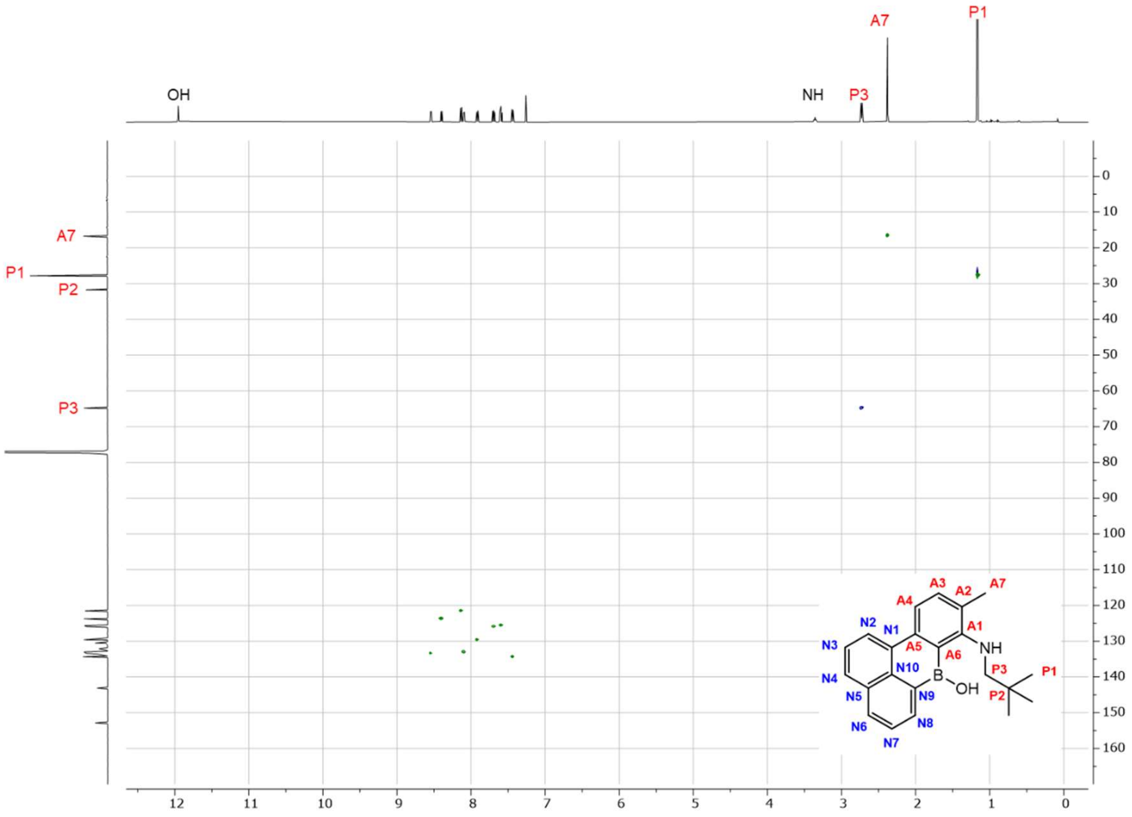

Figure S112. ^1H – $^{13}C\{^1H\}$ HSQC NMR spectrum **29-Me** in $CDCl_3$ at room temperature.

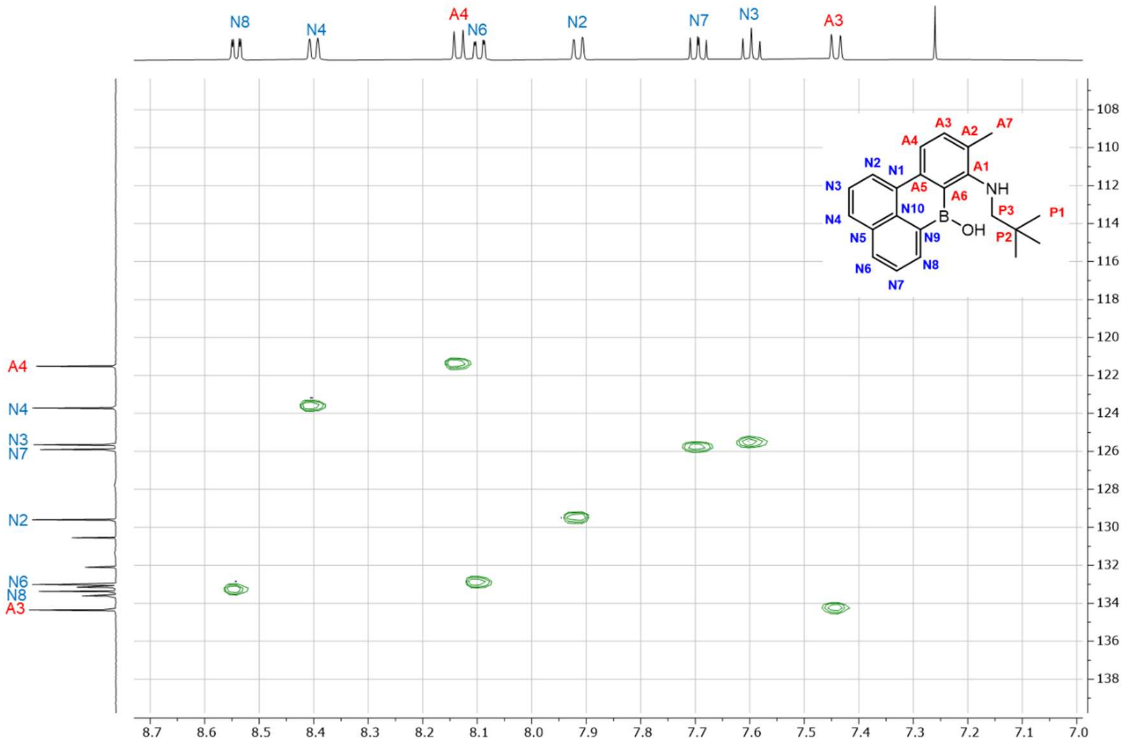

Figure S113. ^1H – $^{13}C\{^1H\}$ HSQC NMR spectrum **29-Me** in $CDCl_3$ at room temperature.

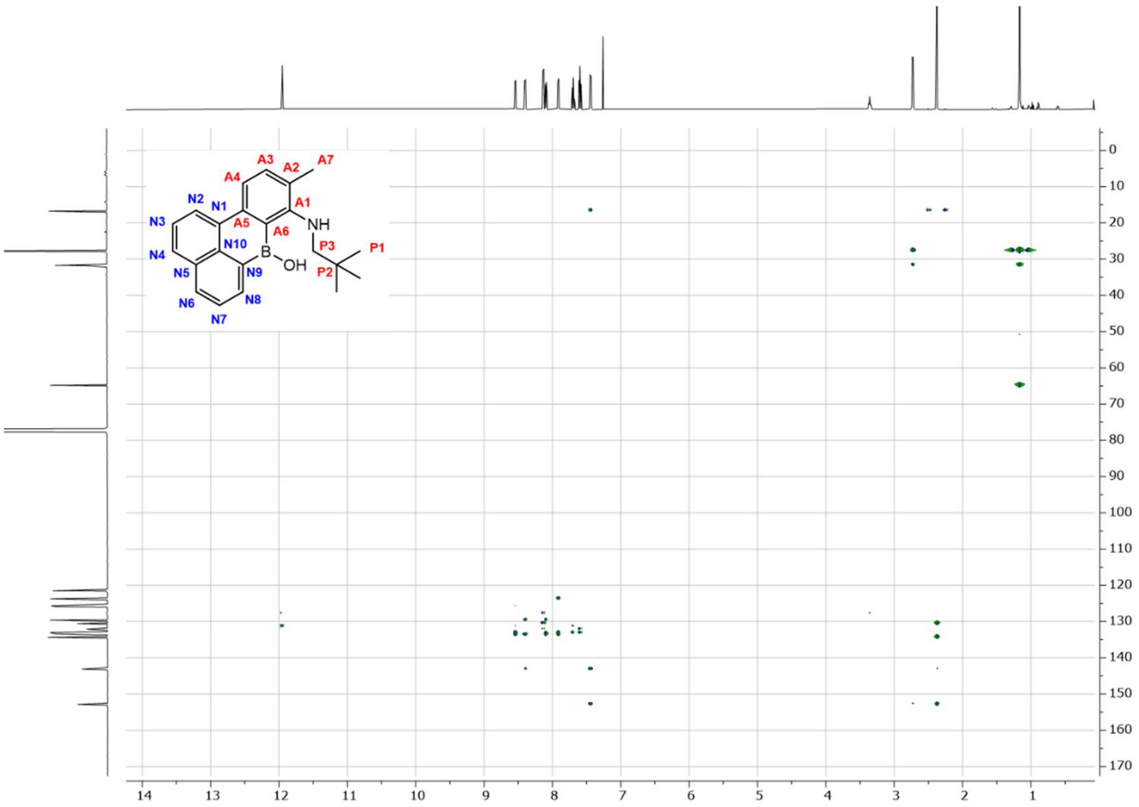

Figure S114. ^{1}H – $^{13}C\{^{1}H\}$ HMBC NMR spectrum of **29-Me** in $CDCl_3$ at room temperature.

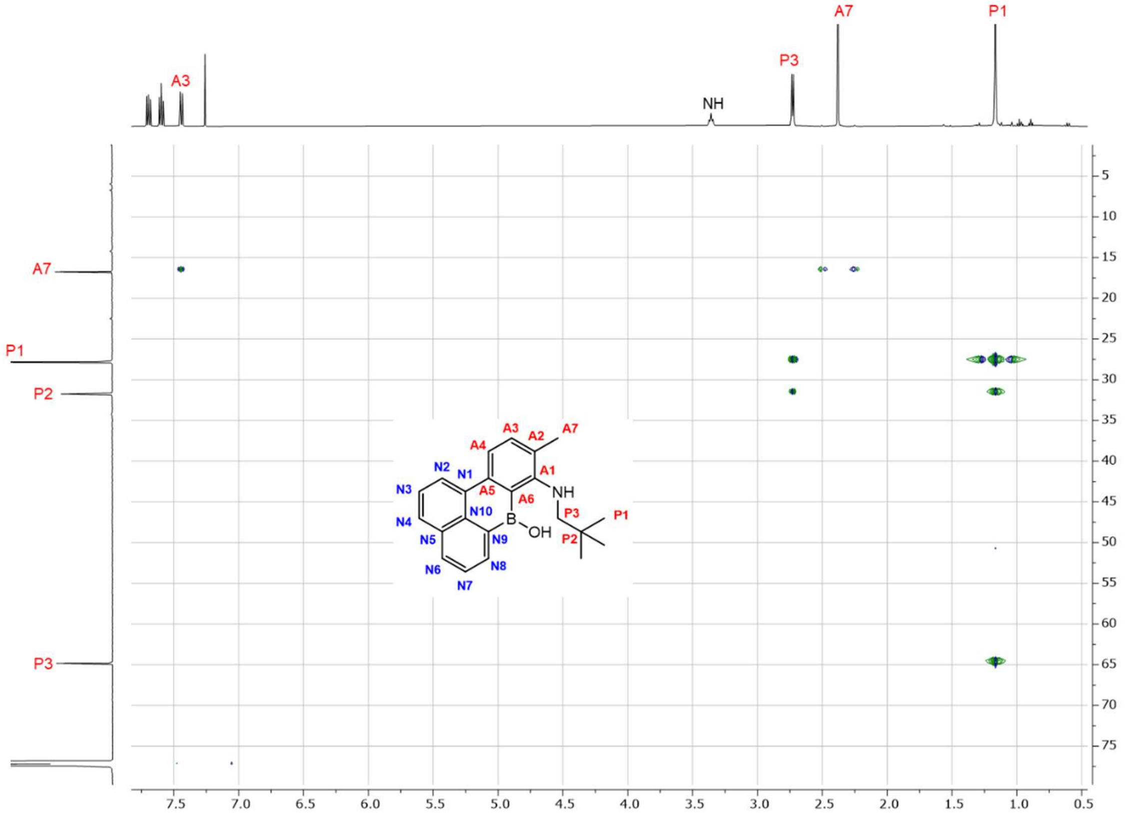

Figure S115. ^{1}H – $^{13}C\{^{1}H\}$ HMBC NMR spectrum of **29-Me** in $CDCl_3$ at room temperature.

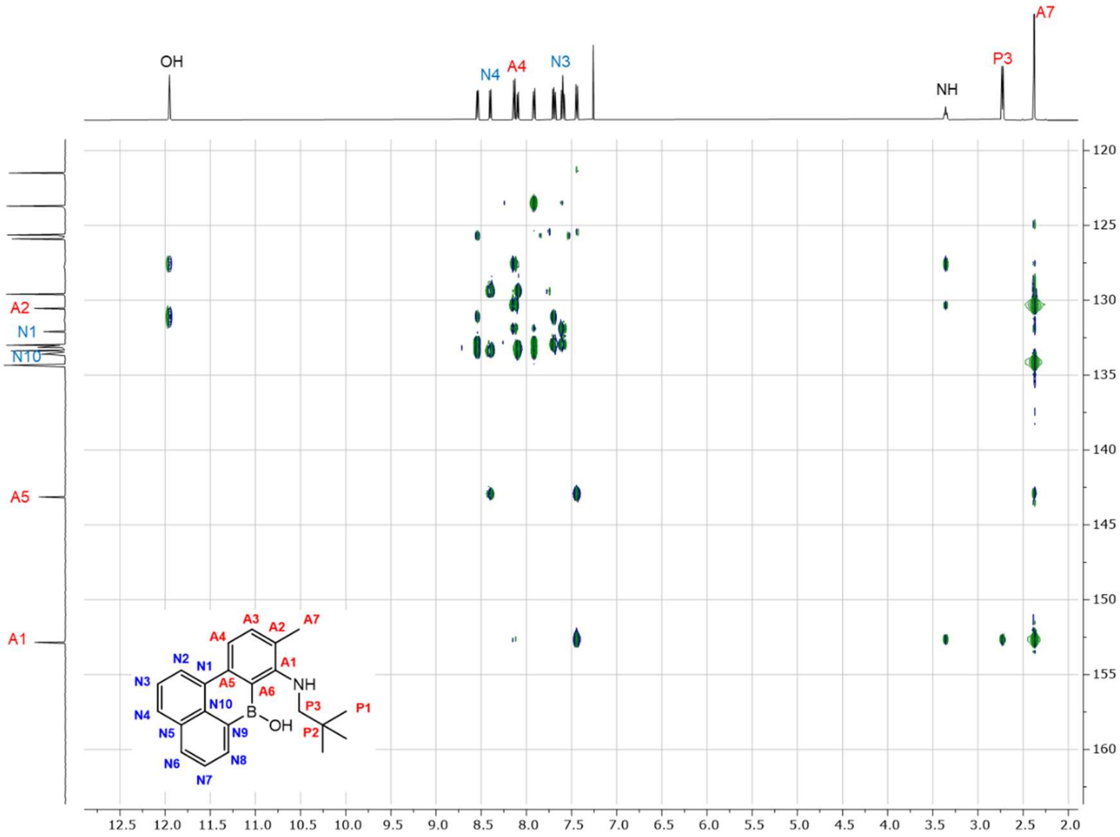

Figure S116. ^1H – $^{13}C\{^1H\}$ HMBC NMR spectrum of **29-Me** in $CDCl_3$ at room temperature.

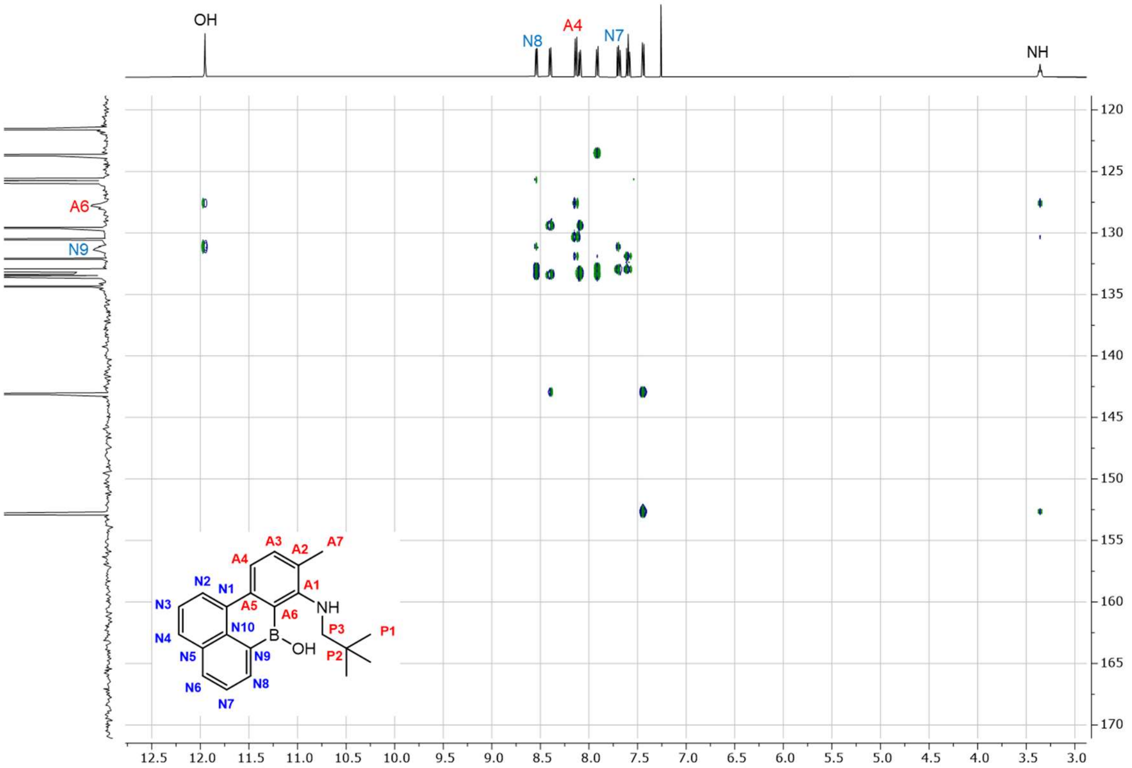

Figure S117. ^1H – $^{13}C\{^1H\}$ HMBC NMR spectrum of **29-Me** in $CDCl_3$ at room temperature.

8 X-Ray Crystallography

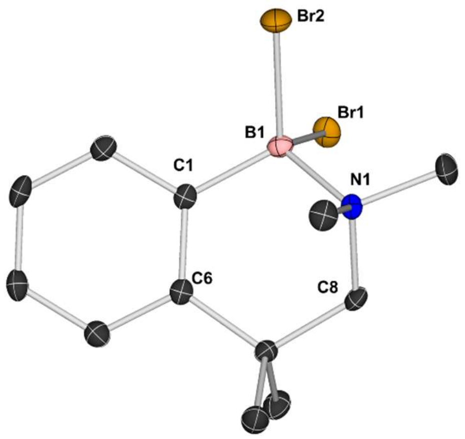

Figure S118. ORTEP representation of **5** with thermal ellipsoids at 50% probability level. All hydrogen atoms have been omitted for clarity.

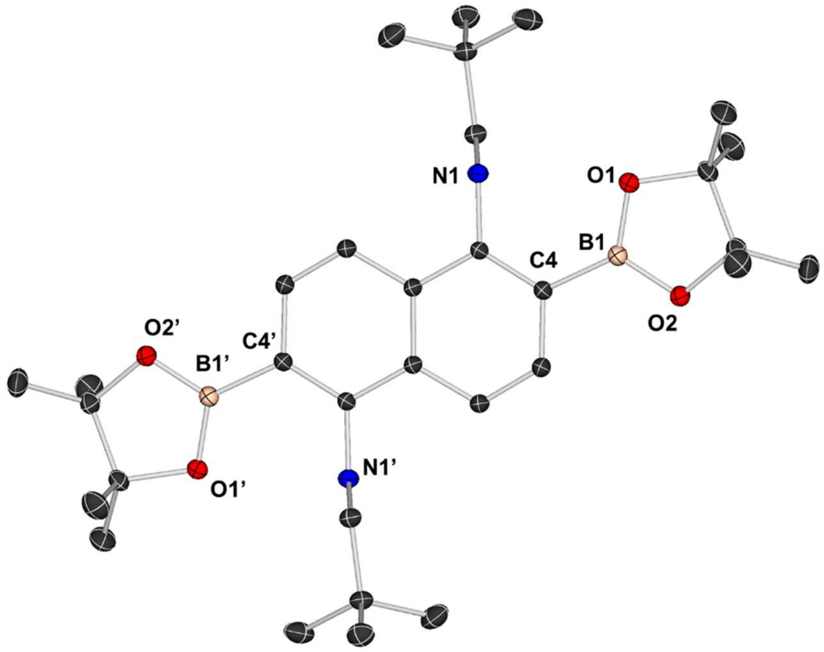

Figure S119. ORTEP representation of **25** with thermal ellipsoids at 50% probability level. All hydrogen atoms have been omitted for clarity. Symmetry operator: -x, -y, -z.

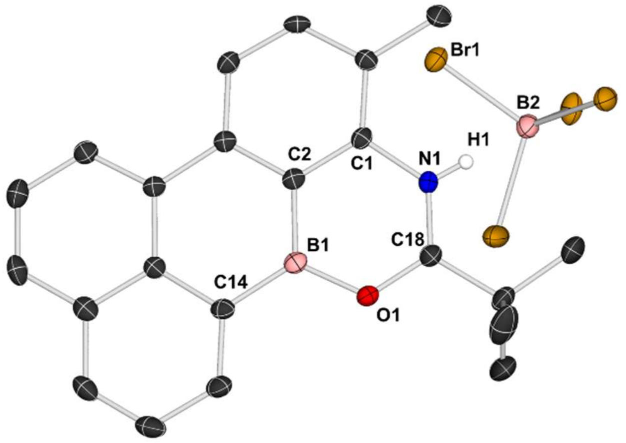

Figure S120. ORTEP representation of **27-Me** with thermal ellipsoids at 50% probability level. All hydrogen atoms, except NH, and crystallising molecule of DCM have been omitted for clarity.

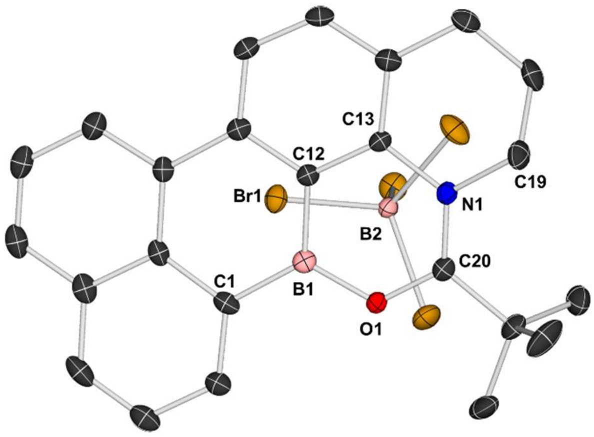

Figure S121. ORTEP representation of **27-THQ** with thermal ellipsoids at 30% probability level. All hydrogen atoms, and disordered crystallising molecule of DCM have been omitted for clarity.

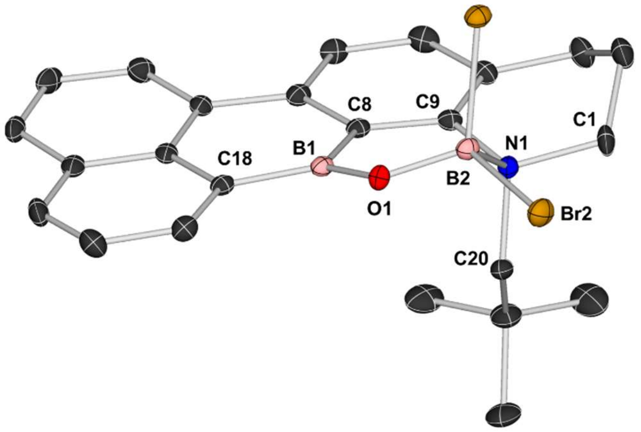

Figure S122. ORTEP representation of **28-THQ** with thermal ellipsoids at 50% probability level. All hydrogen atoms, and crystallising benzene molecule have been omitted for clarity.

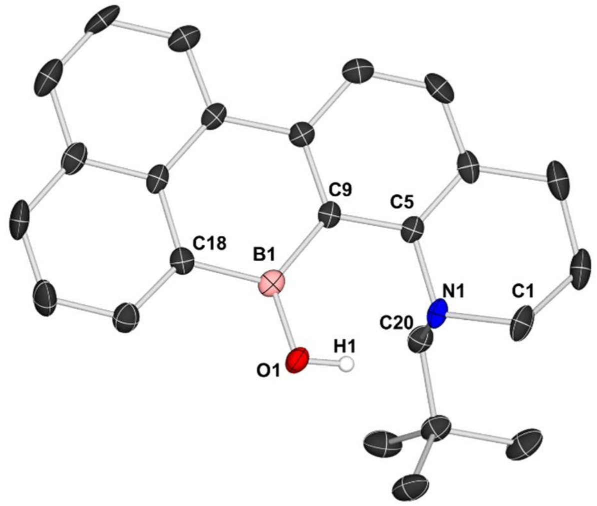

Figure S123 ORTEP representation of **29-THQ** with thermal ellipsoids at 50% probability level. Asymmetric unit contains two independent molecules of [(29-THQ)·DCM], only one is shown here. All hydrogen atoms, and crystallising molecule of DCM have been omitted for clarity.

Crystallographic data for compounds **5**, **25**, **27-Me**, **27-THQ**, **28-THQ,** and **29-THQ** were recorded on a Rigaku Oxford Diffraction Excalibur diffractometer, at 120 K with Mo Kα radiation (λ = 0.71073 Å) equipped with Eos CCD detector.

The CrysAlisPro[7] software package was used for data collection, cell refinement and data reduction. For all datasets the CrysAlisPro software package was used for empirical absorption corrections, which were applied using spherical harmonics, implemented in SCALE3 ABSPACK scaling algorithm. All further data processing was undertaken within the Olex2 software package.[8] The molecular structures of all compounds were solved with the ShelXT[9] structure solution program using Intrinsic Phasing and refined with the ShelXL[10-12] refinement package using Least Squares minimisation. Non-hydrogen atoms were refined anisotropically. Hydrogen atoms were all located in a difference map and repositioned geometrically.

Selected crystallographic data are presented in Table S2 and S3 and full details in cif format can be obtained free of charge from the Cambridge Crystallographic Data Centre via www.ccdc.cam.uk/data_request/cif.

Table S2. Selected crystallographic data for compounds **5**, **25** and **27-THQ.**

	**5**	**25**	**27-THQ**
CCDC No	2221503	2221501	2221504
Empirical formula	$C_{12}H_{18}BBr_2N$	$C_{32}H_{52}B_2N_2O_4$	$C_{24.5}H_{24}B_2Br_4ClNO$
Formula Weight	346.90	550.429	725.16
Temperature (K)	120.01(11)	120.00(10) K	120.01(13)
Radiation	0.71073	0.71073	0.71073
Crystal system	Monoclinic	Triclinic	Monoclinic
Space group	P 2_1/c	P -1	P 2_1/c
a (Å)	15.7521(4)	9.6072(4)	17.5337(11)
b (Å)	6.96070(10)	10.0198(4)	9.0190(3)
c (Å)	12.1360(2)	10.2726(4)	18.6811(8)
α(°)	90	81.790(3)	90
β (°)	90.573(2)	67.850(4)	117.156(6)
γ (°)	90	63.025(4)	90
Cell volume (Å^3)	1330.59(4)	815.76(7)	2628.5(3)
Z	4	1	4
ρcalc (gcm^{-3})	1.732	1.120	1.832
μ (mm^{-1})	6.065	0.071	6.245
F (000)	688.0	300.172	1412.0
Crystal size/ mm^3	0.514 x 0.259 x 0.095	0.269 x 0.101 x 0.048	0.280 x 0.222x 0.043
2θ range for data collection/°	6.716 to 58.958	3.84 to 29.12	6.522 to 58.682
Index ranges	$-21 \leq h \leq 21$; $-9 \leq k \leq 9$; $-16 \leq l \leq 15$	$-13 \leq h \leq 13$; $-12 \leq k \leq 13$; $-13 \leq l \leq 14$	$-23 \leq h \leq 21$; $-11 \leq k \leq 11$; $-24 \leq l \leq 24$
Reflections collected	28453	21826	29847
Independent reflections	3401 [R_{int} = 0.0387; R_{sigma} = 0.0268]	4116 [R_{int} = 0.0415; R_{sigma} = 0.0383]	6372 [R_{int} = 0.0517; R_{sigma} = 0.0591]
Data/restraints/parameters	3401/0/149	4116/0/415	6372/56/319
Goodness-of-fit-on F^2 (*GOF*)	1.047	1.0975	1.013
Final *R* indices [$I>2\sigma(I)$]	R_1 = 0.0236; wR_2 = 0.0485	R_1 = 0.0340; wR_2 = 0.0494	R_1 = 0.0497; wR_2 = 0.0992
R indices (all data)	R_1 = 0.0343; wR_2 = 0.0511	R_1 = 0.0514; wR_2 = 0.0538	R_1 = 0.0913; wR_2 = 0.1163
Largest diff. peak and hole (e Å^{-3})	0.61 / -0.38	0.24 / -0.23	1.96 / -1.26
Flack parameter			

Table S3. Selected crystallographic data for compounds **29-THQ, 27-Me** and **25**.

	**28-THQ**	**29-THQ**	**27-Me**
CCDC No	2221506	2221505	2221502
Empirical formula	$C_{30}H_{31}B_2Br_2NO$	$C_{25}H_{27}BCl_3NO$	$C_{23}H_{23}B_2Br_4Cl_2NO$
Formula Weight	603.00	474.63	741.58
Temperature (K)	119.99(17)	120.1(5)	120.01(10)
Radiation	0.71073	0.71073	0.71073
Crystal system	Orthorhombic	Triclinic	Triclinic
Space group	$P\ 2_12_12_1$	P -1	P -1
a (Å)	9.83580(10)	11.9253(5)	7.3365(2)
b (Å)	11.60820(10)	13.1059(8)	12.5616(4)
c (Å)	22.5888(2)	16.0617(6)	15.3464(4)
α(°)	90	86.088(4)	107.814(3)
β (°)	90	72.721(3)	93.035(2)
γ (°)	90	77.089(4)	97.768(2)
Cell volume (Å^3)	2579.10(4)	2336.4(2)	1327.45(7)
Z	4	4	2
ρcalc (gcm^{-3})	1.553	1.349	1.855
μ (mm^{-1})	3.168	0.410	6.283
F (000)	1224.0	992.0	720.0
Crystal size/ mm^3	0.355 x 0.339 x 0.261	0.380 x 0.245 x 0.109	0.253 x 0.143 x 0.124
2θ range for data collection/°	7.02 to 58.99	6.644 to 58.824	6.902 to 58.652
Index ranges	$-13 \leq h \leq 13$; $-15 \leq k \leq 15$; $-31 \leq l \leq 30$	$-16 \leq h \leq 16$; $-18 \leq k \leq 17$; $-21 \leq l \leq 21$	$-9 \leq h \leq 9$; $-17 \leq k \leq 15$; $-20 \leq l \leq 20$
Reflections collected	57746	18790	29297
Independent reflections	6623 [R_{int} = 0.0380; R_{sigma} = 0.0275]	18790 [R_{int} = 0582; R_{sigma} = 0.0860]	6512 [R_{int} = 0.0351; R_{sigma} = 0.0364]
Data/restraints/parameters	6623/0/329	18790/0/568	6512/1/316
Goodness-of-fit-on F^2 (*GOF*)	1.050	0.961	1.065
Final *R* indices [I>2σ(I)]	R_1 = 0.0256; wR_2 = 0.0557	R_1 = 0.0479; wR_2 = 0.0953	R_1 = 0.0288; wR_2 = 0.0555
R indices (all data)	R_1 = 0.0312; wR_2 = 0.0576	R_1 = 0.0800; wR_2 = 0.1004	R_1 = 0.0445; wR_2 = 0.0597
Largest diff. peak and hole (e Å^{-3})	0.40 / -0.46	0.58 / -0.55	0.52 / -0.48
	0.494(8)		

9 Computational details

All calculations were performed using the Gaussian09 series of programs.[13] Geometries were optimized with the DFT method using M06-2X functional[14] with a 6-311G(d,p) basis set. All geometry optimizations were full, with no restrictions. All stationary points were characterized as minima by vibrational analysis. Solvent effects were introduced using the self-consistent field approach, by means of the integral equation formalism polarizable continuum model (IEFPCM).[15]

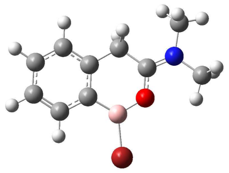

Compound 1$^+$

E(RM062X) = -3117.128717

Br	7.20757500	6.10605100	12.73864700
O	7.79243600	5.72093500	15.43531700
N	7.83885500	5.09145100	17.56411900
C	8.34209000	5.82857300	16.62935700
C	9.82845500	7.70199200	15.76126600
C	9.20741100	7.59881300	14.50836100
C	10.78422400	8.68811800	15.98661600
H	11.26402200	8.76924200	16.95568700
C	8.38040500	5.10720700	18.93144700
H	9.46595300	5.15515000	18.91314300
H	8.07514200	4.18364900	19.41556200
H	7.96991300	5.95806400	19.47662200
C	9.50412500	6.73794800	16.87710100
H	10.37431100	6.09601400	17.06831300
H	9.32142600	7.28149400	17.80781800
C	9.56080200	8.49469900	13.48916600
H	9.08116400	8.41409600	12.52050500
C	11.12009200	9.56938600	14.96683000
H	11.86304100	10.33687100	15.14796000
C	10.51094600	9.47568000	13.71530100
H	10.78024600	10.16704100	12.92711900
B	8.15223000	6.51249300	14.34427300
C	6.67933200	4.21158600	17.34727300
H	5.94317700	4.44128400	18.11622500
H	7.01053700	3.17912900	17.45242400
H	6.25889000	4.37523500	16.36244700

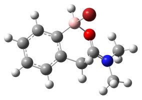

1-BH (hydride added at B)

E(RM062X) = -3117.894006

Br	9.59471900	4.69632300	13.42962900
O	7.74037400	5.75700700	15.40940400
N	7.80992500	5.20079800	17.56339300
C	8.37220900	5.77122500	16.52444500
C	9.89241700	7.58286100	15.68977900
C	9.26172000	7.53495000	14.44362700
C	10.68551900	8.66808000	16.05426400
H	11.16204900	8.69244900	17.02905600
C	8.47061400	5.11871900	18.87061400
H	9.51299100	4.82848900	18.75921400
H	7.96037800	4.35138700	19.44760400
H	8.39800800	6.07020700	19.39955200
C	9.73008700	6.40703900	16.62889900
H	10.44872500	5.61961600	16.35899700
H	9.93699400	6.70139300	17.65559000
C	9.44521000	8.60695600	13.56793200
H	8.95416700	8.59094100	12.59991400
C	10.86647700	9.72097400	15.16297000
H	11.48424900	10.56594900	15.44375800
C	10.24667200	9.68958700	13.91645600
H	10.38324300	10.51140200	13.22311200
B	8.34808300	6.28349100	14.12896600
C	6.47432600	4.60248300	17.48117700
H	5.85811200	5.02700900	18.27395100
H	6.55741900	3.52453400	17.62463100
H	6.03251400	4.81396400	16.51430200
H	7.48429100	6.40151900	13.31655600

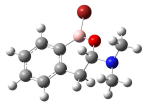

1-CH (hydride added at C)

E(RM062X) = -3117.898158

Br	6.42886400	7.06538900	13.14803500
O	7.41345000	6.11180700	15.60298000
N	7.60641200	5.28997100	17.74597300
C	8.36890700	5.84442900	16.65269700
C	9.72884700	7.78769000	15.81518300
C	9.05597700	7.72773800	14.58307200
C	10.92288200	8.49728100	15.91365800
H	11.44295300	8.54794100	16.86425600
C	8.41511600	5.10140500	18.94671500
H	8.71280700	6.05808800	19.37414000
H	9.31776700	4.49679700	18.75685900
H	7.81023700	4.58083400	19.68912400
C	9.12032000	7.11474500	17.02465600
H	9.91042100	6.87706300	17.73734700
H	8.41495500	7.79272000	17.52011600
C	9.59577800	8.37877100	13.46972800
H	9.07337300	8.33260700	12.52029300
C	11.45113000	9.13629300	14.79672500
H	12.38452700	9.68065300	14.88181600
C	10.79123600	9.07722400	13.57104800
H	11.20916600	9.57462200	12.70441800
B	7.73681700	6.92451300	14.59216900
C	6.99825200	4.02015000	17.35155300
H	6.35459100	3.66828700	18.15821800
H	7.75817700	3.24774000	17.14701800
H	6.39131900	4.16119200	16.45968000
H	9.07709700	5.10407500	16.23708700

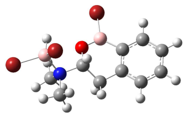

Compound 3

E(RM062X) = -8291.805289

C	-6.79001600	-0.64188100	-0.14665300
C	-5.40870700	-0.66916400	0.01799400
C	-4.69576700	0.52189700	0.11187900
C	-5.37444000	1.75026000	0.04732200
C	-6.76384600	1.76132600	-0.11176100
C	-7.47109500	0.57153500	-0.21282500
H	-2.99582400	0.53192400	1.41147100
H	-7.33746700	-1.57370200	-0.22593000
H	-4.88648000	-1.61819700	0.06995600
C	-3.19594800	0.50959800	0.33575300
H	-7.28692400	2.71003800	-0.16035100
H	-8.54612800	0.58463800	-0.34240000
C	-2.55980200	1.70311100	-0.36503900
C	-0.83439300	2.35182000	1.27833500
H	0.22858900	2.27157100	1.48471600
H	-1.15395300	3.38677600	1.36071200
H	-1.38992100	1.72809500	1.97572800
C	-0.38871700	0.55799400	-0.30272800
H	-0.61799200	-0.08577800	0.54439100
H	-0.72000100	0.10039600	-1.23227100
H	0.68250800	0.73969000	-0.34791900
H	-2.78232700	-0.41601900	-0.06350600
H	-2.67506400	1.59452900	-1.44496600
B	-4.49220600	3.00816000	0.16571200
B	-0.51442300	3.00680200	-1.15602200
H	-1.15497700	3.99010100	-1.02306400
O	-3.15040100	2.92325500	0.03818800

Br	1.44113200	3.40305100	-0.75690700
Br	-0.68276700	2.32259200	-3.07106900
N	-1.07290100	1.87506100	-0.12366000
Br	-5.19885500	4.77404800	0.52774500

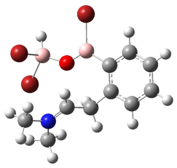

Compound 3-O

E(RM062X) = -8291.811568

C	-6.73004600	-0.65324300	0.08464000
C	-5.42727500	-0.46897100	0.53560100
C	-4.81432000	0.78007200	0.47533700
C	-5.52162200	1.88507600	-0.05326000
C	-6.83211400	1.67097400	-0.49943900
C	-7.43679200	0.42204800	-0.43596100
H	-3.30627600	1.66966100	1.75794000
H	-7.18616900	-1.63435400	0.14308900
H	-4.88005800	-1.31153800	0.94395400
C	-3.39604500	0.88169400	1.00222000
H	-7.39096900	2.50555800	-0.90471200
H	-8.45183800	0.29147300	-0.79033000
C	-2.39494900	1.17707300	-0.06603600
C	-0.50758300	0.85734300	1.41322500
H	-0.21042600	-0.19251200	1.42138300
H	0.36915600	1.49329100	1.51258100
H	-1.19606800	1.06988200	2.22457200
C	-0.18271600	1.43720100	-0.96915800
H	0.53583500	0.61942100	-1.01586400
H	-0.71976800	1.53143800	-1.90838500
H	0.32486300	2.37400300	-0.73654100

H	-3.11880100	-0.05919600	1.48570500
H	-2.73387900	1.40491200	-1.07266700
B	-4.86418800	3.30290000	-0.15459900
O	-3.60083800	3.49806700	0.19740900
N	-1.12953800	1.15402200	0.11655000
B	-2.70274600	4.61774800	0.12347900
Br	-5.91200100	4.82125300	-0.85235500
Br	-1.21906700	4.27209000	1.54792000
Br	-1.81608500	4.57695900	-1.76157400
H	-3.15195000	5.69887400	0.31053800

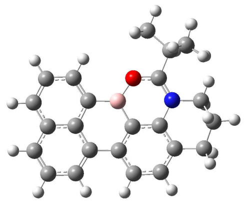

Compound **D**

E(RM062X) = -1083.001006

O	-0.94570500	4.06351200	10.30002500
N	0.03183400	2.80205400	8.67115900
C	-6.14224100	2.53713000	8.64058000
H	-6.26857000	1.83837200	7.82459100
C	-4.75416000	3.84089900	10.13526900
C	-0.05198600	0.71212900	6.57526700
H	0.14241400	1.17454700	5.60174600
H	-0.23043200	-0.34924800	6.39676100
C	1.42024100	4.23015400	10.26073000
C	-4.87464700	2.90847300	9.04751700
C	-5.93435600	4.34247200	10.74928500
C	1.22114800	2.39211000	7.87369000
H	2.10954700	2.58358800	8.45231100
H	1.23350400	3.02522800	6.98284900

C	1.04315200	5.22048100	11.37870600
H	0.44871900	6.05053100	10.99569500
H	1.96968800	5.62106300	11.79148400
H	0.48889500	4.73469700	12.18135600
C	-3.67487400	2.35488100	8.37210000
C	-4.60189300	5.67951800	12.28779000
H	-4.53777000	6.38145100	13.10950300
C	-1.23066600	2.28576400	8.19938000
C	2.21072300	5.03337800	9.20628800
H	2.68734600	4.43327900	8.43581400
H	2.99993100	5.57442800	9.73076000
H	1.56546800	5.77062500	8.72383200
C	-7.20846700	3.92657900	10.28964800
H	-8.09562600	4.32253000	10.77077900
C	-3.48839200	4.29071500	10.63187800
C	2.25723100	3.11039600	10.91584100
H	1.67087500	2.59643300	11.67987200
H	3.11214800	3.57895900	11.40660500
H	2.64421300	2.36313700	10.22541900
C	-7.30645300	3.03930900	9.25272400
H	-8.27520800	2.71513000	8.89410400
C	-3.72659600	1.41883500	7.33438100
H	-4.66665200	1.04315900	6.95397500
C	-2.55583100	0.92605000	6.77846900
H	-2.62385800	0.17987600	5.99391400
C	0.12652600	3.64695400	9.67976100
C	-2.40061500	2.77458100	8.78864500
C	-1.28118400	1.32748400	7.18627300
C	-3.43154900	5.18821100	11.68401900
H	-2.46388000	5.51993800	12.04717300
C	-5.82413700	5.26127600	11.82394500

H	-6.73751500	5.63138000	12.27756400
C	1.13859100	0.92582000	7.49364900
H	1.04833900	0.31928300	8.39899000
H	2.07113200	0.65288000	6.99930500
B	-2.26189900	3.73567000	9.93098200

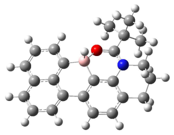

D-BH (hydride added at B)

E(RM062X) = -1083.731760

O	-0.91817900	3.96458500	10.39085600
N	0.03886600	2.62692000	8.82030100
C	-6.12959600	2.39552300	8.76191900
H	-6.24984400	1.66158700	7.97528000
C	-4.74298200	3.70810900	10.26225800
C	-0.06973300	1.04247700	6.36011600
H	0.28003100	1.76820600	5.61724800
H	-0.29851700	0.11860100	5.82604000
C	1.40574900	4.29015400	10.19659900
C	-4.86709900	2.76879800	9.17337600
C	-5.92486700	4.31899300	10.77470600
C	1.25687200	2.14209700	8.12709900
H	2.04415300	2.02402700	8.86376300
H	1.56483600	2.88982800	7.39398800
C	1.00194600	5.56985900	10.95322500
H	0.46351700	6.25977300	10.30043300
H	1.91313500	6.05933800	11.30050400
H	0.37425800	5.34886700	11.81420300
C	-3.65630100	2.29254100	8.44584900
C	-4.58693000	5.67564800	12.26760500

H	-4.50580200	6.44532600	13.02735400
C	-1.24690800	2.21377500	8.27800000
C	2.34368000	4.71951900	9.05200300
H	2.98996500	3.92244900	8.69291900
H	2.99507800	5.50884800	9.43040800
H	1.78485800	5.13164700	8.20817700
C	-7.19829800	3.91787300	10.29253000
H	-8.08621400	4.37882100	10.71213300
C	-3.47506300	4.03289200	10.84322400
C	2.12604200	3.36581600	11.19941800
H	1.46891600	3.12909800	12.03892300
H	3.00081400	3.89286900	11.58689900
H	2.46924400	2.43000600	10.75747000
C	-7.29571500	2.95368500	9.32821700
H	-8.26614100	2.62572400	8.97447900
C	-3.73285500	1.67037300	7.19231600
H	-4.68622200	1.48475000	6.71421300
C	-2.57772000	1.27507300	6.53797900
H	-2.64942300	0.75272800	5.58973700
C	0.11014900	3.58211000	9.75549200
C	-2.39451300	2.53028500	8.99799500
C	-1.30878800	1.53971600	7.05426300
C	-3.42623100	5.01355000	11.81041100
H	-2.46797500	5.26956200	12.25399800
C	-5.81454400	5.31677200	11.77769400
H	-6.72126300	5.78368500	12.14799500
C	1.01113100	0.82900800	7.40889200
H	0.70328100	0.05862500	8.12108100
H	1.95295400	0.51496000	6.95652800
B	-2.22251700	3.13597700	10.44186400
H	-2.01559700	2.26268500	11.27070500

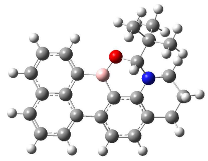

D-CH (hydride added at C)

E(RM062X) = -1083.764528

O	-0.93230100	4.35584100	10.12485900
N	0.06049300	2.73937300	8.68896400
C	-6.02680600	2.10894600	9.15687500
H	-6.10128800	1.15511400	8.64984600
C	-4.72581400	3.97126900	9.99754600
C	-0.04749200	0.88785500	6.43187500
H	-0.11007100	1.35515200	5.44274400
H	-0.05792600	-0.19233300	6.26603300
C	1.40169700	3.91796400	10.54542000
C	-4.80056900	2.73295200	9.26769200
C	-5.92368500	4.54145600	10.51266600
C	1.10336300	2.73431100	7.66681000
H	2.04181200	3.06715700	8.08782100
H	0.84100100	3.42889900	6.85741600
C	1.10480100	4.39975100	11.97816700
H	0.40579900	5.23573100	11.97539000
H	2.03545000	4.72371000	12.44993600
H	0.67882200	3.59503700	12.58400800
C	-3.59384200	2.15416500	8.61862400
C	-4.66932300	6.42311100	11.40242100
H	-4.63798700	7.36630000	11.93456800
C	-1.16276200	2.19838500	8.30908100
C	1.88389000	5.13667100	9.74707700
H	2.23353100	4.90072800	8.74307800
H	2.71193100	5.61124700	10.28026100
H	1.07507900	5.86518800	9.66193300

C	-7.16130500	3.86964300	10.34516900
H	-8.06171900	4.31752800	10.75122700
C	-3.48339800	4.64051500	10.24119000
C	2.46501900	2.81574600	10.65967300
H	2.09996400	2.00782800	11.30027100
H	3.36271200	3.22931600	11.12582800
H	2.76171400	2.37413200	9.70924200
C	-7.20507100	2.66771700	9.69351700
H	-8.14341600	2.13865100	9.57929400
C	-3.68154000	1.21393700	7.58987000
H	-4.63590700	0.83056800	7.25359100
C	-2.52704900	0.80840300	6.92426700
H	-2.61601000	0.09518600	6.10964800
C	0.08346800	3.33695100	10.00771900
C	-2.32306100	2.63185600	8.98388800
C	-1.26193000	1.29369200	7.23546800
C	-3.47708800	5.83548600	10.93205400
H	-2.52773100	6.32467300	11.12684500
C	-5.86341100	5.78023400	11.20230800
H	-6.78844600	6.20696300	11.57624900
C	1.25136400	1.31916000	7.11050900
H	1.48901100	0.64968800	7.94240900
H	2.08085100	1.28194800	6.40169400
B	-2.18677300	3.90524900	9.82246800
H	-0.22115900	2.54440400	10.70798700

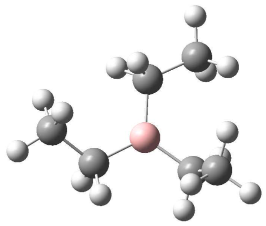

BEt_3

E(RM062X) = -262.461001

B	0.56583500	0.93255200	-0.37784000
C	-0.15248800	-0.38234500	-0.86246700
H	0.38962800	-1.28313600	-0.55767100
H	-1.18031100	-0.46802900	-0.49801800
C	-0.17671300	-0.32081200	-2.40915100
H	0.83561200	-0.25862500	-2.81751000
H	-0.65019500	-1.20823800	-2.83348100
H	-0.72954800	0.55266000	-2.76558100
C	-0.22245800	2.28523400	-0.20098400
H	0.09472300	2.74533900	0.74488700
H	0.17682100	2.97341000	-0.96253900
C	-1.75036200	2.23720000	-0.27989600
H	-2.19529000	3.23058000	-0.18705000
H	-2.08744300	1.81172000	-1.22868200
H	-2.16444200	1.61479500	0.51727200
C	2.12482300	0.89112800	-0.15018500
H	2.23740000	0.39422400	0.82805700
H	2.58329700	0.18759000	-0.85673100
C	2.88584800	2.21995500	-0.14093500
H	3.94742700	2.08117600	0.07548800
H	2.80941700	2.72093400	-1.10963800
H	2.48246300	2.90418200	0.60939900

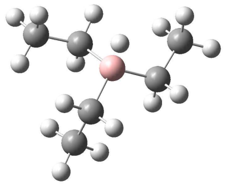

HBEt$_3$

E(RM062X) = -263.151665

B	0.53261300	0.92110300	-0.03794000
H	0.37038300	0.83139300	1.20319600
C	-0.16532300	-0.40142100	-0.73693000
H	0.37440600	-1.31115100	-0.43071400
H	-1.19245300	-0.54028500	-0.36832200
C	-0.21225600	-0.35944900	-2.27174100
H	0.79134300	-0.24111600	-2.69387700
H	-0.64949500	-1.25922200	-2.72147300
H	-0.80228800	0.49610800	-2.61854700
C	-0.18153500	2.31657200	-0.54338200
H	0.24292000	3.17727900	-0.00632800
H	0.05269600	2.49860900	-1.60592900
C	-1.70505200	2.34630900	-0.37104000
H	-2.16637800	3.29373900	-0.67598800
H	-2.18178200	1.55044500	-0.95330600
H	-1.97587200	2.17332800	0.67683600
C	2.15273100	0.94242000	-0.33337600
H	2.61129000	0.01594100	0.04658600
H	2.34287600	0.93538400	-1.41897700
C	2.90088700	2.13776400	0.27050100
H	3.98798300	2.09509900	0.13047900
H	2.55512400	3.07779500	-0.17078100
H	2.71111700	2.20786000	1.34779700

10 References

[1] Two Directing Groups Used for Metal Catalysed Meta-C–H Functionalisation Only Effect Ortho Electrophilic C–H Borylation. S. A. Iqbal, C. R. P. Millet, J. Pahl, K. Yuan, M. J. Ingleson, *Eur. J. Org. Chem.*, **2022**, e202200901.

[2] Acyl Directed ortho-Borylation of Anilines and C7 Borylation of Indoles using just BBr_3. S. A. Iqbal, J. Cid, R. J. Procter, M. Uzelac, K. Yuan and M. J. Ingleson, *Angew. Chem. Int. Ed.*, **2019**, *58*, 15381–15385.

[3] Ultralong blue room-temperature phosphorescence by cycloalkyl engineering. Z. Cong, M. Han, Y. Fan, Y.Fan, K. Chang, L. Xiao, Y. Zhang, X. Zhen, Q. Li, Z. Li, *Mater. Chem. Front*. **2022**, *6*, 1606-1614.

[4] Synthesis of substituted benzooxaborinin-1-ols via palladium-catalysed cyclisation of alkenyl- and alkynyl-boronic acids. L. Benhamou, D. W. Walker, D. K. Bučar, A. E. Aliev, T. D. Sheppard, *Org. Biomol. Chem*. **2016**, *14*, 8039–8043.

[5] Zinc-catalyzed reduction of amides: unprecedented selectivity and functional group tolerance. S. Das, D. Addis, S. Zhou, K. Junge, M. Beller, *J. Am. Chem. Soc.* **2010**, *132*, 1770-1771.

[6] Synthesis of pyridobenzazepines using a one-pot Rh/Pd-catalyzed process. H. Lam, J. Tsoung, M. Lautens, *J. Org. Chem.* **2017**, *82*, 6089-6099.

[7] CrysAlisPro, Agil. Technol. Version 1.1 71.35.19 (release 27-10-2011 CrysAlis171.NET) (compiled Oct 27 2011,150211).

[8] OLEX2: a complete structure solution, refinement and analysis program. O. V. Dolomanov, L. J. Bourhis, R. J. Gildea, J. A. K. Howard, H. Puschmann, *J. Appl. Cryst.* **2009**, *42*, 339 – 341.

[9] *SUPERFLIP*- a computer program for the solution of crystal structures by charge flipping in arbitrary dimensions. L. Palatinus, G. Chapuis, *J. Appl. Cryst.* **2007**, *40*, 786 – 790.

[10] Symmetry determination following structure solution in P1. L. Palatinus, A. Van der Lee, *J. Appl. Cryst.* **2008**, *41*, 975 – 984.

[11] EDMA: a computer program for topological analysis of discrete electron densities. L. Palatinus, S. J. Prathapa, S. Van Smaalen, *J. Appl. Cryst.* **2012**, *45*, 575 – 580.

[12] A short history of SHELX. G. M. Sheldrick, *Acta Cryst.* **2008**, *A64*, 112 – 122.

[13] Gaussian 09, Revision E.01, M. J. Frisch, G. W. Trucks, H. B. Schlegel, G. E. Scuseria, M. A. Robb, J. R. Cheeseman, G. Scalmani, V. Barone, B. Mennucci, G. A. Petersson, H. Nakatsuji, M. Caricato, X. Li, H. P. Hratchian, A. F. Izmaylov, J. Bloino, G. Zheng, J. L. Sonnenberg, M. Hada, M. Ehara, K. Toyota, R. Fukuda, J. Hasegawa, M. Ishida, T. Nakajima, Y. Honda, O. Kitao, H. Nakai, T. Vreven, J. A. Montgomery, Jr., J. E. Peralta, F. Ogliaro, M. Bearpark, J. J. Heyd, E. Brothers, K. N. Kudin, V. N. Staroverov, T. Keith, R. Kobayashi, J. Normand, K. Raghavachari, A. Rendell, J. C. Burant, S. S. Iyengar, J. Tomasi, M. Cossi, N. Rega, J. M. Millam, M. Klene, J. E. Knox, J. B. Cross, V. Bakken, C. Adamo, J. Jaramillo, R. Gomperts,

R. E. Stratmann, O. Yazyev, A. J. Austin, R. Cammi, C. Pomelli, J. W. Ochterski, R. L. Martin, K. Morokuma, V. G. Zakrzewski, G. A. Voth, P. Salvador, J. J. Dannenberg, S. Dapprich, A. D. Daniels, O. Farkas, J. B. Foresman, J. V. Ortiz, J. Cioslowski, and D. J. Fox, Gaussian, Inc., Wallingford CT, **2013**.

[14] The M06 suite of density functionals for main group thermochemistry, thermochemical kinetics, noncovalent interactions, excited states, and transition elements: two new functionals and systematic testing of four Mo6-class functionals and 12 other functionals. Zhao Y.; Truhlar D. G., *Theor. Chem. Acc.* **2008**, *120*, 215-241.

[15] Evaluation of Solvent Effects in Isotropic and Anisotropic Dielectrics and in Ionic Solutions with a Unified Integral Equation Method: Theoretical Bases, Computational Implementation, and Numerical Applications. Mennucci B.; Cancès E.; Tomasi J., *J. Phys. Chem. B* **1997**, *101*, 10506-10517.
